# Schwann cell precursors represent a neural crest‐like state with biased multipotency

**DOI:** 10.15252/embj.2021108780

**Published:** 2022-07-11

**Authors:** Maria Eleni Kastriti, Louis Faure, Dorothea Von Ahsen, Thibault Gerald Bouderlique, Johan Boström, Tatiana Solovieva, Cameron Jackson, Marianne Bronner, Dies Meijer, Saida Hadjab, Francois Lallemend, Alek Erickson, Marketa Kaucka, Viacheslav Dyachuk, Thomas Perlmann, Laura Lahti, Jan Krivanek, Jean‐Francois Brunet, Kaj Fried, Igor Adameyko

**Affiliations:** ^1^ Department of Molecular Neuroscience, Center for Brain Research Medical University Vienna Vienna Austria; ^2^ Department of Physiology and Pharmacology Karolinska Institutet Stockholm Sweden; ^3^ Department of Neuroimmunology, Center for Brain Research Medical University Vienna Vienna Austria; ^4^ Division of Biology and Biological Engineering California Institute of Technology Pasadena CA USA; ^5^ Centre for Discovery Brain Sciences University of Edinburgh Edinburgh UK; ^6^ Department of Neuroscience Karolinska Institutet Stockholm Sweden; ^7^ Max Planck Institute for Evolutionary Biology Plön Germany; ^8^ Almazov Federal Medical Research Centre Saint Petersburg Russia; ^9^ Department of Cell and Molecular Biology Karolinska Institutet Stockholm Sweden; ^10^ Department of Histology and Embryology, Faculty of Medicine Masaryk University Brno Czech Republic; ^11^ Institut de Biologie de l'ENS (IBENS), INSERM, CNRS, École Normale Supérieure PSL Research University Paris France

**Keywords:** multipotency, neural crest, regulons, Schwann cell precursors, Schwann cell lineage, Development, Neuroscience

## Abstract

Schwann cell precursors (SCPs) are nerve‐associated progenitors that can generate myelinating and non‐myelinating Schwann cells but also are multipotent like the neural crest cells from which they originate. SCPs are omnipresent along outgrowing peripheral nerves throughout the body of vertebrate embryos. By using single‐cell transcriptomics to generate a gene expression atlas of the entire neural crest lineage, we show that early SCPs and late migratory crest cells have similar transcriptional profiles characterised by a multipotent “hub” state containing cells biased towards traditional neural crest fates. SCPs keep diverging from the neural crest after being primed towards terminal Schwann cells and other fates, with different subtypes residing in distinct anatomical locations. Functional experiments using CRISPR‐Cas9 loss‐of‐function further show that knockout of the common “hub” gene *Sox8* causes defects in neural crest‐derived cells along peripheral nerves by facilitating differentiation of SCPs towards sympathoadrenal fates. Finally, specific tumour populations found in melanoma, neurofibroma and neuroblastoma map to different stages of SCP/Schwann cell development. Overall, SCPs resemble migrating neural crest cells that maintain multipotency and become transcriptionally primed towards distinct lineages.

## Graphical abstract

A transcriptional, temporal and spatial single‐cell profiling resource uncovers Schwann cell progenitors as a multipotent source of neural crest‐like cells in the developing mouse embryo.

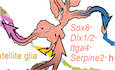

## Introduction

Schwann cell precursors (SCPs) represent a nerve‐associated embryonic cell type that spreads throughout the body using peripheral nerves as navigational scaffolds during vertebrate embryonic development (Furlan & Adameyko, 2018). SCPs give rise to melanocytes, peripheral neurons, Schwann, neuroendocrine and mesenchymal cells (Joseph *et al*, 2004; Adameyko *et al*, 2009; Nitzan *et al*, 2013; Dyachuk *et al*, 2014; Kaukua *et al*, 2014; Uesaka *et al*, 2015; Espinosa‐Medina *et al*, 2017; Furlan *et al*, 2017; Kastriti *et al*, 2019). While initially regarded as progenitors of Schwann cells (SCs) prior to fate restriction towards myelination (Jessen & Mirsky, 1992), both *in vitro* and *ex vivo* studies revealed that these nerve‐associated cells can give rise to many downstream fates (Ciment *et al*, 1986; Morrison *et al*, 1999; Dupin *et al*, 2000). Thus, SCPs are multipotent *in vivo* and associated with essential developmental functions beyond myelination and support of axonal homeostasis within the peripheral nervous system.

Schwann cell precursors arise from neural crest (NC) cells that settle on outgrowing peripheral nerves (Weston, 1963). They spread throughout the developing body via branching innervation, detach from the nerves in specific locations and produce large quantities of pigment cells, autonomic and enteric neurons, chromaffin cells of the adrenal medulla and specific mesenchymal populations within nerves and cranial locations (Joseph *et al*, 2004; Adameyko *et al*, 2009; Nitzan *et al*, 2013; Dyachuk *et al*, 2014; Uesaka *et al*, 2015; Espinosa‐Medina *et al*, 2017; Furlan *et al*, 2017; Kastriti *et al*, 2019). Based on this wide array of SCP‐derived fates, SCPs can be viewed as the nerve‐associated state of neural crest‐like cells that persists into later developmental stages. In line with this concept, we suggested that the evolutionary ancient NC‐like cells navigated via the nerves in early chordates and may have resembled SCPs (Ivashkin & Adameyko, 2013). Experimental support for this hypothesis comes from lamprey and zebrafish, where enteric neurons are generated from the NC‐derived nerve‐associated SCPs navigating via the trunk nerves to the gut of developing larvae (Green *et al*, 2017; El‐Nachef & Bronner, 2020).

In adults, SCPs and nerve‐associated peripheral glial cells may represent a source of the adult NC‐like stem cells residing in numerous tissues. For example, the skin contains multipotent skin‐derived precursor cells (SKP cells; Toma *et al*, 2001; Fernandes *et al*, 2004; Wong *et al*, 2006). Similarly, peripheral glia associated with innervation are known to release signals that stimulate the formation of the regenerating limb blastema (Lehoczky *et al*, 2011; Johnston *et al*, 2016; Carr *et al*, 2019; Storer *et al*, 2020). Thus, identifying adult cells capable of reverting to the NC‐like multipotency has potentially important implications for regenerative medicine.

The fact that SCPs generate diverse cell types in particular locations raises questions about what determines their cell fate choice. For instance, SCPs covering preganglionic autonomic motor nerves generate chromaffin cells near the dorsal aorta, whereas SCPs associated with sensory fibres innervating the skin generate melanocytes and specialised sensory skin end organs (Adameyko *et al*, 2009; Furlan *et al*, 2017; Harty & Monk, 2017; Lumb *et al*, 2018; Abdo *et al*, 2019; Jessen & Mirsky, 2019). This suggests that SCPs might be intrinsically heterogeneous and become fate‐restricted or biased by unknown early cues generated by their local environment. This is consistent with the mechanisms that drive sympathoadrenal differentiation via CXCL12 and BMP signals secreted by dorsal aorta (Saito *et al*, 2012) and by signalling factors inducing melanocyte formation (WNTs, EDN3, KIT ligand) from the NC lineage (Baynash *et al*, 1994; Hosoda *et al*, 1994; Reid *et al*, 1996; Dorsky *et al*, 1998; Dunn *et al*, 2000; Hou *et al*, 2000; Lewis *et al*, 2004). Understanding the spatiotemporal heterogeneity of SCPs is key to answering these mechanistic questions.

In the present study, we employ single‐cell transcriptomic analysis to generate the atlas of the neural crest lineage and elucidate SCP identity, heterogeneity and similarity to the NC and NC‐derived cell types in cancer. Our results show that early SCPs and late neural crest cells (NCCs) share a multipotent “hub” state containing differentially biased cells. We further find that loss‐of‐function of the “hub” gene *Sox8* in the trunk neural crest affects SCPs along the nerve and sympathoadrenal cell fate choice. Once SCPs are driven towards the SC lineage, they diverge from the NC‐like state via orchestrated transcriptional activation, with spatial heterogeneity and sets of genes biasing them to myelinating and non‐myelinating SCs; terminal SCs of neuromuscular junctions and endoneurial fibroblasts. Finally, the comparisons of SCP transcriptional profiles with cells from melanoma, neuroblastoma and neurofibromatosis type 1 predict the reactivation of embryonic‐like cell states.

## Results

### Migratory neural crest cells transit into Schwann cell precursors via a joint multipotent “hub” transcriptional state

**Figure 1:**
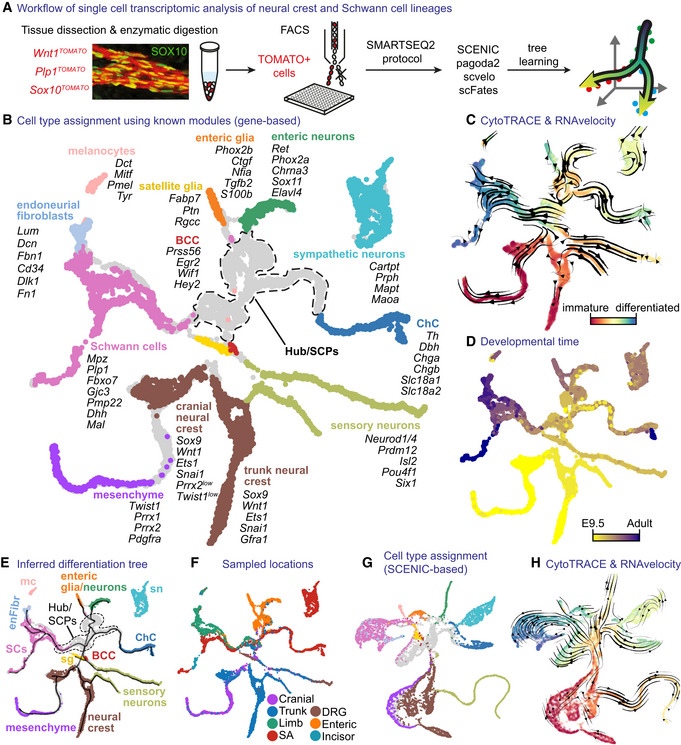
Transcriptomic analysis of neural crest, Schwann cell precursors and their downstream lineages AOverview of single‐cell sampling, transcriptomic analysis and developmental tree assembly.BGene expression‐based UMAP embedding. Cells assigned to defined cell types using shown markers.CUMAP with CytoTRACE values and overlaid RNA velocity‐derived vector streams.DUMAP with developmental stage.EUMAP embedding as in (B) with overlaid learned tree.FUMAP embedding with sampled locations.GUMAP embedding based on SCENIC activity scores (same colouring as B).HSCENIC‐based UMAP with the same information as (C). Data information: BCC, boundary cap cells; ChC, chromaffin cells; DRG, dorsal root ganglia; Mc, melanocytes; SA, sympathoadrenal; SCs, Schwann cells; sn, sympathetic neurons.

**Figure EV1:**
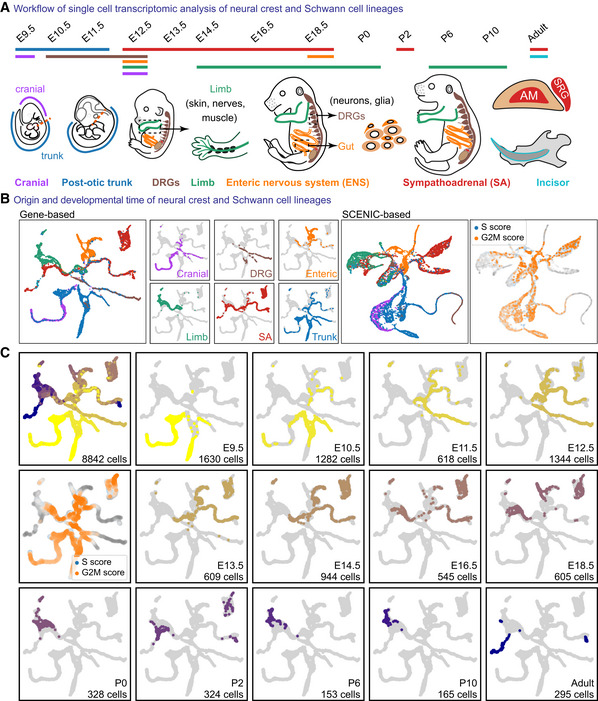
Overview and composition of neural crest and Schwann cell data set AOverview of the sampled locations and time points in mice. AM, adrenal medulla; SRG, suprarenal ganglion.BColour‐coded projections of the tissue of origin, including SCENIC regulon‐based UMAP embedding, with cell cycle shown (right).CUMAP embeddings colour‐coded according to the developmental stage, with cell cycle shown.

To examine the spatiotemporal heterogeneity of the developing neural crest and SC lineage, we used the Smartseq2 single‐cell transcriptomics approach, which allows identifying the expression of 7,000–8,000 genes per cell on average (Fig 1A and Appendix Fig S1). We combined our newly generated data corresponding to a range of developmental and postnatal stages (from E9.5 to adult) and multiple body locations (Fig EV1) with previously generated data sets of mouse NC populations (Soldatov *et al*, 2019). Neural crest‐derived cells were obtained via Cre‐based labelling with the constitutively active *Wnt1‐Cre;R26* ^*TOMATO*^ and tamoxifen‐inducible *Plp1* ^*CreERT2*^;*R26* ^*TOMATO*^ and *Sox10* ^*CreERT2*^;*R26* ^*TOMATO*^ transgenes, with recombination induced 48 h prior to tissue collection (Fig 1A). Additionally, enrichment of sensory neurons was achieved using *Isl1* ^*Cre*^;*R26* ^*TOMATO*^ from newly generated and already published data sets (Faure *et al*, 2020). Starting from E12.5 onwards, we separately examined anatomical regions of the developing embryos to discern regional differences between sympathoadrenal, sensory, SCPs, melanocytes and enteric NC lineages (Fig EV1B). After computational clean‐up of contaminating populations using published markers (including predelaminating neural crest based on neural tube signature defined as *Olig3* ^+^/*Sox2* ^+^/*Msx1* ^+^ cells; Simoes‐Costa & Bronner, 2015; Tabula Muris *et al*, 2018) and low‐quality transcriptomes (Appendix Figs S1 and S2), we recovered the transcriptomes of 8,842 cells covering embryonic and postnatal stages, different locations and neural crest‐derived fates (Fig EV1A–C). The resulting atlas is available for data mining online via internet browser (please see “Data availability” section).

Next, we processed the data set via pagoda2 and SCENIC pipelines with joint UMAP embedding based on: (i) multiscale diffusion space derived from PCA space (generated by gene‐based pagoda2); (ii) multiscale diffusion space derived from regulon activity scores (AUC, generated by SCENIC) showing striking similarities in their structure (Fig 1B–H). This observation suggested that the main differentiation paths of the NC lineage could be abstracted at the level of transcription factor (TF) activity alone. In other words, the detected transactivation activity of identified TFs (i.e. regulons) sufficiently defined the trajectory and the structure of the data set. Therefore, we decided to base our main trajectory analysis by fitting a principal tree on the multiscale diffusion space of the SCENIC AUC regulon activity scores. Visualisations of gene expression were displayed on the UMAP embedding generated from the same diffusion space. Using the combined expression of known markers for NCCs and their derivatives (Appendix Fig S3), we first mapped the cells that clearly define the main populations on the SCENIC‐based UMAP embedding. This resulted in a pool of multipotent NCCs derived from E9.5 and E10.5 (expressing *Wnt1, Ets1, Sox9*; Wu *et al*, 2003; Yanfeng *et al*, 2003; Bronner & Simoes‐Costa, 2016) projecting through cellular streams towards multiple definitive fates (Fig 1B and C).

The structure of the NC pool revealed that cells within this population were divided into two subgroups, separated by the presence of mesenchymal bias in cranial neural crest cells (Figs 1B and EV1B). Intuitively, two parallel paths of NC differentiation converge in terms of nonmesenchymal fates at later time points. The examination of directionality of the NC and downstream nonmesenchymal populations with CytoTRACE and RNA velocity further produced a continuum of transitions and cell states towards the most differentiated transcriptional states (Fig 1C and H). This indicates that the molecular changes that govern the switch from migrating NC to nerve‐bound SCPs take place as a smooth transition of transcriptional states.

**Figure 2:**
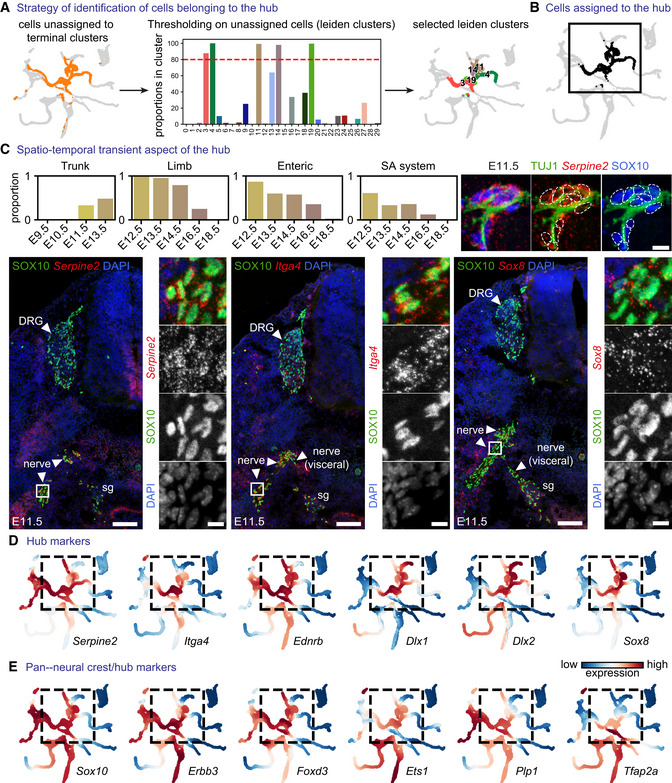
The “hub” state is a transient cell pool occupying peripheral locations with unique markers AStrategy of “hub” identification: Leiden clusters containing ≥ 80% of unassigned cells (red dashed lined demarcating the cutoff for clusters characterised by ≥ 80% of unassigned cells that were considered as “hub”).BCells assigned to the “hub.”CProportion of “hub” cells across sampling locations and developmental stage and (Cox *et al*, 2004) validation of the “hub” gene signature using SOX10 as a pan‐”hub” marker and RNAscope® *in situ* hybridization for *Serpine2*, *Itga4* and *Sox8*. Upper right: SOX10^+^ cells expressing “hub” markers associate with peripheral nerves at E11.5. Scale bar in overview pictures is 100 μm and 10 μm in insets. Scale bar for upper right subpanel = 10 μm.DMAGIC‐imputed expression of selected markers among the top 25 differentially expressed in “hub” cells.EMAGIC‐imputed expression of known neural crest markers. SA system: sympathoadrenal system.

When focusing on the root of the resulting tree of transcriptional events, we observed that one of the directions of differentiation emerging from the NC included a cell population extending towards sensory neurons (Fig 1B). This population appeared to be proliferative, consisting of the late NCCs expressing *Neurod1/4, Prdm12, Isl2 Pou4f1 and Six1* (Fig EV1B and Appendix Fig S3). In contrast to cells differentiating towards this sensory neurogenesis branch, we detected a separating pool of cycling progenitors appearing as a “hub” for downstream fates (Figs 1B and E, 2A and B, and EV1B). The “hub” cells (represented as grey in Fig 1B) mainly originated from E10.5, E11.5 and E12.5 stages (Figs 2C and EV1B) with few admixed E9.5 NCCs. The “hub” cells expressed both pan‐neural crest and SCP markers (Fig 2D and E) and spanned all peripheral locations, including mixed nerves and developing autonomic and enteric ganglia as validated experimentally with combined SOX10 immunodetection and RNAscope® for the “hub” genes *Itga4*, *Serpine2* and *Sox8* (Appendix Figs S5–S7). Even though SOX10^+^ cells representing neural crest migrate freely at E9.5, very few of them are found near the emerging axons. At E10.5, most SOX10^+^ cells become nerve (or peripheral neuron)‐associated. Starting from E11.5, all SOX10^+^ cells are associated with the outgrowing peripheral nerves or neurons consistent with previous studies (Adameyko *et al*, 2012; Dyachuk *et al*, 2014; Furlan *et al*, 2017). Thus, the late non‐sensory‐biased NCCs and nerve‐associated SCPs (at E9.5, E10.5 and E11.5) from cranial and trunk regions converge on a similar multipotent transcriptional state we refer to as “hub,” from which other subtrajectories emerge towards multiple definitive fates according to RNA velocity and trajectory analysis (Fig 1B, C and F). The dynamics of nerve association combined with the onset of detectable “hub” signature (*Itga4, Serpine2, Sox8*) by RNAscope® in neural crest cells at postdelamination stages at E9.5 (Appendix Figs S5–S7) and progressive upregulation with an observed peak at E12.5 (Fig 2C) proposes that the onset of the “hub” state initiates in late neural crest but consolidates and reaches a peak upon cell attachment onto peripheral nerves. The cells of the “hub” state differentiate towards SCs as detected via the onset of expression of *Gjc3, Plp1, Pmp22, Mpz*; (Altevogt *et al*, 2002; Jessen & Mirsky, 2005), melanocytes expressing *Mitf, Dct, Tyr, Pmel* (Steingrimsson *et al*, 2004), enteric neurons (*Hoxb5, Hoxa5, Chrnb4, Chrna3, Chodl, Elavl4*), enteric glia (*S100b, Phox2b, Tgfb2, Nfia, Ctgf*; Steingrimsson *et al*, 2004), autonomic neurons (*Prph, Cartpt, Mapt, Maoa*) and chromaffin cells (*Chga, Chgb, Th, Dbh, Slc18a1, Slc18a2*; Furlan *et al*, 2017; Kastriti *et al*, 2019; Kameneva *et al*, 2021).

**Figure 3:**
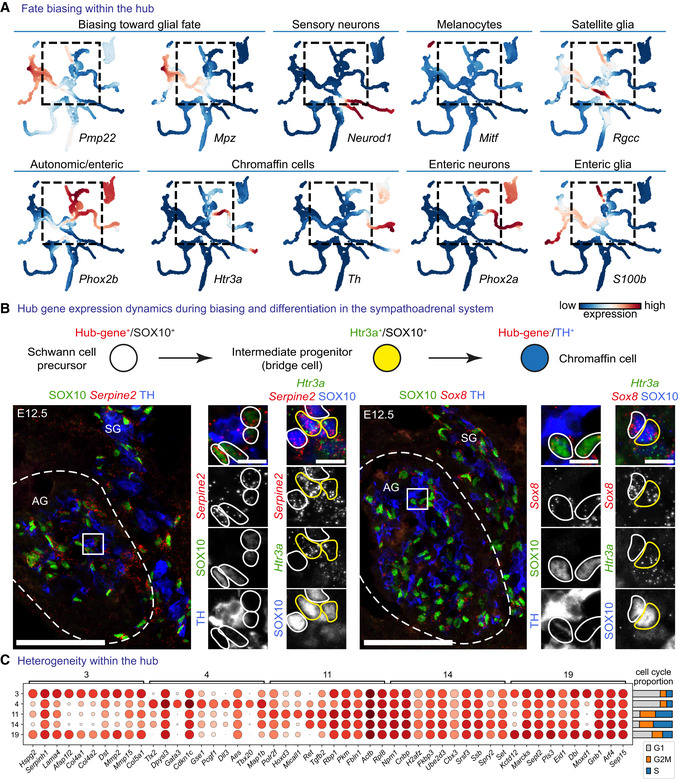
Cells in the “hub” state are subject to fate bias and a high degree of heterogeneity AMAGIC‐imputed markers of fate‐biasing within the “hub.”BRNAscope® *in situ* hybridization for “hub” genes *Serpine2* and *Sox8* in the developing adrenal gland combined with immunohistochemistry for SOX10 (progenitors—Schwann cell precursors (SCPs)), TH (differentiated chromaffin cells) or RNAscope® for committed SCPs towards chromaffin fate—*Htr3a*. Scale bar in overview pictures (left) is 100 and 10 μm in insets (right).CDot plot of top eight differentially expressed genes between Leiden clusters within the “hub.”

Despite the expression of core neural crest transcription factors (*Sox10*, *FoxD3*, *Tfap2a*, *Ets1*; Bronner & Simoes‐Costa, 2016) and signalling genes reported to be upregulated in the neural crest (*Erbb3, Ednrb, Ngfr*; Bernd, 1985; Nataf *et al*, 1996; Britsch *et al*, 1998), the “hub” cell population expressed high levels of specific genes in a unique combination, including *Sox8, Dlx1, Itga4* and *Serpine2*, rendering the “hub” different from the majority of delaminating and early migrating neural crest cells (Fig 2D and E). These “hub”‐specific genes were downregulated when the cells entered differentiation into terminal neural crest fates (Figs 2C–E, and 3A and B). This transient nature of the “hub” was validated experimentally in the developing adrenal gland using immunostaining against SOX10 (SCP marker) and TH (chromaffin cell marker) and RNAscope® to visualise the intermediate *Htr3a*‐expressing progenitors or “hub” genes *Serpine2* and *Sox8*. Namely, we observed the high expression of *Serpine2* and *Sox8* in SOX10^+^ SCPs and *Htr3a*‐expressing bridge cells but no expression upon differentiation towards TH^+^ chromaffin cells (Fig 3B).

On the other hand, the “hub” cells appeared heterogeneous by showing mixed expression of terminal fate‐related genes, which suggests a number of fate biasing programs operating within the “hub” (preceding particular commitment and further differentiation; Fig 3C). This is supported by the expression of fate‐specific genes *Pmp22* and *Mpz* (biasing towards SCs) or *Phox2a/b* and *S100b* (biasing towards autonomic neurons and glia), in the “hub” population (Fig 3A).

When it comes to the developmental origin of melanocytes, the corresponding branch in the gene‐based embedding (Fig 1B and E) does not attach to the rest of the tree likely because of the multiple convergent origins of pigment cells, which are derived from the migratory neural crest and from different populations of cranial and trunk SCPs. At the same time, the SCENIC‐based embedding (Fig 1G) connects the melanocyte branch to the rest of the tree, which suggests common mechanisms in melanocyte fate biasing, specification and differentiation. Similarly, the sympathetic neuronal branch stays disconnected in the gene‐based embedding, as sympathoblasts can originate directly from the ventral pathway of neural crest migration and via SCPs along the ventral nerves (Weston, 1970; Le Douarin & M‐AM, 1974; Kastriti *et al*, 2019). In the SCENIC‐based embedding (Fig 1G), the sympathetic neuron branch connects perfectly due to the common regulation of the differentiation process.

Finally, we identified and connected the satellite glial cells found in association with sensory neurons within dorsal root ganglia, and boundary cap stem cells (BCCs) associated with motor exit points from the neural tube. Using previously known markers (Coulpier *et al*, 2009; Mapps *et al*, 2022), we found the satellite glia and BCCs in the position where the neural crest populations transit into the “hub” (Fig 1B and Appendix Fig S4). The expression of “hub” markers in these cell populations suggests that they represent highly specialised subtypes of multipotent neuron‐ and nerve‐associated cells.

### Loss of “hub” gene *Sox8* results in SCPs defects and biased cell differentiation

**Figure 4:**
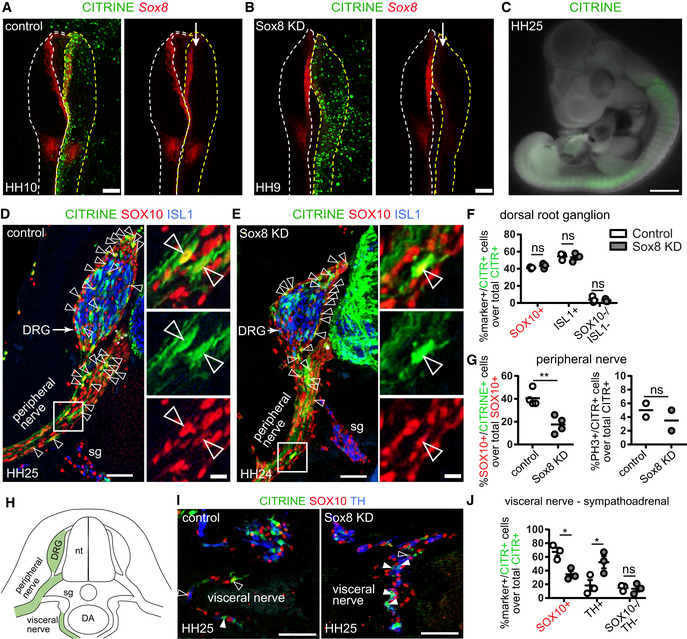
CRISP‐Cas9‐mediated knock down of *Sox8* in developing chicken late neural crest affects migration and differentiation of “hub” cells AElectroporation of the control CRISPR‐Cas9 plasmid (CITRINE^+^ cells) does not affect *Sox8* expression as seen by HCR against *Sox8*. The arrow points to the unilaterally electroporated side of the embryo. Scale bar = 200 μm.BValidation of *Sox8* knock down (KD) using the CRISPR‐Cas9 plasmid containing a *Sox8* guide RNA by HCR. The arrow points to the unilaterally electroporated side of the embryo. Scale bar = 200 μm.CCITRINE^+^ (electroporated) cells found migrating away from the neural tube after 3 days of culture following unilateral electroporation. Scale bar = 1 mm.D, EImmunofluorescence against CITRINE (electroporated cells), SOX10 (Schwann cell precursors and Schwann cells) and ISL1 (sensory and sympathetic neurons) on embryos electroporated with either control CRISPR plasmid (D) or a CRISPR plasmid containing a guide RNA against *Sox8* (E). CITRINE^+^ cells populate the developing dorsal root ganglia (DRG) and peripheral nerves. Examples of CITRINE^+^/SOX10^+^ cells shown by arrowheads. Asterisks show ventral boundary cap glia. Scale bar is 50 μm in overviews and 10 μm in insets.FQuantification of the fate distribution of CITRINE^+^ cells as a % between glial (SOX10^+^) cells, sensory neurons (ISL1^+^) or neither (SOX10^−^/ISL1^−^) in the DRG of control and Sox8KD chick embryos and Sox8KD chick embryos. Biological replicates – *N* = 3 embryos per condition (wild‐type versus *Sox8* KD). Data represented as mean ± SEM. Statistical significance determined using the Holm–Sidak method (α = 0.05; multiple *t*‐tests, unpaired). SOX10^+^ cells: *P* = 0.7901, ISL1^+^ cells: *P* = 0.9206, SOX10^−^/ISL1^−^ cells: *P* = 0.9206. For statistical significance: nonsignificant *P*‐value ≥ 0.05.GQuantification of (left) the % of SOX10^+^ cells in the peripheral nerves that are CITRINE^+^ in control and Sox8KD chick embryos and (right) the % of PH3^+^ CITRINE^+^ cells corresponding to proliferative cells. Biological replicates – *N* = 4 embryos per condition for SOX10^+^ distribution and *N* = 2 for PH3 quantification (wild‐type versus *Sox8* KD). Data represented as mean ± SEM. Statistical significance determined unpaired *t*‐test with two‐tailed *P*‐value. SOX10^+^ cells: *P* = 0.0044, PH3^+^ cells: *P* = 0.4929. For statistical significance: nonsignificant *P*‐value ≥ 0.05, **P‐value < 0.01.HSchematic representation of analysed anatomical locations.IImmunofluorescence against CITRINE (electroporated cells), SOX10 (Schwann cell precursors and Schwann cells) and TH (sympathetic neurons and chromaffin cells) of the sympathoadrenal domain on control and Sox8KD embryos. CITRINE^+^/SOX10^+^ cells shown by empty arrowheads while CITRINE^+^/TH^+^ cells are shown by filled arrowheads. Scale bar = 50 μm.JQuantification of the fate distribution of CITRINE^+^ cells as a % between glial (SOX10^+^) cells, chromaffin cells (TH^+^) or neither (SOX10^−^/TH^−^) in the proximity of the dorsal aorta and visceral nerve of wild‐type and Sox8 KD chick embryos. Biological replicates – *N* = 4 embryos per condition. Data represented as mean ± SEM. Statistical significance determined using the Holm–Sidak method (α = 0.05; multiple *t*‐tests, unpaired). SOX10^+^ cells: *P* = 0.0067, TH^+^ cells: *P* = 0.0067, SOX10^−^/TH^−^ cells: *P* = 0.8819. For statistical significance: nonsignificant *P*‐value ≥ 0.05, *P‐value < 0.05. Data information: In total, six embryos were analysed for the control electroporation and seven embryos were analysed for the *Sox8* knock down electroporation, and data depicted in graphs correspond to mean ± SEM per embryo (corresponding to biological replicates): 3–4 electroporated embryos were analysed per condition (control and SOX8 knock down) with 4–5 sections stained and analysed per embryo per region of interest (DRG, ventral nerve, sympathoadrenal domain). The only exception is the analysis of pH3 staining where two electroporated embryos were analysed per condition with five sections stained and analysed per embryo. DA, dorsal aorta; DRG, dorsal root ganglia; nt, neural tube; sg, sympathetic ganglion.

To test the function of the “hub”‐specific transcription factor *Sox8*, we electroporated the trunk neural tube of HH11 chick embryos with a single plasmid containing gRNA, Cas9 and a citrine reporter (Gandhi *et al*, 2021) to knockout *Sox8* in neural crest‐derived cells (Fig 4A and B). Embryos were then allowed to develop until HH24‐25 (Fig 4C), by which time the dorsal root ganglia (DRG) and visceral nerves had developed (Fig 4H). Quantification revealed a markedly reduced proportion of electroporated CITRINE^+^/SOX10^+^ cells along the ventral nerve after the loss of *Sox8* as compared to controls (Fig 4D, E and G). No significant differences were noted in the numbers of cells undergoing mitosis between experimental and control embryos, suggesting that *Sox8* might be involved in regulating migration or other processes rather than the proliferation of neural crest‐derived cells along the ventral nerve. This is consistent with a previous study suggesting a role for *Sox8* in neural crest migration (O'Donnell *et al*, 2006). Despite the phenotype in the ventral nerves, the loss of *Sox8* did not alter the number of glial (SOX10^+^) or neuronal (ISL1^+^) cells in the DRGs (Fig 4D–F), which supports that loss of SOX8 does not affect cell survival. By contrast, in the sympathoadrenal domain, the proportion of CITRINE^+^/SOX10^+^ nerve‐associated cells appeared reduced, whereas the proportion of CITRINE^+^/TH^+^ cells significantly increased (Fig 4I and J). This result suggests elevated rates of conversion of sympathoadrenal SCPs into adrenergic fates. Taken together, high levels of *Sox8* might specifically stabilise the SCP/“hub” phenotype and assist the migration of neural crest‐derived SOX10^+^ cells along the peripheral nerves.

### Coordinated metaregulons maintain the neural crest‐like state in “hub” cells

The “hub” population represents a transcriptional state common to some late NCCs and SCPs before commitment to other fates (Fig 1B). This favours the interpretation of “hub” cells as a nerve‐associated, late‐NCCs. In cases, when migratory NCCs do not immediately give rise to sensory and autonomic neurons, ectomesenchyme or melanocytes (Soldatov *et al*, 2019), NCCs reach the nerve‐associated “hub” state before differentiation towards “nerve‐derived” autonomic neurons, chromaffin cells and melanocytes. BCCs and satellite glial cells can also give rise to sensory neurons of the second wave and, consistently, they occupy the intermediate position on the embedding expressing “hub”‐enriched markers, such as high levels of *Serpine2* and *Ednrb*, lower levels of *Itga4* and *Sox8*, with the absence of *Dlx1/2* as opposed to the rest of the “hub” (Fig 2D).

**Figure 5:**
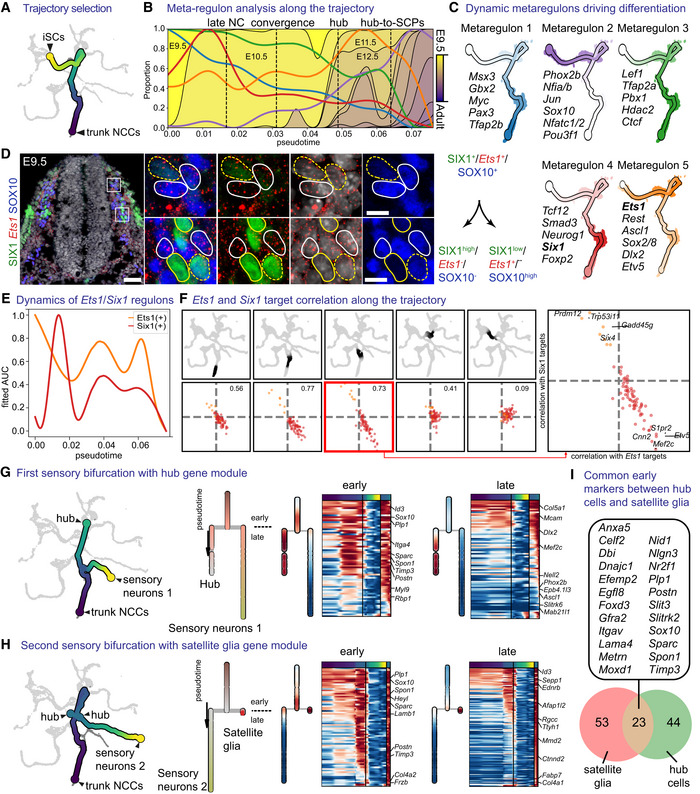
Dynamic changes of metaregulons shape biologically distinct stages of neural crest and Schwann cell precursor development and differentiation ASubset of the tree containing the trajectory of trunk neural crest to immature Schwann cells.BProgression of the metaregulons over the trajectory selected in (A), combined with developmental time. A metaregulon is the mean expression of all regulons composing a cluster over pseudotime.CTrajectory coloured by metaregulons, with a selection of regulons composing them.DValidation of SIX1 and *Ets1* as markers for biasing towards sensory and glia fates, respectively. Scale bar is 50 μm in the overview picture and 10 μm in the insets. Stainings were repeated on two separate occasions on two embryos from the same litter. Cells surrounded by solid white lines correspond to SIX1^-^/Ets1^+^/SOX10^+^ SCPs or glial fate-biased progenitors, while cells surrounded by dashed yellow lines SIX1^+^/Ets1^-^^or low^/SOX10^- or low^ sensory fate-biased progenitors.ENormalised regulon activity scores of *Ets1* and *Six1* over the trajectory.FInter‐/intra‐correlation analysis of the target genes of *Ets1* and *Six1* regulons on nonintersecting windows of cells over the trajectory.G, HBifurcation analysis over two successive sensory branches, highlighting the early and late genes biasing towards “hub” cells (G) and satellite glia (H).IVenn diagram of early genes expressed in the bifurcation between “hub” and satellite glia. Gene list shows the common genes between the two. Data information: iSCs, immature Schwann cells; NCCs, neural crest cells.

To investigate the in‐depth regulatory principles in early migratory NCCs and “hub”/SCPs on the portion of the SC trajectory, we employed pseudotime analysis to reconstruct the finest events of the correlated transcriptional activation. To achieve this, we fitted a principal curve spanning from the cluster of the trunk neural crest proximal to the “hub” state towards the tip of the differentiation of SCs or the emergence point of immature Schwann cells (iSCs; Fig 5A). We then tested and fitted significant AUC scores to the inferred differentiation trajectory and superimposed the biological developmental time transferred from the individual cell transcriptomes (Fig 5B). This allowed us to delineate changes in the transcriptional activation along developmental time. We then relied on the concept of regulons, which correspond to a cluster of coherently co‐activated genes driven by a specific transcription factor. Observations of such coordinated transcriptional activation highlight the key regulatory events driving developmental transitions and help to predict possible causal molecular interactions related to multipotency and cell fate choice.

**Figure EV2:**
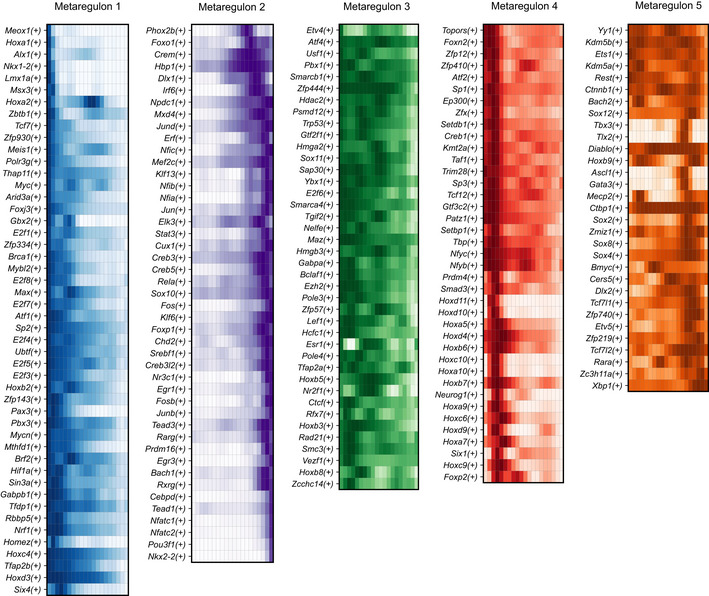
Metaregulon composition Heatmaps of regulon activity (AUC scores) of individual regulons making up metaregulons 1–5, over the neural crest to immature Schwann cell trajectory, summarised into 20 pseudotime bins.

Clustering of the scaled fitted transcriptional activity scores resulted in identifying five groups of coordinated regulons, which we termed metaregulons (Figs 5C and EV2). Of these, two metaregulons were characterised by high activity during the early migratory NC‐stage (E9.5 and to a lesser degree E10.5) that subsequently decreased as the trajectory proceeds towards the “hub”/SCP state and further to SCs (metaregulons 1 and 3). Metaregulon 1 and to a lesser degree metaregulon 3 were composed of classical neural crest specification genes (*Msx3*, *Gbx2*, *Pax3*, *Tfap2a/b*; Basch *et al*, 2006; Khudyakov & Bronner‐Fraser, 2009) or regulators of WNT1 signalling (*Myc*, *Lef1*; Hao *et al*, 2019). Metaregulon 3 included genes involved in chromatin remodelling and accessibility, such as *Ctcf* and *Hdac2*, with key roles in Schwann cell specification and myelination (Jacob *et al*, 2011; Lee *et al*, 2022). The dynamics of metaregulon 3 also show multiple peaks reflecting critical periods during which chromatin remodelling and biasing to promyelinating fate might occur. Comparison of the activity of regulons governing early migratory crest and multipotent “hub”/SCPs leaning towards SC fate uncovered a remarkable similarity of these progenitor types (Figs 5B and EV2). For instance, a significant part of this similarity could be attributed to the joint activity of the *Ets1*(+) (metaregulon 5), *Pax3*(+) and *Tfap2b* (metaregulon 1) and *Tfap2a* (metaregulon 3) regulons (Fig 5C). Concomitantly, we detected smooth and incrementally increasing differences in overall gene expression starting from the NC towards SCPs at the transition between late E10.5 to E11.5 and E12.5, including the onset of activity of *Jun*, *Sox10*, *Nfatc1/2* and *Pou3f1*, all genes with described roles in myelination (metaregulon 2; Fig 5C; Mirsky *et al*, 2008). Notably, *Jun* represses myelination (Parkinson *et al*, 2008), whereas *Pou3f1* and *Sox10* synergistically promote myelination (Britsch *et al*, 2001; Ghislain & Charnay, 2006; Schreiner *et al*, 2007). The simultaneous presence of active repressors and facilitators of myelination within the same group of coordinated regulons reflects opposing cell biasing tendencies, which eventually result in separation of SC fates towards myelinating and non‐myelinating, as seen by the decrease in *Jun* activity and increase in *Pou3f1* activity (Fig EV2). Interestingly, *Nfatc1/2* factors have not been studied so far in SCs, although they are indispensable for myelination by oligodendrocytes, where they synergize with *Sox10* (Weider *et al*, 2018).

The active regulons belonging to metaregulon 4 corresponded to neurogenic sensory bias activated in NC and early “hub” cells at E9.5‐E10.5 and included *Six1* and *Neurog1* regulons (Fig EV2; Sommer *et al*, 1996; Zou *et al*, 2004). Surprisingly, metaregulon 5 showed two peaks of activity, interrupted by the peak of the pro‐neurogenic metaregulon 4, which resolved upon neuronal differentiation as the two lineages split, with *Ets1* expression retained in SOX10^+^ SCPs but downregulated in SIX1^+^ sensory progenitors and neurons, as confirmed by experimental validations using immunofluorescence and RNAscope® (Fig 5D–F). Consistent with this, overexpression of neurogenic factor *Six1* suppresses the expression of *Ets1 in vitro* (Hosseinipour, 2017). The temporary downregulation of *Ets1* metaregulon in the middle of the differentiation trajectory is due to mosaic compositional effects that result from a significant proportion of cells upregulating the activity of the neurogenic *Six1* regulon and downregulating the activity of *Ets1* regulon; other cells in the same location on the trajectory maintained high levels of *Ets1* regulon without switching on *Six1* regulon (and were intermixed with the first population; Fig 5F). Consistently, the sensory neurogenic subtrajectory started from the *Ets1* ^+^ NCCs and generated the first branch of *Six1*‐activating sensory neurons, and also gave rise to the “hub” cells (Fig 5G) and sensory satellite glia (Fig 5H). Sensory satellite glia converged with the “hub” and generated the second sensory neurogenesis branch via activation of *Six1* regulon (Zou *et al*, 2004). The comparison between satellite glia and neighbouring “hub” cells uncovered a set of commonly expressed genes (including classical neural crest “core” of transcription factors), even though the two cellular populations were additionally characterised by separate gene expression programs (Fig 5I).

Metaregulon 5 included the *Ets1* and *Sox8* regulons (part of the essential NC gene expression programs; O'Donnell *et al*, 2006; Bronner & Simoes‐Costa, 2016) and the regulon of the transcriptional repressor *Rest* (Chong *et al*, 1995), which initially showed high activity during the early NC phase. Additionally, *Ascl1*, *Sox2* and *Etv5* regulons, all expressed in embryonic multipotent Schwann cell precursors, belong to metaregulon 5. Accordingly, members of metaregulon 5 were reactivated in the rest of the “hub” and SCPs before commitment to terminal fates in cells from E11.5 to E14.5. TFs *Ets1* and *Sox8* are indispensable for the NC development (Tahtakran & Selleck, 2003; O'Donnell *et al*, 2006; Nie & Bronner, 2015), and their activity may convey multipotency and control the spectrum of available fates in “hub”/SCP populations, which is consistent with our functional experiments with targeting *Sox8* (Fig 4). Furthermore, *Ascl1* correlates with fate‐biasing of SCPs towards autonomic neurons and chromaffin cells (Memic *et al*, 2016; Furlan *et al*, 2017); *Sox2* inhibits melanocyte fate (Adameyko *et al*, 2012) and Schwann cell differentiation/myelination (Hagedorn *et al*, 2000; Le *et al*, 2005; Balakrishnan *et al*, 2020), whereas *Etv5* has been shown to have a dynamic expression in the SCP lineage. Thus, metaregulon 5 reflects transcription factor activity facilitating multipotency at discrete stages of the neural crest and SCP lineage fate restriction.

Next, we questioned the differences in regulon activity and transition into the “hub” in the cranial and trunk neural crest cells, which are both multipotent populations distinguished by the ability of the cranial neural crest to give rise to ectomesenchyme, a property lacking in the trunk neural crest. Additionally, a significant portion of the cranial neural crest cells delaminates from the anterior Hox‐negative neuroepithelium. During a comparative regulon analysis between the trajectories of cranial versus trunk neural crest progressing towards the iSCs stage (Appendix Fig S8), we observed a significant overlap between regulons detected in the cranial and trunk trajectory (175 regulons in common, with 28 trunk‐specific and 14 cranial‐specific). While cranial‐specific regulons included *Tfap2c*, *Msx1/2*, *Lbx2*, trunk‐specific regulons included, as expected, *Hox* genes and *Six1/4*, *Neurog1*, which correspond to sensory neurogenesis prominent in the trunk region. However, both cranial and trunk neural crest cells converge later towards the “hub” state, as shown by RNAvelocity and Cytotrace (Fig 1C and H) and contribute equally to the generation of multipotent Schwann cell precursors found on cranial and trunk peripheral nerves. This is supported by the experimental evidence showing that SCPs covering cranial nerves are multipotent and generate the majority of cranial melanocytes and chromaffin‐like cells of the carotid oxygen‐sensing organ (Adameyko *et al*, 2012; Hockman *et al*, 2018).

Finally, our analysis suggested that although transcriptional changes along the differentiation trajectory and developmental time are smooth, they can be broken down into overlapping patterns of multiple transcriptional activation events. The analysis of regulons provides a discrete picture compared with total gene expression, which allows for defining, isolating and discriminating critical transitions and developmental states of the NC lineage. For instance, the peaks of activity of metaregulon 5 overlap with the developmental stages of experimentally validated SCP and NC multipotency (at E9.5–E10.5 and E11.5–E13.5) and, thus, can be used to define the “hub”/early SCP state versus committed glial progenitor state characterised by the activity of metaregulon 2. Similarly, the activity of NC‐specific metaregulon 1 and cell reprogramming‐related metaregulon 3 helps to set a border between the early migratory NC and later phases connected to the nerve association and “hub” state with what we anatomically term as SCP state (SOX10^+^ cells associated with nerves). Therefore, the “hub”/SCPs might be defined as a cell state with reactivation of metaregulon 5 coinciding with gradually reducing the activity of metaregulons 1 and 3 (linked to neural crest properties and cell reprogramming) before the activation of metaregulon 2 (linked to the terminal differentiation of Schwann cells; Fig 5B and C). Thus, the “hub” state is distinct in terms of regulation from the majority of the neural crest cells where metaregulons 1 and 3 are dominating.

### The “hub”/SCP state is a mixture of differentially biased progenitors that cross‐consolidate multiple fates

The “hub”/SCP state appears to be a point of transcriptional regulation, where fate decisions take place before commitment to definitive cell types. The depth and the quality of the obtained data allowed us to probe the molecular mechanisms of fate selection at this state.

**Figure EV3:**
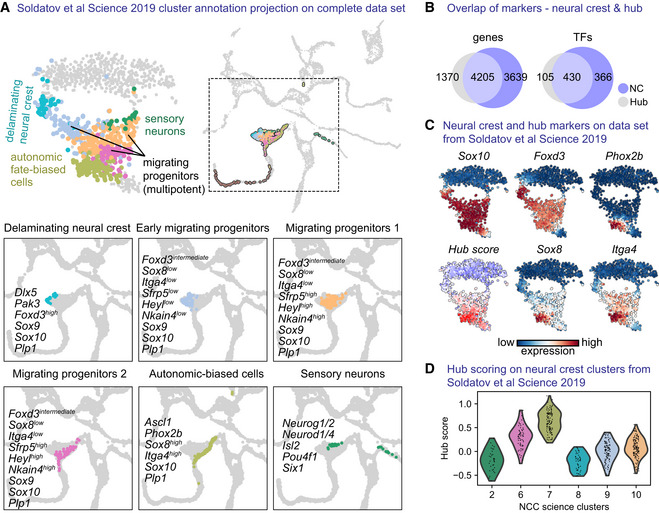
Mapping and comparison of the “hub” state on previously published single‐cell data set of the neural crest AtSNE embedding and annotated clusters from Soldatov *et al* (2019), overlayed onto the UMAP embedding containing our own data set.BVenn diagram showing genes and transcription factors (TFs) positively regulated when comparing, respectively, neural crest cells and “hub” cells to the rest of the cells of the data set (Wilcoxon rank‐sum test).C“hub” scoring on previous data set using gene scoring from the top 25 differentially expressed genes specific to the “hub.”DViolin plot of “hub” score over the published annotated clusters.

Recently, we reported that NCCs differentiate towards downstream fates through a system of consecutive bifurcations mediated by mutually repressive transcriptional programs leading to noncompatible fates (Soldatov *et al*, 2019). With the help of our “hub”‐specific gene expression signature (*Sox8, Itga4* etc.), we mapped the “hub”‐state onto the neural crest Soldatov *et al* data set to detect the emergence of the early “hub” program (Fig EV3). For this, we relied on the described states of neural crest development including delaminating, multipotent migrating progenitors, autonomic‐biased cells and cells differentiating towards neurons (Fig EV3A). Briefly, delaminating neural crest cells are defined by *Dlx5*, *Pak3*, *Sox9*, *Sox10* genes and migrating groups are characterised by *Sfrp5*, *Heyl* and onset of *Nkain4* expression, while sensory neurons expressed *Neurog1/2*, *Pou4f1*, *Isl1*, *Six1* and *Neurod1/4*. Joined analysis of the genes expressed by the neural crest and “hub” cells uncovered different degrees of similarity in gene expression (Fig EV3B and Dataset EV1). Some cells previously considered as multipotent migrating progenitors in Soldatov *et al* data set appeared more similar to the “hub” state as compared to others with the highest “hub” signature identified in the NCCs biased to the autonomic fate before neuro‐glial separation (Fig EV3A, C and D). This observation reinforces the idea that the emerging “hub” state is seen at late neural crest stages, prior to nerve association but is reinforced and upregulated following the attachment of cells to the peripheral nerves. Taking into account these similarities, we analysed the fate choice mechanisms mediating transitions from “hub”/SCPs towards immature Schwann cells (iSCs), enteric populations and sympathoadrenal progenitors in order to compare them with those reported for the NC by Soldatov *et al*.

**Figure 6:**
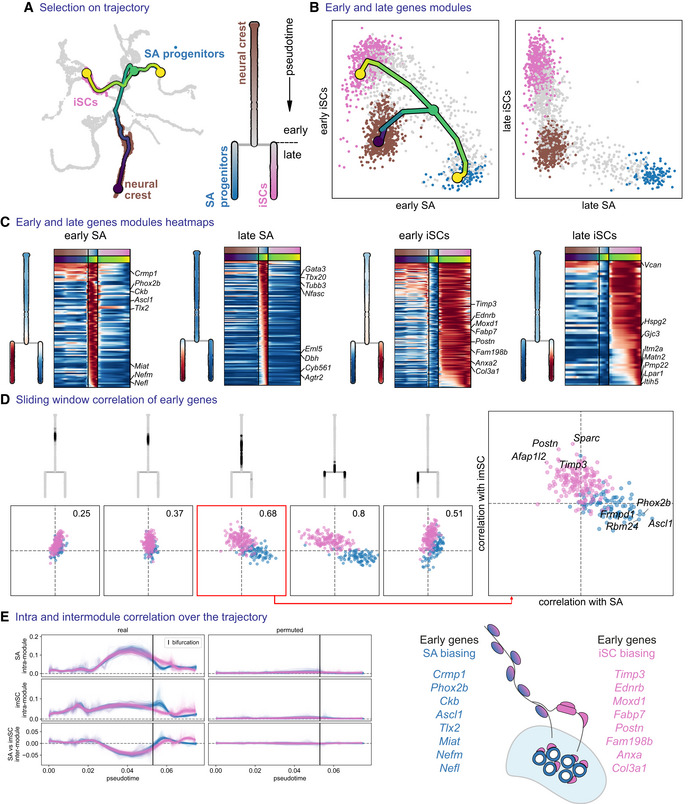
Biasing of bipotent progenitors through the “hub” state towards sympathoadrenal progenitors and immature Schwann cells ASubset tree selection of the trajectory from trunk NCCs to immature Schwann cells versus sympathoadrenal progenitors. An abstract dendrogram is displayed on the right.BMean expression of early and late gene modules for each branch and overlayed tree on the early gene modules.CHeatmap of early and late genes for each branch, with mean expression shown on the dendrogram representations.DInter‐/intra‐correlation analysis of early gene modules of both branches, on nonintersecting windows of cells over the trajectory.EInter‐ and intra‐module correlation performed on a sliding window of cells (over 100 probabilistic mappings of the trajectory) along the pseudotime axis. iSCs, immature Schwann cells; SA progenitors, sympathoadrenal progenitors.

First, we focused on a developmental paradigm specific to the sympathoadrenal domain. During development, spinal preganglionic sympathetic axons innervate the developing adrenal gland, being covered with SCPs (Furlan *et al*, 2017; Lumb *et al*, 2018). In mice, between E11.5 and E13.5, SCPs detach from the preganglionic axons and differentiate towards chromaffin cells that will populate the medulla of the adrenal gland. However, not all SCPs detach from the nerves; some remain associated with axons and generate iSCs (Jessen & Mirsky, 2019). We examined progenitors giving rise to these two cell types starting from NC cells (Fig 6A). Similarly to the mechanisms reported by Soldatov *et al*, this analysis revealed the initial co‐activation of lineage‐specific gene modules composed of “early” genes prior to the bifurcation, then subsequent repression of these modules (detected as the negatively correlated expression of genes belonging to the opposing modules) and, finally, activation of “late” genes around the bifurcation point (Fig 6B–E). In agreement with the previous studies, the onset of *Ascl1* and *Phox2b* (Huber *et al*, 2002, 2005) genes biased SPCs towards sympathoadrenal fate, while important drivers of iSC fate were *Ednrb, Timp3, Moxd1, Postn, Fabp7* and others (Fig 6C and E).

Next, we focused on SCPs that share gene signatures leading towards chromaffin cells and enteric glia (Appendix Fig S10A–C). Even though the two lineages share a common core of genes (i.e. *Ascl1, Phox2b, Hand2*), we could not see a real bifurcation and concomitant fate choice taking place, as we did not identify co‐activation of the two competing prebiasing modules and found that these modules were expressed in different cells (Appendix Fig S10D and E). This is not surprising given that most of the enteric glia come from vagal neural crest cells, whereas sympathoadrenal progenitors arise from the trunk being spatially segregated from the vagal. This result supports the robustness of our approach, which can identify real fate choices from the artificial situations resulting from the transcriptional similarity.

Lastly, we focused on the development of enteric glia (found in the intrinsic enteric ganglia) versus the iSCs found in the extrinsic innervation that reaches the gut (Appendix Fig S11A–C). During development, the enteric nervous system (enteric neurons and glia) is derived from vagal NCCs that migrate to the posterior trunk and populate the developing intestine. However, some SCPs delivered by the external innervation can also differentiate between enteric glia and neurons (Uesaka *et al*, 2015; El‐Nachef & Bronner, 2020). Our bifurcation analysis confirmed the initial co‐expression and then mutual repulsion of “early” enteric genes *Ret, Ascl1, Phox2b* and *Hand2*, and the opposing *Igfbp5, Prss12, Moxd1, Egfl8* program biasing the cells towards remaining on the extrinsic nerves and becoming iSCs (Appendix Fig S11C–E).

Overall, we found that the “hub”/SCP state is comprised of a mixture of differentially biased progenitors expressing early opposing programs, thus highlighting the local heterogeneity of cells in this developmental phase. The basic mechanisms of cell fate choice based on the co‐activation and subsequent mutual repulsion of transcriptional programs leading to definitive fates also appeared consistent with the earlier findings reported for the NC as described by (Soldatov *et al*, 2019).

### Immature Schwann cells are biased towards myelinating, non‐myelinating Schwann cells and other fates

Previous studies established that SCPs along the nerves transit to iSCs before differentiating towards two alternative fates: myelinating SCs or non‐myelinating SCs (also known as Remak cells; Jessen & Mirsky, 2005). Additionally, during perinatal stages in rodents, a *Dhh*‐expressing subpopulation of iSCs has been described to generate endoneurial fibroblasts (Parmantier *et al*, 1999; Joseph *et al*, 2004; Sharghi‐Namini *et al*, 2006).

**Figure 7:**
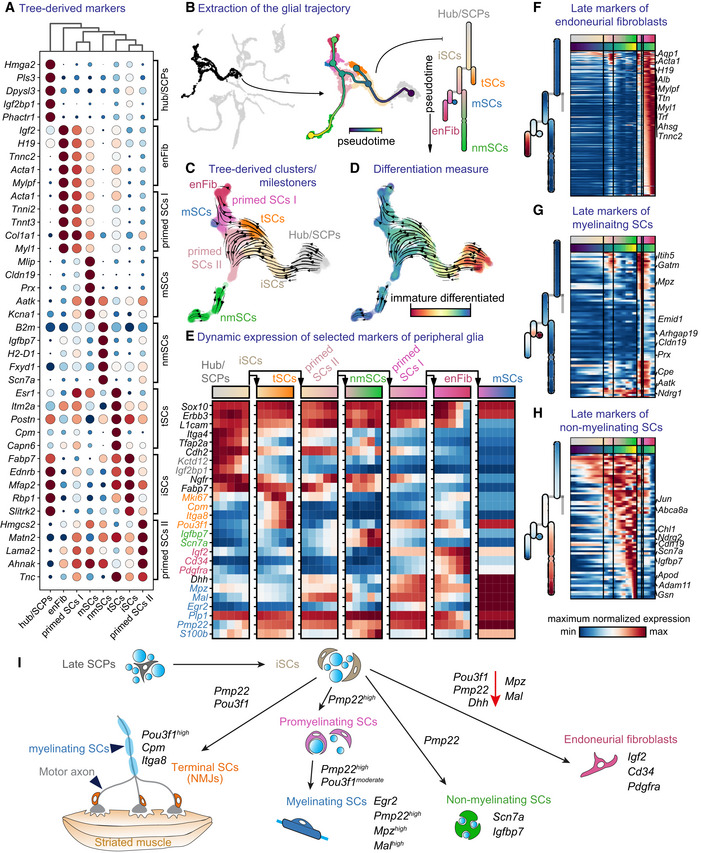
Heterogeneity of Schwann cells and fate biasing in maturing peripheral nerves ADot plot of the top five differentially expressed genes from the milestones of the glial trajectory.BLeft: selected cells (Foster *et al*, 2020) of the glial trajectory, middle: constructed tree overlayed on the UMAP, right: abstract dendrogram.CRNA velocity on tree‐derived clusters (milestones).DRNA velocity with CytoTRACE overlayed on the UMAP.EDynamics of expression of known markers over the trajectory summarised over five bins per segment of the tree.F–HHeatmap of late markers of the three endpoints of differentiation from immature Schwann cells: endoneurial fibroblasts (F) and myelinating (G) or nonmyelinating Schwann cells (H).ISchematic representation of transitions from late “hub” cells biased to immature Schwann cells to all end fates along with mixed motor and sensory nerves. Data information: enFibr, endoneurial fibroblasts; iSCs, immature Schwann cells; mSCs, myelinating Schwann cells; NMJs, neuromuscular junctions; nmSCs, non‐myelinating Schwann cells; SCPs, Schwann cell precursors; tSCs, terminal Schwann cells.

Even though genes driving myelination in SCs are well known (*Sox10*, *Egr2, Pou3f1*; Topilko *et al*, 1994; Bermingham Jr. *et al*, 1996; Jaegle *et al*, 1996, 2003; Le *et al*, 2005; Schreiner *et al*, 2007; Finzsch *et al*, 2010), the exact mechanisms underlying fate choice between the three lineages (myelinating, Remak or fibroblastic) are not fully elucidated. Studies point to the crucial role of NRG1‐type III levels supplied by the nerve and *Sox10* expression, while SOX2 has been shown to suppress myelination (Britsch *et al*, 2001; Taveggia *et al*, 2005; Schreiner *et al*, 2007; Finzsch *et al*, 2010; Roberts *et al*, 2017). To address this, we focused on cells sampled from E11.5 onwards (a time point where all SOX10^+^ cells are considered to be SCPs and no longer NCCs due to complete association with nerves or peripheral neurons), in order to understand fate separations towards myelinating, non‐myelinating SCs, neuromuscular junction‐associated terminal glial cells and endoneurial fibroblasts (Fig 7B).

**Figure 8:**
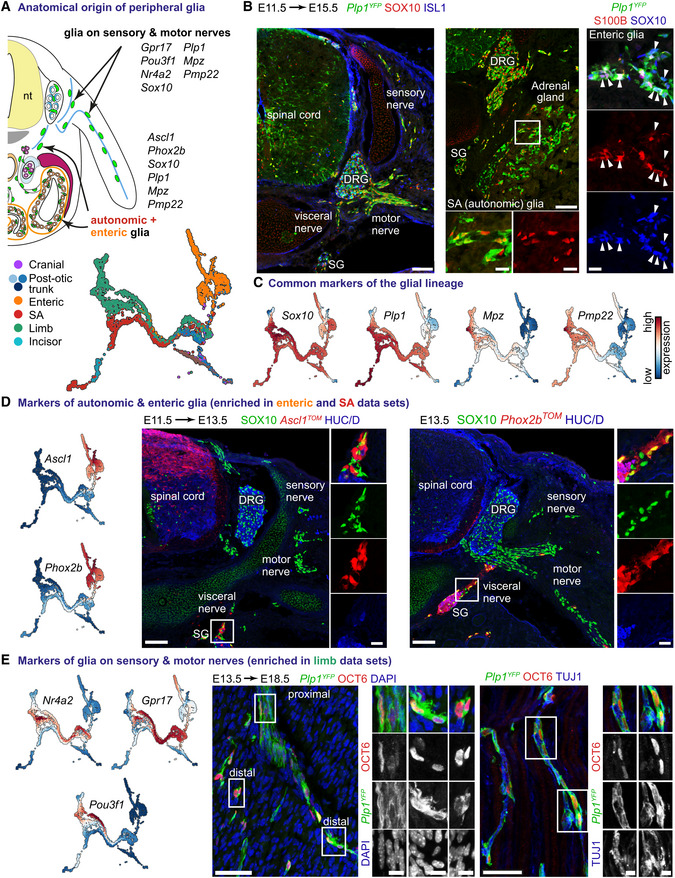
Positional code of heterogeneity of Schwann cell precursors and Schwann cells ATranscriptional code of peripheral glial cells of various anatomical locations.BImmunofluorescent staining against SOX10 (a marker of all peripheral glia) and ISL1 (neuronal marker; left panel) or S100B (a marker of a subset of peripheral glia) and SOX10 (right panel) on E11.5 to E15.5 *Plp1* ^*CreERT2*^ *;R26* ^*YFP*^ trunk in various anatomical locations. Scale bar in the overview pictures is 100 and 10 μm in insets. Arrowheads point to SOX10^+^/S100B^+^ enteric glia.CUMAPs of classical genes shared in peripheral glia.DLeft: UMAPs of *Ascl1* and *Phox2b* expression. Right: Immunofluorescent staining against SOX10, *Ascl1* ^*TOM*^ or *Phox2b* ^*TOM*^ (lineage tracing of autonomic and enteric glia) and HUC/D (neuronal marker) on E13.5 trunk in various anatomical locations. Scale bar in overview pictures is 100 and 10 μm in insets.ELeft: UMAPs of genes enriched in peripheral glia sampled from the developing limbs. Middle: Immunofluorescent staining against OCT6 (encoded by Pou3f1) and TUJ1 (axonal marker; left panel) on hindlimbs of E13.5 to E18.5 *Plp1* ^*CreERT2*^ *;R26* ^*YFP*^ trunk showing the various levels of OCT6 immunoreactivity and variable morphology of *Plp1* ^*YFP*+^/OCT6^+^ cells along the motor nerves. Scale bar in overview pictures is 50 and 10 μm in insets. Stainings were repeated on two separate occasions on multiple embryos from the same litter. Data information: DRG, dorsal root ganglion; SA, sympathoadrenal; SG, sympathetic ganglion.

**Figure EV4:**
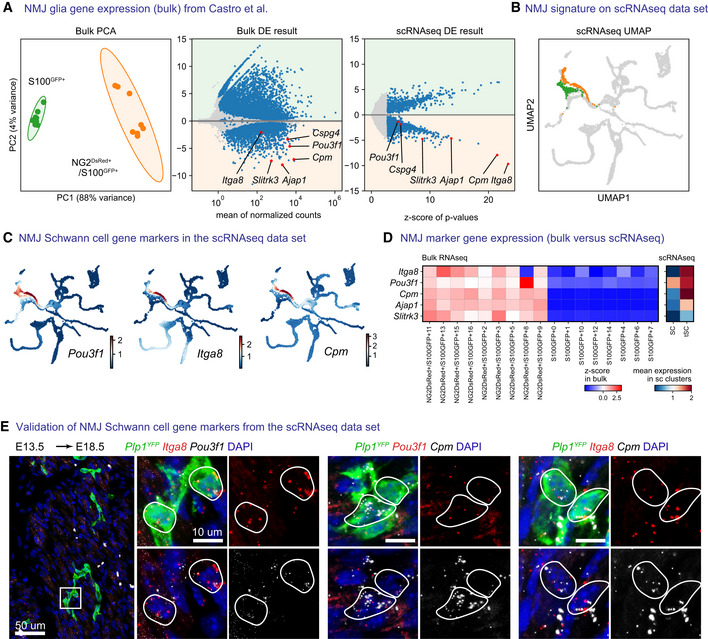
Late embryonic and early postnatal terminal glia representation in the data set ARe‐analysis of bulk RNA sequencing data of S100^GFP+^ cells and NG2^dsRED+^/S100^GFP+^ cells isolated from adult neuromuscular junctions from Castro *et al* (2020) with PCA plot (left) or DESeq2 differential gene expression analysis (center) and comparison of differential expression results (right) from Leiden clusters of terminal Schwann cells and fate‐biased Schwann cells in our own data set.BLeiden clusters defining terminal Schwann cells (orange) and fate‐biased Schwann cells (Green *et al*, 2017).CMAGIC‐imputed expression of *Pou3f1*, *Itga8* and *Cpm* on UMAP embeddings.DComparison of five terminal Schwann cells markers, as z‐scores on bulk data per sample (left) and as mean expression in the Leiden clusters shown in (A and B) in our own data set (right).ERNAscope *in situ* hybridization validation of *Pou3f1*, *Itga8* and *Cpm* as markers of terminal Schwann cells at E18.5 combined with immunofluorescence against *Plp1* ^*YFP*^ on hindlimbs of an embryo injected with tamoxifen at E13.5. Stainings were repeated on two separate occasions on multiple embryos from the same litter.

Firstly, RNA velocity and CytoTRACE analysis both agreed on the directionality of the transition from *Sox10* ^+^/*Erbb3* ^+^ */Ngfr* ^+^ */L1cam* ^+^ */Itga4* ^*high*^ */Cdh2* ^+^ */Tfap2a* ^+^ */Fabp7* ^+^ */Plp1* ^+^ SCPs/“hub” cells towards iSCs, identified by the decrease in *Itga4* levels and progressive *S100b* upregulation (Jessen & Mirsky, 2005; Fig 7A and C–E). Differentially expressed markers suggested an early splitting cell type derived from iSCs, which corresponded to terminal SCs of neuromuscular junction SCs (tSCs; Figs 7C and E, and EV4), based on previously reported markers such as *Cspg4*, *Itga8, Slitrk3, Cpm, Pou3f1* (Castro *et al*, 2020). Biological validations showed the early origin of *Itga8* ^+^ */Cpm* ^+^ */Pou3f1* ^+^ terminal SCs as seen by RNAscope® or immunofluorescence against OCT6 (encoded by *Pou3f1*) on E18.5 *Plp1* ^*YFP*^‐traced embryos when recombination was induced at E13.5 (Figs 8E and EV4E).

Next, the analysis of the differentiation downstream of iSCs and the inference of a tree on the multiscale diffusion space generated from SCENIC AUC scores revealed a bifurcation between two primed states of SCs (primed SCs I and II). Primed SCs I show progressive downregulation of *Ngfr*/*Cdh2* with *Pou3f1*/*Dhh* upregulation differentiating towards *Igf2* ^+^ */Cd34* ^+^ */Pdgfra* endoneurial fibroblasts (Fig 7C–F; Richard *et al*, 2014; Carr *et al*, 2019). Primed SCs II expressed lower levels of *Dhh*, retained *Cdh2* expression, whereas *Fabp7* downregulation coincided with *Ngfr* increase and eventually gave rise to either *Mpz* ^+^ */Prx* ^+^ */Pmp22* ^+^ myelinating or *Scn7a* ^+^ */Jun* ^+^ non‐myelinating SCs (Fig 7C–E, G and H).

Interestingly, biased early SCs (downstream of iSCs), display an overlap of expression of multiple lineage‐specific markers, such as *Mpz*, *Mal*, *Dhh*, *Pmp22* and *Pou3f1* albeit at variable levels (Fig 7E). This suggests that these SCs are experiencing conflicting gene programs for selecting different fates. These observations are compatible with the classical model of myelination onset, as cells of these three paths are characterised by varying expression of *Pou3f1* before the onset of *Egr2* expression, which defines the myelinating terminal state. Surprisingly, the path with the highest experimentally validated expression of *Pou3f1* led to the terminal neuromuscular SCs, side‐tracking early SCs away from the myelinating fate via an unknown mechanism (Figs 6E and EV4). This is consistent with the reasoning that cells along the adjacent fibroblast‐biased path have less chance of acquiring myelinating or non‐myelinating SC fate, which opens up new interesting domains of research in regards to unveiling the factors determining this fate restriction.

Overall, our analysis revealed a complex system of transcriptional control and gene expression characterising differentiation programs that likely play a role in biasing or priming SC populations. For instance, even though *Pou3f1* (coding for OCT6) has been previously reported to play a crucial role in myelination, it additionally emerged as a candidate biasing factor towards terminal neuromuscular junction SCs, suggesting a novel dual role in nerve development.

### Schwann cell lineage heterogeneity reflects positional codes

Given the fate bias‐related heterogeneity of a “hub”/SCP population identified in our single‐cell analysis, we hypothesised that one of the critical aspects driving heterogeneity might be the spatial distribution of SCPs in the embryo (Fig 8A–C). If this hypothesis is correct, the differences in downstream fate acquisition might correlate with differences in location‐specific transcriptional programs in “hub”/SCPs.

For example, our bifurcation analysis revealed gene expression programs biasing “hub” cells/SCPs towards sympathoadrenal progenitors (*Ascl1* and *Phox2b* expression) or future mature glia (*Ednrb, Moxd1, Postn* expression; Fig 6). Additionally, we observed that peripheral glial cells derived from the sympathoadrenal system, gut and developing limbs intermingle in the computed SCP branch towards non‐myelinating SCs (Remak cells) and primed SCs II (Fig 8A). However, glia of sympathoadrenal/enteric locations separated from the branches towards myelinating SC, endoneurial fibroblast and primed SCs I (Fig 8A). This suggests that glia along the visceral nerves (preganglionic axons sprouting from visceral motor neurons and postganglionic axons of the autonomic nervous system) might carry a different positional code as compared to the glia found on sensory and motor nerves that reach the skin or axial muscles. Such positional code could restrict nerve‐associated cells to autonomic neurogenesis and non‐myelinating SCs. In line with this hypothesis, lineage tracing in E13.5 *Ascl1* ^*CreERT2*^ *;R26* ^*TOMATO*^ embryos (traced at E11.5) and E13.5 *Phox2b* ^*Cre*^ *;R26* ^*TOMATO*^ embryos, revealed two lineages restricted to the sympathetic chain, adrenal gland and paraganglia, and, correspondingly, enteric nervous system (Fig 8D and Appendix Fig S12).

Further examination of cells from E11.5 and later stages revealed correlations of gene expression with the anatomical origin of cells (Appendix Fig S13). For example, *Ascl1* and *Phox2b* expression in glia was limited exclusively to the autonomic and enteric nervous system at E12.5. On the other hand, E12.5 glia sampled from the developing limb exclusively expressed high levels of *Nr4a2* (validated using RNAscope®) and increasing levels of *Gjc3* (coding for CX29), which was also observed at E14.5, E16.5, E18.5 and perinatal stages (Fig 8E and Appendix Fig S12). This regionalized expression of *Nr4a2* coincided with that of *Gpr17*, *Pou3f1* and other Schwann cell markers (*Cpm*, *Itga8*; Fig 8E and Appendix Fig S13). Accordingly, we hypothesised that NURR1 (coded by Nr4a2) might be a novel gene in SC fate consolidation or myelination. However, analysis of *Nr4a2* ^−/−^ newborn mice through immunofluorescence showed no apparent defect in SC distribution on the axons innervating the limbs when compared to *Nr4a2* ^+/+^ littermates (Appendix Fig S12D).

Next, OCT6^*high*^ cells (encoded by *Pou3f1*) were predominantly found in developing and postnatal limbs, with an increasing gradient of OCT6 levels from the proximal‐to‐distal part of the peripheral nerves (Fig 8E). These cells were characterised by the signature of terminal neuromuscular SCs as seen by the expression of *Cpm*, *Itga8*, *Slitrk3*, *Cspg4* and other markers previously shown enriched in this SC subpopulation (Fig EV4A, B and D, and Appendix Fig S13). As compared to this subgroup of limb‐dwelling glial cells, sympathoadrenal or enteric glia had much lower *Gjc3* expression during development, which caught up with expression levels of *Gjc3* found in embryonic limbs only postnatally (Appendix Fig S13).

Many genes associated with the early SC lineage are expressed transiently (for instance, *Nrp1*, *Nrp2*, *Ednra/b* and *Ngfr* with a peak of expression during E11.5–E16.5 followed by progressive downregulation; Appendix Fig S13C). Our analysis revealed several novel transient iSC‐specific genes (*Kctd12, Prss12, Slitrk2, Moxd1*) that appeared downregulated predominantly in sympathoadrenal and limb‐derived SCs (Appendix Fig S13C and F).

All these observations point to specific spatial and temporal codes in SCPs and SCs welcoming more focused future research lines.

### Melanoma, neurofibromatosis and neuroblastoma reactivate the transcriptional signatures of “hub”/SCPs and NC

Plasticity and intra‐tumoral heterogeneity are prominent features of NC‐derived tumours, including neuroblastoma, melanoma and neurofibromatosis (Thomas *et al*, 2012; Boeva *et al*, 2017; Grzywa *et al*, 2017; Monroe *et al*, 2017). Such heterogeneity and transitions between cell types within tumours may be related to reactivated NC/SCP multipotency. This is consistent with the fact that SCPs give rise to pigment, sympathoadrenal and mesenchymal cells during normal development. To check if neuroblastoma, melanoma and neurofibromatosis tumour cells reactivate developmental cell states, we searched for SCP‐ and the NC‐specific signatures in different types of NC‐derived tumours.

First, we examined whether we could pinpoint the SCP‐like populations in already published single‐cell transcriptomic analyses of human melanoma (Durante *et al*, 2020). Using CONOS (Barkas *et al*, 2019). For this, we mapped single‐cell transcriptomic data from human uveal melanoma onto our data set (Appendix Fig S14A and C). We extracted single cells forming a trajectory from a single patient and applied CytoTRACE to this subset to correlate the progression of the tumour cells (Appendix Fig S15A). As expected, we found that the majority of the tumour cells along the entire progression identified by CytoTRACE mapped to the population of embryonic melanocytes (Appendix Fig S15A). At the same time, a significant portion of melanoma cells appeared transcriptionally similar to SCPs and the NC (Appendix Fig S14C).

Next, we performed the same analysis using available bulk transcriptomic data derived from cell lines from type 1 neurofibromatosis patients (Appendix Fig S14B). We found that all samples mapped to the mesenchymal NC branch, showing the high bias of such tumours to this fate.

Finally, we used the same approach to associate single‐cell transcriptomic data from neuroblastoma patients (Dong *et al*, 2020) with our transcriptional map of the NC lineage. The single‐cell data derived from eight different patients were analysed separately via pagoda2 and scvelo pipeline (Appendix Fig S15B). For each patient, CONOS was employed for developmental milestone and pseudotime values propagation onto the tumour data. While neuroblastoma cells are mostly similar to adrenergic cells from our developmental trajectory (Appendix Fig S14D and E), some tumour cells also mapped onto embryonic SC lineage (Appendix Fig S15C). This might suggest that some malignant cells reactivate the developmental gene expression signatures becoming similar to SCPs and the NC.

## Discussion

The term “Schwann cell precursor” (SCP) was coined by Jessen and Mirsky. depicted an NC‐derived cell type associated with the outgrowing nerves at the end of the NC migration (Jessen & Mirsky, 1991). In recent years, the NC‐like multipotency of SCPs has become apparent and attracted significant attention (Joseph *et al*, 2004; Adameyko *et al*, 2009; Nitzan *et al*, 2013; Dyachuk *et al*, 2014; Kaukua *et al*, 2014; Uesaka *et al*, 2015; Espinosa‐Medina *et al*, 2017; Furlan *et al*, 2017; Lumb *et al*, 2018; Kastriti *et al*, 2019). However, SCPs differ from the migratory NC in several aspects such as nerve association and specific morphology (Jessen & Mirsky, 1991, 1992, 2005), raising the intriguing challenge of identifying the molecular identity and cell state of SCPs. Indeed, SCPs could potentially represent nerve‐associated NCCs or, alternatively, might be a distinct cell type. The shared transcription factor code and common downstream genes have made it difficult to distinguish between these alternative scenarios (Furlan & Adameyko, 2018). For example, SCPs express transcription factors in common with migratory NC, including *Sox9*, *Sox10, FoxD3, Tfap2a/b* and *Ets1* (Britsch *et al*, 2001; Paratore *et al*, 2001; Parkinson *et al*, 2002; Nitzan *et al*, 2013; Balakrishnan *et al*, 2016).

To resolve the conundrum of SCP identity and similarity to the neural crest, we performed a single‐cell transcriptomic analysis of the entire NC lineage starting from the NC initiation until postnatal stages using Smartseq2 protocol, which produced individual transcriptomes of much higher quality as compared to droplet‐based methods. This atlas is available online for in‐depth exploration by the research community (see “Data availability” section). Our results revealed the existence of a “hub” state of transcriptional convergence, where late NCCs and SCPs mix while becoming nerve‐associated and biased towards final fates. Notably, most cells contributing to the “hub” state are SCPs from E10.5 and later stages and express a set of specific markers including *Sox8, Itga4* and other genes downregulated in the early migratory crest or differentiating Schwann cells. Consistently, targeting *Sox8* in the NC lineage resulted in a deficiency in the nerve‐associated SCPs with a concomitant proportional increase in sympathoadrenal cells. This is consistent with computational predictions, where the “hub” state transited towards SCs and other NC fates, including sympathoadrenal, enteric and melanocytes. Thus, the “hub” cells represent a continuation of the multipotent neural crest in postmigratory and nerve‐associated phases before commitment and differentiation towards definitive cell types. This fits the evolutionary scenario in which proto‐NCCs migrated from the neural tube following sensory and motor nerves in a nerve‐associated state (Ivashkin & Adameyko, 2013).

While visualisation and construction of trees of transcriptional events from individual transcriptomes help to formulate testable hypotheses regarding actual lineage transitions towards terminal states, these may not accurately reflect the actual clonal lineage tree. Based on the generated tree of transcriptional states, there are three possible scenarios of actual NCC lineage transition towards terminal fates. In the first scenario, some NCCs slide through the “hub” state without nerve association to produce differentiated cell types. The second scenario suggests that NCCs do not switch on the “hub” state gene program and instead proceed to the terminal fates while bypassing the “hub” or tunnelling through the “hub.” In the third option, numerous NCCs become nerve‐associated SCPs expressing “hub” genes, followed by the generation of definitive cell types in a nerve‐dependent fashion. The real clonal lineage portrait can be the sum of all three options mixed in different proportions. In any case, the intercalation of the transcriptomes of cells of the “hub”/SCP state between those of NCCs and downstream terminal fates suggests that SCPs are indeed a cellular state functioning as an extension of NCCs in terms of multipotency and expressed genes. The cells of this “extension state” differ from the NCCs in that they use peripheral nerves as their navigation routes and express additional gene expression programs. According to experimental evidence, these additional programs gradually increase in the neural crest‐derived “hub” cells after nerve association and are likely driven by strengthening interactions between the cells and the nerve fibres.

Based on the convergence of SCPs and late NC in the “hub” state, we expected to find similarities in the general logic of cell fate decisions reported previously for the NC. Accordingly, we analysed the fate splits (called “bifurcations”) and found the conserved three‐step model of a fate choice (co‐activation of competing modules, repulsion of competing modules and commitment), as described previously for the murine NCCs at E9.5 (Soldatov *et al*, 2019).

Recent advancements in single‐cell transcriptomics established incremental fluidity of evolving transcriptional profiles during cell lineage development (Marques *et al*, 2018; Nowotschin *et al*, 2019; Mu *et al*, 2020). While observing the fluidity of gene expression programs, we also attempted to detect rather discrete steps in cell differentiation and developmental dynamics based on the molecular transitions within the expanded NC tree. Indeed, it is useful to define cell types and their developmental progression via understanding multigenic modules (regulons) controlled by a specific gene regulatory network (GRN) subcircuit and corresponding to specific functions. The multigenic modules switch on and off rather discretely, driven by specific transcriptional activators. However, since this is happening in the background of other many programs with overlapping dynamic behaviour, the sum of all transcriptional up‐ and downregulation event results is a smooth incremental change of the overall gene expression during differentiation.

To disentangle this fluidity into a set of overlapping discrete programs that help to separate the “hub” from the early migratory crest and differentiating SCs at the level of regulation, we utilised the approach of elucidating orchestrated gene expression modules‐regulons (Soldatov *et al*, 2019; Van de Sande *et al*, 2020). Metaregulons are groups of regulons with co‐regulated or correlated expression profiles. We took advantage of the regulon concept to assist in defining the transient NC, “hub”/SCP and iSC states towards the terminal stages of SC development. Our analysis of regulons revealed how the uninterrupted and smooth NC and SCP differentiation trajectory is divided into discrete steps, which assisted the regulation‐based definition of the “hub.” Furthermore, we discovered that *Ets1* metaregulon is specifically active in both emerging NCCs and SCPs before their transition towards mature SCs or commitment to neuronal fates, suggesting its role in multipotency maintenance. Indeed, the time window of this metaregulon coincides with the actual multipotency of the NC and SCP populations, which is evident from reported lineage‐tracing experiments (Dyachuk *et al*, 2014; Furlan *et al*, 2017; Kastriti *et al*, 2019).

After defining the converging transcriptional states of the NC and SCPs based on the discrete activity of identified regulons, we set out to investigate the transition from SCPs towards a diversity of mature Schwann cell types. Our results showed transcriptional and regulatory paths towards myelinating, non‐myelinating, neuromuscular junction terminal SCs and endoneurial fibroblasts. For instance, endoneurial fibroblasts arise from the nerve‐associated *Dhh*‐expressing cells that lose contact with axons during late embryonic and early postnatal stages of development (Parmantier *et al*, 1999; Joseph *et al*, 2004; Sharghi‐Namini *et al*, 2006). According to our results, the profile of *Dhh* expression reconciles the current and previous findings and provides the precise molecular identity of progenitors biased towards endoneurial fibroblasts.

SC differentiation is guided by the type and diameter of the nerve fibres (key molecules in this process are NEUREGULIN‐1 type III and endothelin) and possibly other currently unknown spatial signals (Brennan *et al*, 2000; Taveggia *et al*, 2005). Following this idea, we attempted to reveal spatial aspects of heterogeneity of SCPs and more advanced glial progenitors. Our results clearly showed that nearly all SCPs belonging to the autonomic nervous system express markers biasing them towards neuronal or neuroendocrine chromaffin fates, such as *Phox2b* and *Ascl1*. On the other hand, SCPs and more advanced glial progenitors that cover sensory nerves in the skin, specifically express *Nr4a2* nuclear receptor gene. In line with these findings, some degree of spatial heterogeneity of SCPs has been reported previously, as evident from the proximal‐to‐distal gradient of SOX2 in SCPs, which affects the SCP‐to‐melanocyte conversion (Adameyko *et al*, 2009, 2012; Nitzan *et al*, 2013).

Finally, the heterogeneity of SCPs might be related to transcriptional states leading to a spectrum of embryonic and adult tumours, including melanoma, neuroblastoma and neurofibromatosis. Furthermore, examples of extra‐cutaneous melanomas and melanoma cells with NC‐like phenotype favour the potential SCP‐dependent origin of some tumour populations (Schatton *et al*, 2008; Boiko *et al*, 2010; Civenni *et al*, 2011; Kaufman *et al*, 2016; Diener & Sommer, 2021). In the case of neurofibromatosis type 1, the tumour‐initiating cells are located within embryonic nerves and may reflect specific SCP populations or other cells of the NC or SC lineage, as reviewed by Li *et al* (2020). Since SCPs generate chromaffin and sympathetic cells (Furlan *et al*, 2017; Kastriti *et al*, 2019), their transitory states might be essential for understanding the origin and plasticity of malignant cells in neuroblastoma (Dong *et al*, 2020; Hanemaaijer *et al*, 2021; Kameneva *et al*, 2021). To address this, we compared the transcriptional states within the NC lineage with type 1 neurofibromatosis, uveal melanoma and neuroblastoma cell populations. Our results revealed subsets of cells with a “reverted” developmental‐like phenotype, which suggests that heterogeneous tumours might contain NC‐like or SCP‐like malignant cells capable of migration via the nerves and potentially metastasizing to the other parts of the body. These results fit the current trend of discovering the role of developmental genetic modules in tumour evolution, spread and survival, including the dynamic transitions between malignant cell populations under treatment (Chakrabarti *et al*, 2012; Boeva *et al*, 2017; Kameneva *et al*, 2021).

In summary, our results revealed the transcriptional identity and heterogeneity of SCPs and other cells of the NC lineage presented here as an open and accessible high‐resolution atlas (7,000–8,000 genes revealed per cell on average). Based on these computational atlas, experimental validations and functional experiments, we defined “hub”/SCPs as NC‐like cells in a multipotent state with multiple detectable fate biases and additional gene expression programs. These programs provide multipotency and control recruitment of nerve‐associated “hub”/SCP cells to other fates. At last, the comparisons of the single‐cell atlas of the entire embryonic NC and “hub” lineage with pathological states provided methodological examples that can be relevant to questioning the cell of origin for specific NC tumour subtypes or for predicting the mechanisms mediating intra‐tumoral plasticity.

## Materials and Methods

### Mouse husbandry, strains and genetics

All mouse work was performed under a permit approved by the Ethical Committee on Animal Experiments (Stockholm North committee) of Sweden or the Ethical Committee for Advice and Assessment of Research Projects on Animals of the Medical University of Vienna (Ethik‐Kommission der MedUni Wien zur Beratung und Begutachtung von Forschungsprojekten am Tier) of Austria adhering to Swedish, Austrian and European regulations and guidelines for animal experimentation.

*Ascl1::CreERT2* mice were received from The Jackson Laboratory, stock number 012882 (full strain name: Ascl1tm1.1(Cre/ERT2)Jejo/J). *Sox10::CreERT2* mice are available from the laboratory of Vassilis Pachnis (The Francis Crick Institute, UK) under a material transfer agreement with the institution. *Phox2b‐Cre;R26* ^*TOMATO*^ embryos were received from the Jean‐François Brunet laboratory (D'Autreaux *et al*, 2011). *Ret::CreERT2* mice were received from D. Ginty laboratory (Harvard University, USA; http://www.informatics.jax.org/allele/MGI:4437245). *Plp1::CreERT2* were received from U. Suter laboratory (ETH Zurich, Switzerland; http://www.informatics.jax.org/allele/MGI:2663093). *R26R* ^*YFP*^ and *R26R* ^*TOMATO*^ mice were received from The Jackson Laboratory (stock number 006148 and 007914, respectively). *Wnt1‐Cre* mice were received from The Jackson Laboratory, stock number 009107 (full strain name B6.Cg‐Tg(Wnt1‐cre)11Rth Tg(Wnt1‐GAL4)11Rth/J). *Isl1‐Cre* mice were received from The Jackson Laboratory (stock number 024242). *Nr4a2* knockout embryos and postnatal tissue were received from the laboratory of Thomas Perlmann (available through The Jackson Laboratory, stock number 017859).

### Tamoxifen‐induced lineage tracing

For all experiments using embryonic tissue, following time‐mating, the day of vaginal plug detection was considered E0.5. In the case of postnatal collections, the day of birth was considered as P0. In the case of inducible lineage tracings (*Plp1::CreERT2*, *Sox10::CreERT2* and *Ascl1::CreERT2* strains), tamoxifen (Sigma, Cat No T5648) was dissolved in corn oil (Sigma, Cat No 8267) and delivered via intraperitoneal (i.p.) injection to pregnant females (0.1 mg/g of body weight). For inducible lineage‐tracing experiments intended for tissue to be subjected to single‐cell transcriptomics, tamoxifen was administered 48 h prior to tissue harvesting.

### Tissue collection from mice and preparation for downstream analysis

Pregnant females or young pups were sacrificed following isoflurane overdose with cervical dislocation. Embryonic or postnatal tissue was dissected within 15 min of the collection and placed on ice. Dissected tissue was fixed with 4% PFA, 4°C, with mild agitation for 2–16 h depending on the developmental stage. Following washes with 1× PBS, tissue was submerged into 30% sucrose in 1× PBS for cryoprotection for 24–48 h, 4°C before embedding in O.C.T. and snap freezing. Frozen tissue blocks were stored at −20 to −80°C until cryosectioning took place. Cryosections of 14–16 μm were collected onto SuperFrost Plus slides, dried at room temperature and stored at −20°C until further use.

### Avian embryos and Sox8 knock down

#### Embryos

Wild‐type chicken embryos were obtained from Rhode Island Red hens (Sunstate Ranch). Eggs were incubated in humidified incubators at 38°C to the desired stage. Embryos were staged according to Hamburger & Hamilton (1992).

#### Making Crispr constructs

Crispr constructs were designed and made according to the protocol by Gandhi *et al* (2021). The plasmid contains a sequence for Cas9, citrine and a guide RNA. The sequence for the guide RNA used in this study to knock down SOX8: 5′ ‐ ATCCACCTTAGCGCCCAGCG – 3′. The control plasmid used contained a synthetic DNA construct not found in the chicken genome in place of the guide RNA: 5′ ‐ GCACTGCTACGATCTACACC – 3′. Cripr plasmids for all experiments were used at a final concentration of 2.5 μg/μl, diluted in EB buffer. To enable visualisation of the injection solution, 0.5 μl of 2% blue food dye was added per 10 μl injection mix.

#### Verifying Cripr construct

HH4 stage embryos were electroporated *in vitro* and cultured overnight. The Cripr construct was injected between the epiblast and the membrane on the right half of the area pellucida. Electroporation was achieved using a flat electrode placed above the injected area with the following settings: 5.7 volts, 50 ms pulses, 5 pulses, off for 100 ms between pulses. Embryos were then cultured overnight until HH9‐10 using the EC method (Chapman *et al*, 2001). The expression pattern of SOX8 was visualised by HCR. Electroporated cells were identified by HCR against citrine. The “Molecular Technologies” protocol for HCR was used.

#### Embryology

HH10‐11 stage embryos were injected and cultured in ovo. The Crispr plasmid was injected into the neural tube via a capillary needle. The needle was used to pierce the dorsal part of the neural tube at the rostral end of the trunk. The solution was injected until the trunk was filled to the posterior neural pore. Unilateral electroporation of the trunk was achieved using a two‐pronged electrode, with the electrodes placed 4 mm apart, either side of the neural tube with the following settings: 15 volts, 50 ms pulses, 5 pulses, off for 100 ms between pulses. Embryos were then cultured for 3 days in ovo at 38°C until they reached HH24‐25.

#### Histology

Embryos were fixed in 4% PFA in PB overnight at 4°C, rinsed three times in PBS with 0.3% triton, then washed overnight at 4°C. Embryos were equilibrated in 5% sucrose in PBS for 2–4 h at 4°C then in 15% sucrose overnight at 4°C. Embryos were transferred to melted gelatin and allowed to equilibrate overnight at 38°C. Embryos were then snap frozen using liquid nitrogen and stored at −80°C for at least overnight before sectioning using a cryostat into 16 μm sections. Sections were kept at room temperature overnight prior to immunohistochemistry staining.

#### Immunohistochemistry

Slides were left in PBS at 42°C for 7 min to remove gelatin, washed in PBS with 0.3% triton‐X100 twice for 15 min at room temperature and blocked (10% donkey serum) for 1 h at room temperature. The following primary antibodies were diluted in block at the following concentrations and kept on slides for 2 days at 4°C: rabbit anti‐Tyrosine Hydroxylase (# AB152, Millipore) 1:500; mouse anti‐Islet (39.4Ds, DSHB) 1:100; rabbit anti‐SOX10 (# HPA068898‐100UL, Sigma) 1:500; mouse anti‐SOX10 (# SC365692, Santa‐Cruz Biotechnology) 1:100; mouse anti‐pH3 (# ab14955, Abcam) 1:500. Slides were then washed three times for 15 min in PBS with 0.3% triton‐X100. The following secondary antibodies were diluted in 10% donkey serum block at the following concentrations and kept on slides overnight at 4°C: Alexa Fluor 647 donkey anti‐rabbit IgG (# A31573, Invitrogen) 1:1,000; Alexa Fluor 647 donkey anti‐mouse IgG (# A31571, Invitrogen) 1:1,000; Alexa Fluor 568 donkey anti‐rabbit IgG (# A10042, Invitrogen) 1:1,000; Alexa Fluor 568 donkey anti‐mouse IgG (# A11037, Invitrogen) 1:1,000; Alexa Fluor 488 donkey anti‐goat IgG (# A11055, Invitrogen) 1:1,000. Slides were then washed two times for 15 min in PBS with 0.3% triton‐X100, one time in PBS for 15 min and for 7 min in PBS with DAPI (1 μg/ml). Slides were rinsed in PBS and mounted using Fluoromount‐G (# 0100–01, Southern Biotech).

#### Imaging and image processing

Immunostained sections were imaged using a Zeiss Imager MZ with an ApoTome module. Images were processed using the “*Fiji*” software, using the “Cell Counter” plugin for counting cells. Four to five sections were quantified per embryo from the forelimb axial level, and 2–4 embryos were quantified per condition.

### Immunofluorescent staining on mouse tissue

Slides were equilibrated to room temperature, and antigen retrieval was performed using a steam cooker by submerging the slides in 1× Target Retrieval Solution (Dako, S1699) for 20 min. Sections were washed three times in PBS with 0.1% Tween‐20 (1× PBST) and incubated at 4°C, overnight with primary antibodies diluted in 1× PBST. Next, sections were washed in 1× PBST and incubated with secondary antibodies diluted in 1× PBST at room temperature for 1 h, washed three times in 1× PBST and mounted using Mowiöl mounting medium.

Primary antibodies used: Goat anti‐GFP (1:500, Abcam, #ab6662), chicken anti‐GFP (1:500, Aves Labs Inc., #GFP‐1020), chicken anti‐mCherry (1:1,000, EnCor Biotech, #CPCA‐mCherry), goat anti‐SOX10 (1:500, Santa‐Cruz, #sc‐17,342), goat anti‐SOX10 (1:1,000, R&D systems, #AF2864), chicken anti‐NF200 (1:1,000, Abcam, #ab4680), chicken anti‐P0 (1:1,000, Abcam, ab134439), mouse anti‐HUC/D (1:100, ThermoFisher Scientific, #A‐21271), mouse anti‐ISL1 (1:250, DSHB, clone 39.4D5‐s), rabbit anti‐S100B (1:500, Synaptic Systems, #287003), mouse anti‐NEUROFILAMENTS (1:100, DSHB, clone 2H3), mouse anti‐βIII Tubulin (1:1,000, Promega, clone G712A), rabbit anti‐OCT6 (1:500, Abcam, ab272925).

When DAPI (Sigma, D9542) was used, it was applied on the sections simultaneously with the secondary antibodies at a concentration of 0.5 mg/ml. For detection of the primary antibodies, secondary antibodies raised in donkeys and conjugated with Alexa‐488, ‐555 and ‐647 fluorophores were used (1:1,000, Molecular Probes, ThermoFisher Scientific).

### RNAscope® *in situ* hybridization

*In situ* hybridization using the RNAscope® Fluorescent Multiplex Assay kit and a commercially available probe against mouse *Nr4a2* (Cat No. 423351), *Itga4* (Cat No. 542901), *Serpine2* (Cat No. 435241), *Sox8* (Cat No. 454781), *Htr3a* (Cat No. 411141‐C3), *Itga8* (Cat No. 417941), *Pou3f1* (Cat No. 436421‐C2), *Cpm* (Cat No. 1121871‐C3) was performed using cryosections of fixed frozen tissue according to the manufacturer's instructions.

### Microscopy

Images were acquired using LSM 780, LSM 880 Zeiss confocal microscopes equipped with 20×, 40× and 63× objectives or a Zeiss Imager MZ with an ApoTome module. Images were acquired in the lsm format and processed with ImageJ for export as tiff files. Figures were compiled using Adobe Photoshop and Illustrator.

### Quantification of RNAscope® *in situ* hybridization

Following export as RGB tiff files, images were cropped by annotated region and loaded onto cellpose for segmentation, using cytoplasm model, SOX10 signal and a diameter parameter of 20 pixels (Stringer *et al*, 2021). From each segmented cell, we calculated (i) the mean intensity of SOX10 and (ii) the background‐corrected mean intensity of RNAscope®‐detected marker. To filter out nonglial cells, two different approaches were used depending on the collected data in a defined region. When the distribution of the mean intensity of SOX10 per cell presented two clear distributions, the SOX10^low^ population was removed (corresponding to cells biased to sensory fate) using a SOX10 mean intensity threshold. When the region contained a specific population of SOX10^low^ and very high intensity of the RNAscope®‐detected marker, with a high correlation between the two values, these cells were considered as blood cells. These contaminants were removed either by applying k‐means with two clusters on the scaled mean intensities of SOX10 and RNAscope®‐detected marker and discarding the blood cell cluster when successfully captured. A SOX10 mean intensity threshold was employed for unsuccessful clustering results. Mean intensities results were then combined and annotated by location and developmental day.

### Statistical analysis and sample size

Sample size was decided according to accepted practices in the field and initial preliminary experiments to estimate variability within the same litter while adhering to the use of the minimum number of animals. Analysis of statistical significance (Fig 4) was performed using GraphPad Prism 7 and the Holm‐Sidak method (α = 0.05; multiple *t*‐tests, unpaired) or unpaired *t*‐test with a two‐tailed *P*‐value. Blinding was not applied, as most validations were performed on wild‐type tissue. In the case of the analysis of *Sox8* KD (Fig 4), quantification was performed the same way on all chick embryos.

### Single‐cell suspension preparation, fluorescence‐activated cell sorting (FACS) and single‐cell transcriptomic sequencing

Collection and dissection of embryonic and postnatal traced tissue were carried out using a stereotactic microscope equipped with a fluorescent light source to enrich the tissue in relevant cells. Dissected tissue was collected in 1× PBS on ice, and either 0.05% Trypsin/0.02% EDTA or 2 mg/ml collagenase P was used for enzymatic digestion of embryonic or postnatal tissue, respectively, incubated at 37°C for 5–20 min depending on the developmental stage. Following enzymatic digestion, the tissue was triturated with a P‐1000 and then P‐200 pipette until dissociation, and cold 10% FBS was added to quench the enzymes. The cell suspension was spun down, 300 × *g*, 5 min, 4°C and washed three times with 1× PBS. Finally, the cellular suspension was filtered through a 40 μm‐pore size cell strainer and collected into a FACS tube on ice.

Single TOMATO^+^ cells were sorted into 384‐well plates prefilled with lysis buffer according to the previously published SmartSeq2 protocol (Picelli *et al*, 2014) using a BD FACSAria Fusion Cell Sorter B5/R3/V3 system with a three‐laser configuration (488, 633 and 405 nm) and 16 fluorescence detectors. Single‐cell transcriptomic sequencing was performed as previously described (Picelli *et al*, 2014).

### Generation of count matrices, QC and filtering

The single‐cell transcriptome data were generated at the Eukaryotic Single‐cell Genomics facility at Science for Life Laboratory in Stockholm, Sweden. The samples were analysed by first demultiplexing the fastq files using deindexer (https://github.com/ws6/deindexer) using the nextera index adapters and the 384‐well plate layout. Individual fastq files were then mapped to mm10_ERCC genome using the STAR aligner using 2‐pass alignment (Dobin *et al*, 2013). Reads were filtered for only uniquely mapped and were saved in BAM file format; count matrices were subsequently produced. Estimated count matrices were gathered and converted to anndata python object prior to scanpy processing. Cells with 5 × 10^4^ ≤ transcripts ≥ 6 × 10^4^, 1,000 ≤ genes ≥ 10,000 or ≤ 15% of ERCC reads were kept.

### Overview and selection of developing glial trajectories

The analysis of the count matrix was performed using the scanpy and scFates python packages with scFates used for a function detecting overdispersed genes following the approach proposed by pagoda2 R package (Fan *et al*, 2016). PCA was performed on the scaled count matrix subset by overdispersed genes. A nearest neighbour graph was generated with 30 neighbours and on 30 PC components. 2D embedding and Clusters were then identified from NN graph using Leiden algorithm (Traag *et al*, 2019) and UMAP, respectively (preprint: McInnes *et al*, 2020). Cells in clusters expressing *Sox10* were kept, as well as linked *Isl1* ^+^ neurogenic clusters. The subset count matrix was further processed in a similar way at the difference of using 15 neighbours for the NN graph. At this stage, scrublet was run on the filtered count raw matrix, and two Leiden clusters enriched with high doublet scores were removed from the data set. As a final step, Palantir (Setty *et al*, 2019) was applied to generate diffusion maps with a NN graph with neighbours on the first 50 PCs. The first nine diffusion components were used for generating a new NN graph, with 40 neighbours, followed by PAGA, which was then used as the basis for a final UMAP embedding (Wolf *et al*, 2019). Using these diffusion components, the count matrix was then imputed using Palantir's function MAGIC. The subset count matrix was used as input for the CytoTRACE pipeline (Gulati *et al*, 2020; using default parameters).

### Cell type assignment and identification of cells assigned to the “hub”

Cell types were assigned by performing gene scoring using the MAGIC‐imputed gene expression known markers (see Appendix Fig S3). Cells were annotated by setting a specific threshold for each cell type. Any cells assigned to two or more cell types were discarded from annotation. “hub” cells were identified as being a member of a Leiden cluster containing more than 80% of unannotated cells. “hub” markers were identified by performing differential gene expression via Wilcoxon rank‐sum test, comparing the “hub” cells to the rest.

### RNA velocity

BAM files from each plate were processed using python command‐line velocity tool (La Manno *et al*, 2018) using run‐smartseq2 command with GENCODE M21 genome and repeat masker annotation files, leading to a loom file for each plate containing spliced and unspliced transcript counts. The loom file was combined and subset to the previously filtered out cells. Using scvelo tool on python (Van de Sande *et al*, 2020), genes with less than 20 spliced counts or unspliced counts were excluded, and the 2,000 top highly variable genes were kept, from which spliced and unspliced counts were then knn smoothed with 30 neighbours. The smoothed values were used to calculate velocity values. The data set was then used as input to BRIE2 pipeline (Huang & Sanguinetti, 2021) to identify differential momentum genes, using the previously measured CytoTRACE value as a continuous covariate. BRIE2 detected 120 genes displaying differential momentum (FDR < 1 × 10^−40^), which were used to generate the velocity graph and were computed using default parameters. Extrapolated states were then projected on the UMAP embedding produced during the initial analysis.

### SCENIC analysis

The SCENIC pipeline was applied using the python package pySCENIC (Aibar *et al*, 2017; Van de Sande *et al*, 2020). Firstly, the normalised count matrix from the pagoda2 pipeline was used as input, combined with a list of known transcription factors (TFs), to generate regulons based on correlation with putative target genes. Next, using the generated adjacency matrix combined with cisTraget databases (mm10 500bpUp100Dw and TSS+/−10kbp), the regulons are refined by pruning targets that do not present an enrichment for a corresponding motif of the TF. Lastly, cells were scored for each regulon with a measure of recovery of target genes from a given regulon. These AUC scores are then used to generate diffusion maps using Palantir (run_diffusion_maps, knn = 40), with the first seven diffusion components used to generate a UMAP embedding. In downstream analyses, the AUC scores of the regulons were weighted according to the log10 fpm expression of the related TF. In such cases, the weight is equal to the log10 fpm value between 0 and 1 and then set to 1 if log10(fpm) > 1.

### Pseudotime tree inference

These steps were performed using scFates v0.4.1, a python package built‐in continuity of the crestree R package (Soldatov *et al*, 2019). The trajectory inference was performed in two stages. A 3,000‐nodes tree was fitted, using SimplePPT approach (Mao *et al*, 2015) on the diffusion space using tl.tree function (scFates, method = “ppt,” sigma = 0.0005, lambda = 200, metric = “euclidean”). Using the developmental time and CytoTRACE score two roots were selected, indicating the vagal and posterior trunk NC. The tree is composed of a soft assignment matrix R, where each node of the tree contains a value for all cells, each cell has an assignment strength to a node between 0 and 1, with 1 being the closest to the node. Pseudotime value was then generated as a distance on the tree from the selected roots and projected to cells using R assignment matrix.

For the glial trajectory, the first tree only captured nmSC and endoneurial fibroblasts. To capture and justify the existence of the mSC fate, the cells were the first subset and processed via cell rank, which used RNA velocity information weighted with cell connectivities. Detected macrostates using Leiden clustering identified nmSC, mSC and endoneurial Fibroblast states, which were selected as terminal states and for absorption probabilities computation. The results were then converted into a 300 nodes principal tree via scFates, which was used as input the absorption probabilities simplex from cell rank and CytoTRACE values. Finally, in order to capture the tSC population and have a more biologically meaningful tree, these cells were further re‐analysed by fitting a tree on the UMAP embedding, allowing to capture all four cell types.

To facilitate the representation of the lineages, highlight bifurcations and roots and endpoints, we employed a concept of milestones, inspired by Saelens *et al* (2019). Specifically, milestones are either a tip or a fork of the fitted tree and cells are assigned to the closest milestone in pseudotime distance.

### Testing for features associated with the tree

Feature expression was modelled as a function of pseudotime in a branch‐specific manner, using cubic spline regression $\exp_{i}∼t_{i}$ for each branch independently. This tree‐dependent model is then compared with the unconstrained model $\exp_{i}∼1\;$using *F*‐test. *P*‐values were then corrected for multiple testing, features were considered significant if FDR < 0.0001.

log10(fpm) count matrix was used to test with GAM to model which genes are significantly changing along the whole tree (scFates, tl.test_association, default parameters), with significant genes being fitted using GAM to obtain smoothed trends (scFates, tl.fit, default parameters). Whole tree was also used in combination with SCENIC‐derived AUC score, to detect significantly changing regulon activities. To do so, AUC scores were tested (scFates, test_association, A_cut = 0.025) and fitted (scFates, tl.fit, default parameters) via GAM.

#### Bifurcation analysis

Branch‐specific genes were first detected via amplitude testing using the following GAM model:

$$g_{i}∼s\left(\text{pseudotime}\right)+s\left(\text{pseudotime}\right):\text{Branch}+\text{Branch}$$

From *s(pseudotime): Branch* interaction term, *P*‐values were extracted and then corrected for multiple testing (scFates, tl.test_fork, fdr_cut = 0.1).

Then, each significant gene was tested for its upregulation along the path from progenitor to terminal state, using the linear model $g_{i}∼\text{pseudotime}$. Differentially expressed genes were then assigned between two postbifurcation branches with fdr < 0.05 and defined differences in expression cutoffs, using tl.branch_specific (scFates, cutoffs were specifically set for each bifurcation). Finally, the pseudotime of activation was estimated by separating the trajectory into 10 bins and by calculating the relative expression rate at a specific bin:

$$r\left(b_{t}\right)=\frac{f\left(b_{t+1}\right)\;-\;f\left(b_{t-1}\right)}{\max\left(f\right)\;-\;\min\left(f\right)}$$

Where *f*(*b*) is the mean fitted expression at a specific bin, if the rate was higher than a defined threshold, the gene was considered to activate at the pseudotime value of the related bin.

To analyse molecular mechanisms of cell fate biasing, cell composition was approximated by a sliding window of cells along the pseudotime axis, cells were manually selected in order to represent the different steps of differentiation. The local gene–gene correlation reflecting the coordination of genes around a given pseudotime *t* was defined as a gene–gene Pearson correlation within each window of cells. The local correlation of a gene g with a module was assessed as a mean local correlation of that gene with the other genes comprising the module. Similarly, intra‐module and inter‐module correlations were taken to be the mean local gene–gene correlations of all possible gene pairs inside one module, or between the two modules, respectively (scFates, tl.slide_cors, default parameters).

### Neural crest to Schwann cell trajectory analysis and glial heterogeneity

The linear trajectory from posterior neural crest cells to immature Schwann cells was a subset from the main tree using the tree milestones. Pseudotime was robustly calculated by running 100 probabilistic mappings where in each run a cell was assigned to a node by using their assignment values as probabilities (see section “Pseudotime tree inference”). Regulons associated with the trajectory were detected over these 100 mappings using tl.test_association (scFates, A_cut = 0.02) on AUC score matrix from SCENIC pipeline. Next, associated regulons were fitted using tl.fit (scFates, default parameters), and fitted regulons were clustered using tl.cluster using cosine distance metric and 40 neighbours.

Similar to bifurcation analysis, two groups of target genes from Six1(+) and Ets1(+) regulons, respectively, were used for correlation analysis, where cell composition was approximated by a sliding window of cells along the pseudotime axis between iSCs and NCC. The local gene–gene correlation reflecting the coordination of genes around a given pseudotime *t* was defined as a gene–gene Pearson correlation within each window of cells.

To focus the analysis on the SCPs and Schwann cell population, the tree generated previously (see section “Pseudotime tree inference”) was subset to keep only SCPs, SCs, tSCs and endoneurial fibroblasts. Differential gene expression analysis was performed on the tree milestones using Wilcoxon rank‐sum test from scanpy package via tl.rank_genes_groups (scanpy, method = “Wilcoxon”).

### Neural crest and Schwann cell lineage comparison with transcriptomic data from tumours

#### Comparison with neurofibromatosis type 1 data

Bulk RNAseq data from neurofibromatosis type 1 plexiform neurofibroma‐derived Schwann cells were obtained as a tpm matrix and were log‐transformed from a published data set (Ferrer *et al*, 2018). Mouse gene names from the SS2 data were converted to human symbols to allow comparison. Both data sets were subsets to keep only common gene names. Pairwise correlation between cells from both data sets was computed using either all common genes or genes presenting a low correlation with cell cycle aspect score identified via pagoda2 pipeline (< 0.1). Bulk data points were projected onto the UMAP embedding of the mouse‐derived SS2 data set by building a kNN graph from the pairwise correlations with five neighbours and by computing a weighted average of both dimension coordinates with the weights of the connectivity between neighbours.

#### Comparison with uveal melanoma data

Using the raw data from a published study on scRNAseq analysis of human uveal melanoma (Durante *et al*, 2020), count matrices from all patients were combined and preprocessed with scanpy python package. Filtering was performed as follows: cells having between 200 and 8,000 detected genes, less than 10% of mitochondrial genes and more than 400 UMIs were kept. Filtered data were normalised to a target sum of UMIs of 10e6 and log normalised (base 10). Highly variable genes were detected using cellRanger approach and were used to compute PCA on scaled data. kNN graph was generated with 50 neighbours on the 10 first PCs, followed by UMAP embedding. Upon inspection of *Sox10* and *Mitf* expression, the data were subset into a single trajectory. Following the same strategy as the original paper, we identified tumour cells with MLANA, MITF and DCT expression. We focused on PRAME^+^ class 2 tumour cells from a single patient and data kept originating from a single patient to avoid batch effect‐related aspects, and cycling cells were removed. Lastly, human gene names were converted to mouse symbols to allow integration. PCA was performed on this subset, and kNN graph was generated with 50 neighbours on the 5 first PCs. Palantir was also applied to the PCA space, and tSNE embedding was generated on the multiscale diffusion space. CytoTRACE was applied to the raw count matrix with default parameters. The melanoma trajectory was reprocessed with pagoda2 pipeline (basicP2proc function, default parameters) and CONOS (Barkas *et al*, 2019) was performed (*k* = 15, *k*.self = 5, space = “PCA,” ncomps = 30) between the melanoma trajectory and the complete mouse‐derived NCC and Schwann cell trajectory. Cell‐type assignment labels from our developmental data set were transferred to the melanoma trajectory. Projection of the cells onto developmental embedding was performed by computing the weighted average of the developmental UMAP coordinates, using the CONOS‐derived connectivities as weights.

#### Comparison with neuroblastoma

Using the raw data from a published study on scRNAseq analysis of neuroblastoma (Dong *et al*, 2020), count matrices from all patients were combined and preprocessed with scanpy python package. Filtering was performed as follows: cells having between 200 and 10,000 detected genes, less than 10% of mitochondrial genes and more than 400 UMIs were kept. Filtered data were normalised to a target sum of UMIs of 10e6 and log normalised (base 10). Highly variable genes were detected using the cellRanger approach and were used to compute PCA on scaled data. kNN graph was generated with 30 neighbours on the 30 first PCs, followed by UMAP embedding. Data were then subsetted to the existing published cell‐type annotation and separated according to patients to avoid the batch effect. Lastly, human gene names were converted to mouse symbols to allow integration. PCA was performed on this subset, and kNN graph was generated with 50 neighbours on the 5 first PCs. Palantir was also applied to the PCA space, and tSNE embedding was generated on the multiscale diffusion space. CytoTRACE was applied to the raw count matrix with default parameters. CONOS (Barkas *et al*, 2019) was performed (*k* = 15, *k*.self = 5, space = “PCA,” ncomps = 30) between the neuroblastoma data sets and the complete mouse‐derived NCC and Schwann cell trajectory. Cell‐type assignment labels from our developmental (SmartSeq2) data set were transferred to the melanoma trajectory. Projection of the cells onto developmental embedding was performed by computing the weighted average of the developmental UMAP coordinates, using the CONOS‐derived connectivities as weights.

### Comparative validation of terminal Schwann cells and satellite glia populations

#### Validation of tSC population using published NMJ bulk RNAseq data

Published bulk RNA seq data containing nonmyelinating, synaptic glia of the neuromuscular junction (NMJ; Mapps *et al*, 2022) was downloaded and processed as follows: fastq files were aligned with STAR tool and count matrices generate via featureCounts using mouse genome annotation gencode M27. Count matrix was then analysed via DESeq2 pipeline, where the covariate to compare was FACS sorting of cells being S100β‐GFP^+^ or S100β‐GFP^+^/NG2‐dsRed. Differential gene expression was generated, and on the top of the results were highlighted five main top markers defining tSC in our data set.

#### Validation of satellite glia using published scRNAseq data set

Published scRNAseq data containing cells from mouse DRG and SCG were downloaded and processed via the kallisto bus pipeline (Bray *et al*, 2016; Melsted *et al*, 2021) using mouse genome GRCm38 release 97. Cells with less than 200 genes and more than 7.5% of mitochondrial proportion were filtered out. Highly variable genes were detected via scFates wrapper for pagoda2 over dispersion detection approach. PCA was generated from the scaled count matrix and used for the NN graph with 20 PCs and 30 neighbours. Cluster‐defining satellite glia (Plp1^+^/Fabp7^+^) was selected and only cells from DRG were used for subsequent analysis. The same process was applied to that subset, and Leiden clustering generated five clusters that could be easily annotated. Mapping was applied by processing that subset and our developmental data set to CONOS pipeline, which consisted in reprocessing with pagoda2, alignment in PCA space (20 PCs) using 15 neighbours and five self‐neighbours, and propagation of cell‐type assignment labels generated from our developmental data (see section *Cell‐type assignment and “hub” cell identification*). Furthermore, projection of the cells onto developmental embedding was performed by computing the weighted average of the developmental UMAP coordinates, using the CONOS‐derived connectivities as weights.

## Author contributions

**Maria Eleni Kastriti:** Conceptualization; data curation; formal analysis; supervision; validation; investigation; visualization; writing – original draft; project administration; writing – review and editing. **Louis Faure:** Resources; data curation; software; formal analysis; investigation; visualization; methodology; writing – original draft; writing – review and editing. **Dorothea Von Ahsen:** Formal analysis; validation; investigation; visualization; methodology. **Johan Boström:** Resources; investigation; writing – review and editing. **Thibault Gerald Bouderlique:** Validation; investigation; visualization; writing – review and editing. **Tatiana Solovieva:** Data curation; formal analysis; investigation; visualization; methodology; writing – review and editing. **Cameron Jackson:** Investigation. **Marianne Bronner:** Resources; supervision; funding acquisition; methodology; writing – review and editing. **Dies Meijer:** Writing – review and editing. **Saida Hadjab:** Resources; funding acquisition; writing – review and editing. **Francois Lallemend:** Resources; funding acquisition; writing – review and editing. **Alek Erickson:** Resources; writing – original draft. **Marketa Kaucka:** Resources; writing – review and editing. **Viacheslav Dyachuk:** Resources; writing – review and editing. **Thomas Perlmann:** Resources; writing – review and editing. **Laura Lahti:** Resources; writing – review and editing. **Jan Krivanek:** Resources; investigation; writing – review and editing. **Jean‐Francois Brunet:** Resources; writing – review and editing. **Kaj Fried:** Writing – review and editing. **Igor Adameyko:** Conceptualization; supervision; funding acquisition; writing – original draft; project administration; writing – review and editing.

## Disclosure and competing interests statement

The authors declare that they have no conflict of interest.

## Supporting information




Appendix

Expanded View Figures PDF

Dataset EV1

## Data Availability

Newly generated single‐cell transcriptomic data and previously published data sets have been uploaded under GSE201257 (https://www.ncbi.nlm.nih.gov/geo/query/acc.cgi?acc=GSE201257). Two pagoda2 web applications are available on the following link: https://adameykolab.srv.meduniwien.ac.at/glia_gene_umap/ with the (gene‐based embedding, and https://adameykolab.srv.meduniwien.ac.at/glia_scenic_umap/ with (SCENIC‐based UMAP embedding). Cropped images used for quantification of the “hub” markers *Itga4*, *Serpine2* and *Sox8* following RNAscope® *in situ* hybridization can be found at DOI 10.6084/m9.figshare.19620102. Code and data for downstream analysis are found on the following repository: https://github.com/LouisFaure/glialfates_paper.
