# Supplementary material for: Schwann cell precursors represent a neural crest‐like state with biased multipotency
Source: EMBO J. 2022 Jul 11;41(17):e108780. doi: 10.15252/embj.2021108780 (PMC9434083; doi:10.15252/embj.2021108780)
Supplement: Supplementary file 1 — Appendix [file EMBJ-41-e108780-s002.pdf]

Appendix

Table of contents

Figure S1. Technical overview of the data set.
Figure S2. Overview of total data set consisting of neural crest cells and downstream lineages.
Figure S3. UMAPs of known markers used for cell type annotation.
Figure S4. Mapping the signature of satellite glia and boundary cap cells (BCCs) on that of the neural crest and Schwann cell lineage.
Figure S5. Mapping the expression of the hub marker *Itga4* in neural crest and post-neural crest stages.
Figure S6. Mapping the expression of the hub marker *Serpine2* in neural crest and post-neural crest stages.
Figure S7. Mapping the expression of the hub marker *Sox8* in neural crest and post-neural crest stages.
Figure S8. Analysis of regulon activity from cranial or trunk neural crest subpopulations to immature Schwann cells.
Figure S9. Detailed bifurcation analyses of the sensory neurogenesis waves.
Figure S10. Bifurcation analysis between sympathoadrenal progenitors and enteric glia.
Figure S11. Bifurcation analysis between immature Schwann cells and enteric glia.
Figure S12. Ascl1- and Phox2b-traced cells are enriched in enteric and sympathoadrenal locations while Nr4a2 is enriched in motor and sensory nerves but is dispensable for myelinating and terminal Schwann cells.
Figure S13. Representation of peripheral glia heterogeneity coinciding with positional code and maturation dynamics.
Figure S14. Comparison of the neural crest and Schwann cell lineage transcriptomes to neurofibroma, melanoma and neuroblastoma.
Figure S15. Overview of joint and individual data sets from melanoma and neuroblastoma single cell transcriptomes.

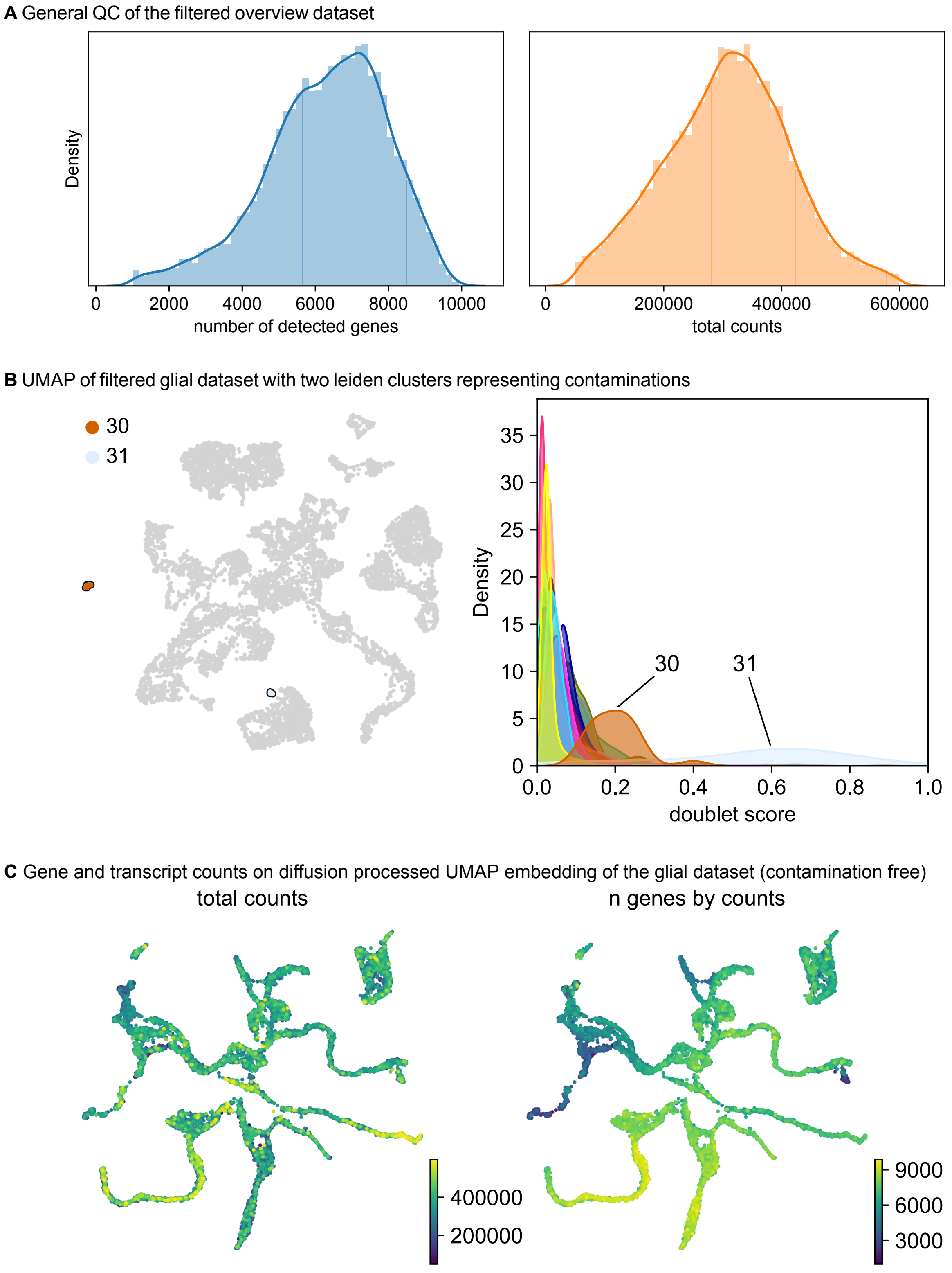

Figure S1. Technical overview of the data set. A) Density plot showing the number of detected genes per cell (left) and total number of transcripts per cell (right). Data shown here is after QC filtering and before filtering of non glial/neuronal cells. **B)** UMAP of filtered glial dataset, with two contaminating clusters having high doublet scores (analysis using scrublet python package). **C)** Same information described in A), this time projected as a colour on the contamination-free glial dataset.

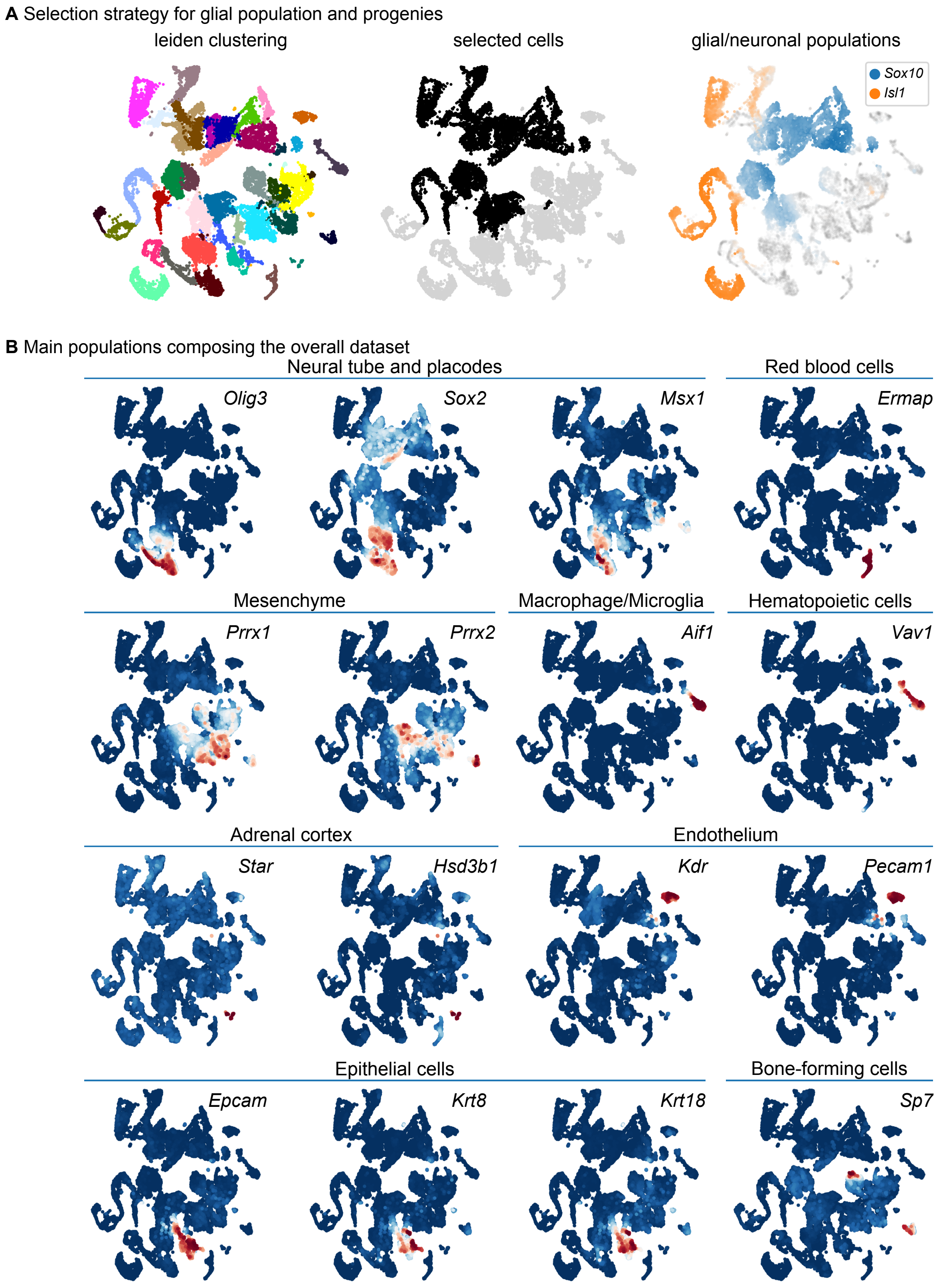

Figure S2. Overview of total data set consisting of neural crest cells and downstream lineages. A) UMAP embedding of the QC filtered dataset, generated from PCA space produced by pagoda2 pipeline. Colored by leiden clustering (left), clusters selected for the rest of the study (middle), based on the expression of pan-neural crest and peripheral glia marker *Sox10* and the neuronal marker *Isl1*. **B)** Characterization of the various cell types and lineages of the QC filtered dataset, with known markers specific to given cell types (Simoes-Costa and Bronner 2015, Tabula Muris, Overall et al. 2018).

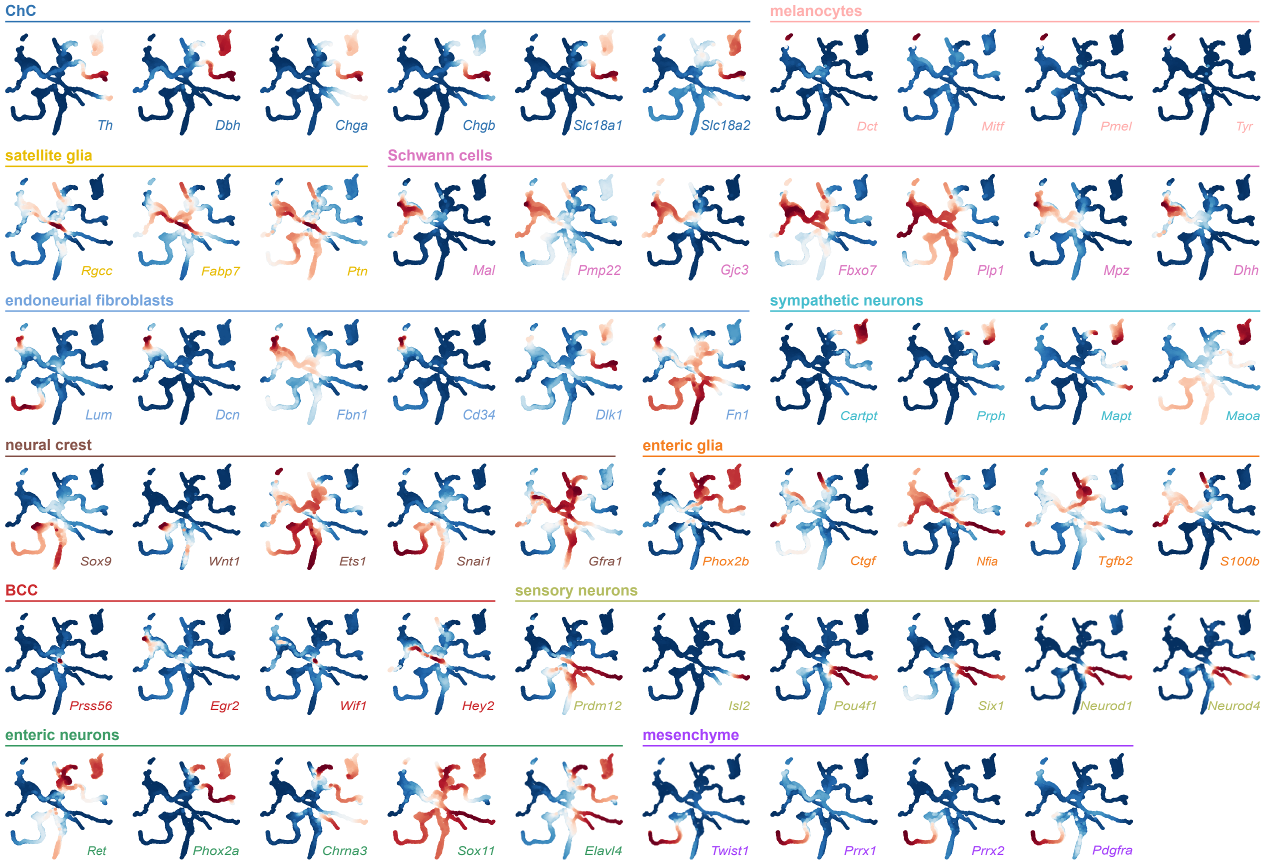

Figure S3. UMAPs of known markers used for cell type annotation. Color-code presented as in Figure 1. (Wu, Saint-Jeannet et al. 2003, Yanfeng, Saint-Jeannet et al. 2003, Bronner and Simoes-Costa 2016, Altevogt, Kleopa et al. 2002, Jessen and Mirsky 2005, Steingrimsson, Copeland et al. 2004, Steingrimsson, Copeland et al. 2004, Furlan, Dyachuk et al. 2017, Kastriti, Kameneva et al. 2019, Richard, Vedrenne et al. 2014, Carr, Toma et al. 2019, Coulpier, Le Crom et al. 2009)

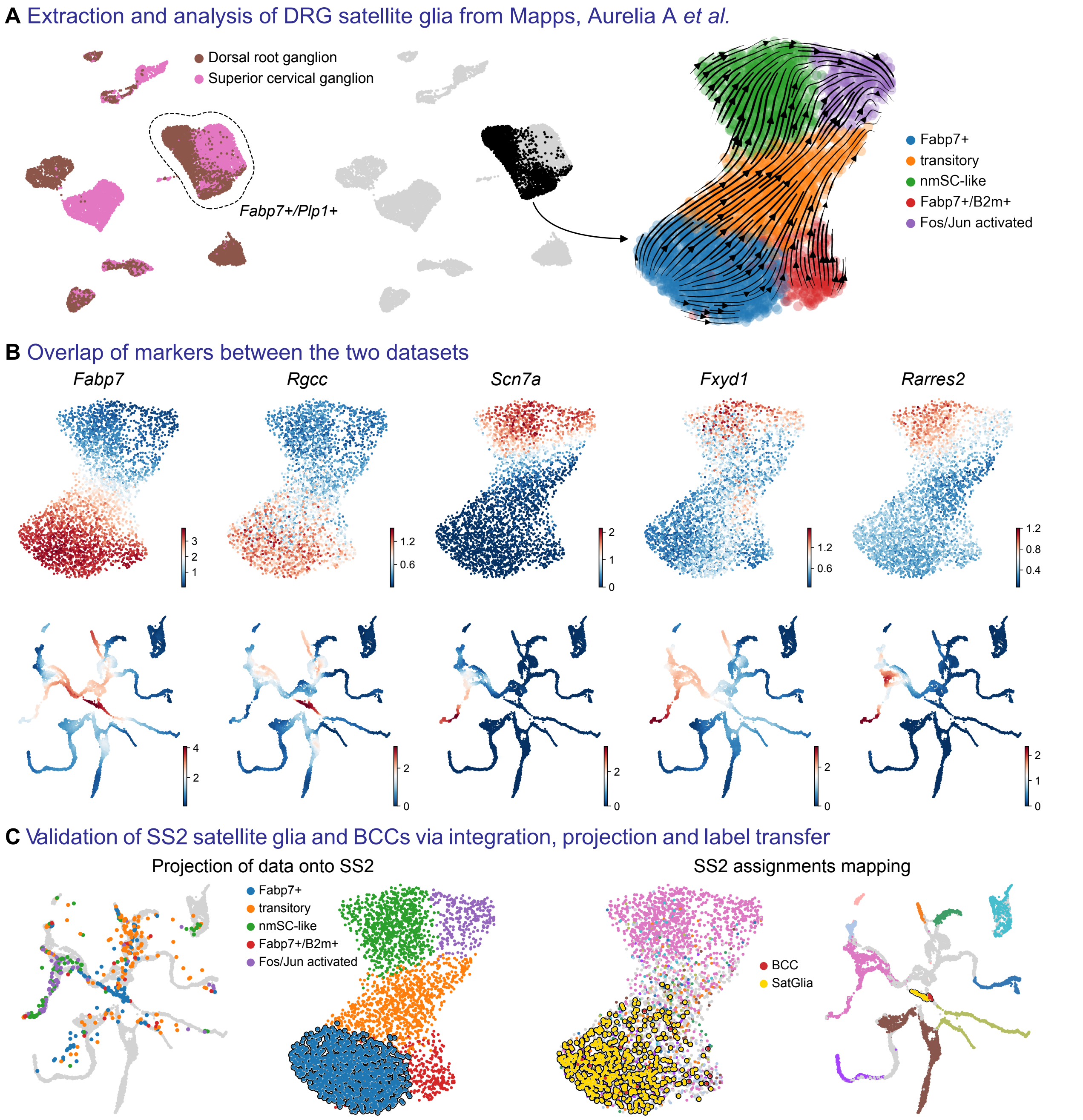

Figure S4. Mapping the signature of satellite glia and boundary cap cells (BCCs) on that of the neural crest and Schwann cell lineage. A) Re-analysis of single cell data set from Mapps, Aurelia A et al. with identified *Plp1*+/*Fabp7*+ satellite glia cluster (left UMAP), selection of dorsal root ganglia-only satellite glia (back cells in centre plot), followed by clustering and RNA velocity analysis. **B)** Overlap of markers of non-myelinating Schwann cells and satellite glia on embeddings of both data sets. **C)** Application of CONOS between satellite glia/BCCs and embryonic data sets, with projection onto embryonic UMAP via knn regression (left), and label transfer from embryonic annotations (right).

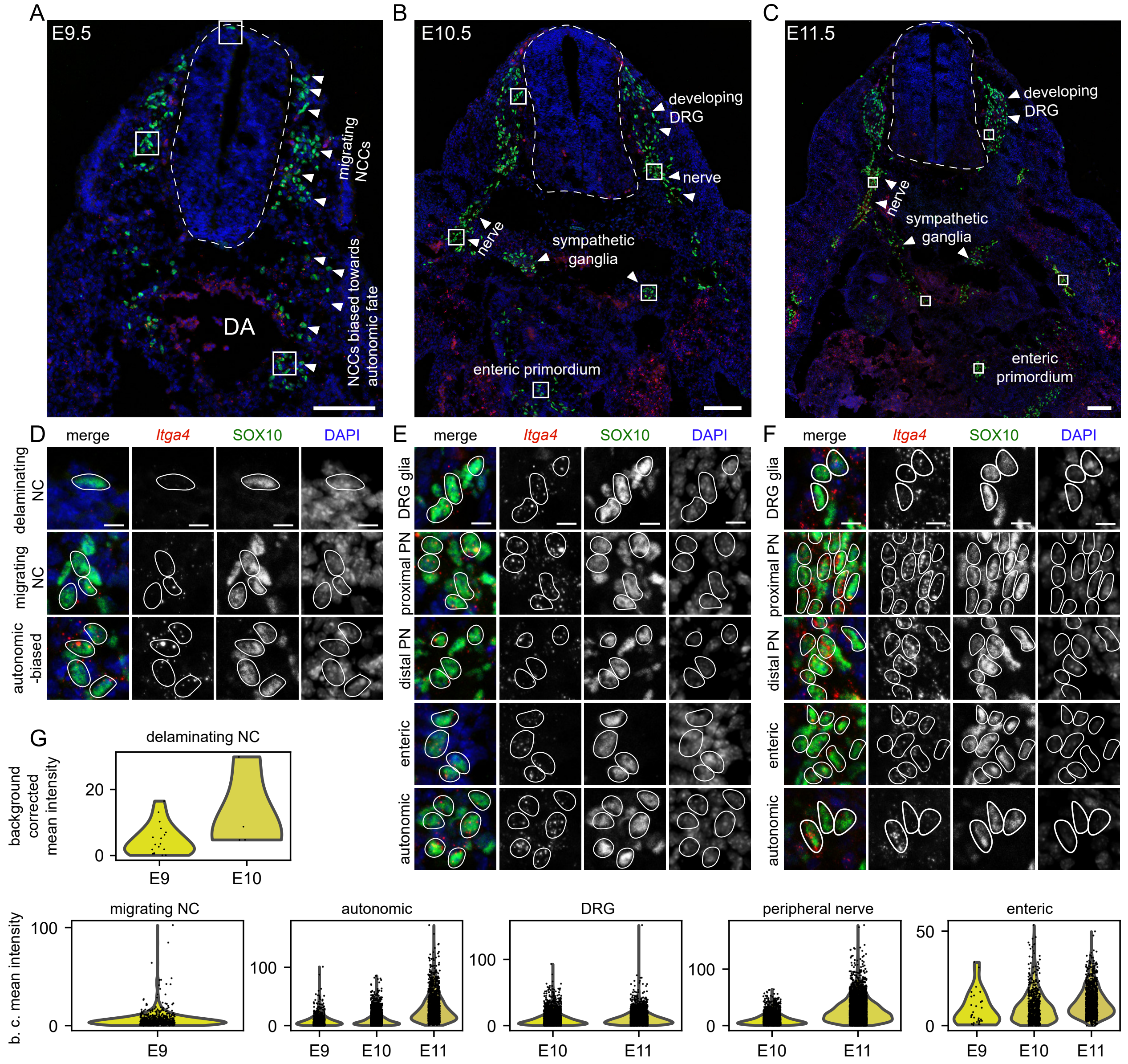

Figure S5. Mapping the expression of the hub marker *Itga4* in neural crest and post-neural crest stages. A-C) Overview of hub cells at different developmental stages from (A) E9.5, (B) E10.5 and (C) E11.5 using SOX10 as a pan-hub marker and RNAscope® in situ hybridization for *Itga4*. Scale bar = 100 µm. **D-F)** Insets of specific locations of hub cells at same developmental stages according to A-C, respectively. N(E9.5)= 2 embryos, 1280 cells, N(E10.5)= 2 embryos, 10163 cells, N(11.5)= 1 embryo, 8427 cells. Scale bar = 10 µm. **G)** Background corrected mean intensity of mRNA signal of *Itga4*, validation by xy method using SOX10 as a pan-hub marker. Stainings replicated in two different instances and with embryos from two different litters.

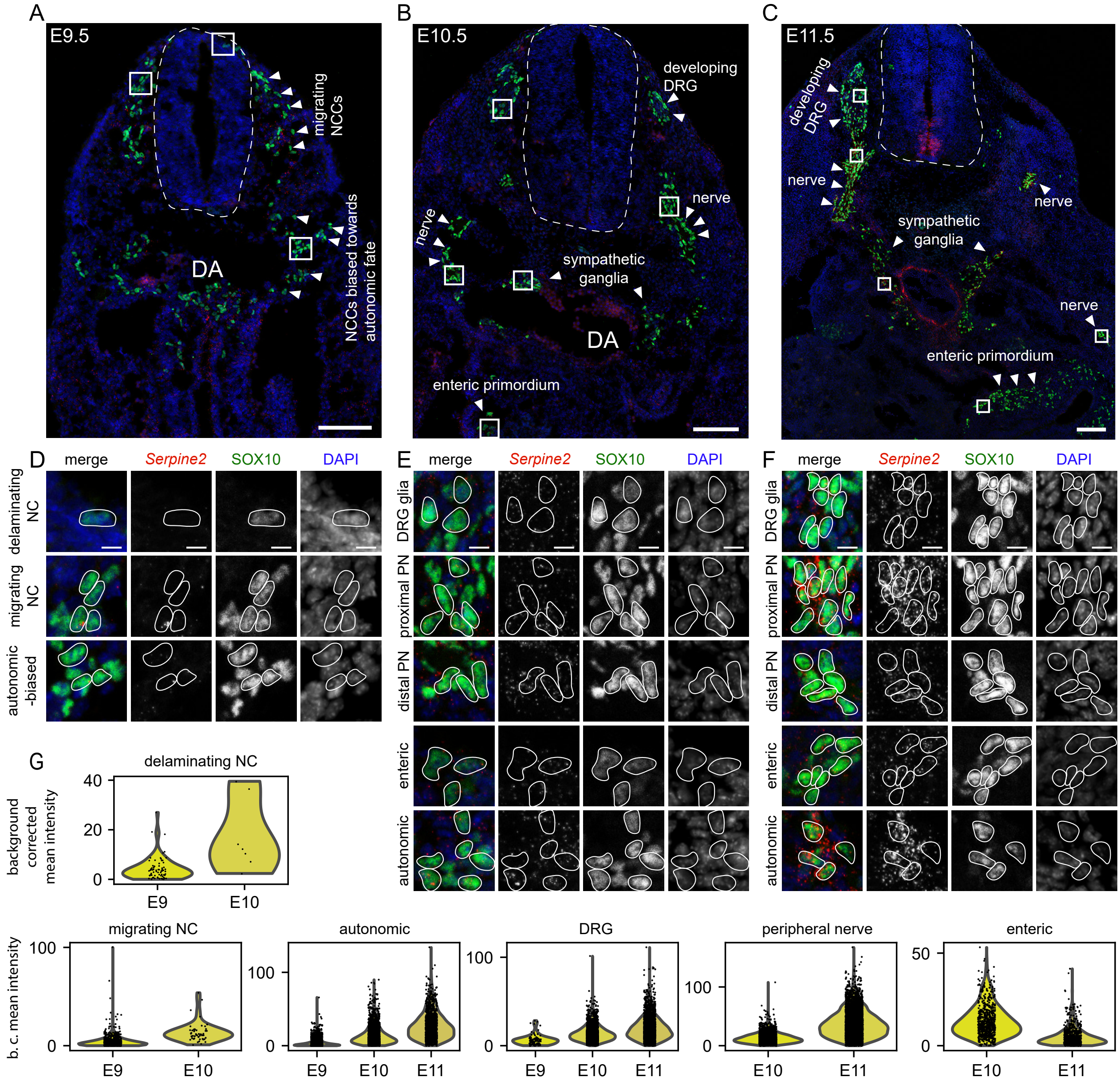

Figure S6. Mapping the expression of the hub marker *Serpine2* in neural crest and post-neural crest stages. A-C) Overview of hub cells at different developmental stages from (A) E9.5, (B) E10.5 and (C) E11.5 using SOX10 as a pan-hub marker and RNAscope® in situ hybridization for *Serpine2*. Scale bar = 100 µm. **D-F)** Insets of specific locations of hub cells at same developmental stages according to A-C, respectively. N(E9.5)= 2 embryos, 3130 cells, N(E10.5)= 2 embryos, 7952 cells, N(11.5)= 1 embryos, 10343 cells. Scale bar = 10 µm. **G)** Background corrected mean intensity of mRNA signal of *Serpine2*, validation by xy method using SOX10 as a pan-hub marker. Stainings replicated in two different instances and with embryos from two different litters.

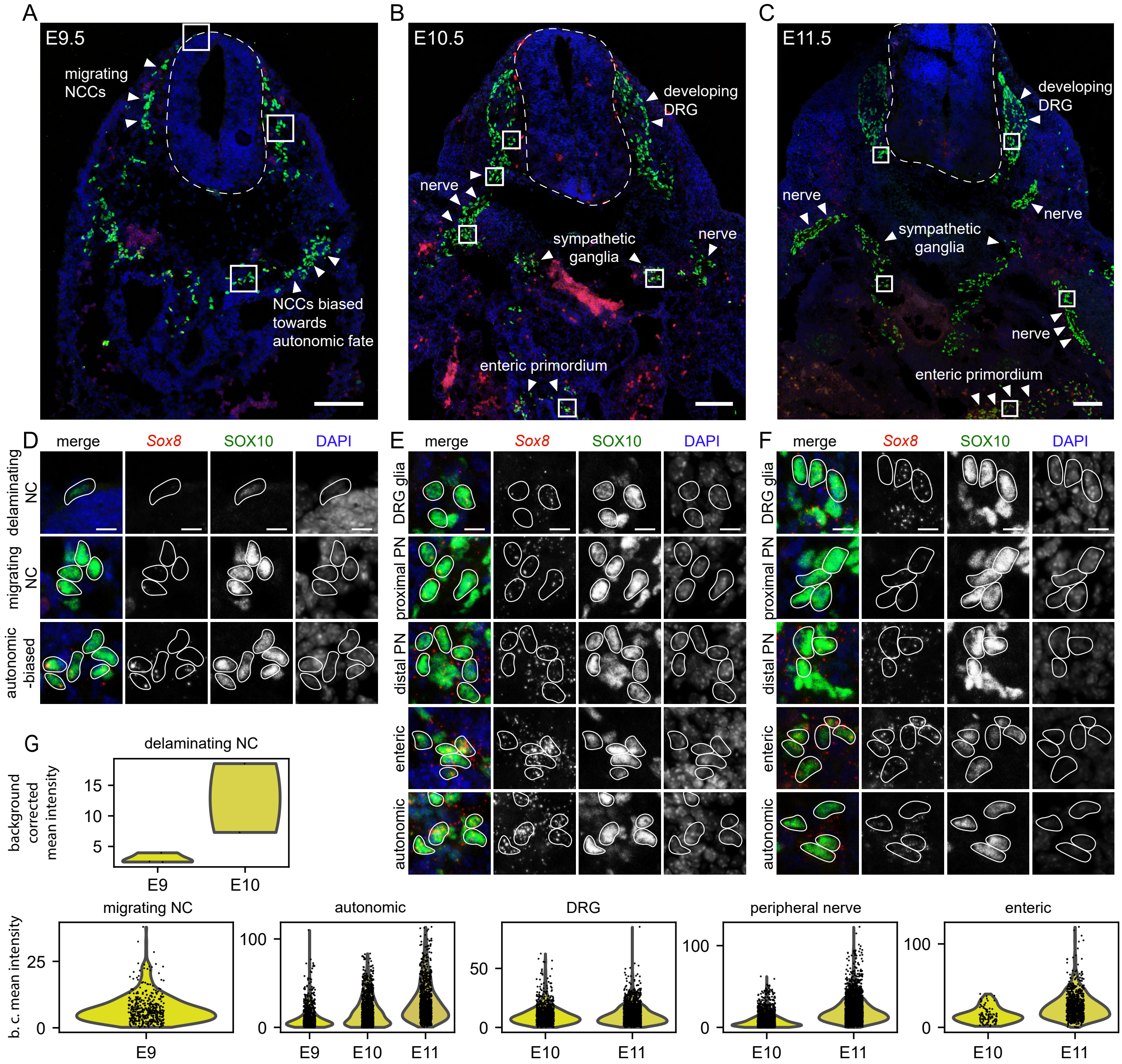

Figure S7. Mapping the expression of the hub marker *Sox8* in neural crest and post-neural crest stages. A-C) Overview of hub cells at different developmental stages from (A) E9.5, (B) E10.5 and (C) E11.5 using SOX10 as a pan-hub marker and RNAscope® in situ hybridization for *Sox8*. Scale bar = 100 µm. **D-F)** Insets of specific locations of hub cells at same developmental stages according to A-C, respectively. N(E9.5)= 1 embryo, 1415 cells, N(E10.5)= 1 embryo, 5953 cells, N(11.5)= 1 embryo, 8107 cells. Scale bar = 10 µm. **G)** Background corrected mean intensity of mRNA signal of *Sox8*, validation by xy method using SOX10 as a pan-hub marker. Stainings replicated in two different instances and with embryos from two different litters.

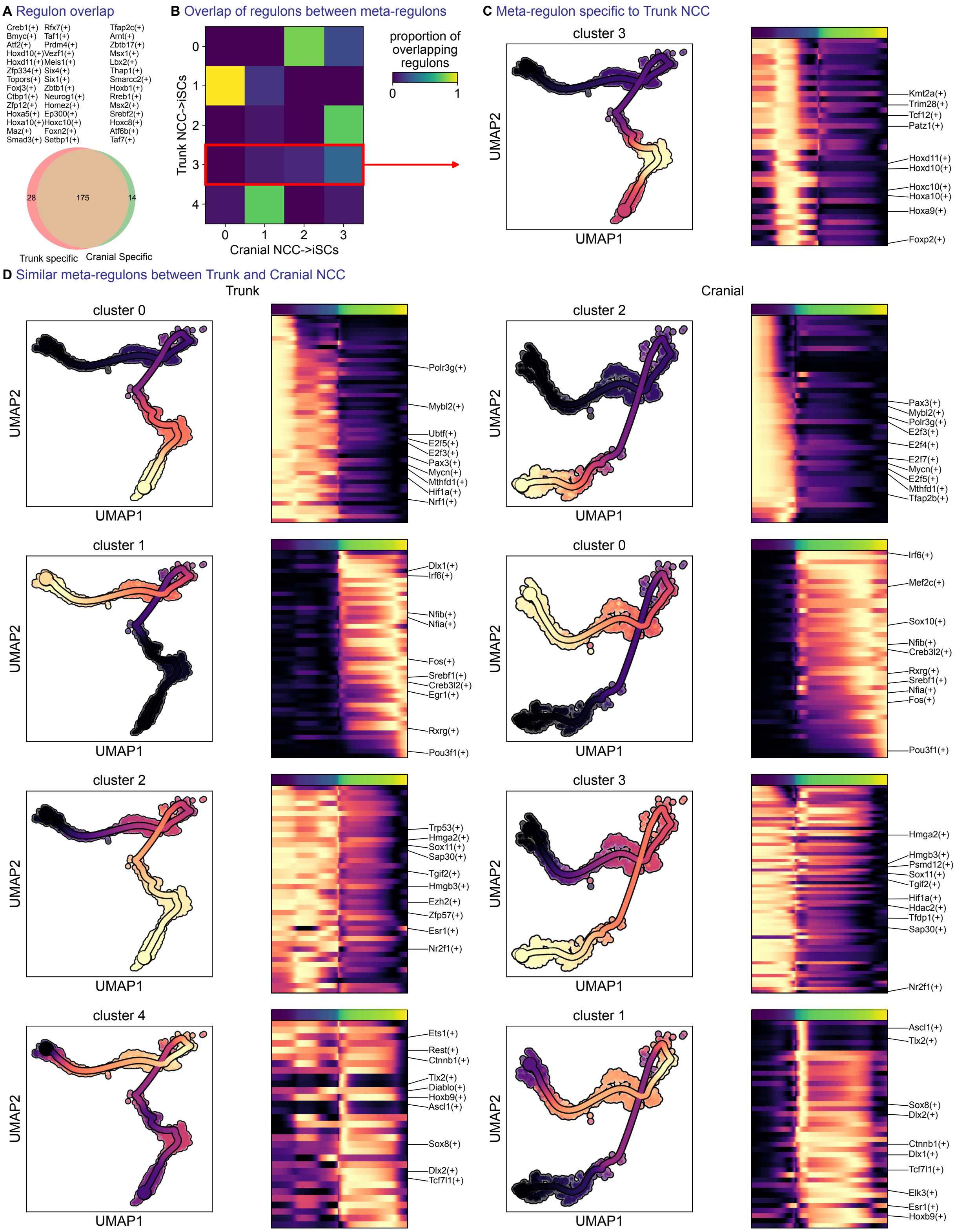

Figure S8. Analysis of regulon activity from cranial or trunk neural crest subpopulations to immature Schwann cells. A) Venn diagram of regulons changing significantly along the trajectory between trunk neural crest cells to immature Schwann cells (left, red) and cranial neural crest cells to immature Schwann cells (right, green) and their overlap shown in orange. Regulons unique to either trajectory shown as lists (trunk: left, cranial: right). **B)** Overlap of regulons between the two trajectories from either cranial or trunk neural crest to immature Schwann cells. Highlighted in red a trunk trajectory cluster/metaregulon (cluster 3) showing the lowest overlap with those active in the cranial trajectory. **C)** Metaregulon specific to the trunk trajectory and heatmap of the activation dynamics (right). **D)** Pairs of metaregulons from both trajectories showing the highest overlap (similar metaregulons shown in each row, left column: trunk, right column: cranial).

A First sensory bifurcation

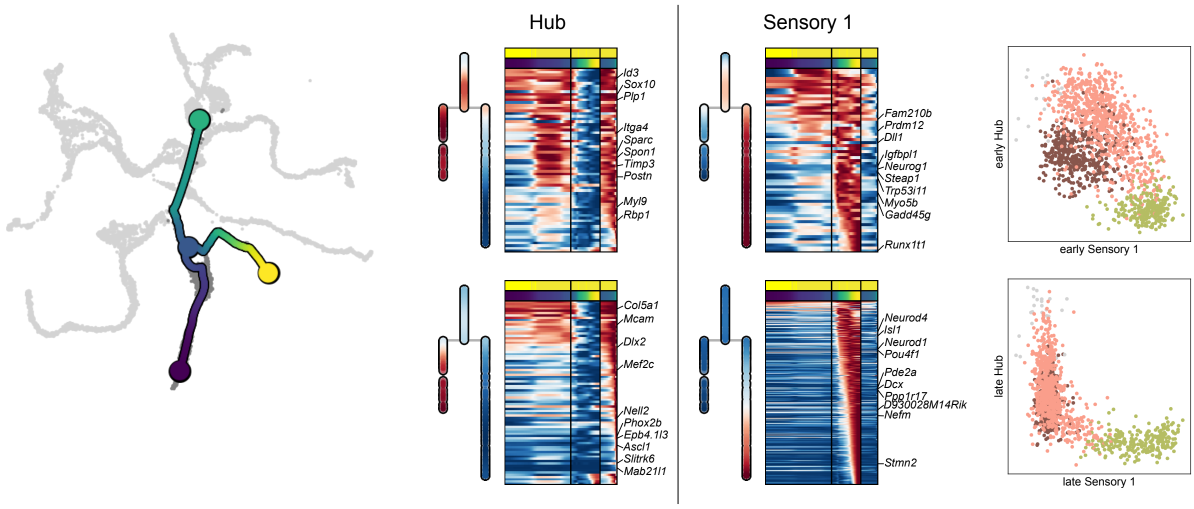

B Second sensory bifurcation

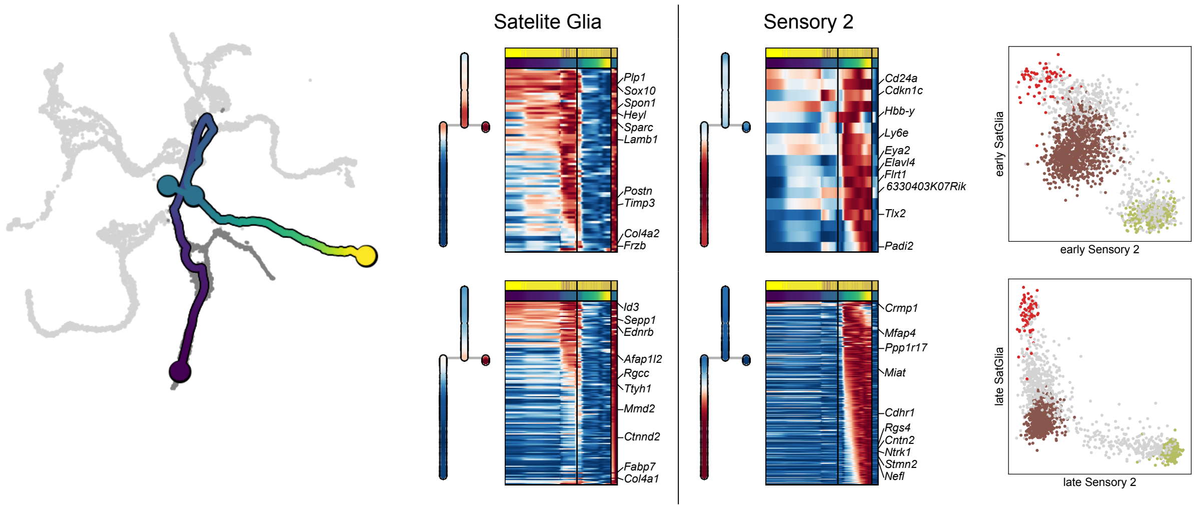

Figure S9. Detailed bifurcation analyses of the sensory neurogenesis waves. A) Focus on the first sensory bifurcation versus hub, showing early and late modules as heatmaps (center) and mean gene expression scatter plots (right), and early and late genes. **B)** Focus on the second sensory bifurcation versus hub, showing early and late modules as heatmaps (center) and mean gene expression scatter plots (right), and early and late genes.

1

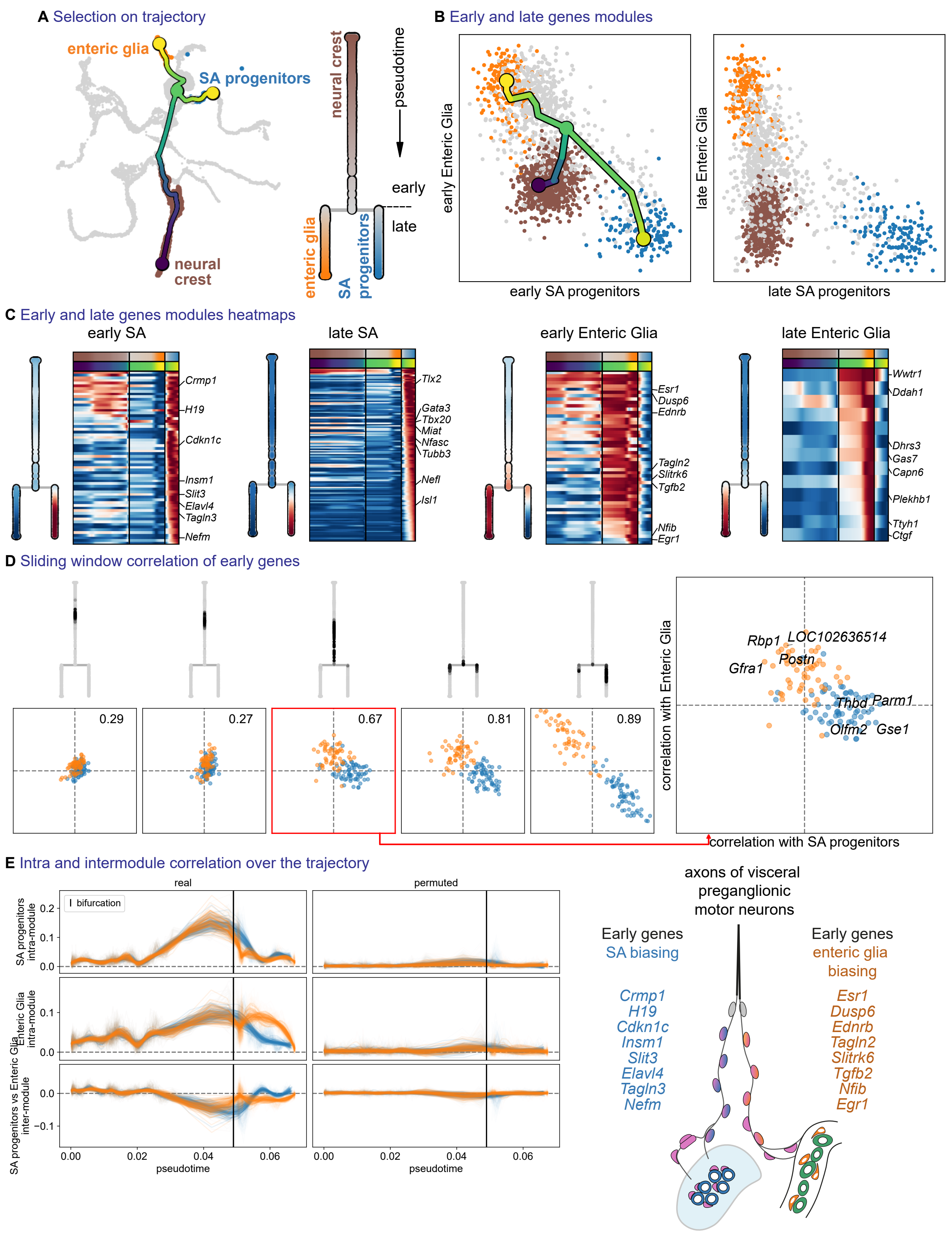

Figure S10. Bifurcation analysis between sympathoadrenal progenitors and enteric glia. A) Subset of the tree containing the trajectory containing the bifurcation between sympatho-adrenal progenitors and enteric glia. An abstract dendrogram is displayed on the right. **B)** Mean expression of detected early and gene module for both branches, with the tree overlaid on the early gene modules. **C)** Heatmap of early and late gene modules of both branches, with mean expression shown on the dendrogram representations. **D)** Inter/intra correlation analysis of early gene modules of both branches, on non-intersecting windows of cells over the trajectory. **E)** Left: Inter/intra correlation trends of early gene modules of both branches, on sliding window of cells over 100 probabilistic mappings of the trajectory. Right: results with local permutation of expression.

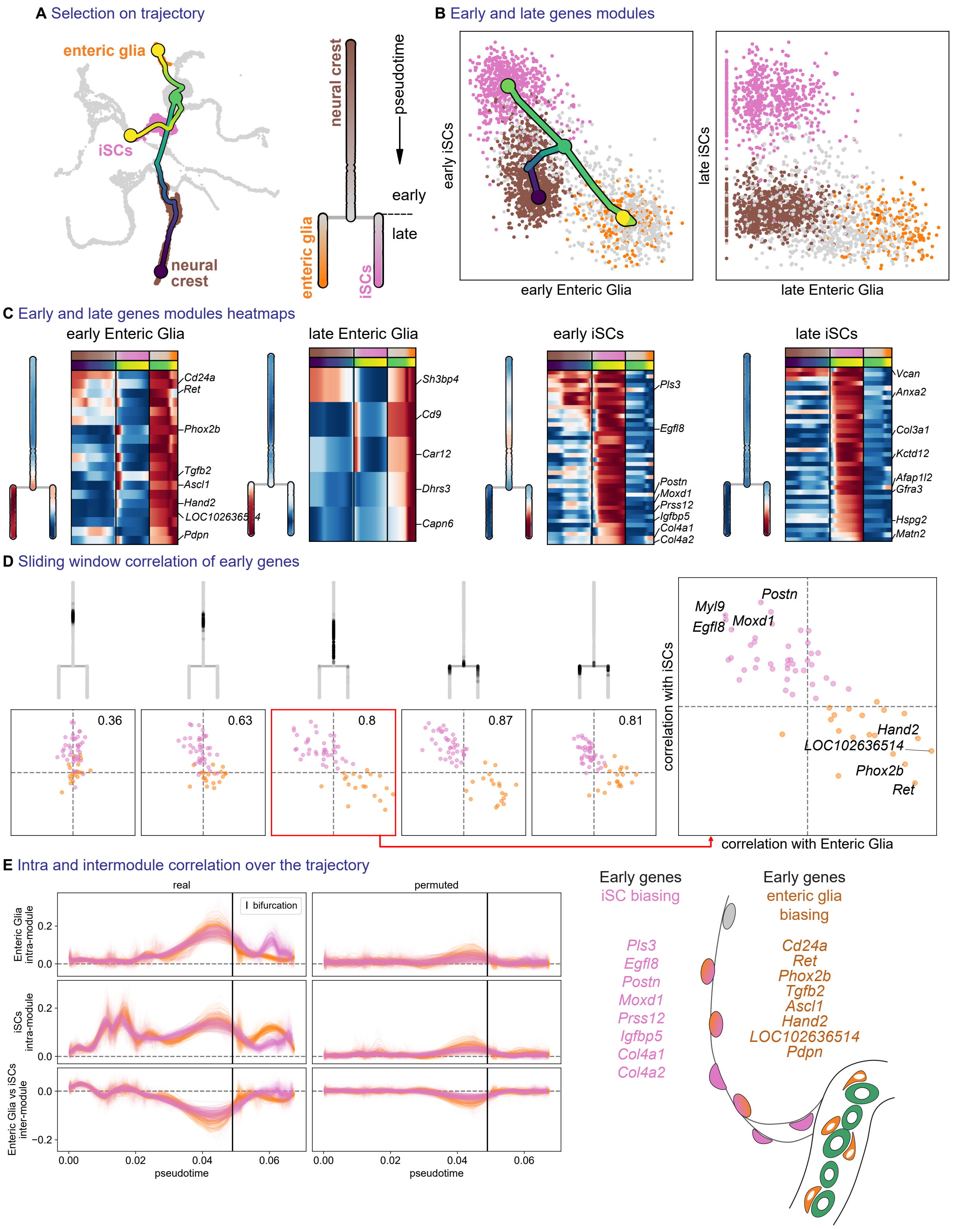

Figure S11. Bifurcation analysis between immature Schwann cells and enteric glia. A) Subset of the tree containing the trajectory containing the bifurcation between immature Schwann cells (iSCs) and enteric glia. An abstract dendrogram is displayed on the right. **B)** Mean expression of detected early and gene module for both branches, with the tree overlaid on the early gene modules. **C)** Heatmap of early and late gene modules of both branches, with mean expression shown on the dendrogram representations. **D)** Inter/intra correlation analysis of early gene modules of both branches, on non-intersecting windows of cells over the trajectory. **E)** Left: Inter/intra correlation trends of early gene modules of both branches, on sliding window of cells over 100 probabilistic mappings of the trajectory. Right: results with local permutation of expression.

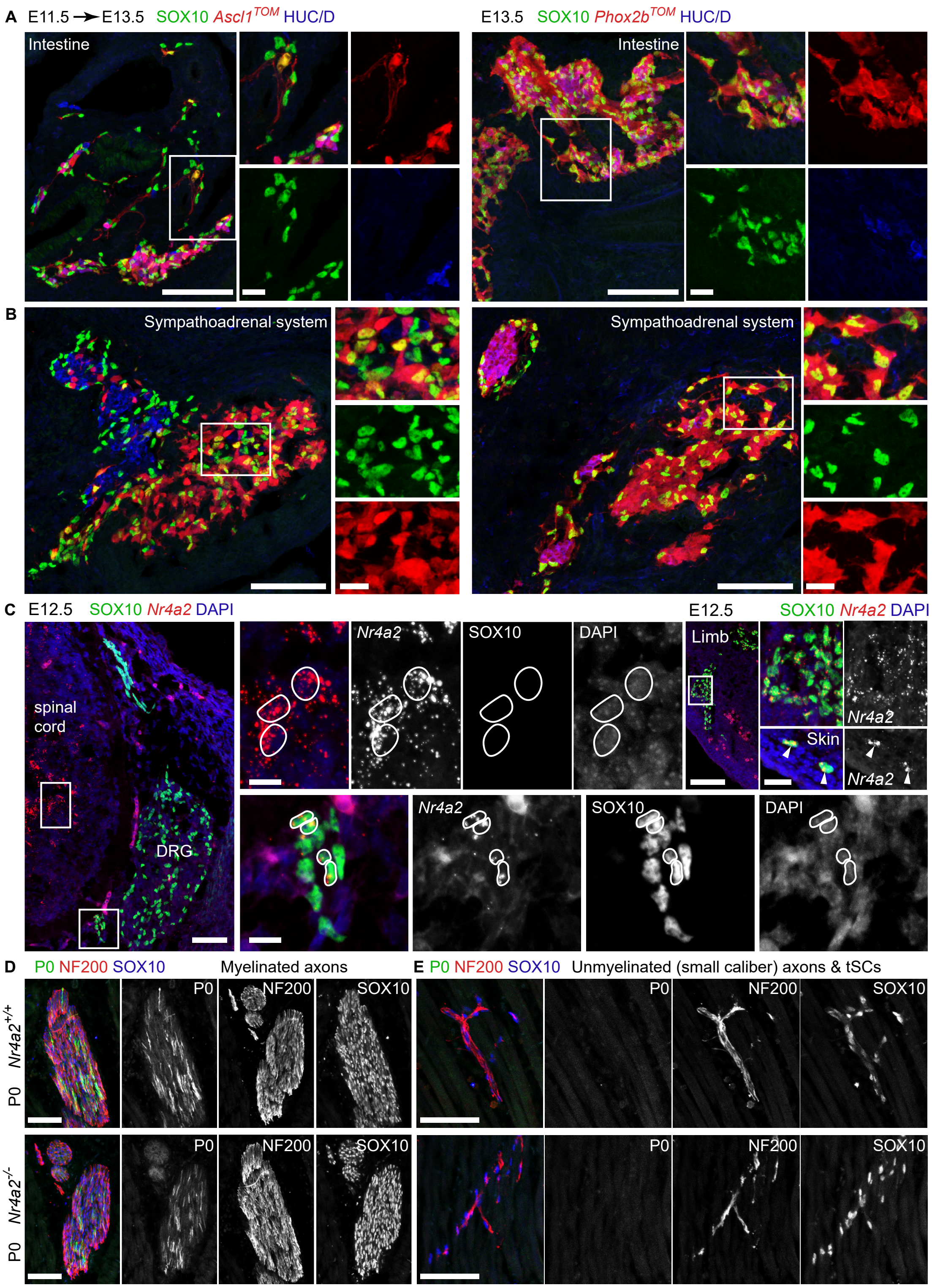

Figure S12. ***Ascl1*- and *Phox2b*-traced cells are enriched in enteric and sympathoadrenal locations while *Nr4a2* is enriched in motor and sensory nerves but is dispensable for myelinating and terminal Schwann cells. A-B)** Immunofluorescent staining against SOX10, *Ascl1*^{TOM} or *Phox2b*^{TOM} (lineage tracing of au-tonomic and enteric glia) and HUC/D (neuronal marker) on E13.5 trunk in the intestine and sympathoadrenal system, respectively. Scale bar in overviews is 100 µm and 20 µm in insets. **C)** Immunofluorescent staining against SOX10 combined with RNAscope for *Nr4a2* on E12.5 trunk (left) and limb (right). Scale bar in overviews is 100 µm and 20 µm in insets. **D)** Immunofluorescent staining against P0 (myelin marker), NF200 (axonal staining) and SOX10 (a marker of all peripheral glia) on newborn (Postnatal day 0=P0) of *Nr4a2* wild type (Nr4a2$^{+/+}$) and knock out (*Nr4a2*$^{-/-}$) pups showing myelinated and non-myelinated axons with associated terminal Schwann cells. Scale bar = 100 µm.

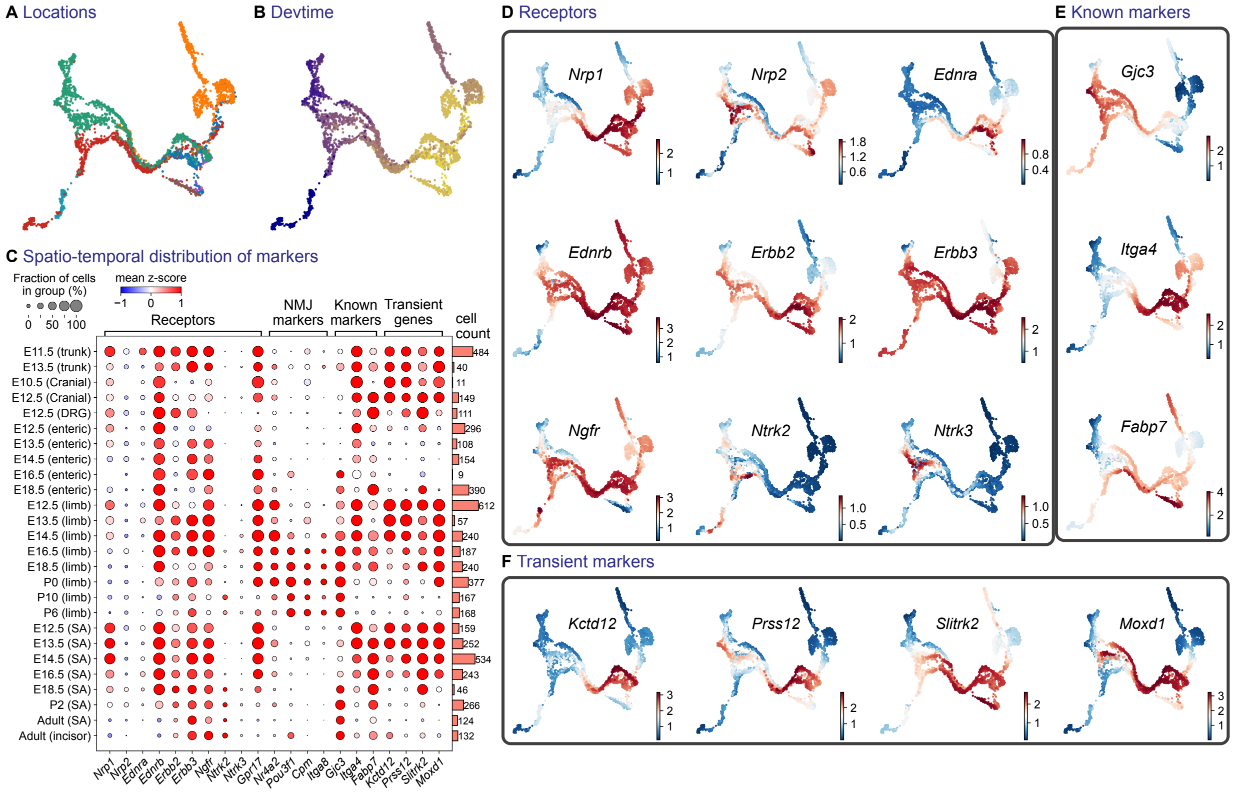

Figure S13. Representation of peripheral glia heterogeneity coinciding with positional code and maturation dynamics. A) UMAP representation of sampled locations of sampled single Schwann cells. **B)** UMAP representation of developmental stage of sampled single Schwann cells. **C)** Dot plot showing expression of known markers of Schwann cells as well as unbiased novel markers from our analysis with a location-based enrichment. **D)** UMAP showing expression of receptors. **E)** UMAP showing expression of known Schwann cell markers. **F)** UMAP showing expression of transient markers uncovered by our analysis.

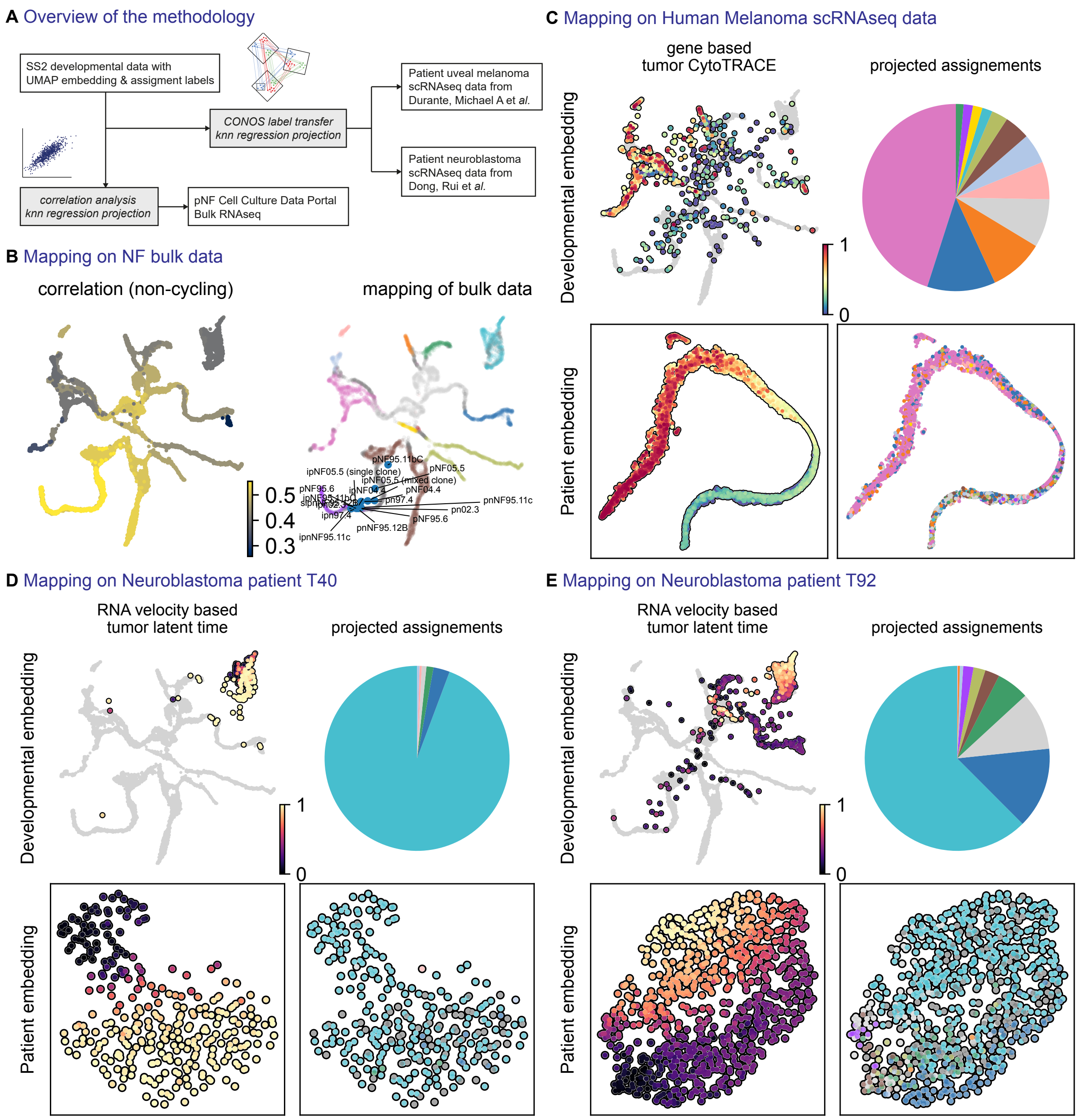

Figure S14. Comparison of the neural crest and Schwann cell lineage transcriptomes to neurofibroma, melanoma and neuroblastoma. A) Methodology of cancer cell mapping onto our developmental data, using CONOS for single cell and correlation analysis for bulk dataset. Knn regression was employed for projection onto embryonic UMAP. **B)** Mapping of neurofibroma bulk data with correlation of bulk transcriptomes with embryonic leiden clusters (left) and projection onto developmental UMAP (right). **C)** UMAP embedding of the embryonic dataset (grey points) with combined mapping of cells from human melanoma (single cell) transcriptomic data, colored with tumor CytoTRACE value (top left). Proportions of projected annotations to the tumor data (top right). Same representation follows as tumor gene based tSNE (lower part). **D and E)** UMAP embedding of the embryonic dataset (grey points) with single cell transcriptomes from two neuroblastoma patients, colored with tumor RNA velocity based latent time (top left). Proportions of projected annotations to the tumor data (top right). Similar representation on the tumor gene based UMAP (lower part).

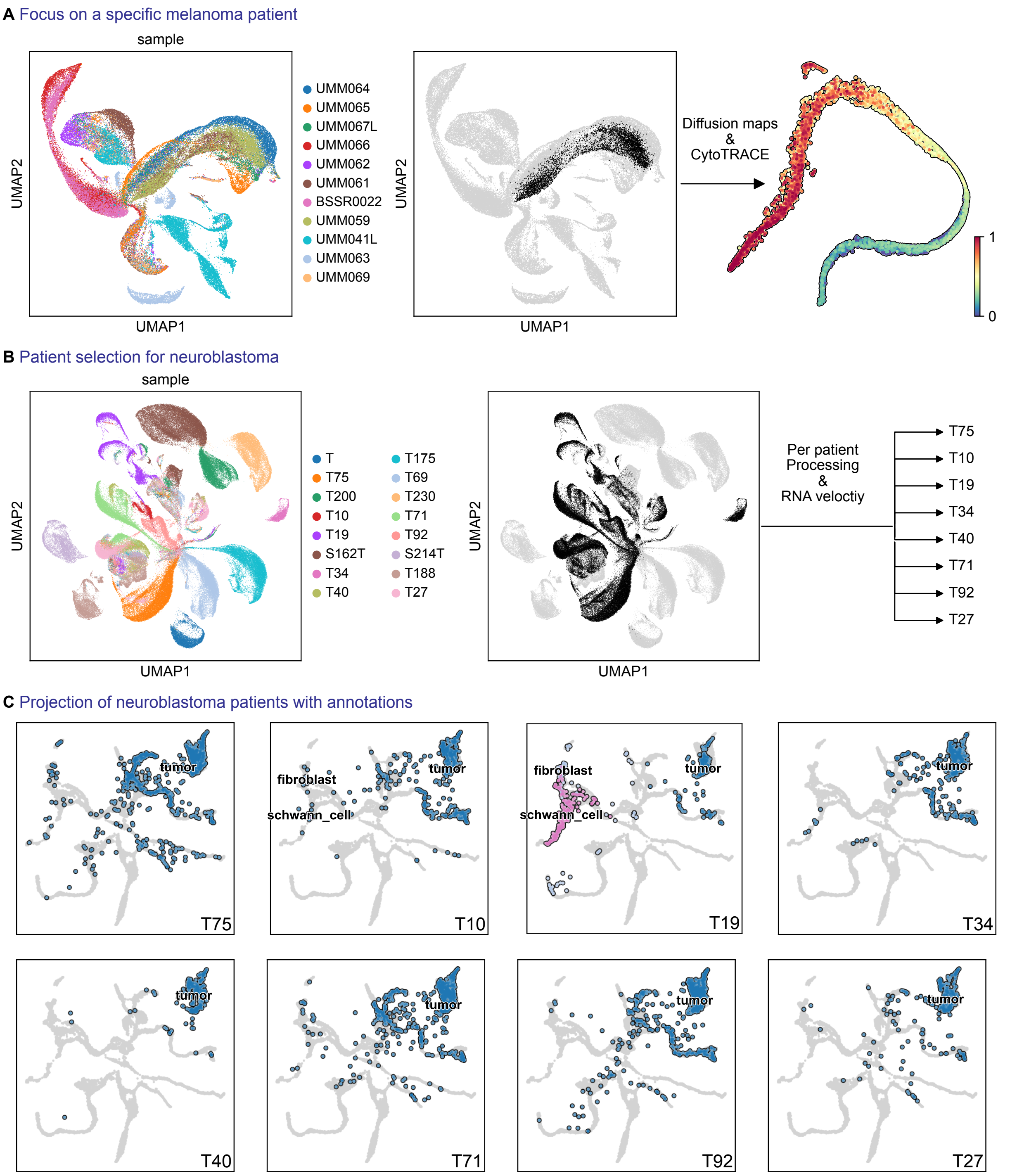

Figure S15. Overview of joint and individual data sets from melanoma and neuroblastoma single cell transcriptomes. A) UMAP of the combined melanoma dataset colored by patient (left), data anal-ysis from a single melanoma patient (middle) and application of CytoTRACE, shown on diffusion map-based UMAP (right). **B)** UMAP of the combined neuroblastoma dataset colored by patient (left), selection of patients (right) for per patient processing and RNA velocity analysis. **C)** Projection of individual neuroblastoma patients onto our developmental dataset, coloring cancer cells with published annotations.
