# Supplementary material for: Schwann cell precursors represent a neural crest‐like state with biased multipotency
Source: EMBO J. 2022 Jul 11;41(17):e108780. doi: 10.15252/embj.2021108780 (PMC9434083; doi:10.15252/embj.2021108780)
Supplement: Supplementary file 2 — Expanded View Figures PDF [file EMBJ-41-e108780-s003.pdf]

Expanded View Figures

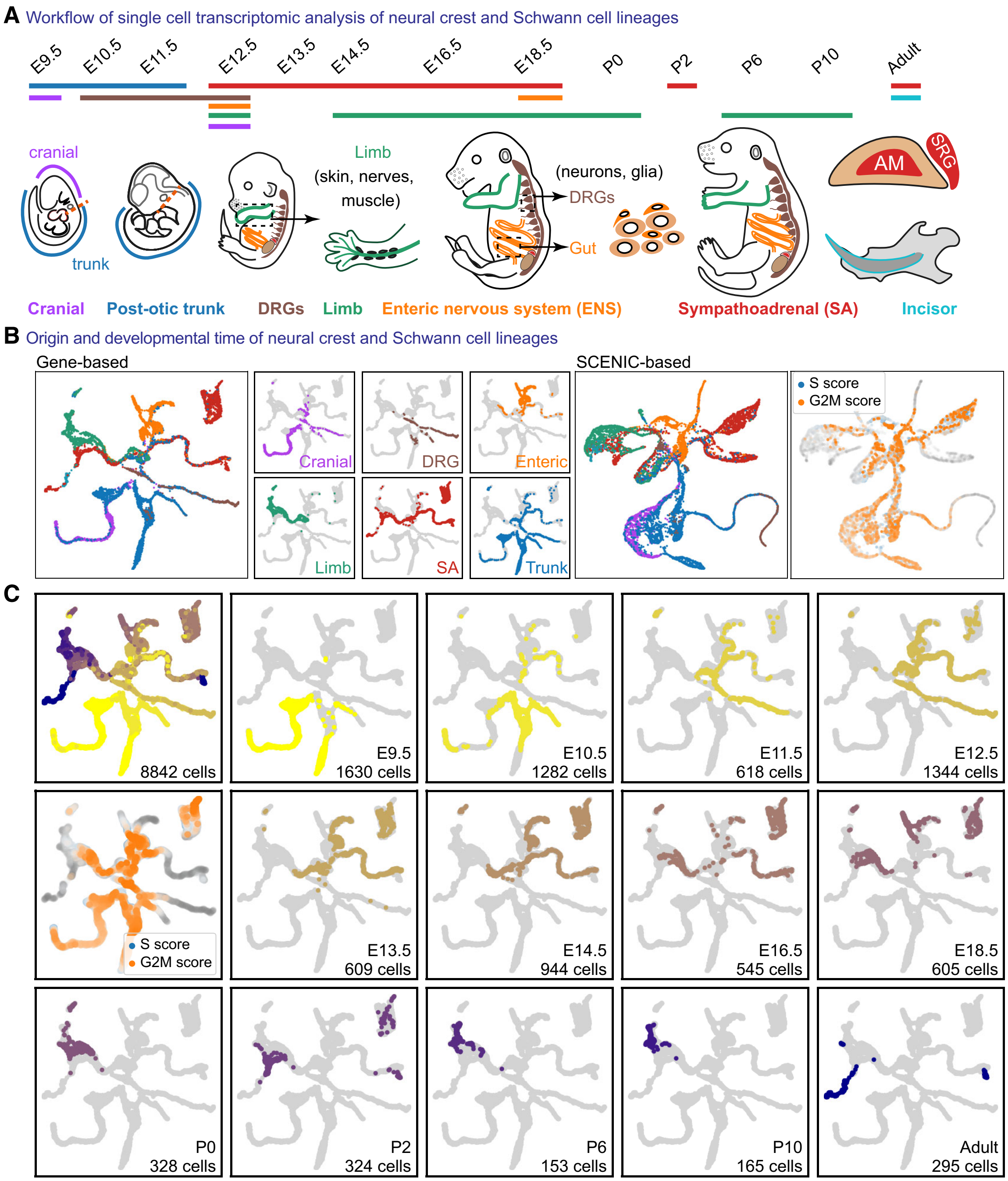

Figure EV1. Overview and composition of neural crest and Schwann cell data set.

A Overview of the sampled locations and time points in mice. AM, adrenal medulla; SRG, suprarenal ganglion.

B Colour-coded projections of the tissue of origin, including SCENIC regulon-based UMAP embedding, with cell cycle shown (right).

C UMAP embeddings colour-coded according to the developmental stage, with cell cycle shown.

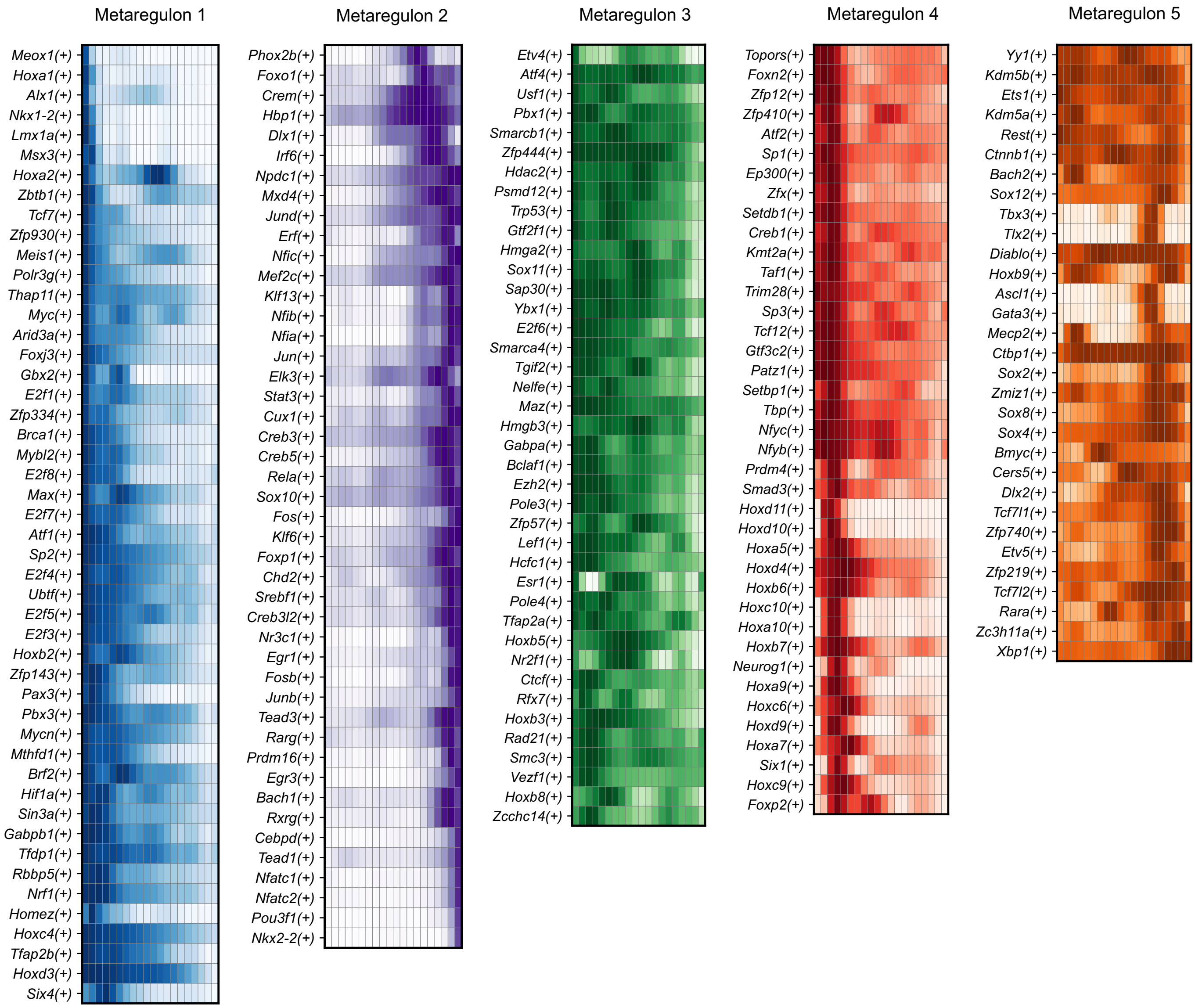

Figure EV2. Metaregulon composition.

Heatmaps of regulon activity (AUC scores) of individual regulons making up metaregulons 1–5, over the neural crest to immature Schwann cell trajectory, summarised into 20 pseudotime bins.

©2022 The Authors

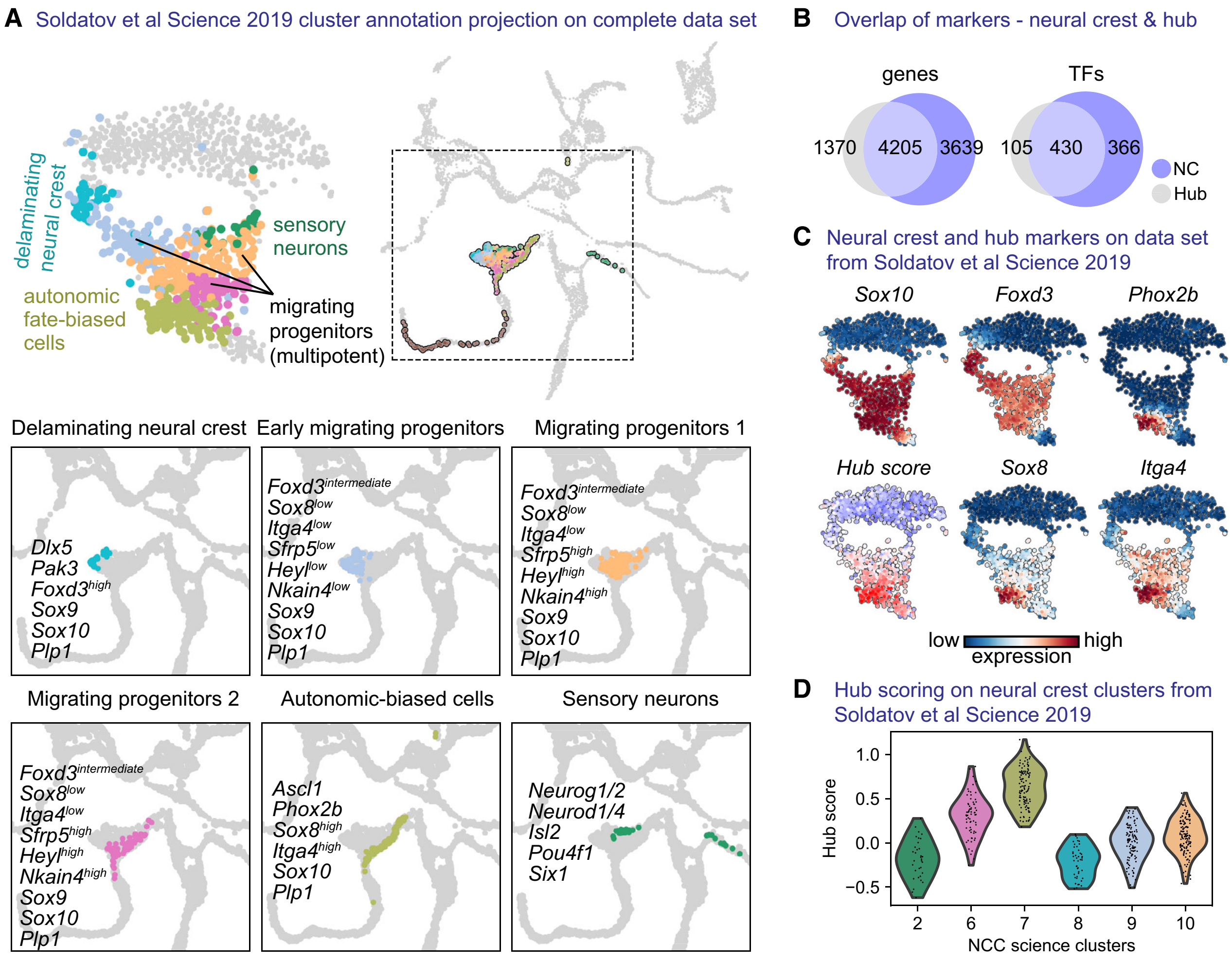

Figure EV3. Mapping and comparison of the "hub" state on previously published single-cell data set of the neural crest.

A tSNE embedding and annotated clusters from Soldatov *et al* (2019), overlayed onto the UMAP embedding containing our own data set.

B Venn diagram showing genes and transcription factors (TFs) positively regulated when comparing, respectively, neural crest cells and "hub" cells to the rest of the cells of the data set (Wilcoxon rank-sum test).

C "hub" scoring on previous data set using gene scoring from the top 25 differentially expressed genes specific to the "hub."

D Violin plot of "hub" score over the published annotated clusters.

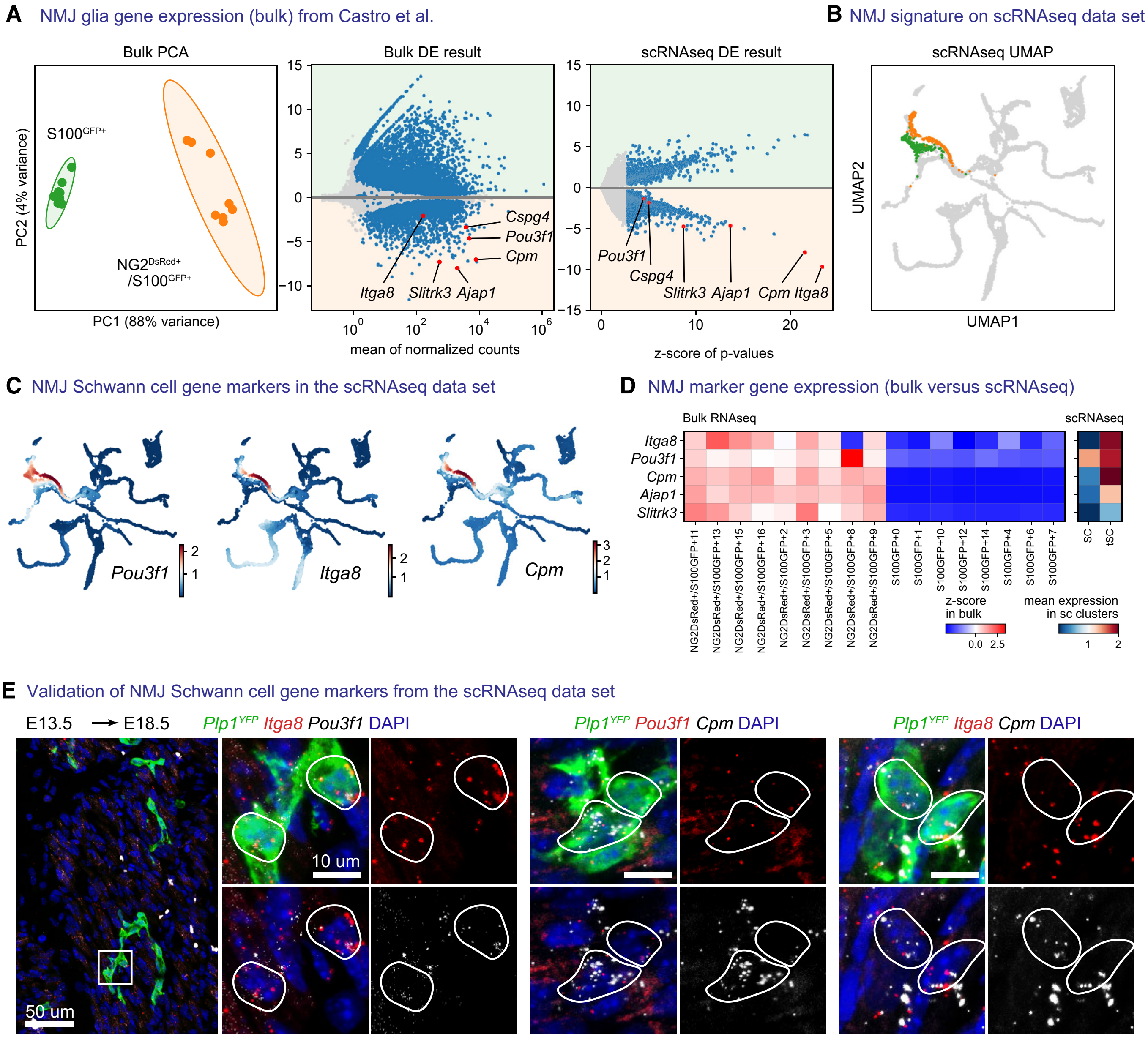

Figure EV4. Late embryonic and early postnatal terminal glia representation in the data set.

A Re-analysis of bulk RNA sequencing data of S100^{GFP+} cells and NG2^{dsRED+}/S100^{GFP+} cells isolated from adult neuromuscular junctions from Castro *et al* (2020) with PCA plot (left) or DESeq2 differential gene expression analysis (center) and comparison of differential expression results (right) from Leiden clusters of terminal Schwann cells and fate-biased Schwann cells in our own data set.

B Leiden clusters defining terminal Schwann cells (orange) and fate-biased Schwann cells (Green *et al*, 2017).

C MAGIC-imputed expression of *Pou3f1*, *Itga8* and *Cpm* on UMAP embeddings.

D Comparison of five terminal Schwann cells markers, as z-scores on bulk data per sample (left) and as mean expression in the Leiden clusters shown in (A and B) in our own data set (right).

E RNAscope *in situ* hybridization validation of *Pou3f1*, *Itga8* and *Cpm* as markers of terminal Schwann cells at E18.5 combined with immunofluorescence against *Plp1*^{YFP} on hindlimbs of an embryo injected with tamoxifen at E13.5. Stainings were repeated on two separate occasions on multiple embryos from the same litter.

©2022 The Authors
