# Supplementary material for: Schwann cell precursors represent a neural crest‐like state with biased multipotency
Source: EMBO J. 2022 Jul 11;41(17):e108780. doi: 10.15252/embj.2021108780 (PMC9434083; doi:10.15252/embj.2021108780)
Supplement: Supplementary file 3 — Dataset EV1 [file EMBJ-41-e108780-s001.pdf]

names	class	TF
Hmga2	common	TRUE
Igf2bp1	common	FALSE
Pabpc1	common	FALSE
Hn1l	common	FALSE
Grb10	common	FALSE
Crabp2	common	FALSE
Mybbp1a	common	FALSE
Kpnb1	common	FALSE
Cdca7	common	FALSE
Cbx5	common	FALSE
Cad	common	FALSE
Gas1	common	FALSE
Cse1l	common	FALSE
Sox9	common	TRUE
Set	common	FALSE
Nrip1	common	FALSE
Supt16	common	FALSE
Dkc1	common	FALSE
Pfas	common	FALSE
Trim28	common	TRUE
Ncl	common	FALSE
Arhgef26	common	FALSE
Mtap	common	FALSE
Ptbp1	common	FALSE
Arhgap28	common	FALSE
Pls3	common	FALSE
Rcc1	common	FALSE
Caprin1	common	FALSE
Peg12	common	FALSE
Col2a1	common	FALSE
Eif4g2	common	FALSE
Nap1l1	common	TRUE
Ets1	common	TRUE
Bub1b	common	FALSE
Ssrp1	common	TRUE
Tcof1	common	FALSE
Hspa4	common	FALSE
G3bp1	common	FALSE
Dtx4	common	FALSE
Uhrf1	common	FALSE
Gsta4	common	FALSE
Gli3	common	TRUE
Mcm6	common	FALSE
Nedd9	common	FALSE
Hspd1	common	FALSE
Yap1	common	FALSE
Pa2g4	common	FALSE
Ipo11	common	FALSE
Parp1	common	TRUE

Mcm5	common	FALSE
Lef1	common	TRUE
Npm1	common	FALSE
Dna2	common	FALSE
Ppp2r1b	common	FALSE
Hnrnpu	common	FALSE
Ppat	common	FALSE
Skp2	common	FALSE
Txnrd1	common	FALSE
Wdr77	common	FALSE
Eftud2	common	FALSE
Mcm4	common	FALSE
Ctnnb1	common	TRUE
Cct8	common	FALSE
Abce1	common	FALSE
Sf3b3	common	FALSE
Atad2	common	FALSE
Msh6	common	FALSE
Chaf1a	common	FALSE
Kif21b	common	FALSE
Fbn2	common	FALSE
Eif5a	common	FALSE
Sep-09	common	FALSE
Greb1	common	FALSE
Mcm3	common	FALSE
Cdk18	common	FALSE
Rbbp7	common	FALSE
Mapk1ip1l	common	FALSE
Pgk1	common	FALSE
Nudt4	common	FALSE
Cdh6	common	FALSE
Mcm7	common	FALSE
Ajuba	common	FALSE
Lrig3	common	FALSE
Mcm2	common	FALSE
Ccnd2	common	FALSE
Hsp90ab1	common	FALSE
Ahcy	common	FALSE
Ppa1	common	FALSE
Igf2bp2	common	FALSE
Polr2m	common	FALSE
Naa15	common	FALSE
Snx5	common	FALSE
Eif3b	common	FALSE
Npr3	common	FALSE
Eif3a	common	FALSE
Pfkl	common	FALSE
Erbb2	common	FALSE
Kif20a	common	FALSE
Odc1	common	TRUE

Nes	common	FALSE
Cct3	common	FALSE
Kif15	common	FALSE
Mest	common	FALSE
Notch3	common	FALSE
Serpinf1	common	FALSE
Umps	common	FALSE
Heatr3	common	FALSE
Gja1	common	FALSE
Fignl1	common	FALSE
Bzw1	common	FALSE
Ran	common	TRUE
Nasp	common	FALSE
Wdr43	common	FALSE
Phf6	common	FALSE
Usp10	common	FALSE
Hells	common	FALSE
Slc25a13	common	FALSE
Gmps	common	FALSE
Api5	common	FALSE
Tmpo	common	FALSE
Smo	common	FALSE
Gspt1	common	FALSE
Nop58	common	FALSE
Zfp568	common	TRUE
Serbp1	common	FALSE
Rrm1	common	FALSE
Cand1	common	FALSE
Nol10	common	FALSE
Ncapg	common	FALSE
Ddx39	common	FALSE
Isg20l2	common	FALSE
Ptcd3	common	FALSE
Notch2	common	FALSE
Fen1	common	FALSE
Smad3	common	TRUE
Smyd5	common	FALSE
Cirh1a	common	FALSE
Etf1	common	FALSE
Mdn1	common	FALSE
Ftsj3	common	FALSE
Kars	common	FALSE
Eif4g1	common	FALSE
Cul4b	common	FALSE
Pole	common	FALSE
St5	common	FALSE
Xpot	common	FALSE
Mthfd1l	common	FALSE
Cct4	common	FALSE
Cdca7l	common	FALSE

Nucks1	common	FALSE
Cnbp	common	FALSE
Srsf6	common	FALSE
Bms1	common	FALSE
Dnmt1	common	TRUE
Clspn	common	FALSE
Spata13	common	FALSE
Hnrnpm	common	FALSE
Rrm2	common	FALSE
Xpo1	common	FALSE
Anp32b	common	FALSE
Eif4b	common	FALSE
Nup93	common	FALSE
Top2a	common	FALSE
Ccne1	common	FALSE
Lap3	common	FALSE
Phactr1	common	FALSE
Cdc20	common	FALSE
Psme3	common	FALSE
Hat1	common	FALSE
Nup188	common	FALSE
Peg10	common	FALSE
Rpa1	common	FALSE
Nol9	common	FALSE
Paics	common	FALSE
Hoxd3	common	TRUE
Ndc1	common	FALSE
Fam210b	common	FALSE
Sae1	common	FALSE
Tfap2a	common	TRUE
Ccnb1	common	FALSE
Gpc3	common	FALSE
Utp20	common	FALSE
E2f3	common	TRUE
Fn1	common	FALSE
Hnrnpf	common	FALSE
Tuba1b	common	FALSE
Tcp1	common	FALSE
Pus7	common	FALSE
Uck2	common	FALSE
Chek1	common	FALSE
Tex10	common	FALSE
Topbp1	common	FALSE
Sephs1	common	FALSE
Aif1l	common	FALSE
Plagl2	common	TRUE
Tpx2	common	FALSE
Lrig1	common	FALSE
Pcdh18	common	FALSE
Trim59	common	FALSE

Rcbtb2	common	FALSE
Pold1	common	FALSE
Tcf12	common	TRUE
Xpo5	common	FALSE
Mre11a	common	FALSE
2610318N(	common	FALSE
Ddx3x	common	FALSE
Ptges3	common	FALSE
Tuba1c	common	FALSE
Kif22	common	TRUE
Nup50	common	FALSE
Nup155	common	FALSE
Nup205	common	FALSE
Gnl3	common	FALSE
Pola1	common	FALSE
Erbb3	common	FALSE
Nup153	common	FALSE
Chd1	common	TRUE
Aaas	common	FALSE
Sufu	common	FALSE
Rfwd3	common	FALSE
Bend3	common	FALSE
Pitrm1	common	FALSE
Rsl1d1	common	FALSE
Vcan	common	FALSE
Nr2f6	common	TRUE
Prps2	common	FALSE
Ifrd2	common	FALSE
Adsl	common	FALSE
Naa50	common	FALSE
Knop1	common	FALSE
Tacc3	common	FALSE
Dtl	common	TRUE
Eef1d	common	TRUE
Mlec	common	FALSE
Dhfr	common	FALSE
Ilf3	common	FALSE
Gcsh	common	FALSE
Fam181b	common	FALSE
Hnrnpk	common	FALSE
Suclg2	common	FALSE
Ncbp1	common	FALSE
Fzd7	common	FALSE
Smarcc1	common	TRUE
Zfp106	common	FALSE
Lig1	common	FALSE
Gpr125	common	FALSE
Dbf4	common	FALSE
Eif2s1	common	FALSE
Prps1l3	common	FALSE

Nup37	common	FALSE
Noc2l	common	TRUE
Galk1	common	FALSE
Uba2	common	FALSE
Ddx11	common	FALSE
Srsf1	common	FALSE
Dnaja2	common	FALSE
Acsl4	common	FALSE
Ikbkap	common	FALSE
Bcar3	common	FALSE
Klhl21	common	FALSE
Cdt1	common	FALSE
Mki67	common	FALSE
Ddx18	common	FALSE
Nup98	common	FALSE
Mms22l	common	FALSE
Nup85	common	FALSE
Xpo4	common	FALSE
Ppid	common	FALSE
Chtf18	common	FALSE
Spred1	common	FALSE
Phgdh	common	FALSE
1700019D(	common	FALSE
Tbl3	common	FALSE
Psmd2	common	FALSE
Cdk2	common	FALSE
Bora	common	FALSE
Tk1	common	FALSE
Cluh	common	FALSE
Pno1	common	FALSE
Rif1	common	FALSE
Dek	common	FALSE
Tbc1d1	common	FALSE
Ado	common	FALSE
Atad3a	common	FALSE
Srsf7	common	FALSE
Chtf8	common	FALSE
Cdca8	common	FALSE
Trim27	common	FALSE
Pole2	common	FALSE
Tyms	common	FALSE
Agps	common	FALSE
Exosc2	common	FALSE
Tmc6	common	FALSE
Ddx1	common	FALSE
Dpy19l1	common	FALSE
Eif3l	common	FALSE
Mrps27	common	FALSE
Ttf2	common	FALSE
Shcbp1	common	FALSE

Ncaph	common	FALSE
Ccna2	common	FALSE
Thop1	common	FALSE
Slc39a10	common	FALSE
Rpa2	common	FALSE
Elavl1	common	FALSE
S1pr2	common	FALSE
Sf1	common	TRUE
Hoxb2	common	TRUE
Nup160	common	FALSE
Nudt5	common	FALSE
Nup43	common	FALSE
Brca2	common	FALSE
Hnrnpll	common	TRUE
Pcna	common	FALSE
Gpd2	common	FALSE
Timeless	common	TRUE
E2f4	common	TRUE
Ppif	common	FALSE
Nol11	common	FALSE
Srsf10	common	FALSE
Eif5	common	FALSE
Ints7	common	FALSE
Hus1	common	FALSE
Pds5a	common	TRUE
Rasa3	common	FALSE
Dhx33	common	FALSE
Prmt3	common	FALSE
Ing5	common	FALSE
Sf3a3	common	FALSE
Strap	common	FALSE
Anapc1	common	FALSE
Ssr1	common	FALSE
Rbpms	common	FALSE
Sf3a1	common	FALSE
Pwp1	common	FALSE
Ncapd2	common	FALSE
Rpl7l1	common	FALSE
Sapcd2	common	FALSE
Ipo9	common	FALSE
Fhdc1	common	FALSE
Txndc5	common	FALSE
Nid2	common	FALSE
Tfdp1	common	TRUE
Zc3hav1l	common	FALSE
Slbp	common	FALSE
Paxip1	common	TRUE
Eif4e	common	FALSE
Spg20	common	FALSE
Cdca4	common	FALSE

Sall2	common	TRUE
Luzp1	common	TRUE
Polr1a	common	FALSE
Zadh2	common	FALSE
Kcnk5	common	FALSE
Ddb1	common	FALSE
Ruvbl1	common	TRUE
Gemin6	common	FALSE
Hdlbp	common	FALSE
Megf6	common	FALSE
Usp37	common	FALSE
Ahctf1	common	TRUE
Vrk1	common	FALSE
Wdr5	common	FALSE
Tmem132e	common	FALSE
Fam60a	common	FALSE
Fubp1	common	TRUE
Snap23	common	FALSE
Btaf1	common	FALSE
Usp39	common	TRUE
Tgif2	common	TRUE
Cpsf2	common	FALSE
Mdk	common	FALSE
Dcp2	common	FALSE
Rest	common	TRUE
Me2	common	FALSE
Nono	common	TRUE
Actl6a	common	FALSE
Eif4ebp1	common	FALSE
Tbl1x	common	FALSE
Nde1	common	FALSE
E2f5	common	TRUE
Nmd3	common	FALSE
Pola2	common	FALSE
Wdr18	common	FALSE
Carm1	common	FALSE
Ranbp1	common	FALSE
Dnph1	common	FALSE
Nup62	common	FALSE
Coa5	common	FALSE
Cand2	common	FALSE
Nme4	common	FALSE
Tra2b	common	FALSE
Lmnb1	common	FALSE
Cnot1	common	FALSE
Pdlim1	common	FALSE
Pes1	common	FALSE
Igf2bp3	common	FALSE
Slc19a1	common	FALSE
Pdk3	common	FALSE

Ubfd1	common	FALSE
Tpp2	common	FALSE
Rpl3	common	FALSE
Clns1a	common	FALSE
Exosc6	common	FALSE
Ezh2	common	TRUE
Smarca5	common	TRUE
Eif2s3x	common	FALSE
Birc5	common	FALSE
Kdelc1	common	FALSE
Ubqln4	common	FALSE
Scube1	common	FALSE
Rbmxl1	common	FALSE
2410016O(	common	FALSE
Sulf2	common	FALSE
Slc25a5	common	FALSE
Hmga1	common	TRUE
Rqcd1	common	FALSE
Haus3	common	FALSE
Mns1	common	FALSE
Smek1	common	FALSE
Rbm38	common	FALSE
Smc2	common	FALSE
Tardbp	common	FALSE
Saal1	common	FALSE
Drosha	common	FALSE
Cenpe	common	FALSE
Prdx1	common	FALSE
Pinx1	common	FALSE
Nhp2	common	FALSE
Usp1	common	FALSE
Knstrn	common	FALSE
Hoxb9	common	TRUE
5730559C1	common	FALSE
Naa40	common	FALSE
Prkrir	common	FALSE
Plekha8	common	FALSE
Ddx56	common	FALSE
Spats2	common	TRUE
Acot1	common	FALSE
Pold3	common	FALSE
Ccnb2	common	FALSE
Nup133	common	TRUE
Cdc25a	common	FALSE
Gtf2h3	common	TRUE
Evc2	common	FALSE
Prodh	common	FALSE
Myo10	common	FALSE
Eef1g	common	FALSE
Nudcd1	common	FALSE

Tubb6	common	FALSE
Cenph	common	FALSE
Nmt1	common	FALSE
Prkci	common	FALSE
Ddx27	common	FALSE
Snx18	common	FALSE
Smchd1	common	FALSE
Noc4l	common	FALSE
Dhx15	common	FALSE
Lig3	common	FALSE
Rfc5	common	FALSE
2810004N2	common	FALSE
Cdc27	common	FALSE
Pkn2	common	FALSE
Gpx3	common	FALSE
2810417H1	common	FALSE
Phb2	common	FALSE
Pvrl3	common	FALSE
Polr2b	common	FALSE
Fastkd2	common	FALSE
Lrp4	common	FALSE
Pdha1	common	FALSE
Eed	common	FALSE
Hspe1	common	FALSE
Mtbp	common	FALSE
Gmnn	common	FALSE
Cnn2	common	FALSE
Asnsd1	common	FALSE
Adi1	common	FALSE
Casp8ap2	common	FALSE
Snrpa1	common	FALSE
Msh2	common	FALSE
Hspa14	common	FALSE
Fign	common	FALSE
Flrt3	common	FALSE
Impdh2	common	FALSE
D10Wsu10	common	FALSE
Cdk1	common	FALSE
Ranbp2	common	FALSE
Sox13	common	TRUE
Snrnp40	common	FALSE
Cpsf6	common	FALSE
Exosc8	common	FALSE
Thrap3	common	FALSE
Dnajc2	common	FALSE
Ppp4c	common	FALSE
Nedd4	common	FALSE
Ythdf3	common	FALSE
Atad5	common	FALSE
Stoml2	common	FALSE

Lsr	common	FALSE
Phactr2	common	FALSE
Spc24	common	FALSE
Rae1	common	FALSE
Dnajc9	common	FALSE
Smpd4	common	FALSE
Grk6	common	FALSE
Lpar6	common	FALSE
Itga5	common	FALSE
Pus7l	common	FALSE
Ubr7	common	FALSE
Cenpa	common	FALSE
Zfp266	common	TRUE
Hnrnpc	common	TRUE
Ppp2r5d	common	FALSE
Fanca	common	FALSE
Smg7	common	FALSE
Mis18bp1	common	FALSE
Cstf1	common	FALSE
St13	common	FALSE
Prpf4	common	FALSE
Prpf31	common	FALSE
Chd1l	common	FALSE
Haglr	common	FALSE
Trip10	common	TRUE
Khsrp	common	FALSE
Heyl	common	TRUE
Ints2	common	FALSE
Arf6	common	FALSE
Mycbp	common	FALSE
Mettl2	common	FALSE
Zfp41	common	TRUE
Oat	common	FALSE
Cdc23	common	FALSE
Spcs3	common	FALSE
Ung	common	FALSE
Scaf11	common	FALSE
Cntrl	common	FALSE
Lrrc40	common	FALSE
Eif4a3	common	FALSE
Hmgb2	common	TRUE
Dus1l	common	FALSE
Hmgn1	common	FALSE
Bend4	common	FALSE
Ncapd3	common	FALSE
Eml4	common	FALSE
Ninl	common	FALSE
Amotl1	common	FALSE
Zfp207	common	TRUE
Dnttip2	common	FALSE

Tipin	common	FALSE
Cpsf3l	common	FALSE
Rbm14	common	FALSE
Lrwd1	common	FALSE
Slc20a2	common	FALSE
Xrcc6	common	FALSE
Golt1b	common	FALSE
Dzip1l	common	FALSE
Tcf3	common	TRUE
Enpp2	common	FALSE
Gapdh	common	FALSE
Pgam1	common	FALSE
Syncrip	common	FALSE
Rgma	common	FALSE
Ubap2l	common	FALSE
Hoxa3	common	TRUE
Nip7	common	FALSE
B3galnt2	common	FALSE
Cdca3	common	FALSE
Lmnb2	common	FALSE
Map2k6	common	FALSE
Atm	common	FALSE
Rpl4	common	FALSE
Tes	common	FALSE
Cbx1	common	FALSE
Rcbtb1	common	FALSE
Msto1	common	FALSE
Taf5l	common	FALSE
Ptpn2	common	FALSE
Adss	common	FALSE
Cdc7	common	FALSE
Hirip3	common	TRUE
Tcerg1	common	FALSE
Exosc3	common	TRUE
Nbn	common	FALSE
Zcchc3	common	FALSE
Ddx52	common	FALSE
Ugdh	common	FALSE
Fzd2	common	FALSE
Trp53	common	TRUE
H2afx	common	FALSE
Iars2	common	FALSE
Psmg1	common	FALSE
Prpf3	common	FALSE
Rnf144a	common	FALSE
Cwc22	common	FALSE
Zkscan17	common	TRUE
Phldb2	common	FALSE
Tyms-ps	common	FALSE
Tada2a	common	FALSE

Sox2	common	TRUE
Ckap2	common	FALSE
Polr2h	common	FALSE
Gpn1	common	FALSE
Srsf3	common	FALSE
Rbm15	common	FALSE
Whsc1	common	FALSE
Psrc1	common	FALSE
Naa16	common	FALSE
Wbp11	common	FALSE
Snrnp200	common	FALSE
Piezo1	common	FALSE
Lmo4	common	FALSE
Aen	common	FALSE
Tgif1	common	TRUE
Csde1	common	FALSE
Ttc23	common	FALSE
Pi4k2b	common	FALSE
Prpf8	common	FALSE
Csrp2bp	common	FALSE
Cenpk	common	FALSE
Golm1	common	FALSE
Tmem47	common	FALSE
Cep192	common	FALSE
Tfam	common	TRUE
Wdr76	common	FALSE
Itga6	common	FALSE
Papola	common	FALSE
Pelp1	common	FALSE
Uchl5	common	FALSE
Ormdl1	common	FALSE
Eif3c	common	FALSE
Mif	common	FALSE
Cdc16	common	FALSE
Foxp4	common	TRUE
Lsm2	common	FALSE
Cmtm7	common	FALSE
Sox10	common	TRUE
D2Wsu81e	common	FALSE
Pdia5	common	FALSE
Ngfr	common	FALSE
Isy1	common	FALSE
Eif2s2	common	FALSE
Ptp4a2	common	FALSE
Rfc1	common	FALSE
Gatad2a	common	FALSE
Vangl1	common	FALSE
Stk24	common	FALSE
Tead2	common	TRUE
Hnrnpdl	common	FALSE

Nxt1	common	FALSE
Sdha	common	FALSE
Prim1	common	FALSE
Dctpp1	common	FALSE
Ppih	common	FALSE
Emilin1	common	FALSE
Rrp15	common	FALSE
Llgl1	common	FALSE
Nt5dc2	common	FALSE
Casd1	common	FALSE
Cdc42ep1	common	FALSE
Ecd	common	FALSE
Sart3	common	FALSE
Dusp4	common	FALSE
Kat2a	common	TRUE
Ide	common	FALSE
Polr3e	common	FALSE
Ola1	common	FALSE
Gins2	common	FALSE
Pigl	common	FALSE
Ddx19a	common	FALSE
Rsl24d1	common	FALSE
Ube2f	common	FALSE
Cherp	common	FALSE
Nmral1	common	TRUE
Nudcd2	common	FALSE
AI506816	common	FALSE
A430005L1	common	FALSE
Fubp3	common	FALSE
Slc9a3r1	common	FALSE
Klhdc4	common	FALSE
Raver1	common	FALSE
Elp2	common	FALSE
Pop1	common	FALSE
Rrp8	common	FALSE
Supv3l1	common	FALSE
1700017BC	common	FALSE
Gas5	common	FALSE
Slc35a4	common	FALSE
Prim2	common	FALSE
Gdpd5	common	FALSE
Tsen15	common	FALSE
Qser1	common	FALSE
Qtrtd1	common	FALSE
Zbtb1	common	TRUE
Utrn	common	FALSE
Hsd11b2	common	FALSE
Yars2	common	FALSE
Psmc3ip	common	FALSE
Inip	common	FALSE

Rad50	common	FALSE
Ephb4	common	FALSE
Soat1	common	FALSE
Xpo7	common	FALSE
Zfp462	common	FALSE
Tamm41	common	FALSE
Snhg3	common	FALSE
Fgd1	common	FALSE
Brix1	common	FALSE
Pms2	common	FALSE
Fam206a	common	FALSE
Prpf40a	common	FALSE
Slmap	common	FALSE
Tmtc3	common	FALSE
Cenpp	common	FALSE
Fam136a	common	FALSE
Dhx8	common	FALSE
Dock1	common	FALSE
Dr1	common	TRUE
Zc3h18	common	FALSE
Ddx47	common	FALSE
Nmt2	common	FALSE
Pabpc4	common	FALSE
Metrn	common	FALSE
Nr2f1	common	TRUE
Klhl13	common	FALSE
Myh10	common	FALSE
Dcaf13	common	FALSE
Siva1	common	FALSE
Ttll12	common	FALSE
Blmh	common	FALSE
Taf1d	common	FALSE
Setd8	common	FALSE
Pdcd11	common	TRUE
Spon1	common	FALSE
Cd3eap	common	FALSE
Rfc4	common	FALSE
Tsen54	common	FALSE
Mapk14	common	FALSE
Med14	common	FALSE
Eif3m	common	FALSE
Drg1	common	FALSE
Homer3	common	FALSE
Gsto1	common	FALSE
Cks1b	common	FALSE
Xrcc5	common	FALSE
Poc1b	common	FALSE
Rfc2	common	TRUE
9430015G1	common	FALSE
AI314180	common	FALSE

Fh1	common	FALSE
Cdc34	common	FALSE
Orc6	common	FALSE
Rbm12	common	FALSE
Med1	common	FALSE
Ddx39b	common	FALSE
Ints9	common	FALSE
Foxn2	common	TRUE
Nacc1	common	FALSE
Tubgcp4	common	FALSE
Tsen2	common	FALSE
Scaf4	common	FALSE
Xrcc1	common	TRUE
Alg8	common	FALSE
Nrf1	common	TRUE
Donson	common	FALSE
Tshz1	common	FALSE
Ppil1	common	FALSE
Champ1	common	FALSE
Tgs1	common	FALSE
Slc20a1	common	FALSE
Gid4	common	FALSE
Hoxd4	common	TRUE
Phb	common	FALSE
Ppp1r15b	common	FALSE
Bclaf1	common	TRUE
Zfp422	common	TRUE
Eri1	common	FALSE
Ss18	common	FALSE
Slc43a1	common	FALSE
Nt5c3b	common	FALSE
Sin3a	common	TRUE
Mettl1	common	FALSE
Nup214	common	FALSE
Shroom2	common	FALSE
Pigm	common	FALSE
Thumpd3	common	FALSE
Pbdc1	common	FALSE
U2af2	common	FALSE
Tdp2	common	FALSE
Txnl1	common	FALSE
Gtf2h4	common	FALSE
Utp14a	common	FALSE
Hnrnpab	common	FALSE
Pxn	common	FALSE
Trap1	common	FALSE
Tom1l1	common	FALSE
Rgs19	common	FALSE
Cebpz	common	TRUE
Psmc6	common	FALSE

Eif3i	common	FALSE
Rlim	common	FALSE
Utp6	common	FALSE
Eif2d	common	FALSE
Herc4	common	FALSE
Trip6	common	FALSE
Ythdf2	common	FALSE
Usp28	common	FALSE
Gabpa	common	TRUE
Gys1	common	FALSE
Gnb2l1	common	FALSE
Denr	common	FALSE
Atf1	common	TRUE
Cops2	common	FALSE
Morf4l2	common	FALSE
Mrfap1	common	FALSE
Fam199x	common	FALSE
N4bp2	common	FALSE
Tdp1	common	FALSE
Psmd5	common	FALSE
Senp1	common	FALSE
Lars	common	FALSE
Mex3c	common	TRUE
Stxbp4	common	FALSE
Cdk4	common	FALSE
Chsy1	common	FALSE
Terf1	common	TRUE
Hbb-y	common	FALSE
Fkbp3	common	FALSE
E130308A1	common	FALSE
Zc3hc1	common	FALSE
Bcat2	common	FALSE
Fbxo30	common	FALSE
Kif18b	common	FALSE
Plcb3	common	FALSE
Rangap1	common	FALSE
Hnrnpd	common	FALSE
Rfc3	common	TRUE
Ppfibp1	common	FALSE
Bud13	common	FALSE
Upf1	common	FALSE
Mbd4	common	TRUE
Skap2	common	FALSE
Rbm27	common	FALSE
Cpsf4	common	TRUE
Pold2	common	TRUE
Csk	common	FALSE
Trmt10a	common	FALSE
Gtf3c4	common	FALSE
Med17	common	FALSE

Zdhhc15	common	TRUE
Pbx2	common	TRUE
Ctdspl2	common	FALSE
Bmp7	common	FALSE
Fxr1	common	FALSE
Cdk8	common	FALSE
Azin1	common	FALSE
Msh3	common	FALSE
Ednrb	common	FALSE
Fancm	common	FALSE
Zw10	common	FALSE
Rpap3	common	FALSE
Ssr3	common	FALSE
Megf10	common	FALSE
Dhx38	common	FALSE
Pus3	common	FALSE
Rhobtb3	common	FALSE
Vwa9	common	FALSE
Nrarp	common	FALSE
Gjc1	common	FALSE
Mob1a	common	FALSE
Mcat	common	FALSE
Impact	common	FALSE
Eif3e	common	FALSE
Mapkapk2	common	FALSE
Haus4	common	FALSE
Tgm4	common	FALSE
Fam53a	common	FALSE
Phf5a	common	FALSE
Daxx	common	FALSE
Nme7	common	FALSE
Baz1a	common	FALSE
Plscr1	common	FALSE
Tug1	common	FALSE
Zzz3	common	TRUE
Metap1	common	FALSE
Eaf1	common	FALSE
Tmem55a	common	FALSE
Ephb3	common	FALSE
Ska2	common	FALSE
Epb4.1l5	common	FALSE
Nudt1	common	FALSE
Fip1l1	common	FALSE
Socs6	common	FALSE
Gfer	common	FALSE
Wapal	common	FALSE
Msantd3	common	FALSE
Lpin2	common	FALSE
Bahcc1	common	FALSE
Vcpkmt	common	FALSE

Asxl1	common	FALSE
Hoxb8	common	TRUE
Syde1	common	FALSE
Pom121	common	FALSE
Arcn1	common	FALSE
Myh9	common	FALSE
Thoc1	common	FALSE
AY358078	common	FALSE
Thumpd1	common	FALSE
Fam111a	common	FALSE
Tmem164	common	FALSE
Manea	common	FALSE
Asun	common	FALSE
Ccdc43	common	FALSE
Hcfc1	common	TRUE
Emg1	common	FALSE
Ints8	common	FALSE
Tubgcp2	common	FALSE
Rbbp4	common	FALSE
Nvl	common	FALSE
Ears2	common	FALSE
6330549D2	common	FALSE
Cnot11	common	FALSE
Zfp334	common	TRUE
Plrg1	common	FALSE
Map3k11	common	FALSE
Pds5b	common	FALSE
Rbm15b	common	FALSE
Tars	common	FALSE
Rbm28	common	FALSE
Zdhhc6	common	FALSE
Eef1a1	common	FALSE
Trnt1	common	FALSE
Lypla1	common	FALSE
Ccar1	common	FALSE
Pabpc4l	common	FALSE
Hmgb3	common	TRUE
Rprd1b	common	FALSE
Cks2	common	FALSE
Hoxb3	common	TRUE
Cdyl	common	FALSE
Mpdz	common	FALSE
Prmt6	common	FALSE
Stk4	common	FALSE
Serp1	common	FALSE
Xxylt1	common	FALSE
4930579G2	common	FALSE
Gemin2	common	FALSE
Rpp30	common	FALSE
Elp3	common	FALSE

Bccip	common	FALSE
Bri3bp	common	FALSE
Heg1	common	FALSE
Sp1	common	TRUE
Gtf3c2	common	TRUE
Rpf1	common	FALSE
Patz1	common	TRUE
E2f6	common	TRUE
Eef1b2	common	FALSE
Anapc15	common	FALSE
Cenpm	common	FALSE
Snrpb	common	FALSE
Tfb1m	common	FALSE
Smn1	common	FALSE
Papd4	common	FALSE
Alg11	common	FALSE
Tjp1	common	FALSE
Mrpl1	common	TRUE
Peli2	common	FALSE
Ppp2r2a	common	FALSE
Bcl2l13	common	FALSE
Itch	common	FALSE
2810006K2	common	FALSE
Ints3	common	FALSE
Apex1	common	TRUE
Pmf1	common	FALSE
1810009A1	common	FALSE
Larp7	common	FALSE
U2surp	common	FALSE
Gm11974	common	FALSE
Aco1	common	TRUE
Hnrnph2	common	FALSE
Tti1	common	FALSE
Appbp2	common	FALSE
Ankrd49	common	FALSE
Parg	common	FALSE
Ubtd2	common	FALSE
Nme6	common	FALSE
Cd320	common	FALSE
Alkbh1	common	FALSE
Cul2	common	FALSE
Usp7	common	FALSE
Anp32a	common	FALSE
Noa1	common	FALSE
Atp13a3	common	FALSE
Pstk	common	FALSE
Prrc2c	common	FALSE
Cdk7	common	FALSE
Tex9	common	FALSE
Phf10	common	FALSE

Psmd12	common	TRUE
Plxna1	common	FALSE
Gnpat	common	FALSE
Lsg1	common	FALSE
Pard6g	common	FALSE
Dck	common	FALSE
Sfpq	common	TRUE
Cyfip1	common	FALSE
Exosc9	common	FALSE
Stag2	common	FALSE
Ccdc58	common	FALSE
D15Ertd62	common	FALSE
Dicer1	common	FALSE
Psma5	common	FALSE
Ggct	common	FALSE
Nae1	common	FALSE
Fat1	common	FALSE
Rexo4	common	FALSE
Hmbs	common	FALSE
Mesdc1	common	FALSE
Ptplb	common	FALSE
Polr2e	common	FALSE
Lonp1	common	FALSE
Psmd10	common	FALSE
Zcchc10	common	FALSE
Rnf138	common	TRUE
Prr11	common	FALSE
Elovl2	common	FALSE
Cactin	common	FALSE
Map2k3	common	FALSE
Pik3c2a	common	FALSE
Thoc6	common	FALSE
Pank2	common	FALSE
Rpusd4	common	FALSE
2810025M	common	FALSE
Pcbp1	common	FALSE
Pald1	common	FALSE
Gtf2h2	common	FALSE
Tmem39a	common	FALSE
Cops4	common	FALSE
Mrps2	common	FALSE
Etv6	common	TRUE
Slitrk2	common	FALSE
Ndufs1	common	FALSE
Dhx9	common	FALSE
Cpsf1	common	FALSE
Tstd2	common	FALSE
Ctbp2	common	TRUE
Hdac1	common	TRUE
Foxd3	common	TRUE

Gltp	common	FALSE
Dlg1	common	FALSE
Iws1	common	FALSE
Wbscr22	common	FALSE
Rnf2	common	FALSE
Rabl3	common	FALSE
Top1	common	FALSE
Srrt	common	FALSE
Vcp	common	FALSE
Lrp6	common	FALSE
Isyna1	common	FALSE
Slc4a7	common	FALSE
Alyref	common	FALSE
Pdgfc	common	FALSE
Setdb1	common	TRUE
Katna1	common	FALSE
Ago2	common	FALSE
Mcmbp	common	FALSE
Clint1	common	FALSE
Gtf3c3	common	FALSE
Smarcd1	common	FALSE
Cpsf3	common	FALSE
Cryz	common	FALSE
Ddx46	common	FALSE
Smu1	common	FALSE
Esf1	common	FALSE
Mrpl45	common	FALSE
Wrb	common	FALSE
Sox8	common	TRUE
Heatr5a	common	FALSE
Ctu2	common	FALSE
Mical1	common	FALSE
1110038B1	common	FALSE
Alkbh5	common	FALSE
Pmpca	common	FALSE
Rabggtb	common	FALSE
Zfp217	common	TRUE
Cpxm1	common	FALSE
Psmd3	common	FALSE
Hoxb7	common	TRUE
Rbm10	common	FALSE
Fam149a	common	FALSE
Ssb	common	FALSE
Slc12a7	common	FALSE
Mrps22	common	FALSE
Gemin8	common	FALSE
Sep-10	common	FALSE
Dusp12	common	FALSE
Rps2	common	FALSE
Topors	common	TRUE

Ddx54	common	FALSE
Pacs1	common	FALSE
Pcca	common	FALSE
Sde2	common	FALSE
Mccc2	common	FALSE
AI597479	common	FALSE
Baz1b	common	FALSE
Senp3	common	FALSE
Mad1l1	common	FALSE
Rbms1	common	TRUE
Zscan12	common	TRUE
Ube2d3	common	FALSE
Farp1	common	FALSE
Mtr	common	FALSE
Rpsa	common	FALSE
Dera	common	FALSE
Sf3b4	common	FALSE
Chst14	common	FALSE
Dsn1	common	FALSE
Scrn2	common	FALSE
Hif1a	common	TRUE
Eif2a	common	FALSE
Cul4a	common	FALSE
Nipa2	common	FALSE
Polr3f	common	FALSE
Polr3d	common	FALSE
Decr1	common	FALSE
Nhp2l1	common	FALSE
Tulp3	common	FALSE
Crk	common	FALSE
Cdc14b	common	FALSE
Pggt1b	common	FALSE
Zranb2	common	FALSE
Casp2	common	FALSE
Ercc3	common	FALSE
Gabpb2	common	FALSE
Mkln1	common	FALSE
Itga4	common	FALSE
Taf2	common	FALSE
Cltc	common	FALSE
Mrps30	common	FALSE
Dld	common	FALSE
Adam12	common	FALSE
Tirap	common	FALSE
Mta1	common	FALSE
Wls	common	FALSE
Exosc5	common	FALSE
Uri1	common	FALSE
Snw1	common	FALSE
Celsr1	common	FALSE

Cep76	common	FALSE
Haus1	common	FALSE
Cul3	common	FALSE
Matr3	common	FALSE
2700094K1	common	FALSE
Gzf1	common	TRUE
Thoc3	common	FALSE
Ythdf1	common	FALSE
Ehmt1	common	FALSE
Sympk	common	FALSE
Abcg2	common	FALSE
2900097C1	common	FALSE
Arih2	common	FALSE
Ubap2	common	FALSE
Ampd2	common	FALSE
Alad	common	FALSE
Eya3	common	FALSE
Taf6	common	FALSE
Sclt1	common	FALSE
Psmd7	common	FALSE
Galnt1	common	FALSE
Brd7	common	FALSE
Mief1	common	TRUE
Rimklb	common	FALSE
Utp3	common	FALSE
Axin1	common	FALSE
Mknk2	common	FALSE
Gpn2	common	FALSE
Nrp1	common	FALSE
Dhx16	common	FALSE
Ttc9c	common	FALSE
Lsm12	common	FALSE
A130010J1	common	FALSE
Orc4	common	FALSE
Prdm5	common	TRUE
Tsr2	common	FALSE
Hif1an	common	FALSE
Cbl	common	FALSE
Mlh1	common	FALSE
Nfya	common	TRUE
Rdx	common	FALSE
Nelfa	common	TRUE
Zfp518b	common	FALSE
Adprhl2	common	FALSE
4930503L1	common	FALSE
Sars2	common	FALSE
Adpgk	common	FALSE
E130309D(	common	FALSE
Erlin1	common	FALSE
Kpna4	common	FALSE

Hba-x	common	FALSE
Aldh16a1	common	FALSE
Sod1	common	TRUE
Ppp1r8	common	FALSE
Dhx35	common	FALSE
Chd7	common	FALSE
Prkab1	common	FALSE
Atl2	common	FALSE
Cep68	common	FALSE
D830031N(	common	FALSE
Glyr1	common	FALSE
Taf1	common	TRUE
Ppie	common	FALSE
Hmcn1	common	FALSE
Hip1	common	FALSE
Rcc2	common	FALSE
Chrna4	common	FALSE
Slc4a2	common	FALSE
Fancl	common	FALSE
Dtymk	common	FALSE
Ddx41	common	FALSE
Nthl1	common	FALSE
Smg8	common	FALSE
Orc3	common	FALSE
Plp1	common	FALSE
Coq4	common	FALSE
Gli2	common	TRUE
Gtf2e2	common	FALSE
Ywhaq	common	FALSE
Pspc1	common	FALSE
Mta2	common	FALSE
2610507B1	common	FALSE
Tsfm	common	FALSE
Dse	common	FALSE
Tubgcp3	common	FALSE
Trim62	common	FALSE
Pgam5	common	FALSE
Mapk6	common	FALSE
Parn	common	FALSE
Tmx3	common	FALSE
Cab39l	common	FALSE
Rtcb	common	FALSE
Cdc14a	common	FALSE
Prelid2	common	FALSE
Sec23ip	common	FALSE
Pum2	common	FALSE
Polr3c	common	FALSE
Cdk12	common	FALSE
Spidr	common	FALSE
Mtfr1	common	FALSE

Poc1a	common	FALSE
Ndufaf4	common	FALSE
Rplp0	common	FALSE
Arl6ip6	common	FALSE
Kirrel	common	FALSE
Taf6l	common	FALSE
Pgd	common	FALSE
Pdf	common	FALSE
Nras	common	FALSE
Nufip2	common	FALSE
Zfp26	common	FALSE
Rpp14	common	FALSE
Trmt1	common	TRUE
Lrrfip1	common	TRUE
Fam178a	common	FALSE
Hdac3	common	TRUE
Elp4	common	FALSE
Ctcf	common	TRUE
Snrnp70	common	TRUE
Trub2	common	FALSE
Unc119	common	FALSE
Nin	common	FALSE
Cotl1	common	FALSE
Psmc4	common	FALSE
Tpr	common	FALSE
Pum1	common	FALSE
Ankrd26	common	FALSE
Polr2d	common	FALSE
Qrich1	common	FALSE
Atg5	common	FALSE
Zfp961	common	TRUE
Parp2	common	FALSE
Lfng	common	FALSE
Cnnm4	common	FALSE
Ndufaf2	common	FALSE
Raly	common	FALSE
Oard1	common	FALSE
Prkra	common	FALSE
Psme4	common	FALSE
Rps6	common	FALSE
Ltn1	common	FALSE
Specc1	common	FALSE
Obfc1	common	FALSE
Gale	common	FALSE
Fbxw8	common	FALSE
Smg5	common	FALSE
Smim15	common	FALSE
Apaf1	common	FALSE
Polr1c	common	FALSE
Tmem248	common	FALSE

Dpp3	common	FALSE
Yeats2	common	FALSE
Anp32e	common	FALSE
Tsku	common	FALSE
Repin1	common	FALSE
Ube2v1	common	TRUE
Maml1	common	FALSE
Sco1	common	FALSE
Leo1	common	FALSE
Rilpl1	common	FALSE
Rnf4	common	FALSE
Sub1	common	FALSE
Pdpn	common	FALSE
Asf1a	common	FALSE
2310022A1	common	FALSE
Erh	common	FALSE
Mtf2	common	FALSE
Coro1c	common	FALSE
Rpl15	common	FALSE
Nck1	common	FALSE
Mier3	common	FALSE
Ptrh2	common	FALSE
N4bp1	common	FALSE
Zfx	common	TRUE
Ext1	common	FALSE
Ctc1	common	FALSE
Polr2a	common	FALSE
Aqr	common	FALSE
Cacul1	common	FALSE
Nudt7	common	FALSE
Pigq	common	FALSE
Fam212a	common	FALSE
Glce	common	FALSE
Edc3	common	FALSE
Phrf1	common	FALSE
Nfxl1	common	FALSE
Abl1	common	TRUE
H2afz	common	TRUE
Atr	common	FALSE
Zfp213	common	TRUE
Hdgfrp2	common	FALSE
Lrrcc1	common	FALSE
Mettl3	common	TRUE
6030458C1	common	FALSE
Fut8	common	FALSE
Hnrnpr	common	FALSE
Lasp1	common	FALSE
Rars2	common	FALSE
Vkorc1l1	common	FALSE
Ikzf5	common	TRUE

Tmem123	common	FALSE
Mrpl3	common	FALSE
Zfp322a	common	TRUE
Tnpo1	common	FALSE
Ccdc77	common	FALSE
Tank	common	FALSE
Hes1	common	TRUE
Cnot3	common	TRUE
Acad9	common	FALSE
Rreb1	common	TRUE
Sec23b	common	FALSE
Nfyb	common	TRUE
Rbbp8	common	FALSE
Eif1ax	common	FALSE
Tgfbr1	common	FALSE
Pnn	common	FALSE
Foxred1	common	FALSE
Cd2ap	common	FALSE
Gpam	common	TRUE
Glo1	common	FALSE
Ndufaf1	common	FALSE
Nhlrc2	common	FALSE
Sntb2	common	FALSE
Mrps35	common	FALSE
Riok2	common	FALSE
Gfra1	common	FALSE
Smc5	common	FALSE
Csnk2a1	common	FALSE
Sppl2b	common	FALSE
Dvl2	common	FALSE
Mier1	common	FALSE
Rnft1	common	FALSE
Sf3b1	common	TRUE
Capza1	common	FALSE
Rfx7	common	TRUE
Dhx40	common	FALSE
Mphosph9	common	FALSE
Hmgxb4	common	FALSE
Nrm	common	FALSE
Hoxa5	common	TRUE
Adh5	common	FALSE
Rnf26	common	FALSE
Pkp4	common	FALSE
Ash2l	common	TRUE
Alg6	common	FALSE
Rps3a1	common	FALSE
Snhg7	common	FALSE
Cops7b	common	FALSE
Prpf38a	common	FALSE
Maml2	common	FALSE

Nsmce4a	common	FALSE
Agfg1	common	FALSE
Cep57	common	FALSE
Zrsr2	common	FALSE
Ctnna1	common	FALSE
Gcn1l1	common	FALSE
Pbx3	common	TRUE
Rbm17	common	TRUE
Zfp710	common	TRUE
Prr5	common	FALSE
Tubg1	common	FALSE
9430008C0	common	FALSE
Mettl6	common	FALSE
Rnf40	common	FALSE
Dnajc10	common	FALSE
Wdr92	common	FALSE
Hnrnpa2b1	common	FALSE
Uchl4	common	FALSE
Eif2ak4	common	FALSE
Qars	common	FALSE
Smndc1	common	FALSE
Klhl22	common	FALSE
Atpaf2	common	FALSE
Mpg	common	FALSE
Myl9	common	FALSE
Txlna	common	FALSE
Ccnh	common	FALSE
Dpy19l3	common	FALSE
Tnks2	common	FALSE
Dscr3	common	FALSE
Snhg1	common	FALSE
Fance	common	FALSE
Cnot10	common	FALSE
Plin2	common	FALSE
Man1a2	common	FALSE
Mpp5	common	FALSE
Pfn1	common	FALSE
Psma1	common	FALSE
E430025E2	common	FALSE
Emc8	common	FALSE
Ift74	common	FALSE
Sumo3	common	FALSE
Dusp7	common	FALSE
Zfp629	common	TRUE
Ranbp3	common	FALSE
Patl1	common	FALSE
Nutf2	common	FALSE
Kif7	common	FALSE
Ssfa2	common	FALSE
Bmpr1a	common	FALSE

Yeats4	common	TRUE
Nus1	common	FALSE
Gdi2	common	FALSE
Mios	common	TRUE
Rbms2	common	FALSE
Tmed5	common	FALSE
Jmjd4	common	FALSE
Qk	common	FALSE
Rin2	common	FALSE
Pacsin2	common	FALSE
Cstf3	common	FALSE
Dpp8	common	FALSE
Caap1	common	FALSE
Zfp507	common	TRUE
Fbxl14	common	FALSE
Marcksl1	common	FALSE
Stk3	common	FALSE
Desi2	common	FALSE
Eif4enif1	common	FALSE
Fadd	common	FALSE
Usp3	common	FALSE
Uhrf2	common	FALSE
C330018D2	common	FALSE
Igfbp2	common	FALSE
Cdc73	common	FALSE
Bckdk	common	FALSE
Dancr	common	FALSE
Katnbl1	common	FALSE
Nit2	common	FALSE
Wdr11	common	FALSE
Sos1	common	FALSE
Ppp6r3	common	FALSE
Bap1	common	FALSE
Tyw5	common	FALSE
1200014J1	common	FALSE
Wdr61	common	FALSE
Nudt21	common	FALSE
Tcf7l1	common	TRUE
Ccdc137	common	FALSE
Pbx1	common	TRUE
Rnmt	common	FALSE
Slc25a37	common	FALSE
Ptpn14	common	FALSE
Pgm1	common	FALSE
Pycrl	common	FALSE
Tpm4	common	FALSE
Poldip3	common	FALSE
Rpl27a	common	FALSE
Ibtk	common	FALSE
Zdhhc5	common	TRUE

Qrsl1	common	FALSE
Senp5	common	FALSE
Samhd1	common	FALSE
Cox18	common	FALSE
Zmynd8	common	FALSE
Psip1	common	FALSE
Prkd3	common	FALSE
Arpp19	common	FALSE
1810055G(	common	FALSE
Med8	common	FALSE
Ppap2c	common	FALSE
Smim12	common	FALSE
Phf13	common	FALSE
Psmg2	common	FALSE
Osbpl1a	common	FALSE
Rabl6	common	FALSE
Actn4	common	FALSE
Uba3	common	FALSE
Kctd20	common	FALSE
Mrps9	common	FALSE
Akap8	common	FALSE
Rpl12	common	FALSE
Acad8	common	FALSE
Zbed4	common	FALSE
Mrps25	common	TRUE
Olfml3	common	FALSE
Mllt1	common	FALSE
Crnkl1	common	FALSE
B3glct	common	FALSE
Nelfe	common	TRUE
Stk40	common	FALSE
Taf12	common	FALSE
Rps27a	common	FALSE
Acp1	common	FALSE
Tomm5	common	FALSE
Ice1	common	FALSE
Magoh	common	TRUE
9530082P2	common	FALSE
Ccdc25	common	TRUE
Itgb3bp	common	FALSE
Sp3	common	TRUE
Foxo4	common	TRUE
Adnp2	common	TRUE
Zfp518a	common	FALSE
Trim32	common	FALSE
Ap1g1	common	FALSE
Fam49b	common	FALSE
Arl14ep	common	FALSE
Tmem216	common	FALSE
Eif2ak1	common	FALSE

Dhx36	common	TRUE
Strada	common	FALSE
Lzic	common	FALSE
Mesdc2	common	FALSE
Usp47	common	FALSE
Gtf3c6	common	FALSE
Scap	common	FALSE
Plcg1	common	FALSE
Smg1	common	FALSE
Ppfia1	common	FALSE
Fam118a	common	FALSE
Tm9sf3	common	FALSE
Srp68	common	FALSE
Pde12	common	FALSE
Med16	common	FALSE
Eif1ad	common	FALSE
Mcm3ap	common	FALSE
Stt3a	common	FALSE
Naca	common	FALSE
Drg2	common	FALSE
Mut	common	FALSE
Suz12	common	TRUE
Zfp516	common	TRUE
Slc35a1	common	FALSE
Fam210a	common	FALSE
Rara	common	TRUE
Aven	common	TRUE
Tspan12	common	FALSE
Dcp1a	common	FALSE
Sh3rf1	common	FALSE
Elmsan1	common	FALSE
Nxn	common	FALSE
Zfp275	common	TRUE
Kdm6a	common	FALSE
Snrpd1	common	FALSE
Mfsd1	common	FALSE
Lpar3	common	FALSE
Yy1	common	TRUE
1810032O(	common	FALSE
Tmem165	common	FALSE
Spire1	common	FALSE
Med6	common	FALSE
Limd2	common	FALSE
Anapc7	common	FALSE
Ppp4r2	common	FALSE
Hs2st1	common	FALSE
Chordc1	common	FALSE
Tysnd1	common	FALSE
Rab13	common	FALSE
Zc3h4	common	FALSE

Arid2	common	FALSE
Pxdn	common	FALSE
Zfp317	common	TRUE
Dcun1d4	common	FALSE
Memo1	common	FALSE
Scly	common	FALSE
Szrd1	common	FALSE
Ppm1m	common	FALSE
Thyn1	common	FALSE
Kcmf1	common	FALSE
Flna	common	FALSE
Gm6524	common	FALSE
Rnaseh2a	common	FALSE
Zfp574	common	TRUE
Wtip	common	FALSE
Lsm14a	common	FALSE
Myrf	common	TRUE
Vangl2	common	FALSE
Id3	common	FALSE
Rhot2	common	FALSE
Aldh7a1	common	FALSE
Nploc4	common	FALSE
Mtmr2	common	FALSE
Taf9	common	TRUE
Usp6nl	common	FALSE
Psmg3	common	FALSE
Papss1	common	FALSE
Rps23	common	FALSE
Usp40	common	FALSE
Dcbld1	common	FALSE
Exosc10	common	FALSE
Ints1	common	FALSE
Smarcad1	common	FALSE
Asb4	common	FALSE
Ppil3	common	FALSE
A230046K(	common	FALSE
Kif2a	common	FALSE
Wasf1	common	FALSE
Pycr2	common	FALSE
Pcm1	common	FALSE
Zbtb14	common	TRUE
Itpripl1	common	FALSE
Snx33	common	FALSE
Lsm8	common	FALSE
Mrpl15	common	FALSE
Dagla	common	FALSE
Evc	common	FALSE
Abt1	common	FALSE
Cnot2	common	FALSE
Huwe1	common	FALSE

Lman1	common	FALSE
Bcl2l12	common	FALSE
Pigf	common	FALSE
Ube2g1	common	FALSE
Zcchc8	common	FALSE
Eif5b	common	FALSE
Ing3	common	TRUE
Dph7	common	FALSE
Tlk1	common	FALSE
Fam129b	common	FALSE
Rap2c	common	FALSE
Reep4	common	FALSE
Polr1d	common	FALSE
Prpsap1	common	FALSE
Prpf39	common	FALSE
Mavs	common	FALSE
Pole4	common	TRUE
Rbl1	common	FALSE
Tmem167	common	FALSE
2310033P0	common	FALSE
Mrpl50	common	FALSE
Cggbp1	common	FALSE
Poll	common	FALSE
Ubp1	common	TRUE
Casp8	common	FALSE
Snord96a	common	FALSE
Ccz1	common	FALSE
Snd1	common	TRUE
Hbs1l	common	FALSE
Ttc32	common	FALSE
Plcd1	common	FALSE
LOC102634	common	FALSE
Kitl	common	FALSE
Srsf4	common	FALSE
Ptk7	common	FALSE
Smek2	common	FALSE
Npat	common	FALSE
2410004N0	common	FALSE
Ptprg	common	FALSE
Nap1l4	common	FALSE
Ktn1	common	FALSE
Prpf4b	common	FALSE
Ppm1f	common	FALSE
Rhot1	common	FALSE
Zfp260	common	TRUE
Rnf8	common	FALSE
Cdc37	common	FALSE
Ston2	common	FALSE
Pdzd8	common	FALSE
Tyw1	common	FALSE

Opa1	common	FALSE
Ugcg	common	FALSE
Ubr5	common	FALSE
Tcf20	common	FALSE
Msi1	common	TRUE
Josd1	common	FALSE
1110008F1	common	FALSE
Akap10	common	FALSE
Arhgap5	common	FALSE
Rcn1	common	FALSE
Mitd1	common	FALSE
Hnrnpul1	common	FALSE
Rnf149	common	FALSE
Ovca2	common	FALSE
Mrps7	common	FALSE
Tyro3	common	FALSE
Zfp105	common	TRUE
Hyal2	common	FALSE
Mum1	common	FALSE
Smc6	common	FALSE
Ccm2	common	FALSE
2810474O1	common	FALSE
Nfatc3	common	TRUE
Snrpd3	common	FALSE
Rpap1	common	FALSE
Mrpl44	common	FALSE
Ddx42	common	FALSE
Gcnt1	common	FALSE
Zscan22	common	TRUE
Rpl7	common	FALSE
Tada1	common	FALSE
Hsf1	common	TRUE
Zfp35	common	TRUE
Zc3h15	common	FALSE
Ube4b	common	FALSE
Rhno1	common	FALSE
Osbpl11	common	FALSE
Usb1	common	FALSE
Rps11	common	FALSE
G6pdx	common	FALSE
Tspan18	common	FALSE
Bloc1s6	common	FALSE
Arl5a	common	FALSE
Ccny	common	FALSE
Bzw2	common	FALSE
Atoh8	common	TRUE
Nxf1	common	FALSE
Ints6	common	FALSE
Mplkip	common	FALSE
Ergic1	common	FALSE

Usp4	common	FALSE
Rnf44	common	FALSE
Fiz1	common	TRUE
Msl2	common	FALSE
Erbb2ip	common	FALSE
Igfbp4	common	FALSE
Usp21	common	FALSE
Lyplal1	common	FALSE
Sh3kbp1	common	FALSE
Fbxo22	common	FALSE
Irf2bp1	common	FALSE
Tgds	common	FALSE
Rad23a	common	FALSE
Bak1	common	FALSE
Edc4	common	FALSE
B4galt1	common	FALSE
Odf2	common	FALSE
Rassf7	common	FALSE
Gramd3	common	FALSE
Frmd8	common	FALSE
Tfip11	common	FALSE
Aagab	common	FALSE
Dnajc13	common	FALSE
Hoxb5	common	TRUE
Srgap2	common	FALSE
Arhgap17	common	FALSE
Adcy6	common	FALSE
Aggf1	common	TRUE
Rap2b	common	FALSE
Rad17	common	FALSE
Clcn5	common	FALSE
Aifm1	common	FALSE
Polr3k	common	FALSE
Srsf9	common	FALSE
Ppp2ca	common	FALSE
Rnf115	common	FALSE
Ppig	common	FALSE
1110059E2	common	FALSE
Ankle2	common	FALSE
Yme1l1	common	FALSE
Gm5801.1	common	FALSE
Oma1	common	FALSE
Nsmaf	common	FALSE
Dcaf17	common	FALSE
Pusl1	common	FALSE
Nqo2	common	FALSE
Htra2	common	FALSE
Bach2	common	TRUE
Vps8	common	FALSE
Igsf3	common	FALSE

Fam96a	common	FALSE
Ints12	common	FALSE
Map3k7	common	FALSE
Gtpbp1	common	TRUE
Copb1	common	FALSE
Mrpl16	common	FALSE
Zfp277	common	FALSE
Rcor2	common	FALSE
Atf7ip	common	FALSE
Hars	common	FALSE
Cbx3	common	TRUE
Cdc40	common	FALSE
Hscb	common	FALSE
Usp9x	common	FALSE
Trpm7	common	FALSE
Nfyc	common	TRUE
Pvrl2	common	FALSE
Tram1	common	FALSE
Tshz2	common	FALSE
Prpf6	common	FALSE
Dpp9	common	FALSE
Rnf130	common	FALSE
Gm5088	common	FALSE
Rad54l2	common	FALSE
Fndc4	common	FALSE
Hvcn1	common	FALSE
Fbxo38	common	FALSE
Idh2	common	FALSE
Klhl9	common	FALSE
Dbt	common	FALSE
Efna4	common	FALSE
Cecr5	common	FALSE
Adamts4	common	FALSE
Cln5	common	FALSE
Gxylt1	common	FALSE
Tacc1	common	FALSE
B230219D2	common	FALSE
Maea	common	FALSE
Cstf2t	common	FALSE
Spsb1	common	FALSE
Abhd13	common	FALSE
Rxra	common	TRUE
Nacc2	common	FALSE
Traf7	common	FALSE
Scaf8	common	FALSE
Sep-02	common	FALSE
Rprd1a	common	FALSE
Ublcp1	common	FALSE
Afg3l1	common	FALSE
Pole3	common	TRUE

Msl3	common	FALSE
Creb1	common	TRUE
Mphosph6	common	FALSE
1810030O(	common	FALSE
Kdm5c	common	FALSE
Agpat5	common	FALSE
Tpbg	common	FALSE
Utp11l	common	FALSE
Tlk2	common	FALSE
9430016H(	common	FALSE
Aasdhppt	common	FALSE
Pgs1	common	FALSE
Prkdc	common	FALSE
Zbtb2	common	TRUE
Mrpl10	common	FALSE
Prcp	common	FALSE
Mgat2	common	FALSE
Yes1	common	FALSE
F2r	common	FALSE
Dtx2	common	FALSE
Rasa2	common	FALSE
Ncaph2	common	FALSE
Cul5	common	FALSE
Camkk2	common	FALSE
Ppp2r5e	common	FALSE
Pik3r2	common	FALSE
Tbc1d14	common	FALSE
Cdk5rap2	common	FALSE
Srsf11	common	FALSE
Snx9	common	FALSE
Ap3m1	common	FALSE
Ireb2	common	FALSE
Kctd5	common	FALSE
Ddx6	common	FALSE
Wipi2	common	FALSE
Amer1	common	FALSE
Exo5	common	TRUE
Rpl31	common	FALSE
Crls1	common	FALSE
Spr	common	TRUE
Hibadh	common	FALSE
Sipa1	common	FALSE
Gle1	common	FALSE
Rpe	common	FALSE
Akr7a5	common	FALSE
Dock11	common	FALSE
Zfp598	common	TRUE
Dab1	common	FALSE
2700049A(	common	FALSE
Prpf38b	common	FALSE

Gprc5b	common	FALSE
Slc7a6os	common	FALSE
Gon4l	common	FALSE
Fam219b	common	FALSE
Mthfsd	common	FALSE
Coq2	common	FALSE
Ttc4	common	FALSE
Pank4	common	FALSE
Ipo13	common	FALSE
Rpl7a	common	FALSE
Dcps	common	FALSE
Clip1	common	FALSE
Ctage5	common	FALSE
Mettl14	common	TRUE
Cln6	common	FALSE
Mmd	common	FALSE
Ankrd52	common	FALSE
Sap130	common	FALSE
Rbm26	common	FALSE
Dzip3	common	FALSE
Zfp184	common	TRUE
Stk11ip	common	FALSE
Trp53bp1	common	FALSE
Tceal8	common	FALSE
Bag4	common	FALSE
Rnps1	common	FALSE
Poldip2	common	FALSE
Rpl11	common	FALSE
Poglut1	common	FALSE
Rpl30.1	common	FALSE
Dclre1a	common	FALSE
Zeb2	common	TRUE
Dcakd	common	FALSE
Oxa1l	common	FALSE
Smox	common	FALSE
B4galt2	common	FALSE
Mettl9	common	FALSE
Dmap1	common	TRUE
Rbm22	common	TRUE
Fam168b	common	FALSE
Glis2	common	TRUE
Rbm45	common	FALSE
Impa2	common	FALSE
Tmem251	common	FALSE
Slk	common	FALSE
Mfge8	common	FALSE
Timm44	common	TRUE
Ptdss2	common	FALSE
Sap30	common	TRUE
Anapc4	common	FALSE

Aebp2	common	TRUE
Fam86	common	FALSE
Zfyve19	common	FALSE
Sep-08	common	FALSE
Hlcs	common	TRUE
Gtf2h1	common	FALSE
Guf1	common	FALSE
Ascc2	common	FALSE
Cmc2	common	FALSE
Zfp553	common	TRUE
Dcun1d1	common	FALSE
Gm8615	common	FALSE
Cdkn2aip	common	FALSE
Sec24b	common	FALSE
Asap1	common	FALSE
Cep131	common	FALSE
Slc35g1	common	FALSE
Gtpbp3	common	FALSE
Hadh	common	FALSE
Ppm1d	common	FALSE
Sart1	common	FALSE
Zfp191	common	FALSE
Gnai1	common	FALSE
Hrsp12	common	FALSE
Minpp1	common	FALSE
BC017158	common	FALSE
Armc1	common	FALSE
Ttf1	common	FALSE
Ap2a1	common	FALSE
Iffo2	common	FALSE
Nudt8	common	FALSE
Pikfyve	common	FALSE
Gpalpp1	common	FALSE
Kansl3	common	FALSE
Dtd2	common	FALSE
Ncbp2	common	TRUE
Gpkow	common	FALSE
Ehd4	common	FALSE
Cdk9	common	FALSE
Pskh1	common	FALSE
Ubap1	common	FALSE
Mpi	common	FALSE
Yaf2	common	FALSE
Tmem18	common	FALSE
Zik1	common	TRUE
Frrs1	common	FALSE
Peli1	common	FALSE
Fmnl3	common	FALSE
D8Ertd82e	common	FALSE
4930453N2	common	FALSE

Hoxb4	common	TRUE
Tomm20	common	FALSE
Glod4	common	FALSE
Mdfi	common	FALSE
Gins4	common	FALSE
2210018M	common	FALSE
Rarb	common	TRUE
Vps54	common	FALSE
Sphk2	common	FALSE
Mrpl49	common	FALSE
Mlst8	common	FALSE
Bloc1s5	common	FALSE
Gorasp1	common	FALSE
Nme2	common	FALSE
Snx1	common	FALSE
Plekhg2	common	FALSE
Mrpl39	common	FALSE
Cwc27	common	FALSE
Birc6	common	FALSE
Zfp746	common	TRUE
Agpat6	common	FALSE
Gmeb1	common	TRUE
Pi4k2a	common	FALSE
Snora68	common	FALSE
Cog4	common	FALSE
C330007P0	common	FALSE
1500012F0	common	FALSE
E2f1	common	TRUE
Hjurp	common	FALSE
Myg1	common	FALSE
Crot	common	FALSE
Helz	common	FALSE
2810468N0	common	FALSE
Zbtb17	common	TRUE
Nemf	common	FALSE
Kansl2	common	FALSE
Smarcal1	common	FALSE
Adam17	common	FALSE
Rab8b	common	FALSE
Med23	common	FALSE
Ccdc132	common	FALSE
Mrpl2	common	TRUE
Ormdl2	common	FALSE
Taf8	common	FALSE
Hsp90b1	common	FALSE
Rpp25l	common	FALSE
Prrc1	common	FALSE
Prpf19	common	FALSE
Nckap1	common	FALSE
4930452G1	common	FALSE

Slc25a3	common	FALSE
Clcc1	common	FALSE
Zc3h14	common	FALSE
Impa1	common	FALSE
Tdrd3	common	FALSE
Mtor	common	FALSE
Commd8	common	FALSE
Nprl3	common	FALSE
Cept1	common	FALSE
Twf2	common	FALSE
Lzts2	common	FALSE
Rnf220	common	FALSE
Mrpl35	common	FALSE
Hiatl1	common	FALSE
Tmem2	common	FALSE
Mtpap	common	FALSE
Bcor	common	FALSE
Hddc2	common	FALSE
Dnajc1	common	FALSE
Asph	common	FALSE
Rad21	common	TRUE
Timm21	common	FALSE
Ankhd1	common	FALSE
Lrrc41	common	FALSE
Gps1	common	FALSE
Itsn1	common	FALSE
Pgm3	common	FALSE
Asap2	common	FALSE
Zfml	common	FALSE
Zfyve16	common	FALSE
Cyba	common	FALSE
Ppp1cc	common	FALSE
Gpc1	common	FALSE
Mtx1	common	FALSE
Msantd2	common	FALSE
Crocc	common	FALSE
Ssbp1	common	FALSE
BC030336	common	FALSE
Mtx2	common	FALSE
Mical3	common	FALSE
Ccdc102a	common	FALSE
Rbm7	common	TRUE
Spg11	common	FALSE
Kat6a	common	FALSE
Dyrk1a	common	FALSE
Wwp2	common	FALSE
Cisd2	common	FALSE
Tln1	common	FALSE
Atad2b	common	FALSE
Pag1	common	FALSE

Fam171a1	common	FALSE
Preb	common	FALSE
Sox5	common	TRUE
Gna13	common	FALSE
Rbm34	common	FALSE
Tbc1d15	common	FALSE
Ipo8	common	FALSE
B630005N1	common	FALSE
Mat2b	common	FALSE
Atpaf1	common	FALSE
Pik3r4	common	FALSE
Smurf2	common	FALSE
Tmem201	common	FALSE
Trps1	common	TRUE
Zbtb26	common	TRUE
Rrp36	common	FALSE
Lmtk2	common	FALSE
Atp5a1	common	FALSE
Tfg	common	FALSE
Mcfd2	common	FALSE
Cox11	common	FALSE
Tsen34	common	FALSE
Pnrc2	common	FALSE
Ccnd3	common	FALSE
Myc	common	TRUE
Mrpl37	common	FALSE
Wdr45b	common	FALSE
Sh3bp1	common	FALSE
Acn9	common	FALSE
Gpc6	common	FALSE
Cdc123	common	FALSE
Snupn	common	FALSE
Rnf20	common	FALSE
Haus8	common	FALSE
Zfp219	common	TRUE
Hmgb1	common	TRUE
Adck5	common	FALSE
Ret	common	FALSE
Fbxo21	common	FALSE
Clpx	common	FALSE
0610030E2	common	FALSE
Miip	common	FALSE
Ube2l3	common	FALSE
Fra10ac1	common	FALSE
Zfp318	common	FALSE
Ccar2	common	FALSE
Itprip	common	FALSE
AU019823	common	FALSE
Chtop	common	FALSE
Arfgap2	common	FALSE

Hdac6	common	TRUE
Oxsr1	common	FALSE
Tubgcp6	common	FALSE
Emc1	common	FALSE
D11Wsu47	common	FALSE
Taf11	common	FALSE
Mterfd2	common	FALSE
Ep400	common	FALSE
Scube3	common	FALSE
Asb3	common	FALSE
Tor1aip1	common	FALSE
Ccdc127	common	FALSE
Macf1	common	FALSE
Slc35b4	common	FALSE
Gmpr2	common	FALSE
Mrpl9	common	FALSE
Mri1	common	FALSE
Psma6	common	TRUE
Litaf	common	FALSE
Zdhhc18	common	FALSE
Snrpe	common	FALSE
Csl	common	FALSE
Sipa1l1	common	FALSE
Dhx30	common	FALSE
Hs1bp3	common	FALSE
Mapkap1	common	FALSE
Zfand5	common	FALSE
Lsm6	common	TRUE
Six5	common	TRUE
Rnf114	common	TRUE
Nek9	common	FALSE
Pex2	common	FALSE
Ttpal	common	FALSE
Golph3	common	FALSE
Tmem131	common	FALSE
Pml	common	TRUE
Snx27	common	FALSE
Zfp609	common	FALSE
Map2k4	common	FALSE
Zfp488	common	FALSE
C430049B0	common	FALSE
Ganab	common	FALSE
Copz1	common	FALSE
Zbed6	common	TRUE
Brcc3	common	FALSE
Twf1	common	FALSE
Usp45	common	FALSE
Fam118b	common	FALSE
Palld	common	FALSE
Tle3	common	FALSE

Mar-05	common	FALSE
Dxo	common	FALSE
Smad5	common	TRUE
Purb	common	FALSE
Map2k1	common	FALSE
Rcn2	common	FALSE
Samm50	common	FALSE
Dnajc19	common	FALSE
Phkb	common	FALSE
Pofut1	common	FALSE
Dpy30	common	FALSE
Fbxo34	common	FALSE
Eps15l1	common	FALSE
Xylt2	common	FALSE
R3hcc1l	common	FALSE
1700020I1	common	FALSE
Atrip	common	FALSE
Tceb3	common	FALSE
Rpl29	common	FALSE
Fam49a	common	FALSE
Mccc1	common	FALSE
Tsc22d2	common	FALSE
Ap3b1	common	FALSE
Cdk17	common	FALSE
Kdelc2	common	FALSE
Mb21d2	common	FALSE
Sesn2	common	FALSE
Zkscan8	common	TRUE
Pter	common	FALSE
Wibg	common	FALSE
Mmab	common	FALSE
Dap3	common	FALSE
Pgap2	common	FALSE
Ints4	common	FALSE
Scrib	common	FALSE
Mtrf1l	common	FALSE
2700029M	common	FALSE
Mrpl11	common	FALSE
Pex6	common	FALSE
Flnb	common	FALSE
Vps4b	common	TRUE
Wdr33	common	FALSE
Gpatch2	common	FALSE
Glrx5	common	FALSE
Tcea1	common	FALSE
BC023829	common	FALSE
Csnk1g2	common	FALSE
Mrpl18	common	FALSE
Cap1	common	FALSE
Gnai3	common	FALSE

Bola3	common	FALSE
Phactr4	common	FALSE
Fkbp10	common	FALSE
Pcdhgc3	common	FALSE
Sfxn5	common	FALSE
Hnrnpl	common	FALSE
Pros1	common	FALSE
Tubgcp5	common	FALSE
Thumpd2	common	FALSE
Slc25a36	common	FALSE
Invs	common	FALSE
Phf23	common	FALSE
Lats2	common	FALSE
H2afy3	common	FALSE
Aarsd1	common	FALSE
Mllt10	common	FALSE
Zfp871	common	FALSE
Xpnpep1	common	FALSE
Ndnl2	common	FALSE
Rnf169	common	FALSE
Mier2	common	FALSE
Kat8	common	FALSE
Slc25a10	common	FALSE
Nnt	common	TRUE
Gm17066	common	FALSE
Erlin2	common	FALSE
Sec14l1	common	FALSE
Ccdc22	common	FALSE
Pttg1	common	FALSE
Snn	common	FALSE
Mcts2	common	FALSE
Plekho2	common	FALSE
AU022252	common	FALSE
Copb2	common	FALSE
Pibf1	common	FALSE
Eif4e2	common	FALSE
Syngr2	common	FALSE
Prdm2	common	FALSE
Dis3l	common	FALSE
Zscan21	common	TRUE
Pak2	common	FALSE
C2cd3	common	FALSE
Kdm3a	common	FALSE
Slmo2	common	FALSE
Plekhg3	common	FALSE
Rapgef1	common	FALSE
Pex13	common	FALSE
Slc38a1	common	FALSE
Cdc26	common	FALSE
Mbtd1	common	FALSE

Tsta3	common	FALSE
Exoc3	common	FALSE
Ankrd11	common	FALSE
Pcbp2	common	FALSE
Limd1	common	FALSE
Rpl6	common	TRUE
Supt20	common	TRUE
Snx24	common	FALSE
Kbtbd2	common	FALSE
Rps6kb1	common	FALSE
Tcf19	common	FALSE
Alg10b	common	FALSE
Rwdd1	common	FALSE
Nlk	common	FALSE
Zfp36l1	common	FALSE
Rnaseh2b	common	FALSE
Nfe2l1	common	TRUE
Zfp69	common	TRUE
Atf6b	common	TRUE
Gm1943	common	FALSE
Mrpl36	common	FALSE
Rab28	common	FALSE
Trnau1ap	common	FALSE
Rpgrip1l	common	FALSE
0610040J0	common	FALSE
Dgkz	common	FALSE
Ap1b1	common	FALSE
Zfp281	common	TRUE
Med7	common	FALSE
Kctd3	common	FALSE
Pced1a	common	FALSE
Ogfr	common	FALSE
Mepce	common	FALSE
Pcnp	common	FALSE
Cdk5rap3	common	FALSE
Cpne2	common	FALSE
Krit1	common	FALSE
Lphn2	common	FALSE
Srp72	common	FALSE
Wdr82	common	FALSE
Fam173b	common	FALSE
Fam53c	common	FALSE
Zfp687	common	TRUE
Lpgat1	common	FALSE
Garem	common	FALSE
Plod2	common	FALSE
Ehd1	common	FALSE
Smyd2	common	FALSE
Mbtps1	common	FALSE
Ski	common	FALSE

Pomt1	common	FALSE
Ube3b	common	FALSE
Tmed1	common	FALSE
Fzd3	common	FALSE
Mad2l1bp	common	FALSE
Frmd4a	common	FALSE
Rprd2	common	FALSE
Traf3	common	FALSE
Ppil4	common	FALSE
Fbxo28	common	FALSE
Psmc3	common	FALSE
Zscan29	common	TRUE
Kbtbd4	common	FALSE
Gng5	common	FALSE
Arap1	common	FALSE
Zfp330	common	FALSE
Sh3tc2	common	FALSE
Inppl1	common	FALSE
Banp	common	TRUE
Atg4c	common	FALSE
Psph	common	FALSE
Hoxd9	common	TRUE
Rfxap	common	TRUE
Auts2	common	FALSE
Sec61a1	common	FALSE
Tor1b	common	FALSE
Rell1	common	FALSE
Lbh	common	FALSE
P4hb	common	TRUE
Nme1	common	TRUE
Zfp384	common	FALSE
Hnrnpa0	common	TRUE
Dhrs4	common	FALSE
Tspan6	common	FALSE
AW549877	common	FALSE
Prr14l	common	FALSE
Homer2	common	FALSE
Cdr2l	common	FALSE
Dgkd	common	FALSE
Oser1	common	FALSE
Gnpda1	common	FALSE
Spata2	common	FALSE
Ethe1	common	FALSE
Cdc42se1	common	FALSE
Ylpm1	common	FALSE
Npl	common	FALSE
M6pr	common	FALSE
Ptpn13	common	FALSE
Zfp111	common	TRUE
Angel2	common	FALSE

Specc1l	common	FALSE
Zfp692	common	TRUE
Smc1a	common	FALSE
Pogz	common	FALSE
Trim33	common	TRUE
Ncoa3	common	FALSE
Prdx3	common	FALSE
Mrpl22	common	FALSE
Slc30a6	common	FALSE
Vars2	common	FALSE
Ndufs2	common	FALSE
Snrpa	common	FALSE
Crtc2	common	TRUE
Nelfb	common	TRUE
Pdcl	common	FALSE
Lhfpl2	common	FALSE
Meaf6	common	FALSE
Lpp	common	FALSE
Hectd1	common	FALSE
Ankrd28	common	FALSE
Vps26a	common	FALSE
Bambi	common	FALSE
Sugt1	common	FALSE
Mar-07	common	FALSE
Naa60	common	FALSE
Ube2q1	common	FALSE
Rbm33	common	FALSE
Dmtf1	common	FALSE
Tm9sf2	common	FALSE
Sumo1	common	FALSE
Ankrd10	common	FALSE
Rpl8	common	FALSE
Med19	common	FALSE
Psma4	common	FALSE
Setd1a	common	FALSE
Dcun1d5	common	FALSE
Zfp212	common	TRUE
Zfp639	common	TRUE
Ncoa5	common	FALSE
Sh3gl1	common	FALSE
Gskip	common	FALSE
Igf2r	common	FALSE
Slc35c2	common	FALSE
Phf12	common	FALSE
Ccdc61	common	FALSE
Smad4	common	TRUE
Snrnp48	common	FALSE
Taf10	common	FALSE
Ptpn11	common	FALSE
Hnrnpul2	common	FALSE

Top3b	common	FALSE
Fgfr1op2	common	FALSE
Fam175b	common	FALSE
Cd2bp2	common	FALSE
Rspry1	common	FALSE
Gsr	common	FALSE
Rhbdf1	common	FALSE
Ubl3	common	FALSE
Egln1	common	FALSE
Vdac3	common	FALSE
Btf3	common	FALSE
Setd2	common	FALSE
H19	common	FALSE
Mbd1	common	TRUE
Brd3	common	FALSE
Gm53	common	FALSE
Rgl1	common	FALSE
Samd4b	common	FALSE
Dusp22	common	TRUE
Acp6	common	FALSE
Mgst1	common	FALSE
5430416N(	common	FALSE
Csrnp2	common	FALSE
Usp34	common	FALSE
Rps19bp1	common	FALSE
Fntb	common	FALSE
Gm5434	common	FALSE
Lias	common	FALSE
Rbbp6	common	FALSE
Zfp354c	common	TRUE
Rnf185	common	FALSE
Ogg1	common	FALSE
Zmym4	common	FALSE
Clpb	common	FALSE
Tiam1	common	FALSE
Mrpl13	common	FALSE
Atxn1l	common	FALSE
Serpinh1	common	FALSE
Acap2	common	FALSE
Zmym2	common	FALSE
Zfp740	common	TRUE
Rps8	common	FALSE
Midn	common	FALSE
Brpf3	common	FALSE
Dcaf12	common	FALSE
Hax1	common	FALSE
Abhd17a	common	FALSE
Gldc	common	FALSE
Dnm1l	common	FALSE
Rttn	common	FALSE

Cfdp1	common	FALSE
Ahcyl1	common	FALSE
Fbxw11	common	FALSE
B4galt7	common	FALSE
Sltm	common	FALSE
Nr2c1	common	TRUE
Upf2	common	FALSE
Chst11	common	FALSE
Ggta1	common	FALSE
Mterfd1	common	FALSE
Ubac2	common	FALSE
Sec24c	common	FALSE
Mtmr12	common	FALSE
Moxd1	common	FALSE
Uros	common	FALSE
Ttc21b	common	FALSE
Rfwd2	common	FALSE
Trim7	common	FALSE
Nsd1	common	FALSE
Clk2	common	FALSE
Pex14	common	FALSE
Mfsd5	common	FALSE
Pnkp	common	FALSE
Hoxa2	common	TRUE
Rbm12b2	common	FALSE
1110037F0	common	FALSE
Tmem126a	common	FALSE
Kdm5a	common	TRUE
Rmnd5a	common	FALSE
Lage3	common	FALSE
Pcgf2	common	FALSE
Sf3b2	common	FALSE
Rfk	common	FALSE
Stk38	common	FALSE
4930594C1	common	FALSE
Nsmce2	common	FALSE
Ctnnd1	common	FALSE
Arfrp1	common	FALSE
Necap2	common	FALSE
Chd8	common	FALSE
Fbf1	common	FALSE
Gtf2f1	common	TRUE
Coa4	common	FALSE
Rac1	common	FALSE
2310003H(	common	FALSE
Id2	common	TRUE
Rrbp1	common	FALSE
Scyl3	common	FALSE
Mcl1	common	FALSE
Fkbp9	common	FALSE

Gigyf2	common	FALSE
Coq6	common	FALSE
Atxn2	common	FALSE
Hoxa4	common	TRUE
Gm5512.1	common	FALSE
Ssh1	common	FALSE
Chst3	common	FALSE
Xpc	common	FALSE
Tmem231	common	FALSE
Akt2	common	FALSE
Slc35e2	common	FALSE
Dlx2	common	TRUE
Mysm1	common	FALSE
2410002F2	common	FALSE
Wdfy1	common	FALSE
Phf20	common	FALSE
Shprh	common	FALSE
Echdc1	common	FALSE
Cog8	common	FALSE
Dnajb12	common	FALSE
Wnk4	common	FALSE
Rft1	common	FALSE
Ewsr1	common	TRUE
Gid8	common	FALSE
Smim13	common	FALSE
Med20	common	FALSE
Rplp1	common	FALSE
Apopt1	common	FALSE
Bcl9	common	FALSE
Peak1	common	FALSE
Insc	common	FALSE
Sepp1	common	FALSE
Mettl10	common	FALSE
Srprb	common	FALSE
Dcaf10	common	FALSE
Ap3s2	common	FALSE
Mtdh	common	FALSE
Naa35	common	FALSE
0610009O2	common	FALSE
Zfp36l2	common	FALSE
Ephb2	common	FALSE
Uba1	common	FALSE
Eefsec	common	FALSE
Igbp1	common	FALSE
Micu2	common	FALSE
Brd1	common	FALSE
Npepps	common	FALSE
Dgcr14	common	FALSE
Man1b1	common	FALSE
Strn	common	FALSE

Cdk10	common	FALSE
Otud7b	common	FALSE
D3Ertd254	common	TRUE
Pts	common	FALSE
Fggy	common	FALSE
Rnf121	common	FALSE
Chd4	common	FALSE
BC003965	common	FALSE
Tmed7	common	FALSE
Rxrb	common	TRUE
Xndc1	common	FALSE
Lepre1	common	FALSE
Fbxl5	common	FALSE
Lrrc42	common	FALSE
Polg	common	FALSE
Arfip1	common	FALSE
Gusb	common	FALSE
Efs	common	FALSE
Ddx50	common	FALSE
Depdc5	common	FALSE
Med24	common	FALSE
Tmem138	common	FALSE
2810055G2	common	FALSE
Tssc4	common	FALSE
Rasa1	common	FALSE
Arnt	common	TRUE
Fzr1	common	FALSE
Gtf2ird1	common	TRUE
Cnpy4	common	FALSE
Mkl2	common	FALSE
Tbc1d7	common	FALSE
Setd5	common	FALSE
H2afy	common	TRUE
4933426M	common	FALSE
Bag5	common	FALSE
Glrx3	common	FALSE
Dusp11	common	FALSE
Sdhd	common	FALSE
Cchcr1	common	FALSE
Ndufab1	common	FALSE
Mtmr10	common	FALSE
5830417I1	common	FALSE
Gcdh	common	FALSE
Scarb1	common	FALSE
Slc39a9	common	FALSE
Ttc13	common	FALSE
Snrpf	common	FALSE
Herpud2	common	FALSE
Stim2	common	FALSE
Cxxc5	common	FALSE

Nckap5l	common	FALSE
Trappc12	common	FALSE
Xiap	common	FALSE
Fbxo11	common	FALSE
Raph1	common	FALSE
Fbxl12	common	FALSE
Magohb	common	FALSE
Fam76b	common	FALSE
Lrp5	common	FALSE
Fam120a	common	FALSE
Tmem29	common	FALSE
Arhgef25	common	FALSE
Trappc5	common	FALSE
Tmem106c	common	FALSE
0610010K1	common	FALSE
Vps16	common	FALSE
Snapc2	common	FALSE
Rad52	common	FALSE
Irak1	common	FALSE
Arap3	common	FALSE
Zfp362	common	TRUE
Men1	common	FALSE
Gm6548	common	FALSE
Rnf139	common	FALSE
Zfp617	common	FALSE
Zfp768	common	TRUE
Ncoa6	common	FALSE
Dak	common	FALSE
2210016F1	common	FALSE
Galc	common	FALSE
Loxl3	common	FALSE
Arih1	common	FALSE
N6amt2	common	FALSE
Apip	common	FALSE
Gtf3c1	common	FALSE
Ryk	common	FALSE
Vcl	common	FALSE
Rpl18	common	FALSE
Rassf1	common	FALSE
Magt1	common	FALSE
R3hdm1	common	FALSE
Rpl17	common	FALSE
Phc2	common	FALSE
Supt6	common	FALSE
Tox4	common	FALSE
Zcchc11	common	FALSE
Eny2	common	FALSE
Wtap	common	FALSE
Rnf111	common	FALSE
Zfp410	common	TRUE

Nrd1	common	FALSE
Dcaf8	common	FALSE
Slc7a6	common	FALSE
Plin3	common	FALSE
Copg2	common	FALSE
Lta4h	common	FALSE
Mrgbp	common	FALSE
Zdhhc20	common	FALSE
Ankrd17	common	FALSE
Cdk11b	common	FALSE
Rps4x	common	TRUE
Arid1a	common	FALSE
Eif4ebp3	common	FALSE
Mrpl17	common	FALSE
Mms19	common	FALSE
Map2k5	common	FALSE
Trmt1l	common	FALSE
Hnrnpa3	common	FALSE
Lmf1	common	FALSE
Sec16a	common	FALSE
Apba3	common	FALSE
Extl2	common	FALSE
Zfp780b	common	TRUE
Cab39	common	FALSE
Sorbs3	common	FALSE
Paip2b	common	FALSE
Zbed3	common	FALSE
Rpl14-ps1	common	FALSE
Fam92a	common	FALSE
Kansl1	common	FALSE
Cyp20a1	common	FALSE
Ccdc9	common	FALSE
Rtca	common	FALSE
Pip5k1a	common	FALSE
Babam1	common	FALSE
Rnf216	common	FALSE
Mark3	common	FALSE
Hmox2	common	FALSE
Dlg3	common	FALSE
Cul7	common	FALSE
Trip12	common	FALSE
Zfp91	common	TRUE
Rab3ip	common	FALSE
Trove2	common	TRUE
Supt7l	common	FALSE
2310067B1	common	FALSE
Pmpcb	common	FALSE
Zfp426	common	TRUE
Ube2d2a	common	FALSE
Armc5	common	FALSE

Plekhh1	common	FALSE
Phtf1	common	TRUE
A830082K1	common	FALSE
Kdm3b	common	FALSE
Ammecr1l	common	FALSE
Dido1	common	FALSE
Phf14	common	FALSE
Krcc1	common	FALSE
Fam73b	common	FALSE
Tmem263	common	FALSE
Pdia6	common	FALSE
Capza2	common	FALSE
Ciz1	common	FALSE
Dot1l	common	FALSE
Tmem68	common	FALSE
Pias2	common	FALSE
Scyl1	common	FALSE
Socs5	common	FALSE
1700123O2	common	FALSE
Fbln2	common	FALSE
Ccnc	common	FALSE
Rpl14	common	FALSE
Tfdp2	common	TRUE
Scfd1	common	FALSE
Brwd1	common	FALSE
Fam109a	common	FALSE
Ambra1	common	FALSE
Rad51b	common	FALSE
Cpd	common	FALSE
Mmadhc	common	FALSE
Slc45a4	common	FALSE
Cnp	common	FALSE
Caskin2	common	FALSE
Vopp1	common	FALSE
Slc30a7	common	FALSE
Zfp618	common	FALSE
Rhod	common	FALSE
Ubr1	common	FALSE
Sdc3	common	FALSE
Ccdc50	common	FALSE
Supt5	common	FALSE
Ubr2	common	FALSE
Phxr4	common	FALSE
Zfp608	common	FALSE
Nit1	common	FALSE
Jag1	common	FALSE
Abi1	common	FALSE
Tmem260	common	FALSE
Med12	common	FALSE
Cc2d1b	common	FALSE

Chuk	common	FALSE
Pign	common	FALSE
Haus7	common	FALSE
Suco	common	FALSE
Hmces	common	FALSE
Pkd2	common	FALSE
Pofut2	common	FALSE
Tnfaip1	common	FALSE
Phip	common	FALSE
Fam58b	common	FALSE
Malsu1	common	FALSE
Sbno1	common	FALSE
Luc7l	common	FALSE
Vrk3	common	FALSE
Tial1	common	FALSE
Mkks	common	FALSE
Ppip5k2	common	FALSE
Ppp2r3c	common	FALSE
Cdon	common	FALSE
Ppifos	common	FALSE
Pigh	common	FALSE
Hist1h4i	common	FALSE
Gatad2b	common	FALSE
Rps3	common	FALSE
Nudt14	common	FALSE
Tpst2	common	FALSE
Ube2j2	common	FALSE
Sh3bgrl	common	FALSE
Smarce1	common	FALSE
Mboat7	common	FALSE
Zfp397	common	TRUE
Exosc4	common	FALSE
Ighmbp2	common	FALSE
Uggt2	common	FALSE
1700021F0	common	FALSE
Terf2	common	TRUE
Stxbp2	common	FALSE
Etl4	common	FALSE
Ppp1ca	common	FALSE
Mfsd10	common	FALSE
1810013L2	common	FALSE
Ppcs	common	FALSE
Pcnxl3	common	FALSE
Mapkapk5	common	FALSE
Aaed1	common	FALSE
Lsm14b	common	FALSE
Chid1	common	FALSE
Mrpl42	common	FALSE
Wbp1l	common	FALSE
Cpsf7	common	FALSE

Htatsf1	common	FALSE
Pyurf	common	FALSE
Cdk13	common	FALSE
Rpl22l1	common	FALSE
Cmtm5	common	FALSE
Trim24	common	TRUE
Prorsd1	common	FALSE
Natd1	common	FALSE
Camk2b	common	FALSE
Smarca4	common	TRUE
Acy3	common	FALSE
Klhl26	common	FALSE
Tmem185b	common	FALSE
2610020H(	common	FALSE
Fto	common	FALSE
Fam73a	common	FALSE
Map4k4	common	FALSE
Srek1	common	FALSE
Plod1	common	FALSE
Zfp157	common	FALSE
Map3k1	common	FALSE
Zc3h13	common	FALSE
Pdia4	common	FALSE
Arpc1b	common	FALSE
Pisd	common	FALSE
Cramp1l	common	FALSE
Smtn	common	FALSE
0610007P1	common	FALSE
St6galnac3	common	FALSE
Snx19	common	FALSE
Tmem161a	common	FALSE
Atmin	common	FALSE
Ppp1r18	common	FALSE
Ywhae	common	TRUE
Slc26a2	common	FALSE
Lnx2	common	FALSE
1810026J2	common	FALSE
Akirin2	common	FALSE
Armc10	common	FALSE
Zbtb34	common	TRUE
Cnnm3	common	FALSE
Coasy	common	FALSE
Lrfn4	common	FALSE
Ccdc71	common	FALSE
Shoc2	common	FALSE
Aamp	common	FALSE
Myl12a	common	FALSE
Hmgcl	common	FALSE
Ccdc66	common	FALSE
Rock2	common	FALSE

Snx4	common	FALSE
Abcb8	common	FALSE
Slc37a3	common	FALSE
Zfp445	common	TRUE
Bach1	common	TRUE
Zfp326	common	TRUE
Fcf1	common	FALSE
Fyttd1	common	FALSE
Zbtb18	common	TRUE
2500004C0	common	FALSE
Ndufv1	common	FALSE
Uck1	common	FALSE
Mrpl28	common	FALSE
Cxxc1	common	TRUE
Btbd3	common	FALSE
Pygo2	common	FALSE
Stk11	common	FALSE
Rexo1	common	FALSE
Ift140	common	FALSE
Rpl23	common	FALSE
Med29	common	FALSE
Itfg3	common	FALSE
Znrd1	common	FALSE
Zgpat	common	FALSE
Tm9sf4	common	FALSE
Nans	common	FALSE
Cep164	common	FALSE
Bub3	common	FALSE
Tab1	common	FALSE
Ccnyl1	common	FALSE
Trip4	common	FALSE
Ttc1	common	FALSE
Entpd7	common	FALSE
Zfp771	common	TRUE
Pomk	common	FALSE
Sumf2	common	FALSE
Senp6	common	FALSE
Spata6	common	FALSE
Mgat1	common	FALSE
G6pd2	common	FALSE
Bphl	common	FALSE
Dennd6a	common	FALSE
D17H6S53E	common	FALSE
Tmem168	common	FALSE
Src	common	FALSE
Kbtbd11	common	FALSE
Kazn	common	FALSE
Lima1	common	FALSE
Fndc3a	common	FALSE
Pkig	common	FALSE

Myo1b	common	FALSE
Ccdc55	common	FALSE
Tor1aip2	common	FALSE
Epb4.1l2	common	FALSE
Mtif2	common	FALSE
Cdk2ap1	common	TRUE
Ube2z	common	FALSE
Sdsl	common	FALSE
4933411K2	common	FALSE
Tbc1d2b	common	FALSE
Efcab14	common	FALSE
Synm	common	FALSE
Hivep1	common	TRUE
2610301B2	common	FALSE
Rbpj	common	TRUE
Rgp1	common	FALSE
Vps72	common	TRUE
Mtch2	common	FALSE
Rpl10a	common	FALSE
Tmem184b	common	FALSE
Inpp5b	common	FALSE
Gclm	common	FALSE
Cbfb	common	TRUE
Fbxo46	common	FALSE
Prr3	common	FALSE
Vmp1	common	FALSE
Ngdn	common	FALSE
Nipbl	common	FALSE
Psmf1	common	FALSE
Map3k3	common	FALSE
Col18a1	common	FALSE
Top2b	common	FALSE
Fuk	common	FALSE
Rhoq	common	FALSE
Zfat	common	FALSE
Cog6	common	FALSE
2410006H1	common	FALSE
Epm2aip1	common	FALSE
Zcchc9	common	FALSE
Klhl5	common	FALSE
Sptlc1	common	FALSE
Snrpg	common	FALSE
Trpv4	common	FALSE
B4galt6	common	FALSE
C030046E1	common	FALSE
Nfat5	common	TRUE
Tab2	common	FALSE
Dmxl1	common	FALSE
Fam3c	common	FALSE
Pdcd2l	common	FALSE

Wrn	common	FALSE
Fam133b	common	FALSE
Srek1ip1	common	FALSE
N4bp2l2	common	FALSE
Filip1l	common	FALSE
Arf3	common	FALSE
Mynn	common	TRUE
Zfp160	common	TRUE
Tbc1d16	common	FALSE
Ndor1	common	FALSE
Gorasp2	common	FALSE
Wasl	common	FALSE
Rps25	common	FALSE
Cd302	common	FALSE
Akap13	common	FALSE
Pex19	common	FALSE
Tab3	common	FALSE
Git1	common	FALSE
Cdc5l	common	TRUE
Scamp4	common	FALSE
Prkd1	common	FALSE
Ube2m	common	FALSE
Rtkn	common	FALSE
Man2c1	common	FALSE
Ift52	common	FALSE
Kremen1	common	FALSE
Tmem33	common	TRUE
Ppp1r16b	common	FALSE
Slc26a11	common	FALSE
Trim26	common	FALSE
Med15	common	FALSE
Nfkb1	common	TRUE
Mir7090	common	FALSE
Tstd3	common	FALSE
Gna12	common	FALSE
Camkv	common	FALSE
Apool	common	FALSE
Rpl36a	common	FALSE
Snx2	common	FALSE
Nlgn3	common	FALSE
Nudt9	common	FALSE
Ctsk	common	FALSE
Mast2	common	FALSE
Mcu	common	FALSE
Mrpl4	common	FALSE
Ilkap	common	FALSE
Cnot6	common	TRUE
Tpm3	common	FALSE
Lacc1	common	FALSE
Tmem101	common	FALSE

Jam3	common	FALSE
Snx10	common	FALSE
Snx6	common	FALSE
Safb2	common	FALSE
Hilpda	common	FALSE
Paxbp1	common	FALSE
Pigu	common	FALSE
Rarg	common	TRUE
Wiz	common	FALSE
Srrd	common	FALSE
Golim4	common	FALSE
Gyg	common	FALSE
Asb7	common	FALSE
Sgsh	common	FALSE
Ccdc59	common	FALSE
Zfp606	common	TRUE
Ikbkg	common	FALSE
Naa20	common	FALSE
Gatad1	common	FALSE
AI846148	common	FALSE
Impad1	common	FALSE
Ccni	common	FALSE
Dolk	common	FALSE
Peg3	common	TRUE
Srp19	common	FALSE
Hoxd8	common	TRUE
Fam179b	common	FALSE
Chchd5	common	FALSE
Ppic	common	FALSE
Acads	common	FALSE
4931406CC	common	FALSE
Mtcp1	common	FALSE
Ing1	common	FALSE
Zfp62	common	TRUE
Fundc1	common	FALSE
Ptpn12	common	FALSE
Gypc	common	FALSE
Anapc10	common	FALSE
Efr3a	common	FALSE
Gm10069	common	FALSE
Pan2	common	FALSE
Frs2	common	FALSE
Ptx3	common	FALSE
Calu	common	FALSE
Tiprl	common	FALSE
Bet1	common	FALSE
Cant1	common	FALSE
Pla2g6	common	FALSE
Gpatch8	common	FALSE
Zfhx4	common	FALSE

Abracl	common	FALSE
Egflam	common	FALSE
4933407K1	common	FALSE
Cog3	common	FALSE
Skil	common	FALSE
Ythdc1	common	FALSE
Rps5	common	FALSE
Abcf3	common	FALSE
Usf2	common	TRUE
Pcyox1l	common	FALSE
Slc12a2	common	FALSE
Lman2	common	FALSE
Ubr3	common	FALSE
Dnttip1	common	TRUE
Morc4	common	FALSE
Sugp1	common	FALSE
Pard3	common	FALSE
Zfyve26	common	FALSE
Prex1	common	FALSE
Upf3b	common	FALSE
Ehd3	common	FALSE
Asrgl1	common	FALSE
Dgcr2	common	FALSE
Phc1	common	FALSE
Rap1b	common	FALSE
Magi1	common	FALSE
Med21	common	FALSE
Chmp6	common	FALSE
Actr2	common	FALSE
Fbxl15	common	FALSE
Bre	common	FALSE
Tceb1	common	FALSE
Hsd17b12	common	FALSE
Ubl4	common	FALSE
Ube2q2	common	FALSE
Plekho1	common	FALSE
Rps20	common	FALSE
Ghr	common	FALSE
Mfap1a	common	FALSE
Map4k3	common	FALSE
Rcor1	common	TRUE
Zcchc17	common	TRUE
Rab9	common	FALSE
Cry2	common	FALSE
Wnk1	common	FALSE
Slc25a24	common	FALSE
Edem3	common	FALSE
Elf2	common	TRUE
Hhat	common	TRUE
Skiv2l	common	FALSE

Rilpl2	common	FALSE
Wac	common	FALSE
Myo5a	common	FALSE
Kxd1	common	FALSE
Uaca	common	FALSE
Ano1	common	FALSE
Phax	common	FALSE
Vat1l	common	FALSE
Ptgr1	common	FALSE
Cntrob	common	FALSE
Tnfrsf1a	common	FALSE
Akna	common	FALSE
Misp	common	FALSE
Flii	common	FALSE
Aldh5a1	common	FALSE
Ccnjl	common	FALSE
Slc27a6	common	FALSE
Ranbp9	common	FALSE
Gpaa1	common	FALSE
2610034B1	common	FALSE
Sec63	common	FALSE
Tor2a	common	FALSE
Kctd10	common	FALSE
Puf60	common	FALSE
Faf2	common	FALSE
Cyb5r3	common	FALSE
Neto2	common	FALSE
Tle4	common	FALSE
D930015E(	common	FALSE
Tpd52l2	common	FALSE
Ccnt2	common	TRUE
Mvb12a	common	FALSE
Mbnl1	common	FALSE
Tsc2	common	FALSE
Rbm4b	common	FALSE
Gramd4	common	FALSE
Dhdds	common	FALSE
As3mt	common	FALSE
Rab26os	common	FALSE
Tmf1	common	FALSE
Gm15421	common	FALSE
Ptrhd1	common	FALSE
St6galnac4	common	FALSE
Mrps15	common	FALSE
Maz	common	TRUE
4921524J1	common	FALSE
Nek7	common	FALSE
Tmem129	common	FALSE
Rbm41	common	FALSE
Rbm25	common	FALSE

Paf1	common	FALSE
Golph3l	common	FALSE
Pex11b	common	FALSE
Dusp6	common	FALSE
Rnf10	common	FALSE
Mppe1	common	FALSE
Tm9sf1	common	FALSE
Crebzf	common	TRUE
Ergic2	common	FALSE
Endov	common	FALSE
Trim8	common	FALSE
Ercc6	common	FALSE
Zfp148	common	TRUE
Rps17	common	FALSE
Ppp6r1	common	FALSE
Ube2a	common	FALSE
Lamb1	common	FALSE
Nck2	common	FALSE
Fam222b	common	FALSE
Cpne3	common	FALSE
Pdxdc1	common	FALSE
Golga7	common	FALSE
Snhg8	common	FALSE
Cnot4	common	TRUE
1810014B0	common	FALSE
Elmo2	common	FALSE
Mrpl21	common	FALSE
Traf4	common	TRUE
Rfx3	common	TRUE
Numa1	common	FALSE
Uqcrc2	common	FALSE
Pon2	common	FALSE
Usp16	common	FALSE
Uso1	common	FALSE
Smad2	common	TRUE
Enc1	common	FALSE
Zfp623	common	TRUE
Sec22a	common	FALSE
B9d1	common	FALSE
Itgb1bp1	common	FALSE
Ralb	common	FALSE
Ost4	common	FALSE
Tceanc2	common	FALSE
Stau1	common	FALSE
Kctd1	common	FALSE
Siah1a	common	FALSE
5530601H0	common	FALSE
Btbd2	common	FALSE
Chm	common	FALSE
Notch1	common	FALSE

Ndst2	common	FALSE
Rhog	common	FALSE
Smurf1	common	FALSE
Mnat1	common	FALSE
Pomgnt1	common	FALSE
Spg7	common	FALSE
Trappc11	common	FALSE
Bod1	common	FALSE
LOC10674(	common	FALSE
Ctbp1	common	TRUE
Map4k5	common	FALSE
Elk3	common	TRUE
Fbxl19	common	TRUE
Sgpl1	common	FALSE
Slc43a3	common	FALSE
Bag3	common	FALSE
Ube2e1	common	FALSE
Nsun4	common	FALSE
Ebpl	common	FALSE
Fam104a	common	FALSE
Hspa13	common	FALSE
Mapk1	common	TRUE
Mtss1l	common	FALSE
Inpp1	common	FALSE
Map1s	common	FALSE
Skp1a	common	FALSE
Igdcc4	common	FALSE
Sox12	common	TRUE
Rpain	common	FALSE
Commd5	common	FALSE
Hsd17b10	common	FALSE
St3gal4	common	FALSE
Zbtb8os	common	FALSE
Asxl2	common	FALSE
Gna11	common	FALSE
Emd	common	FALSE
Lamc1	common	FALSE
Zfand1	common	FALSE
Fhod1	common	FALSE
Sft2d2	common	FALSE
Ncoa2	common	FALSE
Dnajc30	common	FALSE
Cenpb	common	TRUE
Rpn1	common	FALSE
2310009B1	common	FALSE
Pddc1	common	FALSE
Pcdh1	common	FALSE
Fam126a	common	FALSE
Copz2	common	FALSE
Mmgt2	common	FALSE

Pdcd5	common	FALSE
Cytl1	common	FALSE
Lsm5	common	FALSE
Copg1	common	FALSE
Psma2	common	FALSE
Mthfd2l	common	FALSE
Tmem5	common	FALSE
Svil	common	FALSE
Rfxank	common	TRUE
Timm10b	common	FALSE
Rai1	common	FALSE
Mbd3	common	FALSE
Ehmt2	common	FALSE
Pex7	common	FALSE
BC005561	common	TRUE
Adamts1	common	FALSE
Tfcp2	common	TRUE
Etv3	common	TRUE
Sap30bp	common	FALSE
Gcc2	common	FALSE
Trmu	common	FALSE
Hps5	common	FALSE
Acbd3	common	FALSE
Prkag1	common	FALSE
Fxyd3	common	FALSE
Kdm2a	common	TRUE
Fam122a	common	FALSE
Rnf38	common	FALSE
Zfp664	common	TRUE
Rtf1	common	FALSE
Stard5	common	FALSE
Atp5f1	common	FALSE
Ppp2r1a	common	FALSE
0610009B2	common	FALSE
Suds3	common	FALSE
Zkscan1	common	TRUE
Rps27l	common	FALSE
Akt1	common	FALSE
Tsr3	common	FALSE
Fbxo44	common	FALSE
Kdm1b	common	FALSE
Sepn1	common	FALSE
Ifnar1	common	FALSE
Csnk1d	common	FALSE
Adam10	common	FALSE
Hdac2	common	TRUE
Rp2h	common	FALSE
Kif1c	common	FALSE
Zfp68	common	FALSE
Csnk1a1	common	FALSE

Pomt2	common	FALSE
Man2b1	common	FALSE
Atp6v0a2	common	FALSE
2610035D1	common	FALSE
Snrpb2	common	TRUE
Pdia3	common	FALSE
Eapp	common	FALSE
Mlx	common	TRUE
Galnt4	common	FALSE
Brd4	common	FALSE
Arhgap35	common	FALSE
Fnbp1l	common	FALSE
Hhip	common	FALSE
Galk2	common	FALSE
Vash1	common	FALSE
2810403A0	common	TRUE
Mir6910	common	FALSE
Itpa	common	FALSE
Ldlrap1	common	FALSE
Dhrs3	common	FALSE
0610011F0	common	FALSE
Pacsin3	common	FALSE
Tmem43	common	FALSE
Ostc	common	FALSE
Mtfr1l	common	FALSE
Psmb2	common	FALSE
Chchd1	common	FALSE
Ercc4	common	FALSE
C2cd2	common	FALSE
Mir6975	common	FALSE
Rpl22	common	FALSE
Ankrd39	common	FALSE
Armcx4	common	FALSE
Scai	common	FALSE
2810001G2	common	FALSE
Adnp	common	TRUE
Gpr180	common	FALSE
Btbd1	common	FALSE
Chmp2b	common	FALSE
Vps25	common	FALSE
Stx16	common	FALSE
Ptpn23	common	FALSE
Whrn	common	FALSE
Tmem237	common	FALSE
Npm3	common	FALSE
Stat6	common	TRUE
Zfp236	common	TRUE
Atad1	common	FALSE
Dcaf4	common	FALSE
9930021J0	common	FALSE

Myef2	common	TRUE
Extl3	common	FALSE
Atg4a	common	FALSE
Msi2	common	TRUE
Dedd	common	FALSE
Tmem87a	common	FALSE
Mir703	common	FALSE
Sphk1	common	FALSE
Cast	common	FALSE
Gtf2b	common	TRUE
Gfpt1	common	FALSE
Spns1	common	FALSE
Cdan1	common	FALSE
Atp11b	common	FALSE
Stxbp3a	common	FALSE
Decr2	common	FALSE
St8sia5	common	FALSE
Tram2	common	FALSE
Pex3	common	FALSE
Sppl3	common	FALSE
Pop4	common	FALSE
Zmat3	common	FALSE
Zufsp	common	FALSE
Ap2a2	common	FALSE
Fastk	common	FALSE
Mdm2	common	TRUE
Tada3	common	FALSE
Mrps11	common	FALSE
3110040N1	common	FALSE
Dgat1	common	FALSE
Crtap	common	FALSE
Cdk2ap2	common	FALSE
Vwa8	common	FALSE
Smpd2	common	FALSE
Accs	common	FALSE
Slc30a4	common	FALSE
Man1c1	common	FALSE
Lsm4	common	FALSE
Luc7l2	common	FALSE
Cnep1r1	common	FALSE
Cep44	common	FALSE
Capn7	common	FALSE
Dennd4c	common	FALSE
Sdcbp	common	FALSE
Loxl2	common	FALSE
Zfp952	common	FALSE
Sox11	common	TRUE
Snrpc	common	FALSE
Tns3	common	FALSE
Tbc1d20	common	FALSE

Ttc33	common	FALSE
Ell	common	FALSE
Fam13b	common	FALSE
Rrnad1	common	FALSE
Vasp	common	FALSE
Tsn	common	TRUE
Tro	common	FALSE
Ndufaf7	common	FALSE
Sar1a	common	FALSE
Rgs12	common	FALSE
Pdzrn3	common	FALSE
Fbxo18	common	FALSE
Tmod3	common	FALSE
Zfp292	common	FALSE
Hspg2	common	FALSE
Tcf7l2	common	TRUE
Tbx3	common	TRUE
Alg5	common	FALSE
Zfp691	common	TRUE
Scoc	common	FALSE
Kdm1a	common	FALSE
Dll1	common	FALSE
Zfp282	common	TRUE
Rpa3	common	FALSE
Rab12	common	FALSE
Cnih4	common	FALSE
Zfp799	common	FALSE
Vps13a	common	FALSE
Ube2e3	common	FALSE
Vps37a	common	FALSE
Zbtb41	common	TRUE
Pelo	common	FALSE
Hmg20a	common	TRUE
Bche	common	FALSE
1700030K0	common	FALSE
Inpp5e	common	FALSE
Ptpmt1	common	FALSE
Nrp	common	FALSE
Gpsm1	common	FALSE
Ift172	common	FALSE
Nf1	common	TRUE
Tnrc18	common	FALSE
Ikbip	common	FALSE
Fam102a	common	FALSE
Arhgef17	common	FALSE
Ap1s2	common	FALSE
2310011J0	common	TRUE
Obsl1	common	FALSE
Sec31a	common	FALSE
Tjap1	common	FALSE

Zfp532	common	TRUE
Azi2	common	FALSE
Npm3-ps1	common	FALSE
Nkiras2	common	FALSE
Trim44	common	FALSE
Ier5	common	FALSE
Sdhaf1	common	FALSE
Ggcx	common	FALSE
Wwc2	common	FALSE
C1d	common	FALSE
Dnmt3a	common	TRUE
Ccdc174	common	FALSE
Zkscan3	common	TRUE
Ppp1r12a	common	FALSE
Cnih1	common	FALSE
Ptgr2	common	FALSE
Mapk9	common	FALSE
Zdhhc4	common	FALSE
Mrpl32	common	FALSE
Klhl7	common	FALSE
Pcbd2	common	FALSE
Pfkm	common	FALSE
Pitpna	common	FALSE
Tnpo2	common	FALSE
F3	common	FALSE
Apeh	common	FALSE
Sgsm3	common	FALSE
Wrnip1	common	TRUE
Strip1	common	FALSE
1190007I0	common	FALSE
Efhd2	common	FALSE
Clk4	common	FALSE
Btf3l4	common	FALSE
Arpc5l	common	FALSE
Thoc2	common	TRUE
Arf4	common	FALSE
Dnlz	common	FALSE
Asb8	common	FALSE
Nrbp1	common	FALSE
4930503E2	common	FALSE
Marf1	common	FALSE
2510039O1	common	FALSE
1810043H(	common	FALSE
Atg101	common	FALSE
Sirt6	common	TRUE
Adrbk1	common	FALSE
Gstp1	common	FALSE
Adprm	common	FALSE
Prmt1	common	FALSE
Vgll4	common	FALSE

Coq9	common	FALSE
Clptm1l	common	FALSE
Alkbh3	common	FALSE
Ikbkb	common	FALSE
Asna1	common	FALSE
Lyn	common	FALSE
Upf3a	common	FALSE
Dnajc24	common	FALSE
Timm22	common	FALSE
Vps45	common	FALSE
Tfe3	common	TRUE
Tmem175	common	FALSE
Foxk1	common	TRUE
Rnf7	common	FALSE
Ubn1	common	FALSE
Bcas2	common	FALSE
Hexim1	common	FALSE
Ptpru	common	FALSE
Slc12a4	common	FALSE
Acot2	common	FALSE
Zfp444	common	TRUE
Stard3	common	FALSE
Spin1	common	FALSE
Vps51	common	FALSE
Chrac1	common	FALSE
Wdr83	common	TRUE
Paox	common	FALSE
Hdac7	common	FALSE
Lpar4	common	FALSE
Arfgap1	common	TRUE
Slc35f5	common	FALSE
Dlg5	common	FALSE
Akirin1	common	FALSE
Zc4h2	common	FALSE
Dnmbp	common	FALSE
Tmem218	common	FALSE
Spast	common	FALSE
Dkk2	common	FALSE
Sfr1	common	FALSE
Prickle1	common	FALSE
Etv4	common	TRUE
Tnfaip8l1	common	FALSE
Prkaa1	common	TRUE
Bloc1s2	common	FALSE
Enthd2	common	FALSE
Kif13a	common	FALSE
Fnip2	common	FALSE
Dapk3	common	FALSE
Gbf1	common	FALSE
Commd2	common	FALSE

Epc2	common	FALSE
4933434E2	common	FALSE
Spin4	common	FALSE
Usp22	common	FALSE
Cdh24	common	FALSE
Snx12	common	FALSE
Mfap3	common	FALSE
Ino80b	common	FALSE
Stx6	common	FALSE
Zscan26	common	TRUE
Mast4	common	FALSE
Abhd14a	common	FALSE
Ccdc34	common	FALSE
Plod3	common	FALSE
Magi3	common	FALSE
Phldb1	common	FALSE
Ifi30	common	FALSE
Ppp1r14b	common	FALSE
Wipf1	common	FALSE
Sec22b	common	FALSE
Clasrp	common	FALSE
Pdzd11	common	FALSE
Xist	common	FALSE
Uba5	common	FALSE
Sh2b3	common	FALSE
2610020CC	common	FALSE
Sesn3	common	FALSE
Tmem60	common	FALSE
Cwc25	common	FALSE
3110062M	common	FALSE
Hey1	common	TRUE
Mrps26	common	FALSE
Chic2	common	FALSE
Tpra1	common	FALSE
Rhob	common	FALSE
Hipk1	common	FALSE
Tvp23a	common	FALSE
Cat	common	TRUE
Gipc1	common	FALSE
Rragc	common	FALSE
Nsa2	common	FALSE
Vipas39	common	FALSE
Lats1	common	FALSE
Nova1	common	FALSE
Derl2	common	FALSE
Amdhd2	common	FALSE
Pogk	common	FALSE
Tmem64	common	FALSE
Etv5	common	TRUE
Bin3	common	FALSE

Tbc1d9b	common	FALSE
Esd	common	FALSE
Strn3	common	FALSE
Trrap	common	FALSE
H6pd	common	FALSE
Dnajb4	common	FALSE
Bptf	common	TRUE
Dusp16	common	FALSE
Fermt2	common	FALSE
Grtp1	common	FALSE
Cib1	common	FALSE
Zfp738	common	FALSE
Eif2ak2	common	FALSE
Oxr1	common	FALSE
Pigx	common	FALSE
Ispd	common	FALSE
Pan3	common	FALSE
Car12	common	FALSE
Sugp2	common	FALSE
Rsf1	common	FALSE
Dcaf7	common	FALSE
Tomm34	common	FALSE
Rnf166	common	FALSE
B230118H(	common	FALSE
Ppt2	common	FALSE
Akr1b8	common	FALSE
Proser3	common	FALSE
Herc2	common	FALSE
Ufm1	common	FALSE
Mex3a	common	FALSE
Cnot6l	common	FALSE
Acadsb	common	FALSE
Klhl23	common	FALSE
Pigv	common	FALSE
Med10	common	FALSE
6030407O(	common	FALSE
Pygb	common	FALSE
9030624J0	common	FALSE
Mob3a	common	FALSE
Rchy1	common	FALSE
Thoc7	common	FALSE
Cep350	common	FALSE
Golga1	common	FALSE
Lime1	common	FALSE
Zswim4	common	FALSE
Sh2b1	common	FALSE
Tanc1	common	FALSE
Jak1	common	FALSE
Elk1	common	TRUE
Mef2c	common	TRUE

Lrch3	common	FALSE
Cep112	common	FALSE
Polk	common	FALSE
Abhd10	common	FALSE
Cyb561a3	common	FALSE
Slc10a7	common	FALSE
Ahsa2	common	FALSE
Hsd17b4	common	FALSE
Ckap4	common	FALSE
Fam50a	common	FALSE
Psmd9	common	FALSE
Anapc2	common	FALSE
Gstt2	common	FALSE
Zfp27	common	TRUE
Urm1	common	FALSE
Arhgap42	common	FALSE
Tmem214	common	FALSE
Vhl	common	FALSE
Fyn	common	FALSE
Ctdnep1	common	FALSE
4632427E1	common	FALSE
Zfp865	common	FALSE
Ric8b	common	FALSE
Tmem150a	common	FALSE
Txn1	common	FALSE
Casp6	common	FALSE
Leprel2	common	FALSE
Gfra2	common	FALSE
Numb	common	FALSE
4931406P1	common	FALSE
Gpr137b-p	common	FALSE
Prkcsh	common	FALSE
Crkl	common	FALSE
Tet1	common	TRUE
Zmiz1	common	TRUE
Lcmt1	common	FALSE
Spty2d1	common	FALSE
Arhgap1	common	FALSE
Gpx8	common	FALSE
Zcchc6	common	FALSE
Ctbs	common	FALSE
Edem2	common	FALSE
Zfr	common	FALSE
Gpr107	common	FALSE
Ppp2r4	common	FALSE
Nol4l	common	FALSE
Gpbp1l1	common	FALSE
Chpt1	common	FALSE
Mxra7	common	FALSE
Msantd4	common	FALSE

Cmas	common	FALSE
Cdc42bpb	common	FALSE
Letmd1	common	FALSE
Tmem173	common	FALSE
Slc6a8	common	FALSE
Wdr60	common	FALSE
Arl15	common	FALSE
1700086O(	common	FALSE
Arhgef10	common	FALSE
Cops6	common	FALSE
Smad7	common	TRUE
Pot1b	common	FALSE
Ankrd16	common	FALSE
Eif4g3	common	FALSE
Cby1	common	FALSE
Rps29	common	FALSE
Ctdsp2	common	FALSE
Fech	common	FALSE
Ppp1r35	common	FALSE
Prrc2a	common	FALSE
Mpv17	common	FALSE
Hars2	common	FALSE
Glt25d1	common	FALSE
Akr1b10	common	FALSE
Ankrd54	common	FALSE
BC003331	common	FALSE
Zfp319	common	TRUE
Lman2l	common	FALSE
Mid1	common	FALSE
Lsamp	common	FALSE
0610037L1	common	FALSE
Btrc	common	FALSE
Crebbp	common	FALSE
Ptpn9	common	FALSE
Lgi4	common	FALSE
Igf1r	common	FALSE
Laptm5	common	FALSE
Ensa	common	FALSE
Frk	common	FALSE
D630003M	common	FALSE
Uqcrc1	common	FALSE
Sep-07	common	FALSE
Aldh1l2	common	FALSE
Tbl1xr1	common	TRUE
Ifnar2	common	FALSE
Usp49	common	FALSE
Ppp2cb	common	FALSE
Gulp1	common	FALSE
Fcho2	common	FALSE
Apc	common	FALSE

Arvcf	common	FALSE
Sfrp1	common	FALSE
Prdx4	common	FALSE
Ankib1	common	FALSE
Sike1	common	FALSE
Tmem136	common	FALSE
Igfbp5	common	FALSE
Ttc8	common	FALSE
Dstyk	common	FALSE
Fam193a	common	FALSE
Suv420h2	common	FALSE
Ust	common	FALSE
Gpsm3	common	FALSE
Ctdsp1	common	FALSE
Kdelr2	common	FALSE
Ptges3l	common	FALSE
Acaa2	common	FALSE
Zeb2os	common	FALSE
Irak4	common	FALSE
Bmf	common	FALSE
Acvr1	common	FALSE
Kmt2b	common	FALSE
Cers5	common	TRUE
Dnaic2	common	FALSE
Wdr8	common	FALSE
Agpat2	common	FALSE
Bud31	common	FALSE
Gpr17	common	FALSE
Pomp	common	FALSE
Gltscr1	common	FALSE
Rnf25	common	FALSE
Dsel	common	FALSE
Tm2d3	common	FALSE
Acin1	common	FALSE
Dock6	common	FALSE
Arhgef12	common	FALSE
Rhou	common	FALSE
Lrrk1	common	FALSE
Snhg18	common	FALSE
Lims1	common	FALSE
Dhx57	common	FALSE
Eef2	common	FALSE
Sep-01	common	FALSE
Ccdc97	common	FALSE
Rab3gap1	common	FALSE
4931406H2	common	FALSE
Ick	common	FALSE
Tpmt	common	FALSE
4933431E2	common	FALSE
Ppt1	common	FALSE

Pccb	common	FALSE
Vma21	common	FALSE
Ppapdc1b	common	FALSE
Atrn	common	FALSE
Mrps18a	common	FALSE
Stxbp3b	common	FALSE
Rev3l	common	FALSE
Yipf1	common	FALSE
Tor1a	common	FALSE
Lsm1	common	FALSE
Usp19	common	FALSE
Ppp1r12c	common	FALSE
Panx1	common	FALSE
Snrpd2	common	FALSE
Mrps16	common	FALSE
Taz	common	FALSE
Polm	common	FALSE
Zcchc24	common	FALSE
Brf1	common	TRUE
Oraov1	common	FALSE
Nprl2	common	FALSE
Lyrm4	common	FALSE
Tnrc6a	common	FALSE
Taok2	common	FALSE
Setd1b	common	FALSE
Ankmy2	common	FALSE
Slc39a7	common	FALSE
Tfpi	common	FALSE
Mcur1	common	FALSE
Col5a1	common	FALSE
Ap5z1	common	FALSE
Cenpt	common	FALSE
Ago1	common	FALSE
Zfp276	common	TRUE
Nkain4	common	FALSE
Pkn1	common	FALSE
Tbcel	common	FALSE
Lxn	common	FALSE
Plxnb1	common	FALSE
Ube2r2	common	FALSE
Fads2	common	FALSE
Gucd1	common	FALSE
Metap1d	common	FALSE
Abcd3	common	FALSE
Wash	common	FALSE
Rnf122	common	FALSE
Sarnp	common	FALSE
Rabgap1	common	FALSE
Pdcd10	common	FALSE
Fbxl6	common	FALSE

Gtf2i	common	TRUE
Rgl2	common	FALSE
Nelfcd	common	FALSE
1600012H(	common	FALSE
Rps10	common	TRUE
Ptpra	common	FALSE
Pcmtd1	common	FALSE
Pigs	common	FALSE
Wdpcp	common	FALSE
E130307A1	common	FALSE
Arhgef40	common	FALSE
Snhg6	common	FALSE
Tex261	common	FALSE
Smim8	common	FALSE
Ykt6	common	FALSE
Kcnq1ot1	common	FALSE
Exoc6	common	FALSE
Ncoa4	common	FALSE
Cmtm8	common	FALSE
Fam196a	common	FALSE
Rps9	common	FALSE
Ndufa9	common	FALSE
Orai1	common	FALSE
Sec23a	common	FALSE
Pvr	common	FALSE
Atp9b	common	FALSE
Ndufa10	common	FALSE
Man2a2	common	FALSE
Fkbp14	common	FALSE
Agl	common	FALSE
Sh3pxd2b	common	FALSE
Nek8	common	FALSE
Tenm3	common	FALSE
Gstk1	common	FALSE
Mef2a	common	TRUE
Unk	common	FALSE
Timm17b	common	FALSE
Hook3	common	FALSE
Mllt6	common	FALSE
Pdap1	common	FALSE
3110056K(	common	FALSE
Elf1	common	TRUE
Tmem242	common	FALSE
Bnip3l	common	FALSE
Slc25a20	common	FALSE
Sfswap	common	FALSE
2610507I0:	common	FALSE
Echs1	common	FALSE
Hsf2	common	TRUE
Pip4k2c	common	FALSE

Mrpl57	common	FALSE
Tatdn3	common	FALSE
Arrdc3	common	FALSE
Chmp4b	common	FALSE
Trim56	common	FALSE
Hdhd2	common	FALSE
Smap1	common	FALSE
Ube2j1	common	FALSE
Tbc1d10a	common	FALSE
Rhobtb1	common	FALSE
Leprel4	common	FALSE
Col11a1	common	FALSE
Btbd9	common	FALSE
Id1	common	TRUE
Fam178b	common	FALSE
Mrps17	common	FALSE
Proser1	common	FALSE
Dbi	common	FALSE
Map2k7	common	FALSE
Golgb1	common	FALSE
Gpx1	common	FALSE
Dctn4	common	FALSE
Rab22a	common	FALSE
Cdh2	common	FALSE
Fam117a	common	FALSE
Morf4l1	common	FALSE
Ngly1	common	FALSE
Psmb6	common	FALSE
Osbpl7	common	FALSE
Rfx5	common	TRUE
Efemp2	common	FALSE
Commd9	common	FALSE
Zfc3h1	common	FALSE
Rgcc	common	FALSE
Selo	common	FALSE
Zfp57	common	TRUE
Sri	common	FALSE
2610008E1	common	FALSE
Rab18	common	TRUE
Pcgf5	common	FALSE
Syk	common	FALSE
Limch1	common	FALSE
Sntb1	common	FALSE
Ano6	common	FALSE
Baiap2l2	common	FALSE
Arhgef10l	common	FALSE
2010320M	common	FALSE
Lifr	common	FALSE
Tspan14	common	FALSE
Lrrc8d	common	FALSE

Nbas	common	FALSE
Rheb	common	FALSE
Mknk1	common	FALSE
Chmp1a	common	FALSE
Igfbp3	common	FALSE
Cmtm3	common	FALSE
Kdm5b	common	TRUE
Suv420h1	common	FALSE
Mme	common	FALSE
Bnip2	common	FALSE
Osgep	common	FALSE
Zmat2	common	TRUE
Mdp1	common	FALSE
Fgd5	common	FALSE
Lrrc20	common	FALSE
Rps6kb2	common	FALSE
Epc1	common	FALSE
Gstz1	common	FALSE
Fam132a	common	FALSE
Smad6	common	TRUE
Ift27	common	FALSE
Tradd	common	FALSE
Tmco1	common	FALSE
Wdr26	common	FALSE
Mmp17	common	FALSE
Pim3	common	FALSE
Igsf11	common	FALSE
Slc35e3	common	FALSE
Mrps34	common	FALSE
Zc3h7b	common	FALSE
Casc4	common	FALSE
Lmf2	common	FALSE
Glrx	common	FALSE
Scnm1	common	FALSE
Pde5a	common	FALSE
Kifc3	common	FALSE
Antxr1	common	FALSE
Tmppe	common	FALSE
Srr	common	FALSE
Lgr4	common	FALSE
Pigc	common	FALSE
Fbxw17	common	FALSE
Saysd1	common	FALSE
Ypel1	common	FALSE
Tmem230	common	FALSE
Gm14325	common	TRUE
Ugp2	common	TRUE
Ubr4	common	FALSE
Mgea5	common	FALSE
9530068E0	common	FALSE

Zfp329	common	TRUE
BC024978	common	FALSE
Gab1	common	FALSE
Ppil2	common	FALSE
Ppm1b	common	FALSE
Frem2	common	FALSE
4833420G1	common	FALSE
Adamts6	common	FALSE
2610001J0	common	FALSE
Angptl2	common	FALSE
Mmp16	common	FALSE
Klf3	common	TRUE
Bfar	common	FALSE
Sorcs2	common	FALSE
Mmp2	common	FALSE
Adamts10	common	FALSE
Plac1	common	FALSE
Lix1l	common	FALSE
Dync1li2	common	FALSE
Ccdc167	common	FALSE
Thap7	common	FALSE
2310009A0	common	FALSE
3830406C1	common	FALSE
Ifit2	common	FALSE
Myo1c	common	FALSE
Chn2	common	FALSE
Ddah1	common	FALSE
Insig2	common	FALSE
Pabpn1	common	FALSE
Tmem261	common	FALSE
Fbxw7	common	FALSE
Deb1	common	FALSE
Rbm18	common	FALSE
Alkbh6	common	FALSE
Cttn	common	FALSE
Zfpl1	common	FALSE
Mecp2	common	TRUE
Ctso	common	FALSE
Kiz	common	FALSE
Rab4a	common	FALSE
Klhl36	common	FALSE
Aldh6a1	common	FALSE
Ccdc78	common	FALSE
Elmod2	common	FALSE
Vimp	common	FALSE
Zmat5	common	FALSE
Rpl35a	common	FALSE
Pitpnc1	common	FALSE
Zc3h11a	common	TRUE
Tlcd2	common	FALSE

Fbn1	common	FALSE
Col25a1	common	FALSE
Bag1	common	FALSE
Cnot8	common	FALSE
Paqr4	common	FALSE
Rhoj	common	FALSE
Vps41	common	FALSE
Arglu1	common	FALSE
Blvra	common	FALSE
Rictor	common	FALSE
Rps15a	common	FALSE
Pbrm1	common	FALSE
Trim41	common	FALSE
Thap3	common	FALSE
Unc50	common	FALSE
Clmn	common	FALSE
Mamdc2	common	FALSE
Adamts12	common	FALSE
Etfdh	common	FALSE
Cmc1	common	FALSE
Itgb5	common	FALSE
Szt2	common	FALSE
Irs1	common	FALSE
Tmed2	common	FALSE
Cyp39a1	common	FALSE
Cryl1	common	FALSE
Rps16	common	FALSE
Slirp	common	FALSE
Tmem179b	common	FALSE
Tmem80	common	FALSE
Colgalt2	common	FALSE
1110059G1	common	FALSE
Fahd2a	common	FALSE
2700089E2	common	FALSE
Fblim1	common	FALSE
Iah1	common	FALSE
Fnbp1	common	FALSE
Prpf40b	common	FALSE
Pdcd6	common	FALSE
Rn45s	common	FALSE
Wbp4	common	FALSE
Ccdc12	common	FALSE
Arhgef16	common	FALSE
Agpat3	common	FALSE
Fam229b	common	FALSE
Ptpn6	common	FALSE
Stk16	common	FALSE
Becn1	common	FALSE
Ptp4a3	common	FALSE
Exoc7	common	FALSE

Rab5a	common	FALSE
Mrpl30	common	FALSE
Mpdu1	common	FALSE
Pias3	common	FALSE
Pgap3	common	FALSE
R3hdm2	common	TRUE
Rbm4	common	FALSE
Synj2	common	FALSE
Afap1l1	common	FALSE
Galnt2	common	FALSE
Csad	common	FALSE
Rplp2	common	FALSE
Zmym3	common	FALSE
Dock7	common	FALSE
Son	common	FALSE
Gstm5	common	FALSE
Wsb1	common	FALSE
Yipf5	common	FALSE
Ankrd23	common	FALSE
Hunk	common	FALSE
Dvl3	common	FALSE
Wasf2	common	FALSE
Tmem198b	common	FALSE
Smarcb1	common	TRUE
Spry4	common	FALSE
Shisa2	common	FALSE
Tpcn2	common	FALSE
Msl1	common	FALSE
Tcn2	common	FALSE
Zfp866	common	TRUE
Npepl1	common	FALSE
Picalm	common	FALSE
Lrrc45	common	FALSE
Brms1l	common	FALSE
Ppp1r9b	common	FALSE
Rnpepl1	common	FALSE
Rsrc2	common	FALSE
Jarid2	common	FALSE
D17Wsu10	common	FALSE
Spag7	common	TRUE
Tmem170b	common	FALSE
Stard13	common	FALSE
Phf21a	common	TRUE
Arhgef11	common	FALSE
Dbnl	common	FALSE
Mrps12	common	FALSE
Gm15545	common	FALSE
Ccdc13	common	FALSE
Poln	common	FALSE
Vps37b	common	FALSE

Aida	common	FALSE
Ltbp1	common	FALSE
Jmjd7	common	FALSE
Akr1e1	common	FALSE
Hmga2-ps1	common	FALSE
Whsc1l1	common	FALSE
Igdcc3	NCC	FALSE
Grrp1	NCC	FALSE
Plcg2	NCC	FALSE
Fndc3c1	NCC	FALSE
Ipo5	NCC	FALSE
Lin28a	NCC	TRUE
Prtg	NCC	FALSE
Sep-11	NCC	FALSE
Trim71	NCC	FALSE
Psat1	NCC	FALSE
Vars	NCC	FALSE
Robo1	NCC	FALSE
Shmt1	NCC	FALSE
Hk2	NCC	FALSE
E130012A1	NCC	FALSE
Lin28b	NCC	TRUE
Tnfrsf19	NCC	FALSE
Pax3	NCC	TRUE
Mthfd1	NCC	TRUE
Ddx21	NCC	FALSE
Nop2	NCC	FALSE
Gart	NCC	FALSE
Dctd	NCC	FALSE
Slc16a1	NCC	FALSE
Exo1	NCC	FALSE
Heatr1	NCC	FALSE
Sall4	NCC	TRUE
Bub1	NCC	FALSE
Pkm	NCC	TRUE
Lhpp	NCC	FALSE
Cct6a	NCC	FALSE
Nsun2	NCC	FALSE
Nolc1	NCC	FALSE
Fam89a	NCC	FALSE
Slc2a1	NCC	FALSE
Ldha	NCC	FALSE
Mcm10	NCC	FALSE
Nop56	NCC	FALSE
Gemin5	NCC	FALSE
Sdad1	NCC	FALSE
Grwd1	NCC	FALSE
Fkbp4	NCC	FALSE
Prmt5	NCC	FALSE
Ttll4	NCC	FALSE

Lbr	NCC	FALSE
Hsph1	NCC	FALSE
Tomm70a	NCC	FALSE
Atic	NCC	FALSE
Hspa9	NCC	FALSE
Pprc1	NCC	FALSE
Cecr2	NCC	FALSE
Dnmt3b	NCC	FALSE
Lama1	NCC	FALSE
Ctps	NCC	FALSE
Srm	NCC	FALSE
Suv39h2	NCC	FALSE
Gemin4	NCC	FALSE
Bcat1	NCC	FALSE
Sfrp2	NCC	FALSE
Stip1	NCC	FALSE
Cdv3	NCC	FALSE
Gpsm2	NCC	FALSE
Trip13	NCC	FALSE
Melk	NCC	FALSE
Ivns1abp	NCC	FALSE
Sdc1	NCC	FALSE
Abcf2	NCC	TRUE
Plk1	NCC	FALSE
Cct5	NCC	FALSE
Aurka	NCC	FALSE
Aldh1a2	NCC	FALSE
Rrp12	NCC	FALSE
Crabp1	NCC	FALSE
Polr1b	NCC	FALSE
Bop1	NCC	FALSE
Seh1l	NCC	FALSE
C1qbp	NCC	FALSE
Espl1	NCC	FALSE
Asf1b	NCC	FALSE
2700038G2	NCC	FALSE
Rrs1	NCC	FALSE
Nek2	NCC	FALSE
Tcf7	NCC	TRUE
Fgfr2	NCC	FALSE
Ppm1g	NCC	FALSE
Arid3a	NCC	TRUE
Iars	NCC	FALSE
Wdhd1	NCC	FALSE
Ercc6l	NCC	FALSE
Haus6	NCC	FALSE
Cdc6	NCC	FALSE
Spsb4	NCC	FALSE
Dis3	NCC	TRUE
Pknox2	NCC	TRUE

Mina	NCC	FALSE
Mob3b	NCC	FALSE
Incenp	NCC	FALSE
Tfap2b	NCC	TRUE
Nup107	NCC	TRUE
Kif2c	NCC	FALSE
Ect2	NCC	FALSE
Rcl1	NCC	FALSE
Pphln1	NCC	FALSE
Dars	NCC	FALSE
Wfikkn1	NCC	FALSE
Ticrr	NCC	FALSE
Spag5	NCC	FALSE
Eme1	NCC	FALSE
Cbx2	NCC	FALSE
Blm	NCC	FALSE
Foxm1	NCC	TRUE
Nr6a1	NCC	TRUE
Wee1	NCC	FALSE
Rad51	NCC	FALSE
Pkdcc	NCC	FALSE
Ska3	NCC	FALSE
Polr3g	NCC	TRUE
Cdc45	NCC	FALSE
Cdc25c	NCC	FALSE
Sgol1	NCC	FALSE
Nat10	NCC	FALSE
Mybl2	NCC	TRUE
Plk4	NCC	FALSE
Utp15	NCC	FALSE
Wdr36	NCC	FALSE
B3gnt5	NCC	FALSE
Aurkb	NCC	FALSE
Dars2	NCC	FALSE
BC055324	NCC	FALSE
Tsr1	NCC	FALSE
Psmd1	NCC	FALSE
Brca1	NCC	TRUE
Eprs	NCC	FALSE
Ipo4	NCC	FALSE
Prdx6	NCC	FALSE
Tjp2	NCC	FALSE
Ccdc86	NCC	FALSE
Rrp1b	NCC	FALSE
Ipo7	NCC	FALSE
Ccnf	NCC	FALSE
Slc1a3	NCC	FALSE
Fam64a	NCC	FALSE
Cep78	NCC	FALSE
Eif1a	NCC	FALSE

Chaf1b	NCC	FALSE
Tbrg4	NCC	FALSE
Mn1	NCC	FALSE
Mcm8	NCC	FALSE
C330027C0	NCC	FALSE
Fbxo5	NCC	FALSE
Nsl1	NCC	FALSE
Swap70	NCC	FALSE
Lrrc59	NCC	FALSE
Nmnat2	NCC	FALSE
Zwilch	NCC	FALSE
Wdr75	NCC	FALSE
Anln	NCC	FALSE
Ddx20	NCC	TRUE
Scd1	NCC	FALSE
Snai1	NCC	TRUE
Ralgps2	NCC	FALSE
Entpd6	NCC	FALSE
D19Bwg13.	NCC	FALSE
Ttk	NCC	FALSE
Rps6ka1	NCC	FALSE
Dlgap5	NCC	FALSE
Tfrc	NCC	FALSE
Fgfr1op	NCC	FALSE
Itga9	NCC	FALSE
Kif11	NCC	FALSE
Zfp593	NCC	FALSE
Lyar	NCC	FALSE
Suv39h1	NCC	FALSE
3110082I1	NCC	FALSE
Cacybp	NCC	FALSE
Ddx51	NCC	FALSE
Chek2	NCC	FALSE
Prps1	NCC	FALSE
Cdca5	NCC	FALSE
Greb1l	NCC	FALSE
Mir17hg	NCC	FALSE
Actn1	NCC	FALSE
Mars	NCC	FALSE
Tfap4	NCC	TRUE
Zfp280c	NCC	FALSE
Cenpf	NCC	FALSE
Meis2	NCC	TRUE
Kpna1	NCC	FALSE
Ncapg2	NCC	FALSE
Mphosph1(	NCC	FALSE
Ak4	NCC	FALSE
Utp18	NCC	TRUE
Acly	NCC	FALSE
Metap2	NCC	FALSE

Mettl13	NCC	FALSE
Rad54l	NCC	FALSE
Wdr3	NCC	FALSE
Wdr12	NCC	FALSE
Chchd4	NCC	FALSE
Hsp90aa1	NCC	FALSE
Nuf2	NCC	FALSE
Mad2l1	NCC	FALSE
Spdl1	NCC	FALSE
Gfm1	NCC	FALSE
Gar1	NCC	TRUE
Snhg4	NCC	FALSE
Wbscr16	NCC	FALSE
Larp4	NCC	TRUE
Skiv2l2	NCC	FALSE
Rnf219	NCC	FALSE
Bysl	NCC	FALSE
Fam208b	NCC	FALSE
Wdr46	NCC	FALSE
Rbpms2	NCC	FALSE
Kti12	NCC	FALSE
Lck	NCC	FALSE
Sephs2	NCC	FALSE
Cachd1	NCC	FALSE
Arrb1	NCC	FALSE
Nop9	NCC	FALSE
Adk	NCC	FALSE
Gtse1	NCC	FALSE
Prmt7	NCC	FALSE
Lrpprc	NCC	FALSE
Cenpn	NCC	FALSE
Kif20b	NCC	FALSE
Dnajc11	NCC	FALSE
Kif4	NCC	FALSE
Rad51ap1	NCC	FALSE
Slc25a26	NCC	FALSE
E2f7	NCC	TRUE
Sall3	NCC	TRUE
Gcat	NCC	FALSE
Casc5	NCC	FALSE
Pms1	NCC	FALSE
Pcsk9	NCC	FALSE
Cep85	NCC	FALSE
Mcph1	NCC	FALSE
Traip	NCC	FALSE
Gars	NCC	FALSE
Nup54	NCC	FALSE
Orc2	NCC	FALSE
Kpna3	NCC	FALSE
Mpp6	NCC	FALSE

Hmmr	NCC	FALSE
Galnt7	NCC	FALSE
Nupl1	NCC	FALSE
Spc25	NCC	FALSE
Rars	NCC	FALSE
E2f8	NCC	TRUE
Cdca2	NCC	FALSE
Tomm40	NCC	FALSE
Ybx1	NCC	TRUE
Rad18	NCC	FALSE
Nol6	NCC	FALSE
Rrp9	NCC	FALSE
Urb2	NCC	FALSE
Ska1	NCC	FALSE
Ppan	NCC	FALSE
Prdm12	NCC	TRUE
Ednra	NCC	FALSE
Cstf2	NCC	TRUE
Mdc1	NCC	FALSE
BC030867	NCC	FALSE
Nle1	NCC	FALSE
Celf1	NCC	FALSE
Aspm	NCC	FALSE
Elac2	NCC	FALSE
Pwp2	NCC	FALSE
Fkbp11	NCC	FALSE
Polr1e	NCC	FALSE
Tma16	NCC	FALSE
Ruvbl2	NCC	FALSE
Ttc27	NCC	FALSE
Kpna2	NCC	FALSE
BC027231	NCC	FALSE
Hist1h1b	NCC	FALSE
Aatf	NCC	FALSE
Usp25	NCC	FALSE
Fam81a	NCC	FALSE
Aars	NCC	FALSE
Otud6b	NCC	FALSE
Dhx29	NCC	FALSE
Net1	NCC	FALSE
Gal3st4	NCC	FALSE
Ccne2	NCC	FALSE
Zfp503	NCC	TRUE
Tnpo3	NCC	FALSE
Ankrd27	NCC	FALSE
Apitd1	NCC	FALSE
Ddias	NCC	FALSE
Mycn	NCC	TRUE
Ybx3	NCC	FALSE
Enoph1	NCC	FALSE

Cul1	NCC	FALSE
Nadk2	NCC	FALSE
Exosc7	NCC	FALSE
Dscc1	NCC	FALSE
Naa25	NCC	FALSE
Fastkd3	NCC	FALSE
Gtpbp4	NCC	FALSE
Imp4	NCC	FALSE
Ppp1cb	NCC	FALSE
Usp14	NCC	FALSE
Fancd2	NCC	FALSE
Ndc80	NCC	FALSE
Sgol2	NCC	FALSE
Otud4	NCC	TRUE
Dnaaf2	NCC	FALSE
Srsf2	NCC	FALSE
Zfp521	NCC	TRUE
Slc1a5	NCC	FALSE
Las1l	NCC	TRUE
Bckdhb	NCC	FALSE
Nifk	NCC	FALSE
Troap	NCC	FALSE
Kntc1	NCC	FALSE
Gpatch4	NCC	FALSE
Spata5	NCC	FALSE
Pbk	NCC	FALSE
Rbm19	NCC	FALSE
Canx	NCC	TRUE
Gprc5c	NCC	FALSE
Eif3d	NCC	FALSE
Stil	NCC	FALSE
Siah1b	NCC	FALSE
Esco2	NCC	FALSE
Clic4	NCC	FALSE
Rai14	NCC	FALSE
Srfbp1	NCC	FALSE
Uba6	NCC	FALSE
Slc45a3	NCC	FALSE
Med18	NCC	FALSE
Nudc	NCC	FALSE
Ints5	NCC	FALSE
Ppwd1	NCC	FALSE
Zfp930	NCC	TRUE
Eif4ebp2	NCC	FALSE
Cenpq	NCC	FALSE
Cct2	NCC	FALSE
Ctdp1	NCC	FALSE
Tmem132c	NCC	FALSE
Arhgap19	NCC	FALSE
Sall1	NCC	TRUE

Nampt	NCC	FALSE
Cmss1	NCC	FALSE
Ckap2l	NCC	FALSE
Mogs	NCC	FALSE
Cenpi	NCC	FALSE
Zmym1	NCC	FALSE
Trmt6	NCC	FALSE
Ahsa1	NCC	FALSE
D16Ertd47	NCC	FALSE
Ackr3	NCC	FALSE
Eif4h	NCC	FALSE
Dyrk3	NCC	FALSE
Smc4	NCC	FALSE
Prep	NCC	FALSE
Nol8	NCC	FALSE
Shmt2	NCC	FALSE
Gins3	NCC	FALSE
Gins1	NCC	FALSE
Pnpt1	NCC	FALSE
Cep55	NCC	FALSE
Pask	NCC	FALSE
Urb1	NCC	FALSE
Cndp2	NCC	FALSE
Plaa	NCC	FALSE
Cars	NCC	FALSE
Fam98a	NCC	FALSE
Fam84b	NCC	FALSE
Tube1	NCC	FALSE
Tars2	NCC	FALSE
Mak16	NCC	FALSE
Mrto4	NCC	FALSE
Orc1	NCC	FALSE
Oip5	NCC	FALSE
Xrcc6bp1	NCC	FALSE
Rras2	NCC	FALSE
Rmi2	NCC	FALSE
Cenpw	NCC	FALSE
Immt	NCC	FALSE
Tiparp	NCC	FALSE
Noc3l	NCC	FALSE
Mogat2	NCC	FALSE
Mat2a	NCC	FALSE
Ltv1	NCC	FALSE
Gbx2	NCC	TRUE
Cth	NCC	FALSE
Ipmk	NCC	FALSE
Krr1	NCC	FALSE
Rpp40	NCC	FALSE
Dbr1	NCC	FALSE
Wdr55	NCC	FALSE

Ddx10	NCC	FALSE
Cs	NCC	FALSE
Wnt1	NCC	FALSE
L2hgdh	NCC	FALSE
Arhgap11a	NCC	FALSE
Wars	NCC	FALSE
Rad23b	NCC	FALSE
Syce2	NCC	FALSE
Gtpbp10	NCC	FALSE
Axin2	NCC	FALSE
Farsb	NCC	FALSE
Ccnj	NCC	FALSE
Nkd1	NCC	FALSE
Cenpu	NCC	FALSE
Abcb7	NCC	FALSE
Polq	NCC	FALSE
Rpusd2	NCC	FALSE
Dhx37	NCC	FALSE
Stag1	NCC	FALSE
1700052N1	NCC	FALSE
Hnrnph1	NCC	FALSE
Pus1	NCC	FALSE
Gtf2e1	NCC	FALSE
Trmt61a	NCC	FALSE
2810408I1	NCC	FALSE
Cct7	NCC	FALSE
Alg13	NCC	FALSE
Abhd17c	NCC	FALSE
Ror1	NCC	FALSE
Eif2b3	NCC	FALSE
Zfp280b	NCC	TRUE
Neurog2	NCC	TRUE
A630089N(	NCC	FALSE
Nufip1	NCC	FALSE
Shq1	NCC	FALSE
Rps6ka3	NCC	FALSE
Ptgis	NCC	FALSE
Fpgs	NCC	FALSE
Eri2	NCC	FALSE
Cry1	NCC	FALSE
Ece2	NCC	FALSE
Vprbp	NCC	FALSE
Dgcr8	NCC	TRUE
Pecr	NCC	FALSE
Fam195a	NCC	FALSE
L3mbtl2	NCC	FALSE
Apod	NCC	FALSE
Pde3b	NCC	FALSE
Top3a	NCC	FALSE
Rps6ka6	NCC	FALSE

Sass6	NCC	FALSE
Gabpb1	NCC	TRUE
Tinf2	NCC	FALSE
Asns	NCC	FALSE
Ube2c	NCC	FALSE
Clp1	NCC	FALSE
Kif23	NCC	FALSE
Arid3b	NCC	TRUE
Peo1	NCC	FALSE
Mettl16	NCC	FALSE
Gga2	NCC	FALSE
Parl	NCC	FALSE
Slc7a5	NCC	FALSE
Senp2	NCC	FALSE
Gsg2	NCC	FALSE
Ppp1r7	NCC	FALSE
Zranb3	NCC	FALSE
Ccdc160	NCC	FALSE
Psmd14	NCC	FALSE
Zgrf1	NCC	FALSE
Usp15	NCC	FALSE
Orc5	NCC	FALSE
Hoxa1	NCC	TRUE
Thada	NCC	FALSE
Slc16a3	NCC	FALSE
Dhodh	NCC	FALSE
Dut	NCC	FALSE
Ccdc6	NCC	FALSE
Nom1	NCC	FALSE
Pak4	NCC	FALSE
Shb	NCC	FALSE
Gpn3	NCC	FALSE
Mastl	NCC	FALSE
Nars	NCC	FALSE
Tango6	NCC	FALSE
Acy1	NCC	FALSE
Recql4	NCC	FALSE
Nop14	NCC	FALSE
Etaa1	NCC	FALSE
Mrpl12	NCC	FALSE
Prdm4	NCC	TRUE
Hoxaas3	NCC	FALSE
Rpp38	NCC	FALSE
Nadk	NCC	FALSE
Cep135	NCC	FALSE
Naf1	NCC	FALSE
Rwdd4a	NCC	FALSE
Trmt10c	NCC	FALSE
Aars2	NCC	FALSE
Eogt	NCC	FALSE

Coil	NCC	FALSE
Gfm2	NCC	FALSE
Car14	NCC	FALSE
Ppp2r5a	NCC	FALSE
Aldoa	NCC	FALSE
Nop16	NCC	FALSE
Stard7	NCC	FALSE
Nsun5	NCC	FALSE
Lipt2	NCC	FALSE
Nob1	NCC	FALSE
1810011O1	NCC	FALSE
Uspl1	NCC	FALSE
Mdm1	NCC	FALSE
Klhl12	NCC	FALSE
Trmt5	NCC	FALSE
Rnmtl1	NCC	FALSE
Ept1	NCC	FALSE
Enkd1	NCC	FALSE
Rnaseh1	NCC	FALSE
Znrf3	NCC	FALSE
Pcnt	NCC	FALSE
Gfpt2	NCC	FALSE
Mgme1	NCC	FALSE
Znhit6	NCC	FALSE
Trmt11	NCC	FALSE
Fut10	NCC	FALSE
Dusp5	NCC	FALSE
Srpk1	NCC	FALSE
Pcgf6	NCC	FALSE
Baiap2l1	NCC	FALSE
Clhc1	NCC	FALSE
Mis12	NCC	FALSE
Homez	NCC	TRUE
Trappc13	NCC	FALSE
Cpne1	NCC	FALSE
Acvr2b	NCC	FALSE
Wdr90	NCC	FALSE
Rpf2	NCC	FALSE
Itpr1	NCC	FALSE
Fkbp5	NCC	FALSE
Ddx23	NCC	FALSE
Aldh18a1	NCC	FALSE
Racgap1	NCC	FALSE
Cwf19l1	NCC	FALSE
Qtrt1	NCC	FALSE
Lrr1	NCC	FALSE
Mrps18b	NCC	FALSE
Usp24	NCC	FALSE
Wdr73	NCC	FALSE
Ddx31	NCC	FALSE

Rtel1	NCC	FALSE
BC052040	NCC	FALSE
Sms	NCC	FALSE
Desi1	NCC	FALSE
Fanci	NCC	FALSE
Sfrp5	NCC	FALSE
Arhgap10	NCC	FALSE
Bag2	NCC	FALSE
Gmfb	NCC	FALSE
Snora65	NCC	FALSE
Atp11c	NCC	FALSE
9130401M	NCC	FALSE
Nek6	NCC	FALSE
Yars	NCC	FALSE
Ptbp3	NCC	FALSE
Dph5	NCC	FALSE
Rabepk	NCC	FALSE
Tufm	NCC	FALSE
Usp8	NCC	FALSE
G3bp2	NCC	FALSE
Osgepl1	NCC	FALSE
Ap1ar	NCC	FALSE
Xpo6	NCC	FALSE
Ubiad1	NCC	FALSE
Palb2	NCC	FALSE
Hic2	NCC	TRUE
Acaca	NCC	FALSE
Edem1	NCC	FALSE
Grsf1	NCC	FALSE
Eif2b5	NCC	FALSE
Fastkd5	NCC	FALSE
Aimp2	NCC	FALSE
Ccdc117	NCC	FALSE
Ckap5	NCC	FALSE
Nr2f2	NCC	TRUE
Dnaja3	NCC	FALSE
Ubox5	NCC	FALSE
Zfp423	NCC	FALSE
Hist1h2ae	NCC	FALSE
Ppp5c	NCC	TRUE
Zdhhc3	NCC	FALSE
Smtnl2	NCC	FALSE
Wasf3	NCC	FALSE
Xkr5	NCC	FALSE
Xrcc2	NCC	FALSE
Yae1d1	NCC	FALSE
Zmynd19	NCC	FALSE
5730408K0	NCC	FALSE
Ankle1	NCC	FALSE
Tex30	NCC	FALSE

Wdr74	NCC	FALSE
Dimt1	NCC	FALSE
Mis18a	NCC	FALSE
Tkt	NCC	FALSE
Cipc	NCC	FALSE
Rab23	NCC	FALSE
Haus5	NCC	FALSE
Pcid2	NCC	FALSE
Dph1	NCC	FALSE
Spryd4	NCC	FALSE
Arl5b	NCC	FALSE
Atp1b3	NCC	FALSE
Med4	NCC	FALSE
Haus2	NCC	FALSE
Rasl11a	NCC	FALSE
Rad54b	NCC	FALSE
Wdr4	NCC	FALSE
Thg1l	NCC	FALSE
Fem1b	NCC	FALSE
Vamp3	NCC	TRUE
Mutyh	NCC	FALSE
Pkmyt1	NCC	FALSE
Twistnb	NCC	FALSE
Brip1	NCC	FALSE
Pus10	NCC	FALSE
Bbx	NCC	TRUE
Ercc8	NCC	FALSE
Mipep	NCC	FALSE
Uhrf1bp1	NCC	FALSE
Amot	NCC	FALSE
Cit	NCC	FALSE
Gstcd	NCC	FALSE
Slc25a33	NCC	FALSE
Msx3	NCC	TRUE
Emb	NCC	FALSE
Rab34	NCC	FALSE
Trib1	NCC	FALSE
Nek4	NCC	FALSE
Tbc1d31	NCC	FALSE
Slc35e1	NCC	FALSE
Hccs	NCC	FALSE
Abi2	NCC	FALSE
Uchl3	NCC	FALSE
Pidd1	NCC	FALSE
Fam53b	NCC	FALSE
2810408M	NCC	FALSE
Ccdc14	NCC	FALSE
Dcbld2	NCC	FALSE
Kif14	NCC	FALSE
Tefm	NCC	FALSE

Ppp4r1	NCC	FALSE
Ccdc18	NCC	FALSE
Tmem97	NCC	FALSE
Gm17296	NCC	FALSE
Nif3l1	NCC	FALSE
Trub1	NCC	FALSE
Mrpl19	NCC	FALSE
Timm8a1	NCC	TRUE
Errfi1	NCC	FALSE
Hs3st3b1	NCC	FALSE
Cinp	NCC	FALSE
Psmd11	NCC	FALSE
Slc6a6	NCC	FALSE
Mecr	NCC	FALSE
Pawr	NCC	FALSE
Taf4b	NCC	FALSE
Rnf168	NCC	FALSE
Tut1	NCC	FALSE
Lin54	NCC	FALSE
Hist1h2ab	NCC	FALSE
Depdc1a	NCC	FALSE
Hs6st2	NCC	FALSE
Adat1	NCC	FALSE
4930427A(	NCC	FALSE
Heatr2	NCC	FALSE
Nars2	NCC	FALSE
Sfxn2	NCC	FALSE
Gtf2a1	NCC	TRUE
Bivm	NCC	FALSE
Hbb-bh1	NCC	FALSE
Dtwd1	NCC	FALSE
Fam175a	NCC	FALSE
Mnd1	NCC	FALSE
Hdgf	NCC	FALSE
Smcr8	NCC	FALSE
Mcc	NCC	FALSE
Slc25a15	NCC	FALSE
Sfmbt1	NCC	FALSE
Boc	NCC	FALSE
Nusap1	NCC	FALSE
Prc1	NCC	FALSE
Exosc1	NCC	FALSE
Adck1	NCC	FALSE
Rad1	NCC	FALSE
Pja1	NCC	FALSE
Llph	NCC	FALSE
Srbd1	NCC	TRUE
Wnk3	NCC	FALSE
Car11	NCC	FALSE
Dgat2	NCC	FALSE

Slc30a5	NCC	FALSE
Cldn1	NCC	FALSE
Wrap53	NCC	FALSE
Kbtbd8	NCC	FALSE
Usp36	NCC	FALSE
Ice2	NCC	FALSE
Alms1	NCC	FALSE
Hmha1	NCC	FALSE
Rdh13	NCC	FALSE
Rangrf	NCC	FALSE
Wdr70	NCC	FALSE
Grhpr	NCC	TRUE
Cxcr4	NCC	FALSE
Ndufaf6	NCC	FALSE
Lrrc58	NCC	FALSE
Ddx25	NCC	FALSE
Eif2b1	NCC	FALSE
Pdxk	NCC	FALSE
Zfp706	NCC	TRUE
Ush1c	NCC	FALSE
Zfp703	NCC	FALSE
Cyb5b	NCC	FALSE
Fastkd1	NCC	FALSE
Nfx1	NCC	FALSE
Elp6	NCC	FALSE
Rdh10	NCC	FALSE
Ube2t	NCC	FALSE
Mthfd2	NCC	FALSE
Usp46	NCC	FALSE
Myo19	NCC	FALSE
Dclre1b	NCC	FALSE
Lpcat1	NCC	FALSE
Mettl5	NCC	FALSE
Aff3	NCC	FALSE
Piga	NCC	FALSE
Stk39	NCC	FALSE
Rap1gds1	NCC	FALSE
Hoxa9	NCC	TRUE
2700097O(	NCC	FALSE
Chst2	NCC	FALSE
Vac14	NCC	FALSE
Zfp385a	NCC	TRUE
Tubb4b	NCC	FALSE
Gclc	NCC	FALSE
Hyou1	NCC	FALSE
Zfp850	NCC	TRUE
Arxes1	NCC	FALSE
Tmem183a	NCC	FALSE
Pdss1	NCC	FALSE
Gli1	NCC	TRUE

Crlf3	NCC	FALSE
Alpl	NCC	FALSE
Toe1	NCC	FALSE
Pdss2	NCC	FALSE
Ebna1bp2	NCC	FALSE
Bard1	NCC	FALSE
Tbccd1	NCC	FALSE
Homer1	NCC	FALSE
1810043G(	NCC	FALSE
Cep83	NCC	FALSE
Kctd15	NCC	FALSE
Edrf1	NCC	FALSE
Fam83d	NCC	FALSE
Oaf	NCC	FALSE
Zbtb11	NCC	TRUE
Rbm6	NCC	FALSE
Mtrr	NCC	FALSE
Cbll1	NCC	FALSE
Rev1	NCC	FALSE
Snhg12	NCC	FALSE
Tspan4	NCC	FALSE
Nubpl	NCC	FALSE
Zfp451	NCC	TRUE
Nubp1	NCC	FALSE
Polr3b	NCC	FALSE
Usp38	NCC	FALSE
Ada	NCC	FALSE
Larp1	NCC	TRUE
Kif18a	NCC	FALSE
5830418K(	NCC	FALSE
Ttc30a1	NCC	FALSE
Gen1	NCC	FALSE
Mllt3	NCC	FALSE
Fam20b	NCC	FALSE
Ak6	NCC	TRUE
Nupl2	NCC	FALSE
Utp23	NCC	FALSE
Tmem194	NCC	FALSE
Naa10	NCC	FALSE
Rpl34	NCC	FALSE
Aldh9a1	NCC	FALSE
Hoxb6	NCC	TRUE
Timm50	NCC	FALSE
Fam203a	NCC	FALSE
Pafah1b2	NCC	FALSE
Smc3	NCC	TRUE
Ubtf	NCC	TRUE
Fasn	NCC	FALSE
Snhg5	NCC	FALSE
Rbbp5	NCC	TRUE

1190002N1	NCC	FALSE
Mtmr4	NCC	FALSE
Iqgap3	NCC	FALSE
Hira	NCC	FALSE
Col1a2	NCC	FALSE
Zdhhc13	NCC	FALSE
Pitpnb	NCC	FALSE
Bcl2l11	NCC	FALSE
Lactb2	NCC	FALSE
Eno1b.1	NCC	FALSE
Focad	NCC	FALSE
Psmc5	NCC	FALSE
Zswim7	NCC	FALSE
Snip1	NCC	FALSE
Zpr1	NCC	FALSE
Cpt2	NCC	FALSE
Timm10	NCC	FALSE
Ppa2	NCC	FALSE
G2e3	NCC	FALSE
Depdc1b	NCC	FALSE
1110008L1	NCC	FALSE
9930014A1	NCC	FALSE
Thap4	NCC	FALSE
Nfe2l2	NCC	TRUE
Yy2	NCC	TRUE
Unc119b	NCC	FALSE
Mmachc	NCC	FALSE
Nfs1	NCC	FALSE
Tmem245	NCC	FALSE
Tspan9	NCC	FALSE
Wdr48	NCC	FALSE
Zfp442	NCC	TRUE
Traf6	NCC	FALSE
Gnl2	NCC	FALSE
Taf1b	NCC	FALSE
Gm13157	NCC	FALSE
Map3k4	NCC	FALSE
Arv1	NCC	FALSE
Ube2k	NCC	TRUE
Tmem37	NCC	FALSE
Got1	NCC	TRUE
Gnb1l	NCC	FALSE
Dym	NCC	FALSE
Ccrn4l	NCC	FALSE
Ankrd32	NCC	FALSE
Ddx28	NCC	FALSE
Ddx19b	NCC	FALSE
Gtpbp8	NCC	FALSE
Ints10	NCC	FALSE
Glmn	NCC	FALSE

Fam107b	NCC	FALSE
Smug1	NCC	TRUE
Xrcc3	NCC	FALSE
Dcc	NCC	FALSE
Gtf2f2	NCC	FALSE
Arxes2	NCC	FALSE
Thoc5	NCC	FALSE
Cops7a	NCC	FALSE
Lrrc47	NCC	FALSE
Slit1	NCC	FALSE
Get4	NCC	FALSE
9230110C1	NCC	FALSE
Egfl7	NCC	FALSE
Snord52	NCC	FALSE
Trim37	NCC	FALSE
Cblb	NCC	FALSE
Dusp9	NCC	FALSE
Mettl4	NCC	FALSE
Recql	NCC	FALSE
Coa7	NCC	FALSE
Fem1c	NCC	FALSE
Tarbp2	NCC	FALSE
Zfp280d	NCC	TRUE
Faf1	NCC	FALSE
Txnrd3	NCC	FALSE
Lsm3	NCC	FALSE
Dbp	NCC	TRUE
Apex2	NCC	TRUE
Rngtt	NCC	FALSE
Cenpj	NCC	FALSE
Papd5	NCC	FALSE
Tonsl	NCC	FALSE
Ogfod1	NCC	FALSE
Rabl2	NCC	FALSE
Pou2f1	NCC	TRUE
Rmdn3	NCC	FALSE
Cenpl	NCC	FALSE
Hspa5	NCC	TRUE
Vrk2	NCC	FALSE
Prom1	NCC	FALSE
Bid	NCC	FALSE
Coq3	NCC	FALSE
Nup210	NCC	FALSE
Bcs1l	NCC	FALSE
Pctp	NCC	FALSE
Hmgxb3	NCC	FALSE
Ercc2	NCC	FALSE
Epb4.1	NCC	FALSE
Abl2	NCC	FALSE
Flywch1	NCC	FALSE

Txnrd2	NCC	FALSE
Actr3	NCC	FALSE
Ddx24	NCC	FALSE
Atp5b	NCC	FALSE
Helq	NCC	FALSE
Hnrnpa1	NCC	FALSE
Sirt1	NCC	FALSE
Ube2i	NCC	FALSE
Got2	NCC	FALSE
Ercc6l2	NCC	FALSE
Diexf	NCC	FALSE
Cox10	NCC	FALSE
Hspbap1	NCC	FALSE
Osgin2	NCC	FALSE
Steap1	NCC	FALSE
Sdhaf2	NCC	FALSE
L3mbtl3	NCC	FALSE
Rad9a	NCC	FALSE
Cox15	NCC	FALSE
Larp4b	NCC	FALSE
Tti2	NCC	FALSE
Det1	NCC	FALSE
Zfp239	NCC	TRUE
Dlat	NCC	FALSE
Rexo2	NCC	FALSE
Cep72	NCC	FALSE
Zfp131	NCC	TRUE
Dtnb	NCC	FALSE
Anpep	NCC	FALSE
Xab2	NCC	FALSE
Zfp143	NCC	TRUE
Ptcd2	NCC	FALSE
Tyw3	NCC	FALSE
Mrrf	NCC	FALSE
Smarca5-ps	NCC	FALSE
Gnl1	NCC	FALSE
Zbtb16	NCC	TRUE
Setx	NCC	FALSE
Tra2a	NCC	FALSE
Ilf2	NCC	TRUE
Med9	NCC	FALSE
Top1mt	NCC	FALSE
2610305D1	NCC	FALSE
Rpl5	NCC	FALSE
Dtd1	NCC	FALSE
2700062C0	NCC	FALSE
Kat7	NCC	FALSE
Atp2a2	NCC	FALSE
Pdk1	NCC	FALSE
Coq5	NCC	FALSE

Foxp2	NCC	TRUE
Alkbh8	NCC	FALSE
Llgl2	NCC	FALSE
Polr2f	NCC	FALSE
Sypl	NCC	FALSE
Ly75	NCC	FALSE
Alg3	NCC	FALSE
Med26	NCC	FALSE
Pet112	NCC	FALSE
Rad51c	NCC	FALSE
Rrn3	NCC	FALSE
Capn15	NCC	FALSE
Secisbp2	NCC	FALSE
Kdm2b	NCC	TRUE
2700099C1	NCC	FALSE
Setdb2	NCC	FALSE
Fam207a	NCC	FALSE
Gne	NCC	FALSE
2310022B0	NCC	FALSE
Farsa	NCC	FALSE
Slc35d1	NCC	FALSE
Parpbp	NCC	FALSE
Dph2	NCC	FALSE
Zfp668	NCC	TRUE
Rpusd3	NCC	FALSE
Stk38l	NCC	FALSE
Hprt	NCC	FALSE
Abcb6	NCC	FALSE
Akap1	NCC	FALSE
Aar2	NCC	FALSE
Ece1	NCC	FALSE
Ccdc90b	NCC	FALSE
Cenpo	NCC	FALSE
Trmt2a	NCC	FALSE
Fam188b	NCC	FALSE
Rnf34	NCC	FALSE
Fignl2	NCC	FALSE
Usp42	NCC	FALSE
Txnl4a	NCC	FALSE
Acsl3	NCC	FALSE
Ptch1	NCC	FALSE
Dhx34	NCC	FALSE
Dlst	NCC	FALSE
Zbtb9	NCC	FALSE
2700046A0	NCC	FALSE
Csn3	NCC	FALSE
Col4a5	NCC	FALSE
Psmc1	NCC	FALSE
Abcf1	NCC	FALSE
Telo2	NCC	FALSE

Jade3	NCC	FALSE
Nup88	NCC	FALSE
Nagpa	NCC	FALSE
Timm9	NCC	FALSE
Gnpnat1	NCC	FALSE
Morc3	NCC	FALSE
Dnajc21	NCC	TRUE
Hist1h1d	NCC	FALSE
Ttc37	NCC	FALSE
Efnb1	NCC	FALSE
Afg3l2	NCC	FALSE
Fbxo42	NCC	FALSE
Fktn	NCC	FALSE
Crybg3	NCC	FALSE
Zfp646	NCC	TRUE
Tenm4	NCC	FALSE
Stk35	NCC	FALSE
Stt3b	NCC	FALSE
1600002H(	NCC	FALSE
Tfap2c	NCC	TRUE
Etnk1	NCC	FALSE
Cldn12	NCC	FALSE
Csrnp3	NCC	FALSE
Polh	NCC	FALSE
Pde7a	NCC	FALSE
Ddi2	NCC	FALSE
Znhit3	NCC	FALSE
Hace1	NCC	FALSE
Taf5	NCC	FALSE
Simc1	NCC	FALSE
Setd6	NCC	FALSE
Pdcd7	NCC	FALSE
Has2	NCC	FALSE
Tmem209	NCC	FALSE
N4bp3	NCC	FALSE
Slc11a2	NCC	FALSE
Akip1	NCC	FALSE
Slc35a3	NCC	FALSE
Hist1h4d	NCC	FALSE
Def8	NCC	FALSE
Cdkal1	NCC	FALSE
Snord22	NCC	FALSE
Slc38a4	NCC	FALSE
Ippk	NCC	FALSE
Kpna6	NCC	FALSE
Tbp	NCC	TRUE
Pars2	NCC	FALSE
Ccbl2	NCC	FALSE
Rpl24	NCC	FALSE
Txlng	NCC	FALSE

Ubxn2b	NCC	FALSE
Ero1l	NCC	FALSE
Snapc4	NCC	TRUE
Brpf1	NCC	FALSE
Cdk5rap1	NCC	FALSE
Btbd19	NCC	FALSE
Kif24	NCC	FALSE
Opn3	NCC	FALSE
Nudt19	NCC	FALSE
Fam169a	NCC	FALSE
Thap11	NCC	TRUE
Sh2b2	NCC	FALSE
Supt3	NCC	FALSE
Lect1	NCC	FALSE
Prosc	NCC	FALSE
Ccnt1	NCC	FALSE
Ube2n	NCC	FALSE
Snord47	NCC	FALSE
Traf2	NCC	FALSE
Slc35c1	NCC	FALSE
Atp5sl	NCC	FALSE
1110004E0	NCC	FALSE
Pcdh8	NCC	FALSE
Mdm4	NCC	FALSE
Mpped1	NCC	FALSE
Sf3a2	NCC	FALSE
Trpc4ap	NCC	FALSE
Pgm2	NCC	FALSE
Fam101a	NCC	FALSE
Rragd	NCC	FALSE
Eral1	NCC	FALSE
Zcchc4	NCC	FALSE
Snrnp25	NCC	FALSE
Letm1	NCC	FALSE
Uqcc1	NCC	FALSE
Nomo1	NCC	FALSE
Sssca1	NCC	FALSE
Gpr89	NCC	FALSE
Xrn2	NCC	FALSE
Lancl2	NCC	FALSE
Wdr89	NCC	FALSE
Pif1	NCC	FALSE
Senp8	NCC	FALSE
Ecsit	NCC	TRUE
Ccdc163	NCC	FALSE
Mgmt	NCC	FALSE
Mib1	NCC	FALSE
Ube2v2	NCC	FALSE
Actr6	NCC	FALSE
Usp54	NCC	FALSE

Riok1	NCC	FALSE
Polrmt	NCC	FALSE
Rmi1	NCC	FALSE
Snora64	NCC	FALSE
Angel1	NCC	FALSE
Hiat1	NCC	FALSE
Trim6	NCC	FALSE
Khdrbs1	NCC	FALSE
Zfp869	NCC	TRUE
Pcx	NCC	FALSE
Mmgt1	NCC	FALSE
Slx4	NCC	FALSE
Cnksr3	NCC	FALSE
Gpbp1	NCC	FALSE
Casp9	NCC	FALSE
Sestd1	NCC	FALSE
Draxin	NCC	FALSE
Snx8	NCC	FALSE
Fam72a	NCC	FALSE
Tmem110	NCC	FALSE
Slc46a1	NCC	FALSE
Mtpn	NCC	FALSE
Zfp12	NCC	TRUE
Fbl	NCC	FALSE
Gtf3c5	NCC	TRUE
Pnpo	NCC	FALSE
Mrps31	NCC	FALSE
Ptn	NCC	FALSE
Csnk2a2	NCC	FALSE
Sigmar1	NCC	FALSE
Hoxc4	NCC	TRUE
Nln	NCC	FALSE
Rpap2	NCC	FALSE
Zfp566	NCC	TRUE
Pcyt1a	NCC	FALSE
Rpn2	NCC	FALSE
Pacrgl	NCC	FALSE
Tbl2	NCC	FALSE
Caprin2	NCC	FALSE
Lrrc14	NCC	FALSE
Eml3	NCC	FALSE
Fscn1	NCC	FALSE
Far1	NCC	FALSE
Srrm1	NCC	FALSE
Alkbh4	NCC	FALSE
Nkx1-2	NCC	TRUE
Zfp251	NCC	TRUE
Nup35	NCC	FALSE
Kank1	NCC	FALSE
Pdcd2	NCC	FALSE

Phf3	NCC	FALSE
1110057K0	NCC	FALSE
Zfp407	NCC	TRUE
Mettl8	NCC	FALSE
Mob1b	NCC	FALSE
Eftud1	NCC	FALSE
Agk	NCC	FALSE
Cdpf1	NCC	FALSE
Zbtb43	NCC	TRUE
Neil3	NCC	FALSE
Psmb7	NCC	FALSE
Emc6	NCC	FALSE
Zfp7	NCC	FALSE
Trdmt1	NCC	FALSE
Rassf2	NCC	FALSE
Bloc1s4	NCC	FALSE
Fzd10	NCC	FALSE
Rtn4ip1	NCC	FALSE
AK010878	NCC	FALSE
Exoc5	NCC	FALSE
Reps1	NCC	FALSE
Glud1	NCC	FALSE
Hoxc10	NCC	TRUE
Ythdc2	NCC	FALSE
Vps36	NCC	FALSE
Fmr1	NCC	FALSE
Diap3	NCC	FALSE
Crb2	NCC	FALSE
Zbtb48	NCC	TRUE
Foxk2	NCC	TRUE
Slc25a30	NCC	FALSE
Hist1h1e	NCC	FALSE
Gapvd1	NCC	FALSE
Fxn	NCC	FALSE
Pla2g12a	NCC	FALSE
Ubqln1	NCC	FALSE
Akr1c13	NCC	FALSE
Cbwd1	NCC	FALSE
Pdhx	NCC	FALSE
Suox	NCC	FALSE
Irx3	NCC	TRUE
Cep89	NCC	FALSE
Ssx2ip	NCC	FALSE
Spice1	NCC	FALSE
Zfp367	NCC	TRUE
Sp2	NCC	TRUE
Bcorl1	NCC	FALSE
Dhps	NCC	FALSE
Gm9833	NCC	TRUE
2310057M	NCC	FALSE

Zfp142	NCC	FALSE
Strbp	NCC	FALSE
C130026L2	NCC	FALSE
Slc25a38	NCC	FALSE
Batf3	NCC	TRUE
Nkain1	NCC	FALSE
Pithd1	NCC	FALSE
Tmem186	NCC	FALSE
Odf2l	NCC	FALSE
Relt	NCC	FALSE
Gmds	NCC	FALSE
Plagl1	NCC	TRUE
Tasp1	NCC	FALSE
Nedd1	NCC	FALSE
Cdkn2aipnl	NCC	FALSE
Usp44	NCC	FALSE
Pfkfb3	NCC	FALSE
Taf1a	NCC	TRUE
Gmeb2	NCC	TRUE
Pcbp4	NCC	FALSE
Jrk	NCC	FALSE
Cep57l1	NCC	FALSE
Rbm8a	NCC	FALSE
Dnajc27	NCC	FALSE
Sacm1l	NCC	FALSE
Mfsd7b	NCC	FALSE
Exoc1	NCC	FALSE
Pnp	NCC	FALSE
Mira	NCC	FALSE
Rhpn1	NCC	FALSE
Mphosph8	NCC	FALSE
Sap18	NCC	FALSE
Surf6	NCC	FALSE
Mettl21a	NCC	FALSE
Pwwp2a	NCC	FALSE
Vash2	NCC	FALSE
C330006A1	NCC	FALSE
Fgfbp3	NCC	FALSE
Tnfaip8	NCC	FALSE
Prmt10	NCC	FALSE
Comtd1	NCC	FALSE
Tpd52l1	NCC	FALSE
Ing2	NCC	FALSE
Zfp932	NCC	TRUE
Eif5a2	NCC	TRUE
Pepd	NCC	FALSE
Ndufaf5	NCC	FALSE
Ermp1	NCC	FALSE
Fgfr4	NCC	FALSE
Polr3a	NCC	TRUE

Exog	NCC	FALSE
Chka	NCC	FALSE
Tusc2	NCC	FALSE
Rptor	NCC	FALSE
Trmt12	NCC	FALSE
9430038I0:	NCC	FALSE
Nt5dc1	NCC	FALSE
Pex5	NCC	FALSE
Sh3d19	NCC	FALSE
Phf7	NCC	FALSE
Ubqln2	NCC	FALSE
Dab2	NCC	TRUE
Psmc2	NCC	TRUE
Txn2	NCC	FALSE
Zfyve20	NCC	FALSE
Cited2	NCC	FALSE
Tnks	NCC	FALSE
Rasl11b	NCC	FALSE
Atp2b1	NCC	FALSE
Eif6	NCC	FALSE
Ror2	NCC	FALSE
Ddx49	NCC	FALSE
Tnfrsf22	NCC	FALSE
Rc3h1	NCC	FALSE
Cops8	NCC	FALSE
Ghitm	NCC	FALSE
Zfp777	NCC	TRUE
Ybey	NCC	FALSE
Slc5a3	NCC	FALSE
Hoxc5	NCC	TRUE
Zfp937	NCC	TRUE
Rpl21	NCC	FALSE
Clybl	NCC	FALSE
Ntpcr	NCC	FALSE
Zc3h8	NCC	FALSE
Cep152	NCC	FALSE
Rnf165	NCC	FALSE
Coprs	NCC	FALSE
Mum1l1	NCC	FALSE
Wdr24	NCC	FALSE
Cercam	NCC	FALSE
Rbmx2	NCC	FALSE
Ccdc101	NCC	FALSE
Ptchd2	NCC	FALSE
Rps12	NCC	FALSE
Spryd7	NCC	FALSE
Cenpc1	NCC	FALSE
Meis3	NCC	TRUE
Cep97	NCC	FALSE
Fbxl18	NCC	FALSE

Slc4a1ap	NCC	FALSE
Opa3	NCC	FALSE
Grpel2	NCC	FALSE
Heph	NCC	FALSE
Tlcd1	NCC	FALSE
Usp5	NCC	FALSE
Ube3c	NCC	FALSE
Tmem69	NCC	FALSE
Abhd17b	NCC	FALSE
Zkscan5	NCC	TRUE
Snora70	NCC	FALSE
Ccdc134	NCC	FALSE
Pycr1	NCC	FALSE
Dffb	NCC	FALSE
Fam101b	NCC	FALSE
Cog2	NCC	FALSE
Dhcr24	NCC	FALSE
Tmem39b	NCC	FALSE
Brms1	NCC	FALSE
Glul	NCC	FALSE
5031439G(	NCC	FALSE
Ctnnbl1	NCC	FALSE
D230025D:	NCC	FALSE
Ap4b1	NCC	FALSE
Hoxd10	NCC	TRUE
Ttpa	NCC	FALSE
Papolg	NCC	FALSE
Dlc1	NCC	FALSE
Eef1e1	NCC	FALSE
Gss	NCC	FALSE
Ivd	NCC	TRUE
Sgpp2	NCC	FALSE
Grb7	NCC	FALSE
Impdh1	NCC	FALSE
Arhgef39	NCC	FALSE
Kdm8	NCC	FALSE
Cmtr2	NCC	FALSE
BC064078	NCC	FALSE
Gopc	NCC	FALSE
Klhl25	NCC	FALSE
Pcsk5	NCC	FALSE
Ap1g2	NCC	FALSE
Phykpl	NCC	FALSE
Ptar1	NCC	FALSE
Ubxn7	NCC	FALSE
Zbtb5	NCC	TRUE
Ccdc40	NCC	FALSE
Ammecr1	NCC	FALSE
Zfp386	NCC	TRUE
Eif3g	NCC	FALSE

Vps35	NCC	FALSE
Rhbdd3	NCC	FALSE
Ttll5	NCC	FALSE
6720489N1	NCC	TRUE
Asb13	NCC	FALSE
Eno1	NCC	FALSE
Vti1a	NCC	FALSE
Taf4a	NCC	FALSE
Alx1	NCC	TRUE
Mga	NCC	TRUE
Cdhr2	NCC	FALSE
Tsnax	NCC	TRUE
Usp48	NCC	FALSE
Prokr1	NCC	FALSE
Tagap1	NCC	FALSE
Sucla2	NCC	FALSE
Gcfc2	NCC	FALSE
Cars2	NCC	FALSE
Adarb1	NCC	TRUE
Zfp770	NCC	TRUE
Ap4s1	NCC	FALSE
Pdlim5	NCC	TRUE
Mrpl40	NCC	FALSE
Mrpl47	NCC	FALSE
Hmox1	NCC	FALSE
Atp2c1	NCC	FALSE
Pot1a	NCC	FALSE
Plcl2	NCC	FALSE
Jmjd6	NCC	FALSE
Fgfr1	NCC	FALSE
Sh3bp4	NCC	FALSE
Zbtb25	NCC	TRUE
Cops3	NCC	FALSE
Gga3	NCC	FALSE
Ift88	NCC	FALSE
Gm10857	NCC	FALSE
Exoc2	NCC	FALSE
Wdyhv1	NCC	FALSE
N6amt1	NCC	FALSE
Matn3	NCC	FALSE
Rpl26	NCC	FALSE
Wdr53	NCC	FALSE
Mbtps2	NCC	TRUE
Ctu1	NCC	FALSE
Mir1949	NCC	FALSE
Adamts20	NCC	FALSE
Taf3	NCC	FALSE
Zfp346	NCC	FALSE
Zfp13	NCC	TRUE
Smim3	NCC	FALSE

Zswim1	NCC	TRUE
Cebpg	NCC	TRUE
Snora7a	NCC	FALSE
Fancc	NCC	FALSE
Abhd5	NCC	FALSE
D3Ertd751(	NCC	FALSE
Pgrmc2	NCC	FALSE
Yrdc	NCC	FALSE
Gas8	NCC	FALSE
Endog	NCC	FALSE
Spred2	NCC	FALSE
Kin	NCC	FALSE
Diap1	NCC	FALSE
Cpne5	NCC	FALSE
Gng12	NCC	FALSE
Tomm22	NCC	FALSE
Uimc1	NCC	FALSE
Tesk2	NCC	FALSE
Mrpl46	NCC	FALSE
Tprkb	NCC	FALSE
Aldh4a1	NCC	FALSE
Tctex1d2	NCC	FALSE
Hinfp	NCC	TRUE
Tbkbp1	NCC	FALSE
Zbtb44	NCC	TRUE
Rrp7a	NCC	FALSE
1110002L0	NCC	FALSE
Mtmr9	NCC	FALSE
Nudt2	NCC	FALSE
Zfp64	NCC	TRUE
Myo1e	NCC	FALSE
Cep128	NCC	FALSE
Ap5m1	NCC	FALSE
Cep63	NCC	FALSE
Cdc42se2	NCC	FALSE
Cmtm4	NCC	FALSE
Nphp1	NCC	FALSE
Ccdc93	NCC	FALSE
Gspt2	NCC	FALSE
Tmem11	NCC	FALSE
Kbtbd7	NCC	FALSE
Khnyn	NCC	FALSE
Zmpste24	NCC	FALSE
Iqcc	NCC	FALSE
Zfp689	NCC	TRUE
Mnd1-ps	NCC	FALSE
Zak	NCC	FALSE
Psmd6	NCC	FALSE
Polg2	NCC	FALSE
Ftsj2	NCC	FALSE

Pmm2	NCC	FALSE
Brd8	NCC	FALSE
Hoxc6	NCC	TRUE
Ptprk	NCC	FALSE
Psma3	NCC	FALSE
Fam69a	NCC	FALSE
Med22	NCC	FALSE
Mrpl38	NCC	FALSE
Rpusd1	NCC	FALSE
R3hcc1	NCC	FALSE
Avpi1	NCC	FALSE
Hoxa10	NCC	TRUE
Xpnpep3	NCC	FALSE
Lmx1a	NCC	TRUE
Ino80	NCC	FALSE
Amz2	NCC	FALSE
Ppp4r4	NCC	FALSE
Wdr62	NCC	FALSE
Pdcd6ip	NCC	FALSE
Pex10	NCC	FALSE
Nfkbib	NCC	FALSE
Rlf	NCC	TRUE
Zfp84	NCC	FALSE
Mrps5	NCC	FALSE
Aste1	NCC	FALSE
Mto1	NCC	FALSE
Brwd3	NCC	FALSE
Taf15	NCC	FALSE
LOC101056	NCC	FALSE
Zfp830	NCC	TRUE
Sowahc	NCC	FALSE
Six4	NCC	TRUE
Mstn	NCC	FALSE
Vcpip1	NCC	FALSE
Lin9	NCC	FALSE
Rpl10	NCC	FALSE
S100pbp	NCC	FALSE
D030056L2	NCC	FALSE
Lsm11	NCC	FALSE
Cluap1	NCC	FALSE
Ddx59	NCC	FALSE
Tssc1	NCC	FALSE
Ptges2	NCC	FALSE
Alg9	NCC	FALSE
Gmppb	NCC	FALSE
Hibch	NCC	FALSE
Stx18	NCC	FALSE
5730455P1	NCC	FALSE
Wars2	NCC	FALSE
Nkrf	NCC	FALSE

Srl	NCC	FALSE
Atp7a	NCC	FALSE
Idh3a	NCC	FALSE
Zfp622	NCC	TRUE
Pin1	NCC	FALSE
Mcoln3	NCC	FALSE
Car13	NCC	FALSE
Trim23	NCC	FALSE
Banf1	NCC	FALSE
Polr3h	NCC	FALSE
Xylb	NCC	FALSE
Pcf11	NCC	FALSE
Ube2o	NCC	FALSE
Serinc5	NCC	FALSE
Primpol	NCC	FALSE
Mtmr14	NCC	FALSE
1700008J0	NCC	FALSE
Gltpd1	NCC	FALSE
Dus2	NCC	FALSE
Rnf126	NCC	FALSE
Aprt	NCC	FALSE
Shroom3	NCC	FALSE
Naa30	NCC	FALSE
Ccnk	NCC	FALSE
Rhebl1	NCC	FALSE
Tbrg3	NCC	FALSE
Lin52	NCC	FALSE
Zfp146	NCC	TRUE
Atp13a1	NCC	FALSE
Fam149b	NCC	FALSE
Acsl5	NCC	FALSE
Srd5a3	NCC	FALSE
Adipor2	NCC	FALSE
Bmp5	NCC	FALSE
Rpgr	NCC	FALSE
Prune	NCC	FALSE
Sec24a	NCC	FALSE
Mapk12	NCC	FALSE
Cnpy3	NCC	FALSE
Cep250	NCC	FALSE
Sik2	NCC	FALSE
Tbcd	NCC	FALSE
Zfp958	NCC	TRUE
Pla2g7	NCC	FALSE
Morc2a	NCC	FALSE
Zbtb21	NCC	FALSE
Kif5b	NCC	FALSE
Icmt	NCC	FALSE
Camsap2	NCC	FALSE
Tmem161b	NCC	FALSE

Zfp808	NCC	FALSE
Fam98b	NCC	FALSE
Traf3ip1	NCC	FALSE
Jade1	NCC	FALSE
Wipf2	NCC	FALSE
Nudt11	NCC	FALSE
Trmt13	NCC	FALSE
Actr1b	NCC	FALSE
Rpl9	NCC	FALSE
Gpatch3	NCC	FALSE
Pcgf3	NCC	FALSE
Zbtb46	NCC	TRUE
Tbc1d5	NCC	FALSE
Galm	NCC	FALSE
Ppp1r14a	NCC	FALSE
Slc25a46	NCC	FALSE
Hist2h2ac	NCC	FALSE
BC048403	NCC	FALSE
Hoxa7	NCC	TRUE
Tmem70	NCC	FALSE
Nrbf2	NCC	FALSE
Ift80	NCC	FALSE
Iqcb1	NCC	FALSE
4930519F0	NCC	FALSE
Ttc26	NCC	FALSE
Gosr2	NCC	FALSE
Bmi1	NCC	FALSE
Zfp472	NCC	FALSE
Gla	NCC	FALSE
Trmt61b	NCC	FALSE
Cutc	NCC	FALSE
Trp53rk	NCC	FALSE
Otulin	NCC	FALSE
Adat3	NCC	FALSE
Raf1	NCC	FALSE
Zfp101	NCC	FALSE
Grpel1	NCC	FALSE
Alas1	NCC	FALSE
Gm16973	NCC	FALSE
Brf2	NCC	TRUE
Cnot7	NCC	FALSE
Ufsp2	NCC	FALSE
Mtg1	NCC	FALSE
Baz2a	NCC	FALSE
Fam213a	NCC	FALSE
Recql5	NCC	FALSE
Tbc1d22b	NCC	FALSE
Elmo1	NCC	FALSE
Nf2	NCC	FALSE
Meis1	NCC	TRUE

Nrp2	NCC	FALSE
Mkrn2	NCC	FALSE
Ap4m1	NCC	FALSE
Nsf	NCC	FALSE
Bdp1	NCC	TRUE
Gtf3a	NCC	TRUE
2310061I0	NCC	FALSE
Plekhf2	NCC	FALSE
Fkrp	NCC	FALSE
Nsfl1c	NCC	FALSE
Zfp800	NCC	TRUE
BC068281	NCC	FALSE
Zbtb39	NCC	TRUE
Rce1	NCC	FALSE
Intu	NCC	FALSE
Timm23	NCC	FALSE
Ftsj1	NCC	FALSE
Zdhhc16	NCC	FALSE
Lztfl1	NCC	FALSE
Nol12	NCC	FALSE
2410127L1	NCC	FALSE
Ttc7b	NCC	FALSE
Sepsecs	NCC	FALSE
Nop10	NCC	FALSE
Atxn3	NCC	FALSE
C230052I1	NCC	FALSE
Thap1	NCC	TRUE
Bbs12	NCC	FALSE
Zfp655	NCC	TRUE
Zfp942	NCC	FALSE
Ino80e	NCC	FALSE
Slc10a3	NCC	FALSE
Adal	NCC	FALSE
Fv1	NCC	FALSE
Gm608	NCC	FALSE
Vdac1	NCC	FALSE
Hoxb1	NCC	TRUE
Cdkn3	NCC	FALSE
Abhd11os	NCC	FALSE
Mks1	NCC	FALSE
Mars2	NCC	FALSE
Gba2	NCC	FALSE
Qsox2	NCC	FALSE
Lnp	NCC	FALSE
Lins	NCC	FALSE
Alkbh2	NCC	FALSE
Slc15a4	NCC	FALSE
Samd1	NCC	FALSE
Prkacb	NCC	FALSE
Micall1	NCC	FALSE

Trappc8	NCC	FALSE
Dus4l	NCC	FALSE
3110009E1	NCC	FALSE
Tchp	NCC	FALSE
Srf	NCC	TRUE
Med13l	NCC	FALSE
Chst12	NCC	FALSE
Cpn1	NCC	FALSE
Plekhj1	NCC	FALSE
Ebag9	NCC	FALSE
Cyb5r4	NCC	FALSE
Rwdd2b	NCC	FALSE
Cmtm6	NCC	FALSE
Ccdc138	NCC	FALSE
Pop5	NCC	FALSE
Emilin2	NCC	FALSE
Ppp1r16a	NCC	FALSE
Cbr4	NCC	FALSE
Mlh3	NCC	FALSE
Klf16	NCC	TRUE
Zbtb33	NCC	TRUE
Actr5	NCC	FALSE
Prkar2b	NCC	FALSE
Phospho2	NCC	FALSE
Sac3d1	NCC	FALSE
Mbip	NCC	FALSE
Stxbp6	NCC	FALSE
Pir	NCC	TRUE
Tmem199	NCC	FALSE
Surf2	NCC	FALSE
Gorab	NCC	FALSE
Dyrk2	NCC	FALSE
Daglb	NCC	FALSE
Ufl1	NCC	FALSE
Bpnt1	NCC	FALSE
Zfp760	NCC	TRUE
Uhrf1bp1l	NCC	FALSE
Snhg11	NCC	FALSE
Camsap1	NCC	FALSE
Dtwd2	NCC	FALSE
Spata5l1	NCC	FALSE
Ppp1r1a	NCC	FALSE
Mrs2	NCC	FALSE
Wscd1	NCC	FALSE
Golga3	NCC	FALSE
Nhej1	NCC	FALSE
Zfp81	NCC	FALSE
Pigo	NCC	FALSE
BC037034	NCC	FALSE
Zfp39	NCC	TRUE

Eno3	NCC	FALSE
Slc5a6	NCC	FALSE
Fancf	NCC	FALSE
Adat2	NCC	FALSE
Rhobtb2	NCC	FALSE
Taf1c	NCC	FALSE
8030462N1	NCC	FALSE
Mtif3	NCC	FALSE
Cep290	NCC	FALSE
Chchd6	NCC	FALSE
Map4k1	NCC	FALSE
Ascc3	NCC	FALSE
Elac1	NCC	FALSE
Papd7	NCC	FALSE
Tbc1d4	NCC	FALSE
Harbi1	NCC	FALSE
Crnde	NCC	FALSE
Pkn3	NCC	FALSE
Lpcat3	NCC	FALSE
Polr2c	NCC	FALSE
Mipol1	NCC	FALSE
Phtf2	NCC	FALSE
Zbtb22	NCC	TRUE
Ep300	NCC	TRUE
Vps33b	NCC	FALSE
Prdm15	NCC	TRUE
Bbs7	NCC	FALSE
Ranbp6	NCC	FALSE
Bcl7c	NCC	FALSE
Mapre1	NCC	FALSE
Thap6	NCC	FALSE
Zyg11b	NCC	FALSE
Slc1a4	NCC	FALSE
Snord12	NCC	FALSE
Utp14b	NCC	FALSE
Neo1	NCC	FALSE
A830080D(	NCC	FALSE
Lrp3	NCC	FALSE
Socs4	NCC	TRUE
Zfp825	NCC	TRUE
Mtfp1	NCC	FALSE
Pcbd1	NCC	FALSE
Nmnat1	NCC	FALSE
Cldn9	NCC	FALSE
Cep95	NCC	FALSE
Zfp202	NCC	TRUE
Mul1	NCC	FALSE
4930539J0.	NCC	FALSE
Tpst1	NCC	FALSE
Arhgap12	NCC	FALSE

Tmem243	NCC	FALSE
Gadd45a	NCC	TRUE
Gas2	NCC	FALSE
Cycs	NCC	TRUE
Cdr2	NCC	FALSE
Vegfc	NCC	FALSE
Taco1	NCC	FALSE
Fahd1	NCC	FALSE
Clk3	NCC	FALSE
Blzf1	NCC	FALSE
Cep104	NCC	FALSE
Abcc8	NCC	FALSE
Camk2d	NCC	FALSE
Vps18	NCC	FALSE
Rab31	NCC	FALSE
Ncln	NCC	FALSE
Katnb1	NCC	FALSE
Ptp4a1	NCC	FALSE
Sfxn1	NCC	FALSE
Prcc	NCC	FALSE
Slc29a2	NCC	FALSE
Lrrfip2	NCC	FALSE
Mtg2	NCC	FALSE
Nipsnap3b	NCC	FALSE
Stam2	NCC	FALSE
Ccdc91	NCC	FALSE
Smyd4	NCC	FALSE
Gpank1	NCC	TRUE
AW209491	NCC	FALSE
Rfesd	NCC	FALSE
Wdr20	NCC	FALSE
Gm10033	NCC	FALSE
Fert2	NCC	FALSE
Ccdc115	NCC	FALSE
Grip1	NCC	FALSE
Eif4a1	NCC	FALSE
Crcp	NCC	FALSE
Car4	NCC	FALSE
Ercc5	NCC	FALSE
Zfp1	NCC	TRUE
Prkar2a	NCC	FALSE
Tspan5	NCC	FALSE
Rabif	NCC	FALSE
Tatdn1	NCC	FALSE
Hebp1	NCC	FALSE
Bhlhb9	NCC	FALSE
Hoxc8	NCC	TRUE
Stam	NCC	FALSE
Arl6ip4	NCC	FALSE
Ptcd1	NCC	TRUE

Tomm40l	NCC	FALSE
Fdxr	NCC	FALSE
Steap3	NCC	FALSE
Ddt	NCC	FALSE
Pex12	NCC	FALSE
Slain2	NCC	FALSE
Ipp	NCC	FALSE
Aplf	NCC	FALSE
Taf13	NCC	FALSE
Kras	NCC	FALSE
Guk1	NCC	FALSE
Cdh15	NCC	FALSE
Mcm9	NCC	FALSE
Asb6	NCC	FALSE
Arl6	NCC	FALSE
Cwf19l2	NCC	FALSE
Amacr	NCC	FALSE
Tbk1	NCC	FALSE
Zfp592	NCC	TRUE
Mtrf1	NCC	FALSE
Uxs1	NCC	FALSE
Scrn3	NCC	FALSE
Zfp934	NCC	FALSE
Rbm48	NCC	FALSE
Vps33a	NCC	FALSE
Hist1h1a	NCC	FALSE
B3galt6	NCC	FALSE
Zbtb45	NCC	TRUE
Esyt2	NCC	FALSE
Armc6	NCC	FALSE
Trabd	NCC	FALSE
Wdr1	NCC	FALSE
Ppp1r2	NCC	FALSE
Tmem192	NCC	FALSE
Adam9	NCC	FALSE
Zfp652	NCC	TRUE
9930104L0	NCC	FALSE
Acsf3	NCC	FALSE
Cdc25b	NCC	FALSE
Zfp512	NCC	FALSE
Ppp2r2d	NCC	FALSE
Fam122b	NCC	FALSE
Cln8	NCC	FALSE
Slc9a5	NCC	FALSE
Elovl5	NCC	FALSE
Fgd6	NCC	FALSE
Med30	NCC	TRUE
Zfp511	NCC	FALSE
Cbfa2t2	NCC	TRUE
Ogdh	NCC	FALSE

Ctr9	NCC	FALSE
Sec61a2	NCC	FALSE
Snora81	NCC	FALSE
Arl8b	NCC	FALSE
Psma7	NCC	FALSE
Slc25a32	NCC	FALSE
Ccp110	NCC	FALSE
Mrps10	NCC	FALSE
Ankrd13c	NCC	FALSE
Slc38a2	NCC	FALSE
Vdac2	NCC	FALSE
Wdr35	NCC	FALSE
Pik3cb	NCC	FALSE
Prkrip1	NCC	FALSE
Max	NCC	TRUE
Bnip1	NCC	FALSE
Trmt44	NCC	FALSE
Ppp6c	NCC	FALSE
Fam188a	NCC	FALSE
Dhrs7b	NCC	FALSE
Gyk	NCC	FALSE
Trit1	NCC	FALSE
Phka2	NCC	FALSE
5430417L2	NCC	FALSE
Cd164	NCC	FALSE
Nrde2	NCC	FALSE
Sp5	NCC	TRUE
Fancb	NCC	FALSE
Ifrd1	NCC	FALSE
1700066M	NCC	FALSE
Actg1	NCC	FALSE
Dcaf15	NCC	FALSE
Mtfmt	NCC	FALSE
Tldc1	NCC	FALSE
Fam35a	NCC	FALSE
Btbd7	NCC	FALSE
Plekha3	NCC	FALSE
2610015P0	NCC	FALSE
Gnl3l	NCC	FALSE
Mtx3	NCC	FALSE
Zfp820	NCC	FALSE
Cep162	NCC	FALSE
Tnfrsf21	NCC	FALSE
Chchd3	NCC	FALSE
Dusp19	NCC	FALSE
Slc27a3	NCC	FALSE
Mrc2	NCC	FALSE
D430020J0	NCC	FALSE
Cdh11	NCC	FALSE
Sptlc2	NCC	FALSE

AY074887	NCC	FALSE
Pik3c3	NCC	TRUE
Pptc7	NCC	FALSE
2210015D1	NCC	FALSE
Dcun1d2	NCC	FALSE
Arl6ip1	NCC	FALSE
Ccdc15	NCC	FALSE
Cask	NCC	FALSE
Rtkn2	NCC	FALSE
Plekha7	NCC	FALSE
Fgd4	NCC	FALSE
Slc9a1	NCC	FALSE
Tmem206	NCC	FALSE
Znhit2	NCC	FALSE
Zfp113	NCC	TRUE
2010015L0	NCC	FALSE
Pxmp2	NCC	FALSE
1110004F1	NCC	FALSE
Kctd2	NCC	FALSE
Mob2	NCC	FALSE
Alg12	NCC	FALSE
Dolpp1	NCC	FALSE
Sipa1l3	NCC	FALSE
Tmem41a	NCC	FALSE
Crbn	NCC	FALSE
Fhod3	NCC	FALSE
Ldlrad3	NCC	FALSE
Lactb	NCC	FALSE
Dtnbp1	NCC	FALSE
Tmem177	NCC	FALSE
Gbe1	NCC	FALSE
Ube2cbp	NCC	FALSE
Ggps1	NCC	FALSE
Armc9	NCC	FALSE
3000002C1	NCC	FALSE
Zfp763	NCC	FALSE
Fez2	NCC	FALSE
Aasdh	NCC	FALSE
Sep-06	NCC	FALSE
Wwp1	NCC	FALSE
Acsl1	NCC	FALSE
Rrp1	NCC	FALSE
Thap2	NCC	FALSE
Gm15455	NCC	FALSE
Phf8	NCC	TRUE
Chst1	NCC	FALSE
Fem1a	NCC	FALSE
9630033F2	NCC	FALSE
Fam208a	NCC	FALSE
Fnbp4	NCC	FALSE

Pak1ip1	NCC	FALSE
Lca5	NCC	FALSE
Cspp1	NCC	FALSE
Foxj3	NCC	TRUE
2810021J2	NCC	FALSE
Pitpnm2	NCC	FALSE
Etohi1	NCC	FALSE
Dnajc15	NCC	FALSE
Psmd4	NCC	FALSE
Brap	NCC	FALSE
Mfn1	NCC	FALSE
Dnajb1	NCC	FALSE
Vps26b	NCC	FALSE
Fancg	NCC	FALSE
Txnl4b	NCC	FALSE
Tnfsf9	NCC	FALSE
Vill	NCC	FALSE
Ankrd9	NCC	FALSE
Hoxd1	NCC	TRUE
Commd10	NCC	FALSE
Fam192a	NCC	FALSE
Gfod2	NCC	FALSE
Fat3	NCC	FALSE
Actb	NCC	FALSE
Dpcd	NCC	FALSE
Pank1	NCC	FALSE
Stub1	NCC	TRUE
Trp53bp2	NCC	FALSE
Gdpgp1	NCC	FALSE
Acad11	NCC	FALSE
Chml	NCC	FALSE
Ccl25	NCC	FALSE
Gpi1	NCC	FALSE
Raet1e	NCC	FALSE
Asxl3	NCC	FALSE
Vezf1	NCC	TRUE
Tmem126b	NCC	FALSE
Ogfod3	NCC	FALSE
Hpcal1	NCC	FALSE
Pi4kb	NCC	FALSE
Rccd1	NCC	FALSE
Angptl1	NCC	FALSE
Artn	NCC	FALSE
Gm13154	NCC	FALSE
Ino80c	NCC	FALSE
Nubp2	NCC	FALSE
Dennd5b	NCC	FALSE
C230091D(	NCC	FALSE
Zhx1	NCC	TRUE
Pgp	NCC	FALSE

Tmem132a	NCC	FALSE
Pvrl1	NCC	FALSE
Sema3a	NCC	FALSE
Aimp1	NCC	FALSE
Eif2b4	NCC	FALSE
Ap4e1	NCC	FALSE
Gpatch1	NCC	FALSE
Lama5	NCC	FALSE
Cep41	NCC	FALSE
Cyc1	NCC	FALSE
Snord69	NCC	FALSE
Zfp180	NCC	TRUE
Rint1	NCC	FALSE
Ak2	NCC	FALSE
Tmem135	NCC	FALSE
Scyl2	NCC	FALSE
Arhgap32	NCC	FALSE
Bbs5	NCC	FALSE
Slc41a1	NCC	FALSE
Dnajc5	NCC	FALSE
Inpp4b	NCC	FALSE
Rabggta	NCC	FALSE
Zfp335	NCC	TRUE
Pth1r	NCC	FALSE
Hdx	NCC	TRUE
Zfp454	NCC	TRUE
4632415L0	NCC	FALSE
Syde2	NCC	FALSE
Lcmt2	NCC	FALSE
Safb	NCC	FALSE
C2cd5	NCC	FALSE
Slitrk5	NCC	FALSE
Pradc1	NCC	FALSE
Dohh	NCC	FALSE
Gatc	NCC	FALSE
Uap1	NCC	FALSE
Cops5	NCC	FALSE
Mocs3	NCC	FALSE
Med11	NCC	FALSE
Meox1	NCC	TRUE
Atxn7l3	NCC	FALSE
Zfp946	NCC	FALSE
Btbd6	NCC	FALSE
Sav1	NCC	FALSE
9930012K1	NCC	FALSE
4933427D1	NCC	FALSE
Hoxd11	NCC	TRUE
Slc52a2	NCC	FALSE
Ccdc171	NCC	FALSE
Casp7	NCC	FALSE

Mkrn3	NCC	FALSE
2810002D1	NCC	FALSE
Prepl	NCC	FALSE
4931414P1	NCC	FALSE
Pappa	NCC	FALSE
Mospd1	NCC	FALSE
Atg7	NCC	FALSE
Mrpl55	NCC	FALSE
Zfp110	NCC	TRUE
Lrch2	NCC	FALSE
Irak1bp1	NCC	FALSE
Alkbh7	NCC	FALSE
Gls	NCC	FALSE
Slc16a13	NCC	FALSE
Zranb1	NCC	FALSE
Ptk2	NCC	FALSE
Ermard	NCC	FALSE
Doc2a	NCC	FALSE
Zfp944	NCC	FALSE
Stc2	NCC	FALSE
Snord100	NCC	FALSE
Zfp449	NCC	TRUE
Adar	NCC	FALSE
3110045C2	NCC	FALSE
Zfp955a	NCC	FALSE
Leprel1	NCC	FALSE
Pnkd	NCC	FALSE
Ppcdc	NCC	FALSE
Fkbpl	NCC	FALSE
Lig4	NCC	FALSE
Ppp1r10	NCC	TRUE
Zfp647	NCC	TRUE
Card10	NCC	FALSE
D930016D(	NCC	FALSE
Acer3	NCC	FALSE
Slc7a1	NCC	FALSE
Zbtb10	NCC	FALSE
Ankfy1	NCC	FALSE
9130023H2	NCC	TRUE
Prelid1	NCC	FALSE
Stk17b	NCC	FALSE
Dixdc1	NCC	FALSE
Zbtb40	NCC	TRUE
Amotl2	NCC	FALSE
Mettl20	NCC	FALSE
Rab35	NCC	FALSE
Colec12	NCC	FALSE
Zdhhc7	NCC	FALSE
Zfp719	NCC	TRUE
Paip1	NCC	FALSE

Dus3l	NCC	TRUE
9130008F2	NCC	FALSE
Suclg1	NCC	TRUE
Dpagt1	NCC	FALSE
Usp33	NCC	FALSE
Pnpla7	NCC	FALSE
Gpd1l	NCC	FALSE
Zfp748	NCC	FALSE
Lysmd3	NCC	FALSE
Arl13b	NCC	FALSE
Trim39	NCC	FALSE
2410004B1	NCC	FALSE
Cdk16	NCC	FALSE
Rbm3	NCC	TRUE
Stradb	NCC	FALSE
Pyroxd1	NCC	FALSE
Lyrm5	NCC	FALSE
Rab40c	NCC	FALSE
Zfp938	NCC	TRUE
6430573F1	NCC	FALSE
Maoa	NCC	FALSE
Rbak	NCC	TRUE
Pias4	NCC	FALSE
Fjx1	NCC	FALSE
Wfdc2	NCC	FALSE
Hps1	NCC	FALSE
Mta3	NCC	TRUE
Gtdc1	NCC	FALSE
Kcnh2	NCC	FALSE
Clpp	NCC	FALSE
Snord49a	NCC	FALSE
Zbtbd6	NCC	FALSE
5830415F0	NCC	FALSE
Tpgs2	NCC	FALSE
Pigk	NCC	FALSE
Vps11	NCC	FALSE
C87436	NCC	FALSE
Lin7c	NCC	FALSE
Dnajc25	NCC	FALSE
Ripk3	NCC	FALSE
Nfkb2	NCC	TRUE
Lsm10	NCC	FALSE
Slmo1	NCC	FALSE
Rab11fip3	NCC	FALSE
Klhdc2	NCC	FALSE
Dpy19l4	NCC	FALSE
Hgsnat	NCC	FALSE
Prdm10	NCC	TRUE
Kri1	NCC	FALSE
Zfp955b	NCC	FALSE

Yipf6	NCC	FALSE
Fbxo33	NCC	FALSE
Arfgef2	NCC	FALSE
Rhbdd2	NCC	FALSE
Mif4gd	NCC	FALSE
Wdr34	NCC	FALSE
Itm2a	NCC	FALSE
Snapc1	NCC	FALSE
2610044O1	NCC	TRUE
2410131K1	NCC	FALSE
Tmem194b	NCC	FALSE
Snord35a	NCC	FALSE
Klk8	NCC	FALSE
Kit	NCC	FALSE
Atp8b2	NCC	FALSE
Ftx	NCC	FALSE
Sirpa	NCC	FALSE
Mttp	NCC	FALSE
Ccdc94	NCC	FALSE
A630072M	NCC	FALSE
Gmcl1	NCC	FALSE
Sf3b5	NCC	FALSE
Ccdc71l	NCC	FALSE
Hspbp1	NCC	FALSE
Zc3h10	NCC	FALSE
Rabep2	NCC	FALSE
Rassf3	NCC	FALSE
Hps4	NCC	FALSE
Htt	NCC	FALSE
Ddx26b	NCC	FALSE
Atg16l1	NCC	FALSE
Ccdc51	NCC	FALSE
B3gntl1	NCC	FALSE
Rad51d	NCC	FALSE
Ofd1	NCC	FALSE
Dync2h1	NCC	FALSE
Aspscr1	NCC	TRUE
Zfp74	NCC	TRUE
Brat1	NCC	FALSE
Hpdl	NCC	FALSE
Uevld	NCC	FALSE
Chmp3	NCC	FALSE
6330416G1	NCC	FALSE
3110001I2	NCC	FALSE
Klhl2	NCC	FALSE
Raet1d	NCC	FALSE
Zfp940	NCC	FALSE
Brd2	NCC	FALSE
Mettl18	NCC	FALSE
Mettl15	NCC	FALSE

Lclat1	NCC	FALSE
Efcab7	NCC	FALSE
Nespas	NCC	FALSE
Lrrc57	NCC	FALSE
Tctn2	NCC	FALSE
Efcab11	NCC	FALSE
Acat2	NCC	FALSE
Arrdc4	NCC	FALSE
Scfd2	NCC	FALSE
Fam162a	NCC	FALSE
Ddx55	NCC	FALSE
Sh3bgrl2	NCC	FALSE
Rnpc3	NCC	FALSE
2510009E0	NCC	FALSE
Pdpr	NCC	FALSE
Zfp644	NCC	FALSE
Srd5a1	NCC	FALSE
Klhdc9	NCC	FALSE
Foxd2os	NCC	FALSE
Vegfb	NCC	FALSE
Snora33	NCC	FALSE
Snord16a	NCC	FALSE
Tomt	NCC	FALSE
Col23a1	NCC	FALSE
Vav2	NCC	FALSE
D1Ertd622	NCC	FALSE
Frmd4b	NCC	FALSE
Pak1	NCC	FALSE
Abcc1	NCC	FALSE
Wdfy2	NCC	FALSE
Casc3	NCC	FALSE
Elp5	NCC	FALSE
Cabin1	NCC	FALSE
AK129341	NCC	FALSE
Tubd1	NCC	FALSE
Pex26	NCC	FALSE
Fut11	NCC	FALSE
1700011H1	NCC	FALSE
A630012P0	NCC	FALSE
Unc5c	NCC	FALSE
2810429I0	NCC	FALSE
Cyb5rl	NCC	FALSE
Prr5l	NCC	FALSE
A230072C0	NCC	FALSE
2610002J0	NCC	FALSE
Trmt112	NCC	FALSE
Fam117b	NCC	FALSE
Nsun6	NCC	FALSE
Zfp248	NCC	TRUE
Mex3b	NCC	FALSE

Dclre1c	NCC	FALSE
Atf2	NCC	TRUE
Snora28	NCC	FALSE
Pkhd1	NCC	FALSE
Spen	NCC	FALSE
Ier5l	NCC	FALSE
Usp12	NCC	FALSE
Lrrc51	NCC	FALSE
Lpcat4	NCC	FALSE
Acvr2a	NCC	FALSE
Gdap2	NCC	FALSE
Pdik1l	NCC	FALSE
E130317F2	NCC	FALSE
Sema5b	NCC	FALSE
Usp31	NCC	FALSE
Nabp1	NCC	FALSE
Klhdc10	NCC	FALSE
Man2a1	NCC	FALSE
Rora	NCC	TRUE
Uhmk1	NCC	FALSE
Tmem189	NCC	FALSE
Bcdin3d	NCC	FALSE
Swt1	NCC	FALSE
Trp53i11	NCC	FALSE
Rab11fip1	NCC	FALSE
Heatr6	NCC	FALSE
Myo5b	NCC	FALSE
Rybp	NCC	FALSE
Zfp446	NCC	FALSE
B230217C1	NCC	FALSE
Ubac1	NCC	FALSE
Zfp787	NCC	TRUE
Fut4	NCC	FALSE
Hmbox1	NCC	TRUE
Dffa	NCC	FALSE
Sacs	NCC	FALSE
Echdc2	NCC	FALSE
Zfp788	NCC	FALSE
Selt	NCC	FALSE
Ankzf1	NCC	FALSE
Vps13b	NCC	FALSE
Trim11	NCC	FALSE
Cep19	NCC	FALSE
Higd1a	NCC	FALSE
Dnajc3	NCC	FALSE
Smpdl3b	NCC	FALSE
Slc33a1	NCC	FALSE
Kifc1	NCC	FALSE
Dnaja1	NCC	FALSE
Gpatch2l	NCC	FALSE

Efnb2	NCC	FALSE
Skida1	NCC	FALSE
8430429K0	NCC	FALSE
C030006K1	NCC	FALSE
Tmed8	NCC	FALSE
Sdf2l1	NCC	FALSE
Zfp65	NCC	FALSE
4930414L2	NCC	FALSE
Snord65	NCC	FALSE
Ppp1r13l	NCC	FALSE
Tshz3	NCC	FALSE
2210408I2	NCC	FALSE
Wnt5b	NCC	FALSE
Crispld2	NCC	FALSE
Bcap29	NCC	FALSE
Smg9	NCC	FALSE
Pdzk1	NCC	FALSE
Zfp37	NCC	TRUE
Rsad1	NCC	FALSE
9530091C0	NCC	FALSE
Adrm1	NCC	FALSE
Ncoa7	NCC	FALSE
Zfand2a	NCC	FALSE
Nudcd3	NCC	FALSE
Podxl	NCC	FALSE
Ssu72	NCC	FALSE
Zfp58	NCC	FALSE
Sap30l	NCC	FALSE
Slc7a7	NCC	FALSE
Frzb	NCC	FALSE
Eif2b2	NCC	FALSE
P4ha1	NCC	FALSE
Med25	NCC	FALSE
Atl3	NCC	FALSE
Dnajc17	NCC	FALSE
Nuak2	NCC	TRUE
Tpi1	NCC	TRUE
Ino80d	NCC	FALSE
Mettl22	NCC	FALSE
AI837181	NCC	FALSE
Atg12	NCC	FALSE
Zfp599	NCC	FALSE
Gm5141	NCC	FALSE
Qprt	NCC	FALSE
Slc39a14	NCC	FALSE
Wdr25	NCC	FALSE
Ubxn8	NCC	FALSE
Hist2h2bb	NCC	FALSE
Golga5	NCC	FALSE
Slc25a1	NCC	FALSE

Chpf	NCC	FALSE
Lrrc28	NCC	FALSE
Gdap10	NCC	FALSE
Lrrc61	NCC	FALSE
Nudt22	NCC	FALSE
Plag1	NCC	TRUE
Ciapin1	NCC	FALSE
Ube2s	NCC	FALSE
Socs1	NCC	FALSE
Srpr	NCC	FALSE
Tbrg1	NCC	FALSE
Gjb5	NCC	FALSE
Snx11	NCC	FALSE
Fam135a	NCC	FALSE
Inpp5f	NCC	FALSE
Tmem141	NCC	FALSE
Myo7a	NCC	FALSE
9130011E1	NCC	FALSE
Ttc7	NCC	FALSE
Poc5	NCC	FALSE
Sppl2a	NCC	FALSE
3110021N2	NCC	FALSE
Tssk6	NCC	FALSE
L3hypdh	NCC	FALSE
Shf	NCC	FALSE
Setd7	NCC	FALSE
Ube4a	NCC	FALSE
Pafah1b1	NCC	FALSE
Zfp943	NCC	FALSE
Otud5	NCC	FALSE
Map2k2	NCC	FALSE
Gprc5a	NCC	FALSE
Kifc5b	NCC	FALSE
Exoc8	NCC	FALSE
Gpr37	NCC	FALSE
Alg1	NCC	FALSE
Xrn1	NCC	FALSE
Itfg2	NCC	FALSE
Efcab2	NCC	FALSE
Amt	NCC	FALSE
Slc25a22	NCC	FALSE
Psmd13	NCC	FALSE
Fbxo31	NCC	FALSE
Pfkfb1	NCC	FALSE
Sec13	NCC	FALSE
Cog7	NCC	FALSE
Zfp870	NCC	FALSE
Wdr19	NCC	FALSE
Ncs1	NCC	FALSE
Mtap7d3	NCC	FALSE

Tmem17	NCC	FALSE
Fpgt	NCC	FALSE
Slc35b3	NCC	FALSE
Klhl17	NCC	FALSE
Zfp189	NCC	TRUE
Ascc1	NCC	TRUE
Zfp53	NCC	FALSE
Ankrd40	NCC	FALSE
Psen1	NCC	FALSE
1700048O2	NCC	FALSE
Itpkc	NCC	FALSE
Ube2g2	NCC	FALSE
Uvrag	NCC	FALSE
Tbc1d22a	NCC	FALSE
G6pc3	NCC	FALSE
4632428N(	NCC	FALSE
Zfp661	NCC	TRUE
Bex1	NCC	FALSE
Slc16a6	NCC	FALSE
Slc38a7	NCC	FALSE
Zfp874a	NCC	FALSE
Gphn	NCC	FALSE
Rasip1	NCC	FALSE
Pipox	NCC	FALSE
Aacs	NCC	FALSE
Mrps23	NCC	FALSE
Pcyt1b	NCC	FALSE
Klhl11	NCC	FALSE
Timmdc1	NCC	FALSE
Rnf113a2	NCC	FALSE
Ift122	NCC	FALSE
Sp8	NCC	TRUE
Nfil3	NCC	TRUE
Rabgef1	NCC	FALSE
Ccdc88c	NCC	FALSE
Ildr2	NCC	FALSE
Snrnp35	NCC	FALSE
Mad2l2	NCC	FALSE
Ppp3cb	NCC	FALSE
Gpr157	NCC	FALSE
Kptn	NCC	FALSE
Rnf113a1	NCC	FALSE
Klhl20	NCC	FALSE
Zfp697	NCC	TRUE
A730017C2	NCC	FALSE
Ccdc57	NCC	FALSE
Hltf	NCC	TRUE
Klhl15	NCC	FALSE
Zbed5	NCC	FALSE
Triobp	NCC	FALSE

Zfp935	NCC	TRUE
Ccng1	NCC	FALSE
Slc25a44	NCC	FALSE
Col4a6	NCC	FALSE
Srp9	NCC	TRUE
Fbxo45	NCC	FALSE
Parp11	NCC	FALSE
Tigd2	NCC	FALSE
Atp5s	NCC	FALSE
Gm5595	NCC	FALSE
Vps53	NCC	FALSE
Casp3	NCC	FALSE
Msx2	NCC	TRUE
Retsat	NCC	FALSE
Kctd9	NCC	FALSE
Enox1	NCC	FALSE
Xkr8	NCC	FALSE
Pard6b	NCC	FALSE
Rnf170	NCC	FALSE
Cpox	NCC	FALSE
Cdip1	NCC	FALSE
Gpld1	NCC	FALSE
A930004D1	NCC	FALSE
Rufy1	NCC	FALSE
Zfp873	NCC	FALSE
Trmt10b	NCC	FALSE
Gm14326	NCC	TRUE
Gosr1	NCC	FALSE
Sec22c	NCC	FALSE
Sil1	NCC	FALSE
Ttc14	NCC	FALSE
Smg6	NCC	FALSE
Hoxa6	NCC	TRUE
Nek3	NCC	FALSE
Itpk1	NCC	FALSE
Sergef	NCC	FALSE
4930430F0	NCC	FALSE
1600002K0	NCC	FALSE
Rgl3	NCC	FALSE
Heatr5b	NCC	FALSE
Triap1	NCC	FALSE
Slfn9	NCC	FALSE
Zfyve21	NCC	FALSE
Snora3	NCC	FALSE
1700007L1	NCC	FALSE
Tapt1	NCC	FALSE
Uvssa	NCC	FALSE
Apmap	NCC	FALSE
Armcx5	NCC	FALSE
2010204K1	NCC	FALSE

Mon1b	NCC	FALSE
Mbnl3	NCC	FALSE
Mtfr2	NCC	FALSE
Rpl19	NCC	FALSE
Tfb2m	NCC	FALSE
Zfp959	NCC	TRUE
D930007P1	NCC	FALSE
Hist1h3d	NCC	FALSE
Terf2ip	NCC	FALSE
Hook2	NCC	FALSE
Nt5c2	NCC	FALSE
Wdr91	NCC	FALSE
Prpsap2	NCC	FALSE
Dnajc8	NCC	FALSE
Parp16	NCC	FALSE
Mettl17	NCC	FALSE
Rbck1	NCC	FALSE
Znrd1as	NCC	FALSE
D5Ertd579	NCC	FALSE
Aftph	NCC	FALSE
l7Rn6	NCC	FALSE
Palm3	NCC	FALSE
Ripk1	NCC	FALSE
Arsi	NCC	FALSE
Nudt17	NCC	FALSE
Ptpn1	NCC	FALSE
Csgalnact2	NCC	FALSE
Mief2	NCC	FALSE
Ppme1	NCC	FALSE
Tmco6	NCC	FALSE
Als2	NCC	FALSE
Actr8	NCC	FALSE
Dhx32	NCC	FALSE
Dhdh	NCC	FALSE
AI450353	NCC	FALSE
Dennd4b	NCC	FALSE
Poli	NCC	TRUE
Znrf2	NCC	FALSE
Antxr2	NCC	FALSE
Ahdc1	NCC	FALSE
Lnx1	NCC	FALSE
Tgoln1	NCC	FALSE
Med13	NCC	FALSE
Zfp868	NCC	TRUE
Mus81	NCC	FALSE
Msl3l2	NCC	FALSE
Hmgn5	NCC	FALSE
Rpl23a	NCC	FALSE
Rps27	NCC	FALSE
D7Ertd715	NCC	FALSE

Kctd6	NCC	FALSE
Jagn1	NCC	FALSE
Gak	NCC	FALSE
E4f1	NCC	TRUE
Lss	NCC	FALSE
Irs2	NCC	FALSE
H60b	NCC	FALSE
Gm11944	NCC	FALSE
Aldh3a2	NCC	FALSE
Ptch2	NCC	FALSE
Fitm2	NCC	FALSE
Bbs10	NCC	FALSE
Bok	NCC	FALSE
Mar-06	NCC	FALSE
Fdxacb1	NCC	FALSE
Mark2	NCC	FALSE
Kdsr	NCC	FALSE
Prpf18	NCC	FALSE
Ptpla	NCC	FALSE
Klhl18	NCC	FALSE
Slc9a8	NCC	FALSE
4933404O1	NCC	FALSE
Mrps28	NCC	FALSE
Zfp87	NCC	FALSE
Cyb5r1	NCC	TRUE
Ptrh1	NCC	FALSE
Slc39a8	NCC	FALSE
Stbd1	NCC	FALSE
Zfp72	NCC	FALSE
Fam21	NCC	FALSE
Mfn2	NCC	FALSE
Apbb2	NCC	FALSE
Mzt2	NCC	FALSE
Snord32a	NCC	FALSE
Anxa7	NCC	FALSE
Ezr	NCC	TRUE
Dph3	NCC	FALSE
9430020K0	NCC	FALSE
Creld2	NCC	FALSE
Stau2	NCC	TRUE
Hras	NCC	FALSE
Mettl25	NCC	FALSE
Zfp758	NCC	FALSE
Fam172a	NCC	FALSE
Kank2	NCC	FALSE
Gm3219	NCC	FALSE
Ly6e	NCC	FALSE
Taf7	NCC	TRUE
Spsb3	NCC	FALSE
Tctn3	NCC	FALSE

1700037H(	NCC	FALSE
1810044D(	NCC	FALSE
Rpl27	NCC	FALSE
Dcun1d3	NCC	FALSE
Zscan20	NCC	TRUE
4930513N1	NCC	FALSE
Prss36	NCC	FALSE
Nudt10	NCC	FALSE
Zfp764	NCC	TRUE
Mrm1	NCC	FALSE
Gm10336	NCC	FALSE
Zfp109	NCC	TRUE
Dhtkd1	NCC	FALSE
Stat5b	NCC	TRUE
Ralgapb	NCC	FALSE
Wdr37	NCC	FALSE
2610002M	NCC	FALSE
Pgbd1	NCC	FALSE
Adamtsl4	NCC	FALSE
Gyltl1b	NCC	FALSE
Narfl	NCC	FALSE
Fbxo16	NCC	FALSE
D17Wsu92	NCC	FALSE
Aard	NCC	FALSE
Zbtb24	NCC	TRUE
D030028A(	NCC	FALSE
Msrb3	NCC	TRUE
Gpc4	NCC	FALSE
Renbp	NCC	FALSE
Spopl	NCC	FALSE
Mlf1	NCC	FALSE
D430042O(	NCC	FALSE
Mblac1	NCC	FALSE
Aptx	NCC	FALSE
Iqce	NCC	FALSE
Hps3	NCC	FALSE
Pigb	NCC	FALSE
Tecpr2	NCC	FALSE
Spag16	NCC	FALSE
U2af1	NCC	TRUE
Tmem109	NCC	FALSE
Tk2	NCC	FALSE
Tsc1	NCC	FALSE
Atg2b	NCC	FALSE
Zfp263	NCC	TRUE
Psmb10	NCC	FALSE
Pklr	NCC	FALSE
Elovl6	NCC	FALSE
Hoxc9	NCC	TRUE
Ngrn	NCC	FALSE

Steap2	NCC	FALSE
Mvd	NCC	FALSE
Stx17	NCC	FALSE
Arl2	NCC	FALSE
Abcb10	NCC	FALSE
Aoc2	NCC	FALSE
Arhgap18	NCC	FALSE
Cmah	NCC	FALSE
Pdxp	NCC	FALSE
Slc25a40	NCC	FALSE
Nckipsd	NCC	FALSE
Zfp2	NCC	TRUE
Ccdc32	NCC	FALSE
Gas2l3	NCC	FALSE
Snx14	NCC	FALSE
Jmy	NCC	FALSE
Armc8	NCC	FALSE
Mtmr11	NCC	FALSE
Arl16	NCC	FALSE
Oxnad1	NCC	FALSE
Slc38a6	NCC	FALSE
Fgfr3	NCC	FALSE
Tmx1	NCC	FALSE
Mctp2	NCC	TRUE
Helb	NCC	FALSE
Capn10	NCC	FALSE
Mzt1	NCC	FALSE
Rps6kc1	NCC	FALSE
Nfkbil1	NCC	FALSE
Zfp286	NCC	TRUE
Setbp1	NCC	TRUE
Rap1a	NCC	FALSE
Tbc1d19	NCC	FALSE
Oscp1	NCC	FALSE
Angptl4	NCC	FALSE
H2afy2	NCC	FALSE
Snord87	NCC	FALSE
Zfp809	NCC	FALSE
Klf15	NCC	TRUE
Disp1	NCC	FALSE
Ctdspl	NCC	FALSE
Erc1	NCC	FALSE
Ufsp1	NCC	FALSE
Csnk1g3	NCC	FALSE
Snord17	NCC	FALSE
Hddc3	NCC	FALSE
Thnsl2	NCC	FALSE
B4galnt4	NCC	FALSE
Cgn	NCC	FALSE
Tprn	NCC	FALSE

Zfp867	NCC	TRUE
Phf21b	NCC	FALSE
Col9a1	NCC	FALSE
Nup210l	NCC	FALSE
Gm7102	NCC	FALSE
Hip1r	NCC	FALSE
Nkap	NCC	FALSE
Foxe3	NCC	TRUE
Arhgap29	NCC	FALSE
Dnajb14	NCC	FALSE
Fam185a	NCC	FALSE
Tbck	NCC	FALSE
Rasal2	NCC	FALSE
2410089E0	NCC	FALSE
Snx13	NCC	FALSE
Snora61	NCC	FALSE
Chp1	NCC	FALSE
Ccdc166	NCC	FALSE
AW554918	NCC	FALSE
Dhrs13	NCC	FALSE
Mboat2	NCC	FALSE
Cyb5d1	NCC	FALSE
AW822252	NCC	FALSE
4930549G2	NCC	FALSE
Shroom4	NCC	FALSE
Syt7	NCC	FALSE
Zswim5	NCC	FALSE
Hnf1b	NCC	TRUE
Stox2	NCC	FALSE
Mark4	NCC	FALSE
Mapk8	NCC	FALSE
Fam134c	NCC	FALSE
Fam126b	NCC	FALSE
Fam110b	NCC	FALSE
Vasn	NCC	FALSE
Mir3091	NCC	FALSE
Zmym6	NCC	FALSE
Mir6904	NCC	FALSE
Smyd3	NCC	FALSE
Fau	NCC	FALSE
Gas2l1	NCC	FALSE
Zfp654	NCC	TRUE
Zfp708	NCC	TRUE
Tmem158	NCC	FALSE
Zfp493	NCC	FALSE
Slc3a2	NCC	FALSE
Scmh1	NCC	TRUE
Map3k6	NCC	FALSE
Slc2a4rg-ps	NCC	FALSE
Tada2b	NCC	FALSE

64305620:	NCC	FALSE
Slx4ip	NCC	FALSE
Rsbn1l	NCC	FALSE
Zfp61	NCC	TRUE
Pramef8	NCC	FALSE
Pih1d1	NCC	FALSE
Arhgap21	NCC	FALSE
Cables2	NCC	FALSE
Arel1	NCC	FALSE
1600014C1	NCC	FALSE
Bola1	NCC	FALSE
Bcl7b	NCC	FALSE
Tgfbrap1	NCC	FALSE
Nat9	NCC	FALSE
3010026O(	NCC	FALSE
Serhl	NCC	FALSE
Hist1h3i	NCC	FALSE
Lipt1	NCC	FALSE
Hnrnph3	NCC	TRUE
Msra	NCC	TRUE
Foxred2	NCC	FALSE
Sbno2	NCC	FALSE
Bcl10	NCC	FALSE
Atg14	NCC	FALSE
Csrnp1	NCC	FALSE
Dgkq	NCC	FALSE
Mtf1	NCC	TRUE
Mfsd3	NCC	FALSE
Lgalsl	NCC	FALSE
Habp4	NCC	FALSE
Gm9199	NCC	FALSE
Mafg	NCC	TRUE
Tsacc	NCC	FALSE
Pigg	NCC	FALSE
Bnip3	NCC	FALSE
Mgll	NCC	FALSE
Tex2	NCC	FALSE
Dmwd	NCC	FALSE
Spin2c	NCC	FALSE
E2f2	NCC	TRUE
Tob2	NCC	TRUE
Grik1	NCC	FALSE
Bex4	NCC	FALSE
Edn3	NCC	FALSE
Atp7b	NCC	FALSE
Slc25a16	NCC	FALSE
B3gat3	NCC	FALSE
Chd3os	NCC	FALSE
Wwox	NCC	FALSE
Cttnbp2nl	NCC	FALSE

Zfp11	NCC	TRUE
Bbs2	NCC	FALSE
Zfp839	NCC	FALSE
Gm14420	NCC	FALSE
E330009J0	NCC	FALSE
Setmar	NCC	FALSE
Eml1	NCC	FALSE
Sp7	NCC	TRUE
Il12rb2	NCC	FALSE
Snora43	NCC	FALSE
Tmem88	NCC	FALSE
Sos2	NCC	FALSE
Igsf10	NCC	FALSE
Mapk7	NCC	FALSE
Sertad1	NCC	FALSE
Cdkl5	NCC	FALSE
Atxn7l1	NCC	FALSE
Tpm2	NCC	FALSE
Fam160b1	NCC	FALSE
Dis3l2	NCC	FALSE
Socs7	NCC	FALSE
Coa6	NCC	FALSE
Snord34	NCC	FALSE
Gm13152	NCC	FALSE
Nxt2	NCC	FALSE
Dnajc12	NCC	FALSE
Gucy1a2	NCC	FALSE
Dnajb3	NCC	FALSE
Psmg4	NCC	FALSE
Cdx1	NCC	TRUE
B3gnt7	NCC	FALSE
Dgcr6	NCC	FALSE
Setd3	NCC	FALSE
Gm2027	NCC	FALSE
B130024G1	NCC	FALSE
Hkdc1	NCC	FALSE
BC065397	NCC	FALSE
Tor3a	NCC	FALSE
Fsd1	NCC	FALSE
Gm3414	NCC	FALSE
4930558J1	NCC	FALSE
Lypla2	NCC	FALSE
E130218I0	NCC	FALSE
D6Wsu163	NCC	FALSE
Hist1h2an	NCC	FALSE
Neurl4	NCC	FALSE
Thtpa	NCC	FALSE
Rnf146	NCC	FALSE
Gjb3	NCC	FALSE
Tert	NCC	FALSE

BC048507	NCC	FALSE
Nup62-il4i1	NCC	FALSE
Arl1	NCC	FALSE
Pcif1	NCC	FALSE
Ift57	NCC	FALSE
Ceacam1	NCC	FALSE
Rita1	NCC	FALSE
Thnsl1	NCC	FALSE
Slc25a28	NCC	FALSE
Zfp420	NCC	TRUE
Zfp775	NCC	TRUE
Camkmt	NCC	FALSE
Pemt	NCC	FALSE
Aph1a	NCC	FALSE
Papolb	NCC	FALSE
Hyls1	NCC	FALSE
Kif3b	NCC	FALSE
Lmln	NCC	FALSE
Ciart	NCC	FALSE
Gnal	NCC	FALSE
Tmeff1	NCC	FALSE
Zfp862-ps	NCC	FALSE
Efna2	NCC	FALSE
Cmtr1	NCC	FALSE
Snapc3	NCC	FALSE
Lrrc16a	NCC	FALSE
Gchfr	NCC	FALSE
Nabp2	NCC	FALSE
Dhrs11	NCC	FALSE
Snord99	NCC	FALSE
Hoxa11	NCC	TRUE
Tmem180	NCC	FALSE
Necap1	NCC	FALSE
Plekhb2	NCC	FALSE
Siah2	NCC	FALSE
Snora74a	NCC	FALSE
Slc7a2	NCC	FALSE
Carf	NCC	TRUE
Acat3	NCC	FALSE
Wdr31	NCC	FALSE
Nxph3	NCC	TRUE
Ccsap	NCC	FALSE
Tmem144	NCC	FALSE
Cc2d2a	NCC	FALSE
Ercc1	NCC	FALSE
Ciao1	NCC	FALSE
Rpl36al	NCC	FALSE
Myo7b	NCC	FALSE
Dguok	NCC	FALSE
Epo	NCC	FALSE

Cgnl1	NCC	FALSE
Ywhaz	NCC	TRUE
Zfp354a	NCC	TRUE
Isoc1	NCC	FALSE
Pcolce2	NCC	FALSE
Lrfn3	NCC	FALSE
Adssl1	NCC	FALSE
Abhd11	NCC	FALSE
Ntmt1	NCC	FALSE
Zbtb6	NCC	TRUE
Plekhh2	NCC	FALSE
Fxr2	NCC	FALSE
Ptpn4	NCC	FALSE
Dnase1	NCC	FALSE
Smarcd2	NCC	FALSE
Cr1l	NCC	FALSE
Srxn1	NCC	FALSE
A530054K1	NCC	FALSE
Polr2l	NCC	FALSE
Scml2	NCC	FALSE
1110012L1	NCC	FALSE
Ikbke	NCC	FALSE
Hsd17b7	NCC	FALSE
Atg4b	NCC	FALSE
Cdc42ep4	NCC	FALSE
1110034G2	NCC	FALSE
Snord33	NCC	FALSE
Zfp524	NCC	TRUE
Inpp5a	NCC	FALSE
Cdkl3	NCC	FALSE
Zfp960	NCC	TRUE
Lrrc4	NCC	FALSE
Mir7038	NCC	FALSE
Snord55	NCC	FALSE
Tmem132c	NCC	FALSE
Gm11110	NCC	FALSE
Efcab6	NCC	FALSE
Lrrc49	NCC	FALSE
Arsk	NCC	FALSE
Zfp229	NCC	FALSE
Adck4	NCC	FALSE
BC022687	NCC	FALSE
Eid2	NCC	FALSE
Wdr78	NCC	FALSE
Ramp2	NCC	FALSE
9830147E1	NCC	FALSE
Rhbdd1	NCC	FALSE
Gm5523	NCC	FALSE
Auh	NCC	FALSE
Anks3	NCC	FALSE

Prkg2	NCC	FALSE
Gal3st3	NCC	FALSE
Eya2	NCC	FALSE
Chrdl1	NCC	FALSE
Hykk	NCC	FALSE
Dusp28	NCC	FALSE
Ankrd45	NCC	FALSE
Stard4	NCC	FALSE
Ptbp2	NCC	FALSE
Hebp2	NCC	FALSE
Cxadr	NCC	FALSE
Nfatc2ip	NCC	FALSE
Ralgapa2	NCC	FALSE
Gm4961	NCC	FALSE
Zscan18	NCC	TRUE
Tceanc	NCC	FALSE
Usp2	NCC	FALSE
Zfp473	NCC	FALSE
Uxt	NCC	FALSE
Zfp51	NCC	FALSE
Mboat1	NCC	FALSE
Ripk2	NCC	FALSE
Atf7	NCC	TRUE
Tmem107	NCC	FALSE
Tmem67	NCC	FALSE
Mia3	NCC	FALSE
Vps52	NCC	FALSE
Kdm7a	NCC	TRUE
Irx5	NCC	TRUE
Zswim3	NCC	FALSE
Ctps2	NCC	FALSE
Dip2c	NCC	FALSE
Pigw	NCC	FALSE
Ccdc24	NCC	FALSE
2410018L1	NCC	FALSE
Idi1	NCC	FALSE
Man2b2	NCC	FALSE
Tbcc	NCC	FALSE
Il6st	NCC	FALSE
Sord	NCC	FALSE
Ribc1	NCC	FALSE
Gpatch11	NCC	FALSE
Clcn7	NCC	FALSE
E030030I0(	NCC	FALSE
Cdk20	NCC	FALSE
Stx3	NCC	FALSE
Ttc19	NCC	FALSE
Snx7	NCC	FALSE
Snora41	NCC	FALSE
Hapln1	NCC	FALSE

B4galt5	NCC	FALSE
Rsg1	NCC	FALSE
Adora1	NCC	FALSE
Zfp846	NCC	TRUE
Pycard	NCC	FALSE
Pdp2	NCC	FALSE
Srrm2	NCC	FALSE
Gm8363	NCC	FALSE
Dynll2	NCC	FALSE
Tmem181a	NCC	FALSE
Calm2	NCC	FALSE
Fzd9	NCC	FALSE
Spata7	NCC	FALSE
Fbxo10	NCC	FALSE
Tbc1d13	NCC	FALSE
Rnf141	NCC	FALSE
Hemk1	NCC	FALSE
Sh3yl1	NCC	FALSE
Pip4k2b	NCC	FALSE
Mgarp	NCC	FALSE
Iba57	NCC	FALSE
Tfpt	NCC	FALSE
1700010I1	NCC	FALSE
Engase	NCC	FALSE
Clasp1	NCC	FALSE
Tekt1	NCC	FALSE
Nadsyn1	NCC	FALSE
Phlpp1	NCC	FALSE
Vti1b	NCC	FALSE
Dhrs7	NCC	FALSE
1110051M	NCC	FALSE
AF357399	NCC	FALSE
Dpf2	NCC	FALSE
Tst	NCC	FALSE
Lmbrd2	NCC	FALSE
Dnajc14	NCC	FALSE
Fam220a	NCC	FALSE
Rmnd5b	NCC	FALSE
Fchsd2	NCC	FALSE
Phc3	NCC	FALSE
Eldr	NCC	FALSE
Aspg	NCC	FALSE
Bloc1s3	NCC	FALSE
Slc25a47	NCC	FALSE
Lyrm9	NCC	FALSE
Sik3	NCC	FALSE
Zfp59	NCC	TRUE
Flcn	NCC	FALSE
Pcsk7	NCC	FALSE
Ccdc124	NCC	FALSE

1700094D0	NCC	FALSE
Zfp772	NCC	FALSE
Pla2g15	NCC	FALSE
Gstm4	NCC	FALSE
Rabep1	NCC	FALSE
Pdp1	NCC	FALSE
Rnf31	NCC	FALSE
Synj2bp	NCC	FALSE
Kctd18	NCC	FALSE
Sccpdh	NCC	FALSE
Smco4	NCC	FALSE
Sh3bp5l	NCC	FALSE
Sik1	NCC	FALSE
Gm20199	NCC	FALSE
Bbc3	NCC	FALSE
Tcaim	NCC	FALSE
Nr2c2	NCC	TRUE
Fuca2	NCC	FALSE
Slc25a39	NCC	FALSE
Txndc11	NCC	FALSE
Hspa1a	NCC	FALSE
Degs2	NCC	FALSE
Cdc37l1	NCC	FALSE
Zfp46	NCC	TRUE
Gm6981	NCC	FALSE
Pdpk1	NCC	FALSE
Katnal2	NCC	FALSE
Hs6st1	NCC	FALSE
Fbxo17	NCC	FALSE
Slc22a5	NCC	FALSE
Airn	NCC	FALSE
Atp6v1a	NCC	FALSE
Rps6ka2	NCC	FALSE
Slu7	NCC	FALSE
Clock	NCC	TRUE
Gm1976	NCC	FALSE
Slc25a19	NCC	FALSE
Gm3716	NCC	FALSE
Zdbf2	NCC	FALSE
Rab20	NCC	FALSE
Dennd4a	NCC	FALSE
6330408A0	NCC	FALSE
Slc25a51	NCC	FALSE
Nsun3	NCC	FALSE
Acbd6	NCC	FALSE
Slc39a11	NCC	FALSE
St7	NCC	FALSE
Cdc42bpa	NCC	FALSE
Aff1	NCC	FALSE
Isca1	NCC	FALSE

Nosip	NCC	FALSE
Pip5k1c	NCC	FALSE
Mkl1	NCC	FALSE
Pou3f2	NCC	TRUE
Tcfl5	NCC	TRUE
Fxyd6	NCC	FALSE
Atxn7l2	NCC	FALSE
Irf2bp2	NCC	FALSE
AA987161	NCC	FALSE
Pex1	NCC	FALSE
Fars2	NCC	FALSE
Ankrd42	NCC	FALSE
Cgrrf1	NCC	FALSE
Coch	NCC	FALSE
Cacna1a	NCC	FALSE
Gcc1	NCC	FALSE
Btbd10	NCC	FALSE
Hist1h3c	NCC	FALSE
Rundc1	NCC	FALSE
Arf1	NCC	FALSE
Cited4	NCC	FALSE
Tmem59l	NCC	FALSE
Setd4	NCC	FALSE
4930478L0	NCC	FALSE
4933417G(	NCC	FALSE
Ago3	NCC	FALSE
Pex11a	NCC	FALSE
Grk5	NCC	FALSE
Elof1	NCC	FALSE
Zbtb12	NCC	TRUE
Coq7	NCC	FALSE
Car3	NCC	FALSE
Nfrkb	NCC	FALSE
Ppm1a	NCC	FALSE
9130019O:	NCC	TRUE
Kif4-ps	NCC	FALSE
Zfp790	NCC	TRUE
Snapc5	NCC	TRUE
Zcchc7	NCC	FALSE
Crlf1	NCC	FALSE
Slc35d2	NCC	FALSE
Armc7	NCC	FALSE
Zkscan6	NCC	TRUE
Chn1	NCC	FALSE
Fkbp15	NCC	FALSE
Napg	NCC	FALSE
Unc5d	NCC	FALSE
Nipal4	NCC	FALSE
Ift46	NCC	FALSE
Mylk	NCC	TRUE

Tcf15	NCC	TRUE
Tmem127	NCC	FALSE
Dact1	NCC	FALSE
Chmp7	NCC	FALSE
Blcap	NCC	FALSE
Pm20d1	NCC	FALSE
Kif16b	NCC	FALSE
Cradd	NCC	FALSE
Hexim2	NCC	FALSE
Cetn2	NCC	FALSE
Rps13	NCC	FALSE
Cisd1	NCC	FALSE
A230073K1	NCC	FALSE
Guca1a	NCC	FALSE
Fam181a	NCC	FALSE
Zfp607	NCC	FALSE
Tmem41b	NCC	FALSE
Gstm2	NCC	FALSE
Zfp120	NCC	TRUE
Satb1	NCC	TRUE
Rwdd3	NCC	FALSE
Lrif1	NCC	FALSE
Pih1d2	NCC	FALSE
Coq10b	NCC	FALSE
Mamdc4	NCC	FALSE
Coro7	NCC	FALSE
Fhl1	NCC	FALSE
Emc2	NCC	FALSE
Zdhhc21	NCC	FALSE
AW146154	NCC	TRUE
D330050I1	NCC	FALSE
Slc16a4	NCC	FALSE
Mterfd3	NCC	FALSE
Mgat4b	NCC	FALSE
Tnfrsf10b	NCC	FALSE
Prrg1	NCC	FALSE
Nudt15	NCC	FALSE
Rbfa	NCC	FALSE
Rcan1	NCC	FALSE
Atf6	NCC	TRUE
Zfp711	NCC	TRUE
E330033B0	NCC	FALSE
Tmem167b	NCC	FALSE
C1ql2	NCC	FALSE
Gm10389	NCC	FALSE
Dph6	NCC	FALSE
Oxld1	NCC	FALSE
Tmem170	NCC	FALSE
Pnp2	NCC	FALSE
Rasl12	NCC	FALSE

Vta1	NCC	FALSE
Ifitm1	NCC	FALSE
C030034I2:	NCC	FALSE
Ift22	NCC	FALSE
Lpl	NCC	FALSE
Zfp82	NCC	TRUE
Slc2a3	NCC	FALSE
Galnt18	NCC	FALSE
St3gal3	NCC	FALSE
Arhgap8	NCC	FALSE
Naif1	NCC	FALSE
Sult2b1	NCC	FALSE
Zfp715	NCC	FALSE
Lrrc75a	NCC	FALSE
Dfna5	NCC	FALSE
Gm5069	NCC	FALSE
Ccdc96	NCC	FALSE
Tmem222	NCC	FALSE
Elmod3	NCC	FALSE
Pdlim2	NCC	FALSE
Slc36a2	NCC	FALSE
Braf	NCC	FALSE
Snord57	NCC	FALSE
Gm15708	NCC	FALSE
Klhdc3	NCC	FALSE
Wdr41	NCC	FALSE
Tha1	NCC	FALSE
Arid4a	NCC	FALSE
Dnah17	NCC	FALSE
Anxa3	NCC	FALSE
Eva1b	NCC	FALSE
Tgfbr3	NCC	FALSE
Necab2	NCC	FALSE
Snord95	NCC	FALSE
Mfsd2a	NCC	FALSE
Fbxw4	NCC	FALSE
Snhg10	NCC	FALSE
Rnf214	NCC	FALSE
Pten	NCC	FALSE
Pax6	NCC	TRUE
4930452B0	NCC	FALSE
A930005H1	NCC	FALSE
Slc26a6	NCC	FALSE
4921531C2	NCC	FALSE
Fam19a4	NCC	FALSE
Grifin	NCC	FALSE
Pcnxl4	NCC	FALSE
Atxn7	NCC	FALSE
Timp1	NCC	FALSE
Pknox1	NCC	TRUE

Sowaha	NCC	FALSE
Raet1a	NCC	FALSE
Snord2	NCC	FALSE
Ovgp1	NCC	FALSE
Sc5d	NCC	FALSE
Rerg	NCC	FALSE
Tenc1	NCC	FALSE
Eme2	NCC	FALSE
Atp6v1c1	NCC	FALSE
Gm4890	NCC	FALSE
Fam45a	NCC	FALSE
Kcng1	NCC	FALSE
Mrpl43	NCC	FALSE
Akap7	NCC	FALSE
Esrp1	NCC	TRUE
F11r	NCC	FALSE
Gin1	NCC	FALSE
Ndrg3	NCC	FALSE
Gm14305	NCC	TRUE
Bola2	NCC	FALSE
Zfp619	NCC	FALSE
Usp30	NCC	FALSE
BC017643	NCC	FALSE
Gm16523	NCC	FALSE
Ift81	NCC	FALSE
Mir3076	NCC	FALSE
Lcorl	NCC	TRUE
Immp2l	NCC	FALSE
Akap17b	NCC	FALSE
Wnt5a	NCC	FALSE
Leng1	NCC	FALSE
Asl	NCC	FALSE
Cep83os	NCC	FALSE
Gnptab	NCC	FALSE
Fam129c	NCC	FALSE
Ranbp17	NCC	FALSE
Bmper	NCC	FALSE
Akr1c12	NCC	FALSE
Gm20939	NCC	TRUE
A230056P1	NCC	FALSE
A830082N(	NCC	FALSE
Rps4l	NCC	FALSE
Mtmr3	NCC	FALSE
Sdc2	NCC	FALSE
E030024N2	NCC	FALSE
Sprtn	NCC	FALSE
C130046K2	NCC	FALSE
Vapa	NCC	FALSE
Col4a3bp	NCC	FALSE
1500015A(	NCC	FALSE

Iqgap2	NCC	FALSE
Slx1b	NCC	FALSE
Lekr1	NCC	FALSE
Msx1	NCC	TRUE
Slc25a25	NCC	FALSE
Orai3	NCC	FALSE
Ttl	NCC	FALSE
2410004I0	NCC	FALSE
Snord19	NCC	FALSE
Nt5m	NCC	FALSE
Nfkbie	NCC	FALSE
Ocrl	NCC	FALSE
Parvb	NCC	FALSE
Gm9958	NCC	FALSE
Usp13	NCC	FALSE
Rtfdc1	NCC	FALSE
Wdr59	NCC	FALSE
Dach2	NCC	FALSE
Hint3	NCC	FALSE
1300002E1	NCC	FALSE
Gpr19	NCC	FALSE
Lsm7	NCC	FALSE
Mfhas1	NCC	FALSE
Ccdc186	NCC	FALSE
Hoxd12	NCC	TRUE
Cenpv	NCC	FALSE
Dync1li1	NCC	FALSE
2510003E0	NCC	FALSE
Epn2	NCC	FALSE
Rdm1	NCC	FALSE
Rragb	NCC	FALSE
Rbks	NCC	FALSE
Sdr39u1	NCC	FALSE
Bicd2	NCC	FALSE
Samd8	NCC	FALSE
Lonrf1	NCC	FALSE
Yif1b	NCC	FALSE
Appl2	NCC	FALSE
Tbc1d10b	NCC	FALSE
Pced1b	NCC	FALSE
Gm5124	NCC	FALSE
Grasp	NCC	FALSE
Ccdc120	NCC	FALSE
D1Pas1	NCC	FALSE
Zbtb3	NCC	TRUE
Tle1	NCC	FALSE
Ikzf4	NCC	TRUE
5730507C0	NCC	TRUE
Stap2	NCC	FALSE
Slc6a9	NCC	FALSE

Trex1	NCC	FALSE
Zfp605	NCC	FALSE
Klhl29	NCC	FALSE
Trabd2b	NCC	FALSE
Agbl5	NCC	FALSE
Glrb	NCC	FALSE
6820431F2	NCC	FALSE
Abcd4	NCC	FALSE
Add3	NCC	FALSE
Fbxw2	NCC	FALSE
Zbtb37	NCC	TRUE
Bdh1	NCC	FALSE
Cmbl	NCC	FALSE
Masp2	NCC	FALSE
Hoxc12	NCC	TRUE
Mycl	NCC	TRUE
9230105E0	NCC	FALSE
Nptx2	NCC	FALSE
Espn	NCC	FALSE
AU041133	NCC	TRUE
Zfp119a	NCC	TRUE
Rpia	NCC	FALSE
2310035C2	NCC	FALSE
Rnd1	NCC	FALSE
Zfp953	NCC	FALSE
BC033916	NCC	FALSE
Snora52	NCC	FALSE
Amigo2	NCC	FALSE
Fam120b	NCC	FALSE
Fam193b	NCC	FALSE
Zfp931	NCC	TRUE
Arntl2	NCC	TRUE
Serac1	NCC	FALSE
9330151L1	NCC	FALSE
Dnajc16	NCC	FALSE
Zfp526	NCC	TRUE
Itga8	NCC	FALSE
Mss51	NCC	FALSE
Rgs10	NCC	FALSE
Rnf181	NCC	FALSE
Grb2	NCC	FALSE
Ncdn	NCC	FALSE
Galns	NCC	FALSE
Hotair	NCC	FALSE
Zfp324	NCC	TRUE
Hist1h2bm	NCC	FALSE
Hoxa11os	NCC	FALSE
Trim45	NCC	FALSE
Zfa-ps	NCC	TRUE
Doc2g	NCC	FALSE

Myb	NCC	TRUE
Cntfr	NCC	FALSE
Fam63a	NCC	FALSE
Dync2li1	NCC	FALSE
Pop7	NCC	FALSE
Aurkaip1	NCC	FALSE
D130020L0	NCC	FALSE
Fam120c	NCC	FALSE
D2hgdh	NCC	FALSE
Rere	NCC	FALSE
Nat2	NCC	FALSE
Gtf2a2	NCC	TRUE
B130034C1	NCC	FALSE
Tmem143	NCC	FALSE
Dusp14	NCC	FALSE
Nr2c2ap	NCC	FALSE
Magee2	NCC	FALSE
Iqcg	NCC	FALSE
Gm16039	NCC	FALSE
C130036L2	NCC	FALSE
Slc35a5	NCC	FALSE
Raver2	NCC	FALSE
Ttc17	NCC	FALSE
Prg4	NCC	FALSE
Uap1l1	NCC	FALSE
Fbxo27	NCC	FALSE
Tpk1	NCC	FALSE
Lmbr1	NCC	FALSE
Cdyl2	NCC	FALSE
Mir7003	NCC	FALSE
Arhgef18	NCC	FALSE
Ppp1r26	NCC	FALSE
Fam69c	NCC	FALSE
Gm5177	NCC	FALSE
Alpk1	NCC	FALSE
Spag1	NCC	FALSE
Ccdc85b	NCC	FALSE
Rdh11	NCC	FALSE
Cox5a	NCC	FALSE
Tmem87b	NCC	FALSE
Tbc1d24	NCC	FALSE
Adcy3	NCC	FALSE
Gm3604	NCC	TRUE
Cldn14	NCC	FALSE
Gm15706	NCC	FALSE
Ptdss1	NCC	FALSE
Zfp658	NCC	FALSE
Hist4h4	NCC	FALSE
Pcsk1	NCC	FALSE
Rdh12	NCC	FALSE

Trip11	NCC	FALSE
Fancd2os	NCC	FALSE
Plekhm1	NCC	FALSE
Ubxn2a	NCC	FALSE
Zxda	NCC	FALSE
Omd	NCC	FALSE
Pfdn2	NCC	FALSE
Zfp94	NCC	TRUE
Gm14295	NCC	TRUE
Mpp2	NCC	FALSE
Zic5	NCC	TRUE
Gm20324	NCC	FALSE
Mrps18c	NCC	FALSE
Flad1	NCC	FALSE
Mir19b-1	NCC	FALSE
2200002D(	NCC	FALSE
Zbtb42	NCC	TRUE
Ctf1	NCC	FALSE
Snord45b	NCC	FALSE
Gm10364	NCC	FALSE
Smim19	NCC	FALSE
Pxylp1	NCC	FALSE
Tbc1d17	NCC	FALSE
Slc25a53	NCC	FALSE
Mcts1	NCC	FALSE
Hist1h3g	NCC	FALSE
Nudt12	NCC	FALSE
Nrg4	NCC	FALSE
Cd83	NCC	FALSE
Fam160b2	NCC	FALSE
Eepd1	NCC	FALSE
Ring1	NCC	FALSE
Mfap1b	NCC	FALSE
B130006D(	NCC	FALSE
Tmem184c	NCC	FALSE
Lysmd4	NCC	FALSE
Elk4	NCC	TRUE
Hdhd3	NCC	FALSE
Rab10os	NCC	FALSE
Zcchc14	NCC	TRUE
Bicc1	NCC	FALSE
Zkscan4	NCC	TRUE
Fam132b	NCC	FALSE
Mxd3	NCC	TRUE
Fn3krp	NCC	FALSE
St3gal5	NCC	FALSE
Fads1	NCC	FALSE
Spag17	NCC	FALSE
Mir363	NCC	FALSE
3200001D2	NCC	FALSE

4930507D(	NCC	FALSE
Ncf2	NCC	FALSE
Zcchc2	NCC	FALSE
Dpp4	NCC	FALSE
Cdadc1	NCC	FALSE
Atg4d	NCC	FALSE
Atg2a	NCC	FALSE
Osbpl3	NCC	FALSE
Zfp688	NCC	TRUE
9530027J0	NCC	FALSE
Nxpe4	NCC	FALSE
Mterf1a	NCC	TRUE
Rnf19b	NCC	FALSE
Cers6	NCC	TRUE
Ecscr	NCC	FALSE
Kis2	NCC	FALSE
Enox2	NCC	FALSE
Tbce	NCC	FALSE
Dock9	NCC	FALSE
Gm12992	NCC	FALSE
Hgs	NCC	FALSE
AV051173	NCC	FALSE
Akap11	NCC	FALSE
Hist1h3f	NCC	FALSE
Prr22	NCC	FALSE
Gm14403	NCC	TRUE
Snx30	NCC	FALSE
Zfp369	NCC	TRUE
Snord49b	NCC	FALSE
Snap29	NCC	FALSE
Ccdc125	NCC	FALSE
Lmbrd1	NCC	FALSE
Kat5	NCC	FALSE
Fbxo36	NCC	FALSE
Vps39	NCC	FALSE
B230208H1	NCC	FALSE
Fgf15	NCC	TRUE
Asphd2	NCC	FALSE
Casq2	NCC	FALSE
Itgae	NCC	FALSE
Ubn2	NCC	FALSE
Alyref2	NCC	FALSE
Rab43	NCC	FALSE
Ube2e2	NCC	FALSE
Napepld	NCC	FALSE
Nog	NCC	FALSE
Gm16982	NCC	FALSE
Gt(ROSA)2(	NCC	FALSE
Ccdc114	NCC	FALSE
3110043O2	NCC	FALSE

Camk2g	NCC	FALSE
Unc13d	NCC	FALSE
BC006965	NCC	FALSE
Arhgef1	NCC	FALSE
Map2k3os	NCC	FALSE
Smoc1	NCC	FALSE
Mok	NCC	FALSE
Ccdc130	NCC	FALSE
Gpr137	NCC	FALSE
Zc3h12b	NCC	FALSE
Zfand4	NCC	FALSE
Vps37c	NCC	FALSE
Sparcl1	NCC	FALSE
Zxdc	NCC	TRUE
Per3	NCC	FALSE
Fsbp	NCC	FALSE
Qdpr	NCC	FALSE
Galnt11	NCC	FALSE
Zfp119b	NCC	TRUE
Pin1rt1	NCC	FALSE
Rab8a	NCC	FALSE
Il18bp	NCC	FALSE
Hist1h2bb	NCC	FALSE
Neu3	NCC	FALSE
Snora17	NCC	FALSE
Zfp438	NCC	TRUE
Degs1	NCC	FALSE
Gm13212	NCC	TRUE
5730480H(	NCC	FALSE
Prrg3	NCC	FALSE
Rps6ka4	NCC	FALSE
Anxa9	NCC	FALSE
Ogfrl1	NCC	FALSE
Zic2	NCC	TRUE
Slc36a4	NCC	FALSE
Zfyve9	NCC	FALSE
Hoxc11	NCC	TRUE
Tmub1	NCC	FALSE
Snord83b	NCC	FALSE
Myd88	NCC	FALSE
Nkx3-1	NCC	TRUE
Nipsnap1	NCC	FALSE
Gnat2	NCC	FALSE
Slc25a48	NCC	FALSE
6330418K(	NCC	FALSE
Lrrc14b	NCC	FALSE
Dcaf5	NCC	FALSE
D330045A:	NCC	FALSE
Tmc7	NCC	FALSE
Slc39a6	NCC	FALSE

Zxdb	NCC	TRUE
C330021F2	NCC	FALSE
Hist1h2bk	NCC	FALSE
Timm17a	NCC	FALSE
Gna14	NCC	FALSE
Eea1	NCC	FALSE
Wdtc1	NCC	FALSE
Hist1h3e	NCC	FALSE
Gabra4	NCC	FALSE
Nkapl	NCC	FALSE
Map6d1	NCC	FALSE
Efcc1	NCC	FALSE
Malt1	NCC	FALSE
B3gnt1	NCC	FALSE
Sbds	NCC	FALSE
Slc25a29	NCC	FALSE
Esco1	NCC	FALSE
AU040320	NCC	FALSE
Ganc	NCC	FALSE
Slc9b2	NCC	FALSE
Mvk	NCC	FALSE
Pdcl3	NCC	FALSE
Foxd2	NCC	TRUE
9430091E2	NCC	FALSE
Adam15	NCC	FALSE
Tdg	NCC	FALSE
2610524H(	NCC	FALSE
Ankrd37	NCC	FALSE
Lypd6	NCC	FALSE
Khdrbs3	NCC	FALSE
Rpl28	NCC	FALSE
Naaladl1	NCC	FALSE
Lyrm1	NCC	FALSE
Lingo3	NCC	FALSE
Gm7120	NCC	FALSE
1110054M	NCC	FALSE
Gpr63	NCC	FALSE
Kcnk1	NCC	FALSE
Arl4c	NCC	FALSE
Plch1	NCC	FALSE
Tspyl5	NCC	FALSE
E430018J2	NCC	TRUE
Gm10653	NCC	FALSE
Zfp97	NCC	TRUE
Hfe2	NCC	FALSE
Nell2	Hub	FALSE
Cdkn1c	Hub	FALSE
Dlx1	Hub	TRUE
Rbp1	Hub	FALSE
Slitrk6	Hub	FALSE

Cthrc1	Hub	FALSE
Ldhb	Hub	FALSE
Tgfb2	Hub	FALSE
S100a16	Hub	FALSE
Serpine2	Hub	FALSE
Celf2	Hub	FALSE
Wwtr1	Hub	FALSE
Dpysl3	Hub	FALSE
Cdh10	Hub	FALSE
Ckb	Hub	FALSE
Col5a2	Hub	FALSE
Cacna1h	Hub	FALSE
Kif21a	Hub	FALSE
Tmem98	Hub	FALSE
Irf6	Hub	TRUE
Pgpep1	Hub	FALSE
Anxa6	Hub	FALSE
Fabp7	Hub	FALSE
Ascl1	Hub	TRUE
Mmp14	Hub	FALSE
Fbln1	Hub	FALSE
Sorcs1	Hub	FALSE
Kctd12	Hub	FALSE
Samd5	Hub	FALSE
Cyp2j6	Hub	FALSE
E130114P1	Hub	FALSE
Lgals1	Hub	FALSE
Lipa	Hub	FALSE
Anxa5	Hub	FALSE
Adam23	Hub	FALSE
Adamts7	Hub	FALSE
Rhoc	Hub	FALSE
Ptprm	Hub	FALSE
Tbx2	Hub	TRUE
Chst5	Hub	FALSE
Gpr126	Hub	FALSE
Lama4	Hub	FALSE
Dclk1	Hub	FALSE
Pxdc1	Hub	FALSE
Pdcd4	Hub	FALSE
Sh3pxd2a	Hub	FALSE
Sash1	Hub	FALSE
Rps18	Hub	FALSE
S100a11	Hub	FALSE
Cdh19	Hub	FALSE
Maged2	Hub	FALSE
Mfap2	Hub	FALSE
Rps26	Hub	FALSE
Ankrd50	Hub	FALSE
Aldh2	Hub	FALSE

Daam2	Hub	FALSE
Reck	Hub	FALSE
Mpzl1	Hub	FALSE
Mab21l1	Hub	FALSE
Mov10	Hub	FALSE
Atp10b	Hub	FALSE
Zfp536	Hub	TRUE
Srpk2	Hub	FALSE
Sox2ot	Hub	FALSE
Ccnd1	Hub	FALSE
Klhl30	Hub	FALSE
Aes	Hub	FALSE
Gas7	Hub	FALSE
Itgav	Hub	FALSE
Ptprz1	Hub	FALSE
Ssbp4	Hub	FALSE
Prss12	Hub	FALSE
Phox2b	Hub	TRUE
Postn	Hub	FALSE
Aldh1a3	Hub	FALSE
Ywhab	Hub	FALSE
Ctsl	Hub	FALSE
Sparc	Hub	FALSE
Slc39a1	Hub	FALSE
Spry2	Hub	FALSE
Tcirg1	Hub	FALSE
Mcam	Hub	FALSE
Grik2	Hub	FALSE
Dlx1as	Hub	FALSE
Atat1	Hub	FALSE
Rpl39	Hub	FALSE
Foxo1	Hub	TRUE
Csrp1	Hub	FALSE
Rpl13a	Hub	FALSE
Zdhhc2	Hub	FALSE
Gng11	Hub	FALSE
Sort1	Hub	FALSE
Scrg1	Hub	FALSE
Rpl32	Hub	FALSE
Rps19	Hub	FALSE
Tmtc2	Hub	FALSE
Oxct1	Hub	FALSE
Fam184b	Hub	FALSE
1700025G(	Hub	FALSE
Tcf4	Hub	TRUE
Ypel2	Hub	FALSE
Mvb12b	Hub	FALSE
Agrn	Hub	FALSE
Gpr124	Hub	FALSE
Fam173a	Hub	FALSE

Ccdc8	Hub	FALSE
Fuca1	Hub	FALSE
Epha7	Hub	FALSE
Shfm1	Hub	FALSE
Gnai2	Hub	FALSE
Ddr1	Hub	FALSE
Rnaseh2c	Hub	TRUE
Epb4.1l3	Hub	FALSE
Rps14	Hub	FALSE
Shc4	Hub	FALSE
Ppdpf	Hub	FALSE
Nid1	Hub	FALSE
Fam198b	Hub	FALSE
Celsr2	Hub	FALSE
Marcks	Hub	FALSE
Akr1a1	Hub	TRUE
Col9a3	Hub	FALSE
Rps24	Hub	FALSE
Endod1	Hub	FALSE
Megf9	Hub	FALSE
Esyt1	Hub	FALSE
Ajap1	Hub	FALSE
Eid1	Hub	FALSE
Cadps	Hub	FALSE
Pde9a	Hub	FALSE
Fibin	Hub	FALSE
Arhgap23	Hub	FALSE
Pard3b	Hub	FALSE
Msn	Hub	FALSE
Mmp15	Hub	FALSE
Ndst3	Hub	FALSE
Fstl1	Hub	FALSE
Ppib	Hub	FALSE
Col9a2	Hub	FALSE
Itm2c	Hub	FALSE
Eif3f	Hub	FALSE
Hdac9	Hub	FALSE
Ctnnal1	Hub	FALSE
Rpl37a	Hub	FALSE
Myo18a	Hub	FALSE
Acss1	Hub	FALSE
Rps7	Hub	FALSE
Tpt1	Hub	FALSE
Tspan7	Hub	FALSE
Ppp1r9a	Hub	FALSE
Dzip1	Hub	FALSE
Fbxo7	Hub	FALSE
Rps15a-ps4	Hub	FALSE
Slit3	Hub	FALSE
Akap12	Hub	FALSE

Rpl18a	Hub	FALSE
Myh14	Hub	FALSE
Oaz2	Hub	FALSE
Rftn2	Hub	FALSE
Ndfip1	Hub	FALSE
Ptprj	Hub	FALSE
Rbms3	Hub	FALSE
Tia1	Hub	TRUE
Crem	Hub	TRUE
Luzp2	Hub	TRUE
Etfa	Hub	FALSE
Wipi1	Hub	FALSE
Sh3glb1	Hub	FALSE
Cox7a2l	Hub	FALSE
Pfdn1	Hub	FALSE
Plekhg1	Hub	FALSE
Trp53inp1	Hub	FALSE
Cst3	Hub	FALSE
Ube2h	Hub	FALSE
Gpx7	Hub	FALSE
Olfm2	Hub	FALSE
Trappc1	Hub	FALSE
Fez1	Hub	TRUE
Hmgn3	Hub	TRUE
Plekha5	Hub	FALSE
Padi2	Hub	FALSE
Sox4	Hub	TRUE
Tspan3	Hub	FALSE
Wbp5	Hub	FALSE
Prdx2	Hub	FALSE
St6gal1	Hub	FALSE
H2afv	Hub	FALSE
Pbxip1	Hub	FALSE
Efna5	Hub	FALSE
C1ql1	Hub	FALSE
Id4	Hub	TRUE
Tax1bp3	Hub	FALSE
Kdm4a	Hub	TRUE
Ctsb	Hub	FALSE
Stk10	Hub	FALSE
Pde7b	Hub	FALSE
Ilk	Hub	FALSE
Hmg20b	Hub	TRUE
Vamp8	Hub	FALSE
Axl	Hub	FALSE
Deptor	Hub	FALSE
Ddit4	Hub	FALSE
Col20a1	Hub	FALSE
Il1rap	Hub	FALSE
Sgce	Hub	FALSE

Btg1	Hub	FALSE
Hmgn2	Hub	FALSE
Dennd2a	Hub	FALSE
Itgb1	Hub	FALSE
Gpm6b	Hub	FALSE
Atrx	Hub	FALSE
Cald1	Hub	FALSE
Trf	Hub	FALSE
Capg	Hub	FALSE
Grik3	Hub	FALSE
Twsg1	Hub	FALSE
Chl1	Hub	FALSE
Tox2	Hub	FALSE
Nlgn2	Hub	FALSE
Rpl13	Hub	FALSE
Ltbr	Hub	FALSE
Ndufa3	Hub	FALSE
Gbas	Hub	FALSE
Laptm4b	Hub	FALSE
2700060E0	Hub	FALSE
Slc35f1	Hub	FALSE
Rps28	Hub	FALSE
Lgmn	Hub	FALSE
Rgs16	Hub	FALSE
Plxnd1	Hub	FALSE
Hadhb	Hub	FALSE
Afap1l2	Hub	FALSE
Ahr	Hub	TRUE
Camk1	Hub	FALSE
Gm13826	Hub	FALSE
Cd276	Hub	FALSE
Egfl8	Hub	FALSE
Plgrkt	Hub	FALSE
Phlda3	Hub	FALSE
Gsk3b	Hub	FALSE
Srgap1	Hub	FALSE
Rps15	Hub	FALSE
Map2	Hub	FALSE
Hbp1	Hub	TRUE
Atp1b2	Hub	FALSE
Bmp1	Hub	FALSE
Eci2	Hub	FALSE
Haghl	Hub	FALSE
Erf	Hub	TRUE
Slc44a1	Hub	FALSE
0610031J0	Hub	FALSE
Sema3c	Hub	FALSE
Sema4c	Hub	FALSE
Gria3	Hub	FALSE
Surf4	Hub	FALSE

Akap2	Hub	FALSE
Clic1	Hub	FALSE
AW549542	Hub	FALSE
Il1rapl1	Hub	FALSE
Adamts5	Hub	FALSE
Epb4.1l4a	Hub	FALSE
Arl2bp	Hub	FALSE
Pcdh7	Hub	FALSE
Acot13	Hub	FALSE
Cep170	Hub	FALSE
Arid5b	Hub	TRUE
Fdx1	Hub	FALSE
Abhd4	Hub	FALSE
Vim	Hub	FALSE
Timp3	Hub	FALSE
Ttyh3	Hub	FALSE
Rnasel	Hub	FALSE
1700021K1	Hub	FALSE
Rcn3	Hub	FALSE
Taok1	Hub	FALSE
Cnn3	Hub	FALSE
Serf1	Hub	FALSE
2010111I0	Hub	FALSE
Ccdc23	Hub	FALSE
Schip1.3	Hub	FALSE
Ramp3	Hub	FALSE
Zfp827	Hub	FALSE
Opcml	Hub	FALSE
Wbp1	Hub	FALSE
Bcar1	Hub	FALSE
Jmjd1c	Hub	FALSE
Prkaca	Hub	FALSE
Spcs2	Hub	FALSE
Pnrc1	Hub	FALSE
Tmem9	Hub	FALSE
Fibp	Hub	FALSE
Foxp1	Hub	TRUE
Spred3	Hub	FALSE
Eci1	Hub	FALSE
Rit1	Hub	FALSE
Cbr3	Hub	FALSE
Sep-15	Hub	FALSE
Gng10	Hub	FALSE
Slc35b2	Hub	FALSE
Zfp60	Hub	FALSE
Parva	Hub	FALSE
Dennd5a	Hub	FALSE
Hs3st1	Hub	FALSE
Zyx	Hub	FALSE
1810058I2	Hub	FALSE

Rpl41	Hub	FALSE
Uggt1	Hub	FALSE
Coro2b	Hub	FALSE
Ing4	Hub	TRUE
Snx22	Hub	FALSE
Phf20l1	Hub	FALSE
LOC10050⁄	Hub	FALSE
Sema3d	Hub	FALSE
Lims2	Hub	FALSE
Rps21	Hub	FALSE
Tmc8	Hub	FALSE
Scarf2	Hub	FALSE
Arhgef9	Hub	FALSE
Dap	Hub	FALSE
Gtf2h5	Hub	FALSE
Fam102b	Hub	FALSE
Rtn4	Hub	FALSE
Mrps14	Hub	FALSE
Txndc12	Hub	FALSE
Chd6	Hub	FALSE
Myo9b	Hub	FALSE
Scp2	Hub	FALSE
Ldb2	Hub	FALSE
Rpl37	Hub	FALSE
Lamb2	Hub	FALSE
Nr4a2	Hub	TRUE
Commd3	Hub	FALSE
Creb3l2	Hub	TRUE
Smpdl3a	Hub	FALSE
Npc1	Hub	FALSE
Glipr2	Hub	FALSE
Plekhg5	Hub	FALSE
Uqcrh	Hub	FALSE
Tma7	Hub	FALSE
Ankrd29	Hub	FALSE
Ptpn21	Hub	FALSE
Vgll3	Hub	FALSE
Rassf4	Hub	FALSE
Fam115a	Hub	FALSE
Gm2a	Hub	FALSE
Cuedc2	Hub	FALSE
Mxd4	Hub	TRUE
Nkd2	Hub	FALSE
Mrpl52	Hub	FALSE
Mar-02	Hub	FALSE
Rab14	Hub	TRUE
Cstb	Hub	FALSE
Ei24	Hub	FALSE
Cpped1	Hub	FALSE
Ttyh1	Hub	FALSE

Gpx4	Hub	FALSE
Tpp1	Hub	FALSE
Pcyox1	Hub	FALSE
Mgat3	Hub	FALSE
St3gal1	Hub	FALSE
Plekhb1	Hub	FALSE
Mmp11	Hub	FALSE
Smim11	Hub	FALSE
6330403K0	Hub	FALSE
Josd2	Hub	FALSE
Tmem14c	Hub	FALSE
Rcsd1	Hub	FALSE
Mxra8	Hub	FALSE
2610027K0	Hub	FALSE
Ak3	Hub	FALSE
Arf5	Hub	FALSE
Cux1	Hub	TRUE
Adam19	Hub	FALSE
Tmem106b	Hub	FALSE
Ap3m2	Hub	FALSE
Ucp2	Hub	FALSE
AI414108	Hub	FALSE
Acadl	Hub	FALSE
Psmd8	Hub	FALSE
Rims1.1	Hub	FALSE
Pmepa1	Hub	FALSE
Ddx17	Hub	FALSE
Rgmb	Hub	FALSE
Shisa4	Hub	FALSE
Ldb1	Hub	FALSE
Etfb	Hub	TRUE
Jam2	Hub	FALSE
Ntan1	Hub	FALSE
Uba52	Hub	FALSE
Hadha	Hub	FALSE
Cox14	Hub	FALSE
Lamtor2	Hub	FALSE
Ppp2r2b	Hub	FALSE
Cebpzos	Hub	FALSE
Cfl1	Hub	FALSE
Nme3	Hub	FALSE
Zfand3	Hub	FALSE
Cflar	Hub	FALSE
Arfip2	Hub	FALSE
Tgfbr2	Hub	FALSE
Pde8b	Hub	FALSE
Lrrc8b	Hub	FALSE
Lyrm2	Hub	FALSE
Kdelr1	Hub	FALSE
Tle2	Hub	FALSE

2310045N(	Hub	FALSE
Tsc22d1	Hub	FALSE
Myrfl	Hub	FALSE
Matn4	Hub	FALSE
Trim13	Hub	FALSE
C1qtnf6	Hub	FALSE
Ppp1r14c	Hub	FALSE
Map1a	Hub	FALSE
Hint2	Hub	FALSE
2300009A(	Hub	FALSE
Palm	Hub	FALSE
Gstm7	Hub	FALSE
Lars2	Hub	FALSE
Mbnl2	Hub	TRUE
Ifngr1	Hub	FALSE
Erp29	Hub	FALSE
Tmem203	Hub	FALSE
Ap1s1	Hub	FALSE
Gpt2	Hub	FALSE
Tmem128	Hub	FALSE
Emp3	Hub	FALSE
Fundc2	Hub	FALSE
Tmem223	Hub	FALSE
Asah1	Hub	FALSE
Atp13a2	Hub	FALSE
Nucb1	Hub	TRUE
Lamtor4	Hub	FALSE
Hcn1	Hub	FALSE
Robo2	Hub	FALSE
Ftl1	Hub	FALSE
Rela	Hub	TRUE
Zfand6	Hub	FALSE
Chd3	Hub	FALSE
Mob4	Hub	FALSE
Ist1	Hub	FALSE
Bmpr2	Hub	FALSE
Tead1	Hub	TRUE
Ndufa4	Hub	FALSE
Prr14	Hub	FALSE
Kdm6b	Hub	FALSE
Dbn1	Hub	FALSE
Mef2d	Hub	TRUE
Cdc42ep5	Hub	FALSE
Cklf	Hub	FALSE
Tspan31	Hub	FALSE
Tomm7	Hub	FALSE
Aak1	Hub	FALSE
Swi5	Hub	FALSE
Atxn7l3b	Hub	FALSE
Thsd7a	Hub	FALSE

Rapgef2	Hub	FALSE
Nod1	Hub	FALSE
Tmed9	Hub	FALSE
Tmem176a	Hub	FALSE
Cnpy2	Hub	FALSE
Btd	Hub	FALSE
Tmem176b	Hub	FALSE
Sdhb	Hub	FALSE
Fkbp7	Hub	FALSE
Gnb5	Hub	FALSE
R3hdm4	Hub	FALSE
Toporsos	Hub	FALSE
Cacfd1	Hub	FALSE
Uba7	Hub	FALSE
Pnpla3	Hub	FALSE
Ppox	Hub	FALSE
Sorbs1	Hub	FALSE
Wdr6	Hub	FALSE
Phpt1	Hub	FALSE
H1fx	Hub	TRUE
Alcam	Hub	FALSE
Ext2	Hub	FALSE
Ogt	Hub	FALSE
Ccs	Hub	FALSE
Nynrin	Hub	FALSE
Kank4	Hub	FALSE
Ndufb5	Hub	FALSE
Apold1	Hub	FALSE
Fam65a	Hub	FALSE
Fus	Hub	FALSE
Slc25a4	Hub	FALSE
Cdk6	Hub	FALSE
Plxna4	Hub	FALSE
Ndufa7	Hub	FALSE
Chd2	Hub	TRUE
Dlgap4	Hub	FALSE
Lbx2	Hub	TRUE
Mtss1	Hub	FALSE
Rp9	Hub	FALSE
Mid2	Hub	FALSE
Zfp467	Hub	TRUE
Evi5	Hub	FALSE
P2ry1	Hub	FALSE
Arhgef2	Hub	FALSE
Apoa1bp	Hub	FALSE
Sdhc	Hub	FALSE
Isca2	Hub	FALSE
Frmd6	Hub	FALSE
Irf3	Hub	TRUE
Cyp4f16	Hub	FALSE

Kcnt2	Hub	FALSE
Ccdc104	Hub	FALSE
1110038F1	Hub	FALSE
Cyth1	Hub	FALSE
Pcmtd2	Hub	FALSE
Tecpr1	Hub	FALSE
Esr1	Hub	TRUE
Tagln2	Hub	TRUE
Capn6	Hub	FALSE
Sox6	Hub	TRUE
Jun	Hub	TRUE
Zdhhc12	Hub	FALSE
Otor	Hub	FALSE
Mpz	Hub	FALSE
Ranbp10	Hub	FALSE
Kdr	Hub	FALSE
Laptm4a	Hub	FALSE
Lcp2	Hub	FALSE
Pja2	Hub	FALSE
Nedd8	Hub	FALSE
Bin1	Hub	FALSE
Eif1b	Hub	FALSE
Zmynd11	Hub	FALSE
Rnaset2b	Hub	FALSE
Crtc1	Hub	FALSE
Stim1	Hub	FALSE
Capn2	Hub	FALSE
Gemin7	Hub	FALSE
2410015M	Hub	FALSE
Hif3a	Hub	TRUE
Atp5o	Hub	FALSE
Otub1	Hub	FALSE
Prickle2	Hub	FALSE
Arid4b	Hub	FALSE
Tbca	Hub	FALSE
Atp5c1	Hub	FALSE
Ssr2	Hub	FALSE
Ndufs3	Hub	FALSE
Igsf9b	Hub	FALSE
Rbm24	Hub	FALSE
Dag1	Hub	FALSE
Furin	Hub	FALSE
Nbea	Hub	FALSE
H3f3a	Hub	FALSE
Snx17	Hub	FALSE
Polr3gl	Hub	FALSE
Gm16907	Hub	FALSE
Gm6402	Hub	FALSE
Mageh1	Hub	FALSE
1190002F1	Hub	FALSE

Fam115c	Hub	FALSE
Tmem50a	Hub	FALSE
Tmem134	Hub	FALSE
Tead3	Hub	TRUE
Uqcc2	Hub	FALSE
Ndufs8	Hub	FALSE
Dexi	Hub	FALSE
Smim4	Hub	FALSE
Prr12	Hub	FALSE
Acvrl1	Hub	FALSE
Pgls	Hub	FALSE
Coro1b	Hub	FALSE
Jup	Hub	FALSE
Zswim6	Hub	FALSE
Edaradd	Hub	FALSE
Nrcam	Hub	FALSE
Arhgef7	Hub	FALSE
Pick1	Hub	TRUE
Ccnl1	Hub	FALSE
Aph1b	Hub	FALSE
Stra13	Hub	FALSE
Phf2	Hub	TRUE
Snord104	Hub	FALSE
Fam89b	Hub	FALSE
Il11ra1	Hub	FALSE
Vkorc1	Hub	FALSE
Emc3	Hub	FALSE
Krtcap2	Hub	FALSE
Dock5	Hub	FALSE
Cck	Hub	FALSE
Scamp2	Hub	FALSE
Ctxn1	Hub	FALSE
Alg14	Hub	FALSE
Armcx2	Hub	FALSE
Cuta	Hub	FALSE
4930579G1	Hub	FALSE
Leprot	Hub	FALSE
Med12l	Hub	FALSE
Tmem63b	Hub	FALSE
Tmem258	Hub	FALSE
Tmbim6	Hub	FALSE
Gnas	Hub	FALSE
Pigt	Hub	FALSE
Pik3ip1	Hub	FALSE
Mro	Hub	FALSE
2610307P1	Hub	FALSE
Ndufa2	Hub	FALSE
Jtb	Hub	FALSE
Qpct	Hub	FALSE
Rpl35	Hub	TRUE

Cadps2	Hub	FALSE
Dapp1	Hub	FALSE
Prmt2	Hub	FALSE
Cd81	Hub	FALSE
Carkd	Hub	FALSE
Osbpl5	Hub	FALSE
Mink1	Hub	FALSE
Pthlh	Hub	FALSE
Arhgap31	Hub	FALSE
Sipa1l2	Hub	FALSE
Unc93b1	Hub	FALSE
Agmo	Hub	FALSE
Serf2	Hub	FALSE
Fopnl	Hub	FALSE
Csf2ra	Hub	FALSE
Tomm6	Hub	FALSE
Ptger1	Hub	FALSE
Sars	Hub	FALSE
Gpr137b	Hub	FALSE
Jmjd8	Hub	FALSE
Cd200	Hub	FALSE
Angpt2	Hub	FALSE
Phka1	Hub	FALSE
Map7d1	Hub	FALSE
Tmed4	Hub	FALSE
Gtl3	Hub	FALSE
4933413L0	Hub	FALSE
Jkamp	Hub	FALSE
Ap3s1	Hub	FALSE
Anxa2	Hub	FALSE
Ndufa12	Hub	FALSE
Cyhr1	Hub	FALSE
Srsf5	Hub	FALSE
Itsn2	Hub	FALSE
Psme2b	Hub	FALSE
Commd6	Hub	FALSE
Il1rl2	Hub	FALSE
Lrrtm1	Hub	FALSE
Dyrk1b	Hub	FALSE
Eps15	Hub	FALSE
Ict1	Hub	FALSE
Nisch	Hub	FALSE
Ets2	Hub	TRUE
Tmem234	Hub	FALSE
Slc22a23	Hub	FALSE
Ssna1	Hub	FALSE
Shkbp1	Hub	FALSE
Gng7	Hub	FALSE
Tnrc6b	Hub	FALSE
Fxyd5	Hub	FALSE

Atp1a1	Hub	FALSE
Sema6b	Hub	FALSE
Ncstn	Hub	FALSE
Tecrl	Hub	FALSE
Gm5607	Hub	FALSE
Rbm5	Hub	FALSE
Ik	Hub	FALSE
Txndc16	Hub	FALSE
Stx4a	Hub	FALSE
Sgk3	Hub	FALSE
Tnk2	Hub	FALSE
Eif3k	Hub	FALSE
Med28	Hub	FALSE
Arpc2	Hub	FALSE
Nt5dc3	Hub	FALSE
Dab2ip	Hub	FALSE
Dpysl4	Hub	FALSE
Glb1	Hub	FALSE
Hdac5	Hub	FALSE
1810037I1	Hub	FALSE
Atp5j	Hub	FALSE
Vwa5a	Hub	FALSE
Crip2	Hub	FALSE
Rab29	Hub	FALSE
Pkd1	Hub	FALSE
Acp2	Hub	FALSE
Sct	Hub	FALSE
Grik5	Hub	FALSE
Cyr61	Hub	FALSE
Zc3h12c	Hub	FALSE
Hes6	Hub	TRUE
Ufc1	Hub	FALSE
Bcl11b	Hub	TRUE
4930402H2	Hub	FALSE
Prss23	Hub	FALSE
Nckap5	Hub	FALSE
Msrb2	Hub	FALSE
Capns1	Hub	FALSE
Psen2	Hub	FALSE
Plec	Hub	FALSE
Spata24	Hub	FALSE
Lmo1	Hub	FALSE
Prkcdbp	Hub	FALSE
Edf1	Hub	FALSE
Brinp1	Hub	FALSE
Chchd7	Hub	FALSE
Nr4a1	Hub	TRUE
Mrps21	Hub	FALSE
Uqcrb	Hub	TRUE
Rpp21	Hub	FALSE

Dnajc7	Hub	FALSE
Aamdc	Hub	FALSE
Bckdha	Hub	FALSE
Olfml2b	Hub	FALSE
Tvp23b	Hub	FALSE
Sema3b	Hub	FALSE
Tlr3	Hub	FALSE
Smarcd3	Hub	FALSE
Nktr	Hub	FALSE
Nudt16l1	Hub	FALSE
Calcoco1	Hub	FALSE
Aup1	Hub	FALSE
Slc40a1	Hub	FALSE
Rps15a-ps6	Hub	FALSE
Bcl11a	Hub	TRUE
Fam114a2	Hub	FALSE
Abca1	Hub	FALSE
Tbc1d23	Hub	FALSE
Etv1	Hub	TRUE
Ube2w	Hub	FALSE
Brk1	Hub	FALSE
Cox6c	Hub	FALSE
Rbm39	Hub	FALSE
Mir6236	Hub	FALSE
Plscr3	Hub	FALSE
Scaf1	Hub	FALSE
Gab2	Hub	FALSE
Rnf19a	Hub	FALSE
1810026B0	Hub	FALSE
Lamtor1	Hub	FALSE
Mocs2	Hub	FALSE
Gsn	Hub	FALSE
Pik3r3	Hub	FALSE
Manba	Hub	FALSE
Mrpl34	Hub	FALSE
Spaca6	Hub	FALSE
Gkap1	Hub	FALSE
Evi5l	Hub	FALSE
Ssh2	Hub	FALSE
Htr3a	Hub	FALSE
Clip3	Hub	FALSE
Mrps33	Hub	FALSE
2700081O1	Hub	FALSE
Cox6b1	Hub	FALSE
Psmb5	Hub	FALSE
Cisd3	Hub	FALSE
Cd1d1	Hub	FALSE
Narf	Hub	FALSE
Kcna6	Hub	FALSE
Gga1	Hub	FALSE

Nox4	Hub	FALSE
Uqcr10	Hub	FALSE
Cpt1c	Hub	FALSE
Glrx2	Hub	FALSE
Spry1	Hub	FALSE
Aifm2	Hub	FALSE
Ndufc2	Hub	FALSE
Idh3b	Hub	FALSE
D630045J1	Hub	FALSE
Ptprf	Hub	FALSE
Fam222a	Hub	FALSE
Kctd17	Hub	FALSE
Atf5	Hub	TRUE
P2rx4	Hub	FALSE
Slc25a23	Hub	FALSE
Arhgef3	Hub	FALSE
Zfp523	Hub	TRUE
Stk25	Hub	FALSE
C130071C0	Hub	FALSE
Sidt2	Hub	FALSE
Snap47	Hub	FALSE
Ndufa6	Hub	FALSE
Cux2	Hub	TRUE
Zfp513	Hub	TRUE
Pbx4	Hub	TRUE
Gm16617	Hub	FALSE
Zfhx3	Hub	TRUE
Eif3h	Hub	FALSE
Mapk3	Hub	FALSE
Nrep	Hub	FALSE
Gamt	Hub	FALSE
Akt1s1	Hub	FALSE
Tesk1	Hub	FALSE
Cox8a	Hub	FALSE
Galt	Hub	FALSE
Scarb2	Hub	FALSE
Fgfrl1	Hub	FALSE
Rnf217	Hub	FALSE
Znf512b	Hub	FALSE
Dpm3	Hub	FALSE
Gdpd2	Hub	FALSE
Zdhhc8	Hub	FALSE
Cap2	Hub	FALSE
Rras	Hub	FALSE
Uqcrq	Hub	FALSE
Sra1	Hub	FALSE
Sh3glb2	Hub	FALSE
Bcl7a	Hub	FALSE
Bcl2l2	Hub	FALSE
Loxl4	Hub	FALSE

Faim	Hub	FALSE
G0s2	Hub	FALSE
A730017L2	Hub	FALSE
B2m	Hub	FALSE
Speg	Hub	FALSE
Fzd1	Hub	FALSE
Zswim8	Hub	FALSE
Dlx5	Hub	TRUE
Prkca	Hub	FALSE
Arntl	Hub	TRUE
Astn1	Hub	FALSE
Lpcat2	Hub	FALSE
Klc4	Hub	FALSE
Pfdn5	Hub	FALSE
Fzd8	Hub	FALSE
Hdac4	Hub	FALSE
Plxnb3	Hub	FALSE
Gm2382	Hub	FALSE
Fam168a	Hub	FALSE
Riiad1	Hub	FALSE
Ift20	Hub	FALSE
Chpf2	Hub	FALSE
Ddc	Hub	FALSE
Gm10863	Hub	FALSE
Luc7l3	Hub	FALSE
Mbd6	Hub	FALSE
Dip2b	Hub	FALSE
Phyh	Hub	FALSE
Mrpl24	Hub	FALSE
Ccnl2	Hub	FALSE
Nbr1	Hub	FALSE
Npc2	Hub	FALSE
Tagln	Hub	FALSE
Trim35	Hub	FALSE
Stx5a	Hub	FALSE
Bad	Hub	TRUE
Nalcn	Hub	FALSE
Rpl38	Hub	FALSE
Ypel5	Hub	FALSE
Tdrkh	Hub	FALSE
Cwc15	Hub	FALSE
Smim20	Hub	FALSE
Kif1b	Hub	FALSE
Mtch1	Hub	FALSE
Naa38	Hub	FALSE
Mrps24	Hub	FALSE
Itga2	Hub	FALSE
Atp8a1	Hub	FALSE
Higd2a	Hub	FALSE
Acaa1a	Hub	TRUE

Immp1l	Hub	FALSE
Cntln	Hub	FALSE
Fam195b	Hub	FALSE
D8Ertd738	Hub	FALSE
Elovl1	Hub	FALSE
Atp1a2	Hub	FALSE
Synrg	Hub	FALSE
Rgs2	Hub	FALSE
Abcd2	Hub	FALSE
2310039H(	Hub	FALSE
Shc1	Hub	FALSE
Wdsub1	Hub	FALSE
Dhh	Hub	FALSE
Fam214b	Hub	FALSE
Ndst1	Hub	FALSE
Nab1	Hub	FALSE
Fam214a	Hub	FALSE
Tecta	Hub	FALSE
Nudt13	Hub	FALSE
Creg1	Hub	FALSE
Kmt2d	Hub	FALSE
Gpr153	Hub	FALSE
Tmem208	Hub	FALSE
Krt10	Hub	FALSE
Cryab	Hub	FALSE
Dll3	Hub	FALSE
Qsox1	Hub	FALSE
Hsdl2	Hub	FALSE
Fbxo25	Hub	FALSE
Rc3h2	Hub	FALSE
Pacs2	Hub	FALSE
Tle6	Hub	FALSE
D10Jhu81e	Hub	FALSE
Trib2	Hub	FALSE
Fam134b	Hub	FALSE
Atp11a	Hub	FALSE
Itpr3	Hub	FALSE
Gtpbp2	Hub	FALSE
Sumf1	Hub	FALSE
Mettl23	Hub	FALSE
Txndc9	Hub	FALSE
Ggh	Hub	FALSE
D330023K1	Hub	FALSE
Chchd2	Hub	FALSE
Golga4	Hub	FALSE
Ptgfrn	Hub	FALSE
Gm3258	Hub	FALSE
Mlxip	Hub	TRUE
Fbxw5	Hub	FALSE
Pla2r1	Hub	FALSE

Tmx4	Hub	FALSE
Pnpla8	Hub	FALSE
Plxnb2	Hub	FALSE
0610012G(	Hub	FALSE
Mfsd11	Hub	FALSE
Gm14057	Hub	FALSE
Palm2	Hub	FALSE
S100a10	Hub	FALSE
Epn1	Hub	FALSE
Bcap31	Hub	FALSE
Tbx20	Hub	TRUE
Zfp395	Hub	FALSE
Plekha4	Hub	FALSE
Tm4sf1	Hub	FALSE
Rnf182	Hub	FALSE
Sdf2	Hub	FALSE
Tmem241	Hub	FALSE
Mrpl14	Hub	FALSE
Flrt2	Hub	FALSE
Fuom	Hub	FALSE
Pear1	Hub	FALSE
Slc25a12	Hub	FALSE
Rdh14	Hub	FALSE
Mthfr	Hub	FALSE
Bend5	Hub	FALSE
Paip2	Hub	FALSE
Atp5h	Hub	FALSE
Ip6k1	Hub	FALSE
Insl6	Hub	FALSE
Cntn6	Hub	FALSE
Ccng2	Hub	FALSE
Ash1l	Hub	FALSE
Rnpep	Hub	FALSE
Frg1	Hub	FALSE
Lztr1	Hub	FALSE
Polr2g	Hub	FALSE
B4galt3	Hub	FALSE
Msrb1	Hub	FALSE
Erlec1	Hub	FALSE
Il3ra	Hub	FALSE
Ptov1	Hub	FALSE
Psme2	Hub	FALSE
Iffo1	Hub	FALSE
Grid2	Hub	FALSE
Dok1	Hub	FALSE
Rock1	Hub	FALSE
C1galt1c1	Hub	FALSE
Farp2	Hub	FALSE
Erp44	Hub	FALSE
Rcan3	Hub	FALSE

Sec61b	Hub	FALSE
Foxn3	Hub	TRUE
Myl6	Hub	FALSE
Itpkb	Hub	FALSE
Lrrc8c	Hub	FALSE
Pxmp4	Hub	FALSE
Gps2	Hub	FALSE
Crym	Hub	FALSE
Anapc16	Hub	FALSE
Flrt1	Hub	FALSE
Mpnd	Hub	FALSE
Fndc3b	Hub	FALSE
Cxx1b	Hub	TRUE
Cdk19	Hub	FALSE
Hp1bp3	Hub	TRUE
Znrf1	Hub	FALSE
Smim24	Hub	FALSE
Gpcpd1	Hub	FALSE
2010107E0	Hub	FALSE
Chmp5	Hub	FALSE
Rec8	Hub	FALSE
Mcee	Hub	FALSE
Baiap2	Hub	FALSE
Tmem104	Hub	FALSE
Sp3os	Hub	FALSE
Aktip	Hub	FALSE
Hspb11	Hub	FALSE
S100b	Hub	FALSE
Cox6b2	Hub	FALSE
Col3a1	Hub	FALSE
Kif3a	Hub	FALSE
Gm10789	Hub	FALSE
Insr	Hub	FALSE
Ilvbl	Hub	FALSE
Pik3r1	Hub	FALSE
2310030G(	Hub	FALSE
Dad1	Hub	FALSE
Fbxl20	Hub	FALSE
Xylt1	Hub	FALSE
Amfr	Hub	FALSE
Sec24d	Hub	FALSE
Lemd3	Hub	FALSE
Caml	Hub	FALSE
Urod	Hub	FALSE
Enpp4	Hub	FALSE
Glt8d1	Hub	FALSE
Nfib	Hub	TRUE
Bri3	Hub	FALSE
Rer1	Hub	FALSE
Git2	Hub	TRUE

Ndufa13	Hub	FALSE
Ncor2	Hub	TRUE
Zfp945	Hub	FALSE
Sf3b6	Hub	FALSE
Cyp2j9	Hub	FALSE
Stat3	Hub	TRUE
Mamld1	Hub	FALSE
Dpm2	Hub	FALSE
Pnisr	Hub	FALSE
Dact3	Hub	FALSE
Akr1cl	Hub	FALSE
Trmt2b	Hub	FALSE
Ddah2	Hub	FALSE
Zfyve1	Hub	FALSE
Hand2	Hub	TRUE
Anapc13	Hub	FALSE
Vsnl1	Hub	FALSE
Cbr1	Hub	FALSE
Atp6ap2	Hub	FALSE
Slc22a17	Hub	FALSE
Fam160a2	Hub	FALSE
Phkg2	Hub	FALSE
Hspb2	Hub	FALSE
Adprh	Hub	FALSE
A330049N(	Hub	FALSE
Itfg1	Hub	FALSE
Ndufs6	Hub	FALSE
Pcdhga12	Hub	FALSE
Tmem191c	Hub	FALSE
Ndufb3	Hub	FALSE
Gramd1a	Hub	FALSE
9330188P(	Hub	FALSE
Sec14l2	Hub	FALSE
1700113A1	Hub	FALSE
Grcc10	Hub	FALSE
Tmx2	Hub	FALSE
Cpm	Hub	FALSE
Rgs3	Hub	FALSE
Deaf1	Hub	TRUE
Nanos3	Hub	FALSE
Nradd	Hub	FALSE
Dmpk	Hub	FALSE
Tmem119	Hub	FALSE
Ankrd46	Hub	FALSE
Tmem88b	Hub	FALSE
Mib2	Hub	FALSE
Rin3	Hub	FALSE
Nron	Hub	FALSE
Sh3gl3	Hub	FALSE
Rnf13	Hub	FALSE

Ddost	Hub	FALSE
Tmsb15b1	Hub	FALSE
Cdk5	Hub	FALSE
1810022K0	Hub	FALSE
Scand1	Hub	FALSE
Rraga	Hub	FALSE
Nfia	Hub	TRUE
5930403L1	Hub	FALSE
Trerf1	Hub	TRUE
Yipf3	Hub	FALSE
Fuz	Hub	FALSE
Agtrap	Hub	FALSE
Mthfs	Hub	FALSE
Cox20	Hub	FALSE
Lonp2	Hub	FALSE
Exoc4	Hub	FALSE
Abtb2	Hub	FALSE
Slc44a2	Hub	FALSE
Shank1	Hub	FALSE
Hn1	Hub	FALSE
Creld1	Hub	FALSE
G530011O(	Hub	FALSE
Ksr1	Hub	FALSE
Pld3	Hub	FALSE
Lancl1	Hub	FALSE
Pttg1ip	Hub	FALSE
Rnf11	Hub	FALSE
Athl1	Hub	FALSE
Mrpl27	Hub	FALSE
Fendrr	Hub	FALSE
Rnf157	Hub	FALSE
Sin3b	Hub	FALSE
Eps8	Hub	FALSE
2900026A(	Hub	FALSE
Mob3c	Hub	FALSE
Lrrc8a	Hub	FALSE
Lrch1	Hub	FALSE
Timm8b	Hub	FALSE
Rsu1	Hub	FALSE
Pfkfb4	Hub	FALSE
Ier3ip1	Hub	FALSE
Neu1	Hub	FALSE
Strn4	Hub	FALSE
Slc2a8	Hub	FALSE
Exoc6b	Hub	FALSE
Arhgap22	Hub	FALSE
Pet100	Hub	FALSE
Shisa5	Hub	FALSE
Zmiz2	Hub	FALSE
Lrrn1	Hub	FALSE

Tubb4a	Hub	FALSE
Carhsp1	Hub	FALSE
Dhrs1	Hub	FALSE
Gfra3	Hub	FALSE
Slc25a17	Hub	FALSE
Lemd2	Hub	FALSE
Spcs1	Hub	FALSE
1110065P2	Hub	FALSE
Gm5617	Hub	FALSE
Tmem40	Hub	FALSE
Slc30a9	Hub	FALSE
Akap8l	Hub	FALSE
Slc16a7	Hub	FALSE
Chmp1b	Hub	FALSE
Snapin	Hub	FALSE
Stk32a	Hub	FALSE
Cope	Hub	FALSE
Slc27a4	Hub	FALSE
Usp11	Hub	FALSE
Dclk2	Hub	FALSE
Fam120aos	Hub	FALSE
Sun1	Hub	FALSE
Tspan13	Hub	FALSE
Zfp637	Hub	TRUE
Ppargc1a	Hub	TRUE
Tnni1	Hub	FALSE
Hist1h2ag	Hub	FALSE
Ncor1	Hub	TRUE
Bcl2l1	Hub	FALSE
Tm7sf3	Hub	FALSE
Nab2	Hub	FALSE
Clk1	Hub	TRUE
Ebf4	Hub	TRUE
Rnf150	Hub	FALSE
Snrnp27	Hub	FALSE
C1galt1	Hub	FALSE
Hdac8	Hub	TRUE
Atp5j2	Hub	FALSE
Crtc3	Hub	FALSE
Limk1	Hub	FALSE
Sec61g	Hub	FALSE
Rasgrp2	Hub	FALSE
Tulp4	Hub	FALSE
Lmna	Hub	FALSE
Rab1b	Hub	FALSE
Rhoa	Hub	FALSE
Prkd2	Hub	FALSE
Ticam1	Hub	FALSE
1110058L1	Hub	FALSE
Plxdc2	Hub	FALSE

Trappc6a	Hub	FALSE
Lrp12	Hub	FALSE
Agpat1	Hub	FALSE
Syap1	Hub	FALSE
Pde8a	Hub	FALSE
Rnh1	Hub	FALSE
Hexb	Hub	FALSE
Ndufc1	Hub	FALSE
Kmt2a	Hub	TRUE
Rnf135	Hub	FALSE
Mbd2	Hub	TRUE
Gpm6a	Hub	FALSE
Dnajb2	Hub	FALSE
Arl6ip5	Hub	FALSE
Plekhm2	Hub	FALSE
1500011K1	Hub	FALSE
Ypel3	Hub	FALSE
Atp6v0e2	Hub	FALSE
Lrrc4b	Hub	FALSE
2610206C1	Hub	FALSE
Sbk1	Hub	FALSE
Galnt10	Hub	FALSE
Chst7	Hub	FALSE
Rxrg	Hub	TRUE
Urgcp	Hub	FALSE
Mast3	Hub	FALSE
Sfxn4	Hub	FALSE
Ggact	Hub	FALSE
A330076H(	Hub	FALSE
Ccdc82	Hub	FALSE
Fam19a5	Hub	FALSE
Mff	Hub	FALSE
Slc25a11	Hub	FALSE
I730030J21	Hub	FALSE
Gm1673	Hub	FALSE
Prrc2b	Hub	FALSE
Pigyl	Hub	FALSE
Ctsa	Hub	FALSE
Gap43	Hub	FALSE
Jak2	Hub	FALSE
Psmb1	Hub	FALSE
2310015A1	Hub	FALSE
Dctn5	Hub	FALSE
Kcne1l	Hub	FALSE
Aff2	Hub	FALSE
Vps9d1	Hub	FALSE
Mpped2	Hub	FALSE
Slc12a6	Hub	FALSE
Mprip	Hub	FALSE
Secisbp2l	Hub	FALSE

Nhs	Hub	FALSE
Dnajc18	Hub	FALSE
Srebf1	Hub	TRUE
Rnf215	Hub	FALSE
Mon2	Hub	FALSE
Npas3	Hub	FALSE
Nfkbiz	Hub	FALSE
Egln2	Hub	FALSE
Psme1	Hub	FALSE
Creb3	Hub	TRUE
Cadm4	Hub	FALSE
Klhl24	Hub	FALSE
Cryzl1	Hub	FALSE
Mgrn1	Hub	FALSE
Tspo	Hub	FALSE
Hacl1	Hub	FALSE
Gm15663	Hub	FALSE
Mospd3	Hub	FALSE
Ccdc142	Hub	FALSE
Slc25a45	Hub	FALSE
Dmrta1	Hub	TRUE
Cox7c	Hub	FALSE
Araf	Hub	FALSE
Ppp2r5c	Hub	FALSE
Fis1	Hub	FALSE
Tmed3	Hub	FALSE
Rffl	Hub	FALSE
Gnb4	Hub	FALSE
Chrm1	Hub	FALSE
Otud1	Hub	FALSE
Zfp93	Hub	TRUE
Pqbp1	Hub	TRUE
Snx25	Hub	FALSE
Creb5	Hub	TRUE
Gm7694	Hub	FALSE
Hsd3b7	Hub	FALSE
2610203C2	Hub	FALSE
Tspyl3	Hub	FALSE
Mocs1	Hub	FALSE
Rfx1	Hub	TRUE
Lamtor5	Hub	FALSE
Zfp810	Hub	FALSE
Ager	Hub	FALSE
Cdipt	Hub	FALSE
Scamp3	Hub	FALSE
Ubl5	Hub	FALSE
Acox1	Hub	FALSE
Cd99l2	Hub	FALSE
Dync1i2	Hub	FALSE
Dennd1b	Hub	FALSE

Dnpep	Hub	FALSE
Plcb2	Hub	FALSE
Ccdc47	Hub	FALSE
F630111L1	Hub	FALSE
Herc3	Hub	FALSE
Calhm2	Hub	FALSE
Pdgfb	Hub	FALSE
Zdhhc1	Hub	FALSE
Ppm1k	Hub	FALSE
Rab11a	Hub	FALSE
2810013P0	Hub	FALSE
Cpq	Hub	FALSE
Hipk3	Hub	FALSE
Chsy3	Hub	FALSE
Praf2	Hub	FALSE
Whamm	Hub	FALSE
Snf8	Hub	FALSE
Rnf145	Hub	FALSE
Rpph1	Hub	FALSE
Trp53i13	Hub	FALSE
Atp5g3	Hub	FALSE
Fzd6	Hub	FALSE
Nfu1	Hub	FALSE
Nat6	Hub	FALSE
Mfng	Hub	FALSE
Cxx1a	Hub	TRUE
Gsk3a	Hub	FALSE
Car6	Hub	FALSE
Dcaf6	Hub	FALSE
Slc37a2	Hub	FALSE
Supt4a	Hub	FALSE
Adam22	Hub	FALSE
Pld1	Hub	FALSE
Polr2i	Hub	FALSE
Syvn1	Hub	FALSE
Myo9a	Hub	FALSE
Sec62	Hub	FALSE
Pold4	Hub	FALSE
Mpc2	Hub	FALSE
Ssr4	Hub	FALSE
Tpd52	Hub	FALSE
Nphp3	Hub	FALSE
Zmym5	Hub	FALSE
2210016L2	Hub	FALSE
Brox	Hub	FALSE
Ttc5	Hub	FALSE
Cox19	Hub	FALSE
Fkbp8	Hub	FALSE
Fam32a	Hub	FALSE
Ly6g6f	Hub	FALSE

Thbs2	Hub	FALSE
Ttc39c	Hub	FALSE
Tet2	Hub	FALSE
Iscu	Hub	FALSE
Glb1l	Hub	FALSE
AA465934	Hub	FALSE
Selm	Hub	FALSE
Mtmr7	Hub	FALSE
Mpp1	Hub	FALSE
Mmd2	Hub	FALSE
Hap1	Hub	FALSE
Zfp628	Hub	TRUE
C030037D(	Hub	FALSE
Lrrc1	Hub	FALSE
Txndc15	Hub	FALSE
Diablo	Hub	TRUE
Tacc2	Hub	FALSE
Ndufa11	Hub	FALSE
Drap1	Hub	FALSE
Ddhd1	Hub	FALSE
Dynll1	Hub	FALSE
Comt	Hub	FALSE
Stom	Hub	FALSE
Nol7	Hub	FALSE
Cic	Hub	TRUE
Pygo1	Hub	FALSE
Hsbp1	Hub	FALSE
Fam46a	Hub	FALSE
Rab11fip5	Hub	FALSE
Tlx2	Hub	TRUE
Pdgfd	Hub	FALSE
Kat2b	Hub	FALSE
Bnc2	Hub	TRUE
Sec11c	Hub	FALSE
Atp5d	Hub	FALSE
Apobec3	Hub	FALSE
Appl1	Hub	FALSE
Gabarap	Hub	FALSE
C030039L0	Hub	FALSE
Churc1	Hub	TRUE
Sdccag3	Hub	FALSE
Ubxn4	Hub	FALSE
Prdx6b	Hub	FALSE
4930412O:	Hub	FALSE
Bcl9l	Hub	FALSE
Zcwpw1	Hub	FALSE
Zfp30	Hub	TRUE
Fhit	Hub	FALSE
Dcxr	Hub	FALSE
Dusp3	Hub	FALSE

Hexa	Hub	FALSE
Ttc28	Hub	FALSE
Fam3a	Hub	FALSE
Cd40	Hub	FALSE
Arhgap39	Hub	FALSE
Fbrs	Hub	FALSE
Trappc9	Hub	FALSE
Gm13315	Hub	FALSE
Lamp2	Hub	FALSE
Stk33	Hub	FALSE
Sar1b	Hub	FALSE
Rab3gap2	Hub	FALSE
Ly96	Hub	FALSE
Zfhx2	Hub	TRUE
Dnm2	Hub	FALSE

Common genes shared between neural crest and "hub" cells. Transcription factors (TFs) are designated

in the "TF" column with "TRUE"
