# Supplementary material for: Schwann cell precursors represent a neural crest‐like state with biased multipotency
Source: EMBO J. 2022 Jul 11;41(17):e108780. doi: 10.15252/embj.2021108780 (PMC9434083; doi:10.15252/embj.2021108780)
Supplement: Supplementary file 4 [file EMBJ-41-e108780-s004.pdf]

THE EMBO JOURNAL

Schwann cell precursors represent a neural crest-like state with biased multipotency

Maria Kastriti, Louis Faure, Dorothea von Ahsen, Thibault Bouderlique, Johan Boström, Tatiana Solovieva, Cameron Jackson, Marianne Bronner, Dies Meijer, Saida Hadjab-Lallemend, francois Lallemend, Alek Erickson, Marketa Kaucka, Viacheslav Dyachuk, Thomas Perlmann, Laura Lahti, Jan Krivanek, Jean-Francois Brunet, Kaj Fried, and Igor Adameyko

DOI: 10.15252/embj.2021108780

Corresponding author(s): Igor Adameyko (igor.adameyko@ki.se)

Review Timeline:

Submission Date:	20th May 21
Editorial Decision:	2nd Jul 21
Revision Received:	20th Apr 22
Editorial Decision:	31st May 22
Revision Received:	14th Jun 22
Accepted:	15th Jun 22

Editor: Daniel Klimmeck

Transaction Report:

(Note: With the exception of the correction of typographical or spelling errors that could be a source of ambiguity, letters and reports are not edited. Depending on transfer agreements, referee reports obtained elsewhere may or may not be included in this compilation. Referee reports are anonymous unless the Referee chooses to sign their reports.)

1st Editorial Decision 2nd Jul 2021

Dear Igor,

Thank you for the submission of your manuscript (EMBOJ-2021-108780) to The EMBO Journal. Please accept my apologies for the extended duration of the peer-review process due to protracted referee input and detailed discussions in the team. Your manuscript has been sent to four reviewers, referees #1 to #3 with neuro-developmental expertise and referee #4 being a single-cell bioinformatician. We have received reports from three of them, which I enclose below. There is still one review pending, which got much delayed. However in the interest of time, we have now decided to base our decision on the three existing reviews.

As you will see, the referees acknowledge the good quality and robustness of your analysis, and also indicate interest and potential novelty of your results and value as a resource to the field, although they also express a number of issues that will have to be conclusively addressed before they can be supportive of publication of your manuscript in The EMBO Journal. In more detail, referee #2 states that the reported hub state is not comprehensively characterized, and various claims made insufficiently supported (ref#2, pt.3). This expert is also concerned that the related in situ context is not well documented and discrepancies to existing literature too little discussed (ref#2, pts1,2; 5,6). In line, reviewer #1 finds that spatial context, developmental kinetics and functional dependencies of the hub state are inconclusively addressed (ref#1, pts. 1-3, see also ref#2 stand first). Referee #4 states that while per se thorough, the regulon calculation should be revised in order to capture a comprehensive view; further, technical biases are to be excluded to achieve the level of robustness and clarity needed for The EMBO Journal.

I judge the comments of the referees to be generally reasonable and given their overall interest, we are in principle happy to invite you to revise your manuscript experimentally to address the referees' comments,
pending the outstanding report by the fourth referee will not raise substantial technical issues with the analysis.

I will inform you as soon as I have received the report from this expert.

Please let me know any time if you have additional questions or need further input on the referee comments.

In light of the criticism raise by the reviewers, I would appreciate if you could contact me during the next weeks via e.g. a video call to discuss your perspective on the comments and potential plan for revisions.

Please see below for additional instructions for preparing your revised manuscript.

We generally allow three months as standard revision time. As a matter of policy, competing manuscripts published during this period will not negatively impact on our assessment of the conceptual advance presented by your study. However, we request that you contact the editor as soon as possible upon publication of any related work, to discuss how to proceed. Should you foresee a problem in meeting this three-month deadline, please let us know in advance and we may be able to grant an extension.

Thank you for the opportunity to consider your work for publication. I look forward to your revision.

Kind regards,

Daniel

Daniel Klimmeck, PhD
Senior Editor
The EMBO Journal

****

Instructions for preparing your revised manuscript:

When submitting your revised manuscript, please carefully review the instructions below and include the following items:

1) a .docx formatted version of the manuscript text (including legends for main figures, EV figures and tables). Please make sure that the changes are highlighted to be clearly visible.

2) individual production quality figure files as .eps, .tif, .jpg (one file per figure).

3) a .docx formatted letter INCLUDING the reviewers' reports and your detailed point-by-point response to their comments. As part of the EMBO Press transparent editorial process, the point-by-point response is part of the Review Process File (RPF), which will be published alongside your paper.

4) a complete author checklist, which you can download from our author guidelines (https://wol-prod-cdn.literatumonline.com/pb-assets/embo-site/Author Checklist%20-%20EMBO%20J-1561436015657.xlsx). Please insert information in the checklist that is also reflected in the manuscript. The completed author checklist will also be part of the RPF.

5) Please note that all corresponding authors are required to supply an ORCID ID for their name upon submission of a revised manuscript.

6) It is mandatory to include a 'Data Availability' section after the Materials and Methods. Before submitting your revision, primary datasets produced in this study need to be deposited in an appropriate public database, and the accession numbers and database listed under 'Data Availability'. Please remember to provide a reviewer password if the datasets are not yet public (see https://www.embopress.org/page/journal/14602075/authorguide#datadeposition).
In case you have no data that requires deposition in a public database, please state so in this section. Note that the Data Availability Section is restricted to new primary data that are part of this study.
*** Note - All links should resolve to a page where the data can be accessed. ***

7) Our journal encourages inclusion of *data citations in the reference list* to directly cite datasets that were re-used and obtained from public databases. Data citations in the article text are distinct from normal bibliographical citations and should directly link to the database records from which the data can be accessed. In the main text, data citations are formatted as follows: "Data ref: Smith et al, 2001" or "Data ref: NCBI Sequence Read Archive PRJNA342805, 2017". In the Reference list, data citations must be labeled with "[DATASET]". A data reference must provide the database name, accession number/identifiers and a resolvable link to the landing page from which the data can be accessed at the end of the reference. Further instructions are available at .

8) We would also encourage you to include the source data for figure panels that show essential data. Numerical data can be provided as individual .xls or .csv files (including a tab describing the data). For 'blots' or microscopy, uncropped images should be submitted (using a zip archive or a single pdf per main figure if multiple images need to be supplied for one panel). Additional information on source data and instruction on how to label the files are available at .

9) We replaced Supplementary Information with Expanded View (EV) Figures and Tables that are collapsible/expandable online (see examples in https://www.embopress.org/doi/10.15252/embj.201695874). A maximum of 5 EV Figures can be typeset. EV Figures should be cited as 'Figure EV1, Figure EV2" etc. in the text and their respective legends should be included in the main text after the legends of regular figures.

- For the figures that you do NOT wish to display as Expanded View figures, they should be bundled together with their legends in a single PDF file called *Appendix*, which should start with a short Table of Content. Appendix figures should be referred to in the main text as: "Appendix Figure S1, Appendix Figure S2" etc. See detailed instructions regarding expanded view here: .

- Additional Tables/Datasets should be labelled and referred to as Table EV1, Dataset EV1, etc. Legends have to be provided in a separate tab in case of .xls files. Alternatively, the legend can be supplied as a separate text file (README) and zipped together with the Table/Dataset file.

10) When assembling figures, please refer to our figure preparation guideline in order to ensure proper formatting and readability in print as well as on screen:
http://bit.ly/EMBOPressFigurePreparationGuideline

Please remember: Digital image enhancement is acceptable practice, as long as it accurately represents the original data and conforms to community standards. If a figure has been subjected to significant electronic manipulation, this must be noted in the figure legend or in the 'Materials and Methods' section. The editors reserve the right to request original versions of figures and the original images that were used to assemble the figure.

11) For data quantification: please specify the name of the statistical test used to generate error bars and P values, the number (n) of independent experiments (specify technical or biological replicates) underlying each data point and the test used to calculate p-values in each figure legend. The figure legends should contain a basic description of n, P and the test applied. Graphs must include a description of the bars and the error bars (s.d., s.e.m.).

Further information is available in our Guide For Authors: https://www.embopress.org/page/journal/14602075/authorguide

Revision to The EMBO Journal should be submitted online within 90 days, unless an extension has been requested and

approved by the editor; please click on the link below to submit the revision online before 30th Sep 2021:

https://emboj.msubmit.net/cgi-bin/main.plex

Referee #1:

This work constitutes a large, detailed and valuable resource (transcriptional, temporal and some spatial information on NC and SCP lineages). It has real depth as the authors characterize 7000 by SmartSeq. The bioinformatics is state of the art and the analysis is very well done. In general, the concept of characterizing transcriptional states of neural crest cells, Schwann cell precursors and their derivatives through developmental time is a useful one. One issue is that the paper is extremely dense and a rather difficult read. It will be a great resource but the authors are encouraged to discuss biological relevance in more detail. Specific suggestions are elaborated below.

Major Points:

1. While the work focuses primarily on transcriptional identity of cells over time, the authors rely a bit too heavily on pseudo-time analysis given that they have data on the developmental stages from which each cell comes.

2. Related to this, I also felt that spatial information for the cells (for which they had some data) was a little neglected, and only referred to towards the end of the manuscript.

3. The authors mention that "late non-sensory biased NCCs and early nerve associated SCPs converge on the same multipotent transcriptional state" - the 'hub state'. Please could the authors define the stage/origin of the 'late NCCs' and of the 'early SCPs' to which they refer.

4. Regarding the hub state, can the authors' eliminate the hub by knocking out key transcription factors? The prediction from their data would be that the sensory lineage is retained or that all cells should become sensory. This is a central claim of the title and wuld be straight-forward to test.

5. In Figure 1, panel E it would be useful to also have a figure where cells are color coded according to the specific stage at which they were collected (as in supplementary file 1, page 3, panel C) rather than just showing a general color scale.

6. Figure S1B - how did the authors define the delaminating (pink) and migrating (orange, yellow, purple) cells? Please include gene names used.

Minor points:

On page 14, in the paragraph starting "Both methods consistently extracted..", Fig 6 is referenced when I think the authors meant Fig 4.

Referee #2:

In this study, Kastriti, Fauvre et al. re-analyzed published single cell transcriptomics data to redefine the identity of genetically labelled Schwann cell precursors (SCPs) in mice and to investigate the extent of their multipotency. The authors propose that SCPs and neural crest cells (NCCs) form a "hub state", in which these two cell types are intermingled. The findings confirm a previous hypothesis mostly promoted by this group that SCPs basically represent a nerve-associated neural crest population with similar potentials. Moreover, analysis of transcriptional programs (metaregulons) revealed a striking similarity between early SCPs and late migratory NCCs, supporting their similarity in cell state. Furthermore, detailed analysis of the Schwann cell lineage tree pointed to spatial heterogeneity and new paths towards myelination. Lastly, the authors showed that malignant tumours, such as melanoma, contain tumour populations that map to SCP/NCC cell state, confirming previous studies.

This study addresses an intriguing concept, mainly introduced before by the Adameyko lab, that nerve-associated SCPs maintain NCC traits. It presents a very interesting model of how multiple derivatives are generated from NCCs/SCPs. However, more often than not it is difficult to understand how exactly the authors came to their conclusions. The proposed SCP hub state (in particular its association with nerves) has to be investigated in situ. Likewise, certain key statements need to be functionally validated. Finally, I feel that the part on tumor cells sharing an NCC/SCP signature is not novel as such and is only loosely connected to the rest of the paper.

Main points to be addressed:
1) The study is almost exclusively based on scRNA seq data analysis. Based on these data, the authors perform annotations, such as "SCPs" and "NCCs", and infer from this that what they call SCPs must be nerve-associated - hence, the main model of the paper proposes that nerve-associated cells maintain NCCs traits and serve as a pool of multipotent progenitors. However, it remains to be shown that the SCP/NCC "hub state" is indeed associated with nerves. Alternatively, late migratory NCC cells could already express "SCP" markers even before association with nerves. This possibility cannot be excluded given that at least regulatory elements for "glial" markers such as Plp and MPZ are already expressed in NCC before association with nerves (Hari et al., 2012; Kaucka et al., 2016), in line with transcription of "glial" genes already at the migratory NCC state. This is a central point of the paper and has to be experimentally addressed in situ.

2) Likewise, they often refer to early migratory NCCs and late migratory NCCs. Wnt1-Cre also labels premigratory NCCs. How do the authors know that what they call "NCCs" (blue in Fig.1 B) are not premigratory NCCs, while the hub cells are/comprise migratory NCCs?

3) Along these lines, the authors discuss at length about the resulting fates from the "hub" cells and also mention that it is a "common transcriptional state", but fail to actually describe exactly what this common state is. Could they elucidate how exactly is this "hub state" defined? What are the markers for this population? How is this different from the neighbouring populations?

4) The authors mention in the text: "When observing the root of the resulting tree of transcriptional events, we observed that one of the directions of differentiation..." in context of Fig. 1 - however, there is no tree or trajectory shown in the Figure. The overall trajectory has to be established.

5) Related to this, Figure 1 suggests that almost all derivatives are generated via SCPs. Do the authors interpret these data that none of the derivatives, including the entire population of enteric neurons/glia and sympathetic neurons, are directly generated from migratory NCCs? This contradicts previous literature and should be discussed. Again, the cluster labelled "SCPs" (i.e. nerve-associated cells) might just be wrongly annotated (see point 1) above).

6) Likewise, based on Fig.1 and Suppl. Fig. 1, melanocytes appear to be generated only from cranial neural crest, and neither from the SCP hub state nor from trunk NCCs. This also contradicts previous literature, including some of the authors' own studies, and should be clarified.

7) On p.7, the authors state that they don't focus on mesenchymal fate-biased cells (i.e. cranial NCCs (?)) and, yet, later in the manuscript (e.g. Fig. 3A) they appear to specifically include the cranial NCC population. This is confusing and should be clarified.

8) In Supplementary File A, (Page1, A) the authors show a "UMAP embedding of the QC-filtered dataset" and then highlight the clusters used for the rest of the study. However, the UMAP of the highlighted clusters does not seem to match the final UMAP embedding shown in Fig 1A and the rest of the paper. Could the authors clarify the relation between these different UMAPs?

9) Related to Figure 1, the authors state: "Joint UMAP embedding based on 1) multiscale diffusion space derived from PCA space (generated by gene-based pagoda2) and on 2) multiscale diffusion space derived from regulon activity scores (AUC, generated by SCENIC) showed striking similarities in their structure. This observation suggested that the main differentiation paths of the NC lineage could be abstracted to the level of transcription factor (TF) activities alone."
Could the authors indicate where (and if) they show these two UMAP embeddings in the paper and what is the exact similarity/overlap and differences if any?

10) The authors should compare the gene expression signatures that they found to published literature. For example, the gene expression signature of the endoneurial fibroblast population should be compared to the one shown in Carr et al, 2019. Furthermore, they also do not comment on the discrepancies between their SCP and iSCs signatures and the ones proposed a while ago by the Jessen and Mirsky lab. Moreover, the markers that they used to delineate the adult mSCs and nmSCs are not very common in the field, so they should comment on this.

11) Likewise, the metaregulons presented in Fig. 2 need some discussions in view of current knowledge in the field. For instance, how to explain the presence of Foxd3 or Jun (p. 11) in metaregulon 6? Both genes have been shown before to be associated with early steps in NC/SCPO development. In addition, Jun is a negative regulator of myelination (Parkinson et al. 2008) by inhibiting Krox20-induced myelin protein expression (Mpz), so it is surprising to find it in the same group as e.g. the pro-myelinating factor Pou3f1 (Oct6). Is there any evidence for their statement (p.11) that "the role of FoxD3 changes from transcriptional repression ....to transcriptional activation..."?

12) Do the regulons shown in Fig. 2(B) correspond to the entire set of regulons resulting from the analysis? If no, could the authors provide the complete set? And do the genes mentioned in each regulon correspond to the entire list for that particular regulon? Is the principal tree/pseudotime trajectory mentioned here the same as referred to in my point 4 above?

13) On p.14 and elsewhere (related to Fig. 4), the authors suggest a new path to myelination. How do they explain in this

context (absence of previously dubbed immature SCs) the process of radial sorting? Are these Pou3f1-expressing (OCT6+) immature pro-myelinating SCs associated with large caliber axons? If Oct6 is ablated in this population does this, in addition to loss of mSCs, result into loss of the endoneurial fibroblast population, too? Is it known whether Oct6 binds to any transcriptional locus that drives fibroblast gene expression? How would myelinating (!) SCs be able to generate endoneurial fibroblasts without de-differentiation? In sum, there are several novel (and surprising) statements regarding the SC lineage tree that should be, at least to a certain extent, functionally validated. Otherwise, one starts to doubt the validity of the conclusions drawn from the (after all "descriptive") transcriptomics data.
This is also relevant because the authors' only approach for functional validation in this context actually failed: they validated the functionality of Nr4a2 that they have introduced as a novel gene in terminal glia specification or myelination. However, upon loss of this nuclear receptor, mSCs (and nmSCS) are still formed normally during development.

14) The authors propose that there is heterogeneity in SCPs/SCs associated with a specific spatial and temporal code. However, the authors completely neglect the presence of satellite glia in different ganglia (sensory, different types of autonomic) that could explain their findings. The statement made on p.7 that starting from E11.5 all Sox10+ cells are associated with peripheral nerves is wrong as satellite glia that are known to express lineage/location-specific markers also express Sox10 and many other glial markers. This issue needs to be addressed.

15) In the last chapter of the Results section, the authors say that malignant cells re-enact developmental gene expression. This is not novel; the findings presented in Figures 6, 7 are rather descriptive (the transcriptome of some NCCs/SCPs/SCs maps to some tumor cells) and do not add much to what is already known in the field. In their present form, these data are preliminary (because they don't give novel insights, for instance, into how NCC/SCP signatures contribute to tumorigenesis, etc.), the link to the rest of the paper is poorly established. I suggest to delete these figures.

16) The legend of Fig S1 D reads "D) Dot plot showing top differentially expressed genes (left) and regulon AUC scores (right) between the two NCC selections made in (C)". However, in the figure there is no dot plot or regulon AUC scores in D, but what seems to be a feature plot differentiating between cranial and neural stem cells.

17) With respect to Figure 3, the authors mention that "a version of the NC and SC developmental tree as a system of interconnected "nodes"..." - Is this a new tree fitting or merely a subset of the tree referred to earlier?

18) In the discussion, the authors state that Dhh is expressed in the progenitors of endoneurial fibroblasts that are stated in this paper as Pou3f1-expressing (OCT6+) immature pro-myelinating SCs. However, in Figure 4J, high Dhh gene expression does not appear to be present in this population, how can this be explained?

Minor points:
Because of the missing figure labeling and inconsistent figure formatting, it was somewhat tedious to evaluate the data presented in the figures.

On page 2 (summary) there is a word missing in this sentence: "Our results revealed that early SCPs and late migratory crest show an indistinguishable...

On page 3, wrong references (Le Douarin, Westin) are given regarding the statement that SCPs give rise to multiple cell types.

On page 6, the stage(s) at which Plp-CreERT2 and Sox10-CreERT2 were induced should be indicated.

In Fig 2(B): What is meant by 'Density', what are the values defining the different fields ranging from yellow to purple?

In Fig 2(D): What are the p values associated with the correlation?
In Fig 2 (G): Heatmap legend is missing.

In Figure 3 and Suppl. Fig. 4, what is the evidence for convergence between a NCC and a hub state, i.e. why is there a bi-directional arrow between the two states?

Fig. 4(E) is referred to in the text before 4(A) and others.

Fig 6(A): "We extracted single cells forming a trajectory from a single patient and applied..." - why was only a single a patient chosen for this? The exact message of the middle panel for Fig 6(A) is not clear as there are significant differences between what the figure legend says and what is mentioned in the text.

The legend to Figure 7(A) says "uveal melanoma" - it should be "neuroblastoma".

On page 9, the authors describe Ednrb, Heyl and Fabb7 as glial markers. This should be supported by references. Furthermore, the authors say that the Sp7 regulon is absent upon NCC differentiation into SCPs. In both figures, 2B and S3, Sp7 is not depicted, though; this should be corrected or clarified. Moreover, metaregulon 4 contains Ets1 and Tfap2a regulons that are

described as essential for NC gene expression program. References for this statement are missing.

On page 11, the last sentence before the new subchapter should be written more clearly. What is meant by locked between the activity of metaregulon 1,2, and 6? Also including 3?

On page 37 (supplementary figure legends), the authors should include the references for the published markers they have used.

In Figure 5, B should be in bold. Importantly, in B and D, the labeling for 'sensory nerve' and 'visceral nerve' should be reconsidered; the labelled structures appear to be cartilage, not nerves.

On page 27, in Figure legend 6B, the authors write that the dot plot shows the top 8 gene markers, however there are actually 10 displayed, this should be corrected.

In Figure 7, why do the dot plots show bipotent sympathoadrenal progenitors in A and in B SCs? Should not both contain both of these populations to illustrate that in A the cells are more similar to progenitors, whereas in B to differentiated cells?

Referee #4:

Eleni et al use single-cell transcriptomics to address the question of Schwann cell precursor (SCP) potency and identity. The authors build their conclusions on RNA velocity, regulon (co-expression), cell embedding and FISH analysis taking advantage of new and publicly available data sets.

Overall they performed a detailed and thorough analysis that will be interesting for many in the field. Nevertheless, addressing some points might further improve the manuscript and will help to better grasp the main points of the study. This mainly refers to the hub state which is the main finding of the paper.

Major points

The calculation of the regulons is only based on one portion of the NCCs. It is stated in the manuscript that this was done intentionally as the other NCC state was already analyzed elsewhere. However, it should be still included in the analysis maybe showing that these cells have a similar programme or a constrasting one. This can be a supplementary figure but the reasoning to exclude should come from the data and needs to be justified. Overall it seems that the hub cells orginiate from both mesenchymal and non-mesenchymal NCCs.

In Figure 3A it is suggested that there might be a fluidic fate split with two distinct hub cell types stemming from mesenchymal and non-mesenchymal NCCs. Would this mean that any cell type can originate from any NCC and that there is fluidity in the hub state? Can these states be defined via the regulon analysis (see first point)? Can the authors comment on other possible scenarios?

The data presented here stem from two different collections - previously prepared cells up to E11.5 and cells starting from E12.5. Could this explain the unexpected behaviour in Figure 2B with oscillations and a peak in E12.5 and later samples? Can the authors exclude a technical bias due to the data collection differences?

The authors have used data from human tumour samples and integrated / projected the cells on the mouse data. Can the authors comment on how they extracted the malignant cells from the data sets? Could they also include non-malignant cells to show the robustness of the embedding? Have the authors corrected for copy number variations in the tumours?

Minor points

Due to the detailed view on the data set it is not easy to follow which cell type would correspond to which cluster in the central figure F1B. The naming is not consistent throughout the manuscript and should be clarified. One example (among many) relates to Figure 2A - here the SCPs relate to the hub/early SCPS while the previously assigned SCPs are called iSCs.

Additionally, the neural crest population is given twice in Figure 1B and split up in the supplements. It would be better to state at least one discriminating gene in the list in the figure.

Figure 2B: What are the yellow and lilac curves? Developmental time? Needs a label.

Typo in abstract - there is at least one word missing ("show an indistinguishable" XXX).

On page 14 the wrong Figure is cited. (Fig 6A, I is given should be Figure 4?)

Figure 5B and 5C are not referenced in the text.

Response to Reviewers

Referee #1:

This work constitutes a large, detailed and valuable resource (transcriptional, temporal and some spatial information on NC and SCP lineages). It has real depth as the authors characterize 7000 by SmartSeq. The bioinformatics is state of the art and the analysis is very well done. In general, the concept of characterizing transcriptional states of neural crest cells, Schwann cell precursors and their derivatives through developmental time is a useful one. One issue is that the paper is extremely dense and a rather difficult read. It will be a great resource but the authors are encouraged to discuss biological relevance in more detail. Specific suggestions are elaborated below.

We want to thank the reviewer for seeing the importance of this work for the field. In the current version, we expanded the cell numbers to better cover minor populations in the transcriptomics dataset (now 8842 cells versus 6977 before), repeated thorough bioinformatics, performed more validations, added functional experiments and rewrote the text to improve clarity. We specifically focused on relating our observations to biological implications and expanding our analysis to include more aspects of Schwann cell heterogeneity, while still bridging it with pre-existing knowledge in the field.

All changes in the revised manuscript are coloured in yellow.

The single cell atlas is available for exploration here:

https://adameykolab.srv.meduniwien.ac.at/glia_gene_umap/ with the (gene-based embedding)

https://adameykolab.srv.meduniwien.ac.at/glia_scenic_umap/ with (SCENIC-based UMAP embedding).

Major Points:

1. While the work focuses primarily on transcriptional identity of cells over time, the authors rely a bit too heavily on pseudo-time analysis given that they have data on the developmental stages from which each cell comes.

- We thank the reviewer for this comment and we agree that developmental time should be used and mentioned more often. In the revised manuscript, to improve data representation and bridge the pseudotime analysis with known developmental stages (as this information is always in place for every individual cell), we devised the updated Figure EV1. Additionally, we show the flow of

developmental stages on the main UMAP in Fig 1D, as well as an overlay of regulon analysis with developmental time in Fig 5B, which enables better correlation of coordinated transcription factor activity and developmental stage. We did not find conflicts between our pseudotime analysis and prior knowledge of developmental stages. This is very important, because pseudotime analysis is performed computationally in an unbiased way, and any contradictions would have to be curated and corrected manually, which would suggest bad quality of data or insufficient sampling. The coherence of pseudotime and actual stages supports good quality of the data and the analysis.

We put emphasis on the developmental time when breaking down specific aspects of the data set in relation to regulon analysis, to ensure that the reader can always relate the developmental stage to the relevant observations:

- *Lines 294-297: "We then tested and fitted significant AUC scores to the inferred differentiation trajectory and superimposed the biological developmental time transferred from the individual cell transcriptomes (Fig 5B)."*
- *Lines 305-308: "Of these, two metaregulons were characterized by high activity during the early migratory NC-stage (E9.5 and to a lesser degree E10.5) that subsequently decreased as the trajectory proceeds towards the "hub"/SCP state and further to SCs (metaregulons 1 and 3)."*
- *Lines 321-325: "Concomitantly, we detected smooth and incrementally increasing differences in overall gene expression starting from the NC towards SCPs at the transition between late E10.5 to E11.5 and E12.5, including the onset of activity of Jun, Sox10, Nfatc1/2 and Pou3f1, all genes with described roles in myelination (metaregulon 2) (Fig 5C) (Mirsky, Woodhoo et al. 2008)."*
- *Lines 336-338: "The active regulons belonging to metaregulon 4 corresponded to neurogenic sensory bias activated in NC and early "hub" cells at E9.5-E10.5 and included Six1 and Neurog1 regulons (FigEV2) (Sommer, Ma et al. 1996, Zou, Silvius et al. 2004)."*
- *Lines 364-366: "Accordingly, members of metaregulon 5 were reactivated in the rest of the "hub" and SCPs before commitment to terminal fates in cells from E11.5-E14.5."*

2. Related to this, I also felt that spatial information for the cells (for which they had some data) was a little neglected, and only referred to towards the end of the manuscript.

- We understand this reasoning and agree. Although the major focus of the paper is a concept of Schwann cell precursors playing a role as a ""hub"", we enriched the spatial heterogeneity aspect in the revised manuscript (according to the reviewer's advice). Please see the description of improvements we made to the analysis of spatial heterogeneity in the revised manuscript:

  a. Uncovering the unique transcriptome of developing terminal Schwann cells positioned at the neuromuscular junctions, along with important validations of specific markers that are in agreement with recently published

data from the adult neuromuscular junction (Castro, Taetzsch et al. 2020) (Fig 7, Fig EV4). Relevant part of the text:

Lines 516-522: ".Differentially expressed markers suggested an early splitting cell type derived from iSCs, which corresponded to terminal SCs of neuromuscular junction SCs (tSCs) (Fig 8C,E, Fig EV4), based on previously reported markers such as Cspg4, Itga8, Slitrk3, Cpm, Pou3f1 (Castro, Taetzsch et al. 2020). Biological validations showed the early origin of Itga8+/Cpm+/Pou3f1+ terminal SCs as seen by RNAscope® or immunofluoresnce against OCT6 (encoded by Pou3f1) on E18.5 Plp1YFP-traced embryos when recombination was induced at E13.5 (Fig 8F, Fig EV4F)."

Lines 588-593: "Next, OCT6^{high} cells (encoded by Pou3f1) were predominantly found in developing and postnatal limbs, with an increasing gradient of OCT6 levels from proximal to distal part of the peripheral nerves (Fig 8E). These cells where characterized by the signature of terminal neuromuscular SCs as seen by the expression of Cpm, Itga8, Slitrk3, Cspg4 and other markers previously shown enriched in this SC subpopulation (Fig EV4A,B,D, Appendix FigS13)."

- b. Analysis of "hub" marker genes (*Itga4*, *Serpine2*, *Sox8*) in discrete anatomical locations which revealed that they are found in all analysed locations (in dorsal root ganglia, along mixed peripheral nerves, autonomic nerves and ganglia, enteric locations) (Appendix FigS5-7)

  Lines 185-189: "The "hub" cells expressed both pan-neural crest as well as SCP markers (Fig 2D,E) and spanned all peripheral locations, including mixed nerves and developing autonomic and enteric ganglia as validated experimentally with combined SOX10 immunodetection and RNAscope® for the "hub" genes Itga4, Serpine2 and Sox8 (Appendix Figures S5,6,7)."

- c. Identification of two subtypes of peripheral glia with special interest in terms of their location: satellite glia from the dorsal root ganglia using markers from a recently published data by and boundary cap cells (Fig 1B, Appendix FigS3, S4)

Lines 244-251: *"Finally, we identified and connected the satellite glial cells found in association with sensory neurons within dorsal root ganglia, and boundary cap stem cells (BCCs) associated with motor exit points from the neural tube. Using previously known markers (Coulpier, Le Crom et al. 2009, Mapps, Thomsen et al. 2022), we found the satellite glia and BCCs in position where the neural crest populations transit into the "hub" (Fig 1B, Appendix FigS4). The expression of "hub" markers in these cell populations suggests that they represent highly specialized subtypes of multipotent neuron- and nerve-associated cells."*

3. The authors mention that "late non-sensory biased NCCs and early nerve associated SCPs converge on the same multipotent transcriptional state" - the '"hub"

state'. Please could the authors define the stage/origin of the 'late NCCs' and of the 'early SCPs' to which they refer.

- In the pseudotime embedding, the delaminating neural crest cells (derived from E9.5 embryos) transit into early migratory neural crest, which splits into sensory-biased and (late) post-sensory. Post-sensory NCCs in turn eventually transit into early SCPs, which correspond to the ““hub”” state in our manuscript (we validate these dynamics using the “hub” specific markers *Itga4*, *Serpine2* and *Sox8*, see new Fig 2C and Appendix FigS5-7, consistent with Soldatov et al., Science 2019) (Soldatov, Kaucka et al. 2019). This smooth transition is reflected in our UMAPs (revised Figure 1), and the border of this transition is not sharp, showing a mix of cells from E9.5, E10.5 and even some E11.5 cells. From the relevant developmental stages, we know that cells from E9.5 represent migratory and very few nerve-associating neural crest cells, E10.5 cells are a mix of free-migrating and nerve-associated cells and E11.5 are nerve-associated Schwann cell precursors. We previously described this mixing at the transition from NCCs to SCPs as “converging multipotent state”. Additionally, using SOX10 as a pan-neural crest/Schwann cell precursor marker, we have previously shown the onset of nerve association of SOX10+ cells at E10.5, and at this stage some late neural crest cells (freely migrating) co-exist with early Schwann cell precursors (already nerve-associated).
  In order to better clarify the situation in the revised manuscript, we generated new panels and figures explaining how the ““hub”” is defined and how the ““hub”” and the neural crest cells (delaminating, migrating etc.) relate to each other in terms of transitions and similarities (please see updated Figures 2 and 3, Figure EV4).

  To clarify this, we also replaced the phrase "late non-sensory biased NCCs and early nerve associated SCPs converge on the same multipotent transcriptional state" - the '“hub” state' by the following:

  Lines 195-199: “Thus, the late non-sensory-biased NCCs and nerve-associated SCPs (at E9.5, E10.5 and E11.5) from cranial and trunk regions converge on a similar multipotent transcriptional state we refer to as “hub”, from which other sub-trajectories emerge towards multiple definitive fates according to RNA velocity and trajectory analysis (Fig 1B, C, F).”

4. Regarding the “hub” state, can the authors' eliminate the “hub” by knocking out key transcription factors? The prediction from their data would be that the sensory lineage is retained or that all cells should become sensory. This is a central claim of the title and wuld be straight-forward to test.

- We are grateful for this suggestion and we must admit that this was the hardest part of the revision. To address this point, we identified several key genes enriched in the “hub”, including *Sox8* (Fig2). We had no chance to produce conditional knock out mice during the time of the revision and run a full analysis on them, so we attempted to knock down *Sox8* in chick embryos via electroporation of Cas9/CRISPR guides into the neural crest domain (Fig

4). As predicted, knockout and downregulation of SOX8 resulted in selective depletion of Schwann cell precursors leaving sensory neurons unaffected. This depletion was not due to decreased proliferative activity. Furthermore, we observed a shift in proportions between SCPs and forming TH+ chromaffin cells, which suggested higher conversion rates of SCPs into TH+ fate in the case of SOX8 downregulation. Thus, *Sox8* preserved the SCP/"hub" state balancing possible recruitments into other fates. Please see new Figure 4 for checking these functional data and see the relevant text in the revised manuscript in the section *"Loss of "hub" gene Sox8 results in SCPs defects and biased cell differentiation"*:

- *Lines 255-275: "To test the function of the "hub"-specific transcription factor Sox8, we electroporated the trunk neural tube of HH11 chick embryos with a single plasmid containing gRNA, Cas9 and a citrine reporter (Gandhi, Li et al. 2021) to knock-out Sox8 in neural crest-derived cells (Fig 4, A-B). Embryos were then allowed to develop until HH24-25 (Fig 4, C), by which time the dorsal root ganglia (DRG) and visceral nerves had developed (Fig 4H). Quantification revealed a markedly reduced proportion of electroporated CITRINE+/SOX10+ cells along the ventral nerve after loss of Sox8 as compared to controls (Fig 4 D-E and G). No significant differences were noted in numbers of cells undergoing mitosis between experimental and control embryos, suggesting that Sox8 might be involved in regulating migration or other processes rather than proliferation of neural crest-derived cells along the ventral nerve. This is consistent with a previous study suggesting a role for Sox8 in neural crest migration (O'Donnell, Hong et al. 2006). Despite the phenotype in the ventral nerves, the loss of Sox8 did not alter the number of glial (SOX10+) or neuronal (ISL+) cells in the DRGs (Fig 4 D-F), which supports that loss of SOX8 does not affect cell survival. By contrast, in the sympathoadrenal domain, the proportion of CITRINE+/SOX10+ nerve-associated cells appeared reduced, whereas the proportion of CITRINE+/TH+ cells significantly increased (Fig 4 I-J). This result suggests elevated rates of conversion of sympatho-adrenal SCPs into adrenergic fates. Taken together, high levels of Sox8 might specifically stabilise the SCP/"hub" phenotype and assist the migration of neural crest-derived SOX10+ cells along the peripheral nerves."*

5. In Figure 1, panel E it would be useful to also have a figure where cells are color coded according to the specific stage at which they were collected (as in supplementary file 1, page 3, panel C) rather than just showing a general color scale.

- This is done now, and the colour coding according to developmental stages is shown in revised Figure 1D. A more detailed outline of stages contributing to the embedding is shown in the revised Figure EV1.

6. Figure S1B - how did the authors define the delaminating (pink) and migrating (orange, yellow, purple) cells? Please include gene names used.

- The definitions of the neural crest stages in the current mouse Smartseq2 single cell data was taken directly from our previous paper published in

Science in 2019 (Soldatov, Kaucka et al. 2019), where these stages were reliably characterized based on combination of Smartseq2 single cell transcriptomics and spatial transcriptomics. Since our dataset contained the exact same published cells from that time and location, we could directly colour them by the already characterized clustering from that study. We hope the reviewer will not insist on repeating experiments and definitions we already published recently. In brief, delaminating cells are defined by Dlx5, Pak3, Sox9, Sox10 genes, and migrating groups are characterized by *Sfrp5*, *Heyl* and *Nkain4* expression.

In the revised Fig EV3, panel A, we incorporated a more complete gene list derived from our previous work.

We have introduced this explanation in the manuscript as follows:
Lines 425-433: "With the help pf our "hub"-specific gene expression signature (Sox8, Itga4 etc.), we mapped the "hub"-state onto the neural crest Soldatov et al. dataset to detect the emergence of the early "hub" program (Fig EV3). For this, we relied on the described states of neural crest development including delaminating, multipotent migrating progenitors, autonomic-biased cells and cells differentiating towards neurons (Fig EV3A). Briefly, delaminating neural crest cells are defined by Dlx5, Pak3, Sox9, Sox10 genes, and migrating groups are characterized by Sfrp5, Heyl and onset of Nkain4 expression, while sensory neurons expressed Neurog1/2, Pou4f1, Isl1, Six1 and Neurod1/4."

Minor points:

On page 14, in the paragraph starting "Both methods consistently extracted..", Fig 6 is referenced when I think the authors meant Fig 4.

- Thanks so much for spotting this! Now we updated all figures, and we hope that the referencing of all panels is correct.

Referee #2:

In this study, Kastriti, Faure et al. re-analyzed published single cell transcriptomics data to redefine the identity of genetically labelled Schwann cell precursors (SCPs) in mice and to investigate the extent of their multipotency.

- We thank the referee for in depth analysis of our manuscript and would like to clarify that the majority of cells in this study was sequenced *de novo* in addition to previously published data. During this revision, we added many more freshly sequenced cells from different developmental time points to clarify the reviewers's questions.

  The single cell atlas is available for exploration here:

https://adameykolab.srv.meduniwien.ac.at/glia_gene_umap/ with the (gene-based embedding)

https://adameykolab.srv.meduniwien.ac.at/glia_scenic_umap/ with (SCENIC-based UMAP embedding).

The authors propose that SCPs and neural crest cells (NCCs) form a "“hub” state", in which these two cell types are intermingled. The findings confirm a previous hypothesis mostly promoted by this group that SCPs basically represent a nerve-associated neural crest population with similar potentials. Moreover, analysis of transcriptional programs (metaregulons) revealed a striking similarity between early SCPs and late migratory NCCs, supporting their similarity in cell state. Furthermore, detailed analysis of the Schwann cell lineage tree pointed to spatial heterogeneity and new paths towards myelination. Lastly, the authors showed that malignant tumours, such as melanoma, contain tumour populations that map to SCP/NCC cell state, confirming previous studies.

This study addresses an intriguing concept, mainly introduced before by the Adameyko lab, that nerve-associated SCPs maintain NCC traits. It presents a very interesting model of how multiple derivatives are generated from NCCs/SCPs. However, more often than not it is difficult to understand how exactly the authors came to their conclusions. The proposed SCP “hub” state (in particular its association with nerves) has to be investigated in situ.

- We agree with the reviewer, and we followed the advice above. In the previous published works (Adameyko et al., Development 2012; Dyachuk et al., Science 2014) we already analysed the timeline of nerve association extensively, which mainly takes place in mouse embryos around E10. In this study, we correlated it with the onset of ““hub”” marker expression. ““hub””

markers showed a gradual increase according to bioinformatics predictions and new experimental validations predominantly after the nerve association, which is known to take place between E9.5 and E10.5. Please see new Appendix FigS5-7 and note that the peak of ""hub"" marker expression is detected at E12.5 (revised Fig 2C).

In our UMAPs, nerve-associated *Sox10+* cells from E10.5 co-clustered with some migratory neural crest from E9.5. Upon observing the relevant ""hub" score" in cells from E9.5, we find that ""hub"" genes start being detectable in previously identified multipotent migratory NC progenitors and autonomic fate-biased NCCs, characterized by the expression *Sox10*, *Foxd3* and *Phox2b* (see new results in Fig EV1B and Fig EV3C). This is suggesting that the onset of the ""hub"" state is triggered prior to nerve-association (in free migrating neural crest) but reaches a peak gradually already after the nerve association is complete according to experimental (new Appendix FigS5-7) and computational evidence (Fig EV3). We also performed a functional experiment by knocking out ""hub"" marker *Sox8 in vivo* in ventral nerves. The result showed a specific depletion of nerve-associated cells correlated with the change of the proportions in nerve-associated and sympatho-adrenal cells, suggesting that high levels of *Sox8* prevent the recruitment of SCPs into other fates (see new Figure 4).

Likewise, certain key statements need to be functionally validated.

- As we just mentioned above, we could specifically target the ""hub"" state in the nerves via downregulating *Sox8* gene (Figure 4). Please see the relevant text in the revised manuscript in the section *"Loss of "hub" gene Sox8 results in SCPs defects and biased cell differentiation"*:

- *Lines 255-275: "To test the function of the "hub"-specific transcription factor Sox8, we electroporated the trunk neural tube of HH11 chick embryos with a single plasmid containing gRNA, Cas9 and a citrine reporter (Gandhi, Li et al. 2021) to knock-out Sox8 in neural crest-derived cells (Fig 4, A-B). Embryos were then allowed to develop until HH24-25 (Fig 4, C), by which time the dorsal root ganglia (DRG) and visceral nerves had developed (Fig 4H). Quantification revealed a markedly reduced proportion of electroporated CITRINE+/SOX10+ cells along the ventral nerve after loss of Sox8 as compared to controls (Fig 4 D-E and G). No significant differences were noted in numbers of cells undergoing mitosis between experimental and control embryos, suggesting that Sox8 might be involved in regulating migration or other processes rather than proliferation of neural crest-derived cells along the ventral nerve. This is consistent with a previous study suggesting a role for Sox8 in neural crest migration (O'Donnell, Hong et al. 2006). Despite the phenotype in the ventral nerves, the loss of Sox8 did not alter the number of glial (SOX10+) or neuronal (ISL+) cells in the DRGs (Fig 4 D-F), which supports that loss of SOX8 does not affect cell survival. By contrast, in the sympathoadrenal domain, the proportion of CITRINE+/SOX10+ nerve-*

associated cells appeared reduced, whereas the proportion of CITRINE+/TH+ cells significantly increased (Fig 4 I-J). This result suggests elevated rates of conversion of sympatho-adrenal SCPs into adrenergic fates. Taken together, high levels of Sox8 might specifically stabilise the SCP/"hub" phenotype and assist the migration of neural crest-derived SOX10+ cells along the peripheral nerves."

-

Finally, I feel that the part on tumor cells sharing an NCC/SCP signature is not novel as such and is only loosely connected to the rest of the paper.

- We agree in principle, although the Editor encouraged us to keep these data in the manuscript as a methodological example. We moved them to the Appendix (FigS14, S15) and reduced the discussion of these data as compared to the original version of the manuscript. We also would like to note that the uncovered temporal and spatial heterogeneity of SCPs brings the comparisons between neural crest lineage and tumour cells to a new level of detail.

Main points to be addressed:

1) The study is almost exclusively based on scRNA seq data analysis. Based on these data, the authors perform annotations, such as "SCPs" and "NCCs", and infer from this that what they call SCPs must be nerve-associated - hence, the main model of the paper proposes that nerve-associated cells maintain NCCs traits and serve as a pool of multipotent progenitors. However, it remains to be shown that the SCP/NCC "“hub” state" is indeed associated with nerves.

- From E10.5 and onwards, the vast majority of SOX10+ cells in the dataset are nerve-associated (or associated with peripheral neurons in the case of satellite glia), because the migration of the neural crest is completed, as shown by previous studies (please see Adameyko et al., Development 2012 and Dyachuk et al., Science 2014 for whole mount-based validations we did at that time and further data in Furlan et al., Science 2017). To address the timing of ““hub”” cell markers, we experimentally measured the expression of *Itga4* and *Sox8* in *Sox10+* cells between E9.5 and E11.5. The results revealed that the increase and consolidation of these ““hub”” state markers occurred after E10.5 (Appendix FigS5-7, Fig EV§), i.e. following nerve-association stage and, most likely, being supported by interactions with nerve fibers. Therefore, the ““hub”” state is predominantly represented by the nerve-associated SCPs. At the same time, some migratory neural crest cells started showing weaker expression of ““hub”” state markers prior to massive nerve-association events. In that case, the level of ““hub”” marker expression is comparatively low, which supports that the ““hub”” program becomes consolidated much later within the nerves.

  We added the following lines to the Results and Discussion:

Lines 199-204: "The dynamics of nerve association combined with the onset of detectable "hub" signature (Itga4, Serpine2, Sox8) by RNAscope® in neural crest cells at post-delamination stages at E9.5 (Appendix FigS5-7) and progressive upregulation with an observed peak at E12.5 (Fig 3B) proposes that the onset of the "hub" state initiates in late neural crest but consolidates and reaches a peak upon cell attachment onto peripheral nerves."
Lines 435-441: "Some cells previously considered as multipotent migrating progenitors in Soldatov et al. dataset appeared more similar to the "hub" state as compared to others with the highest "hub" signature identified in the neural crest cells biased to the autonomic fate before neuro-glial separation (Fig EV3A, C, D). This observation reinforces the idea that the emerging "hub" state is seen at late neural crest stages, prior to nerve-association, but is reinforced and upregulated following the attachment of cells to the peripheral nerves."

Alternatively, late migratory NCC cells could already express "SCP" markers even before association with nerves. This possibility cannot be excluded given that at least regulatory elements for "glial" markers such as Plp and MPZ are already expressed in NCC before association with nerves (Hari et al., 2012; Kaucka et al., 2016), in line with transcription of "glial" genes already at the migratory NCC state. This is a central point of the paper and has to be experimentally addressed in situ.

- We addressed this possibility by detecting and quantifying the "hub"-enriched markers revealed by our analysis (*Itga4*, *Serpine2* and *Sox8*) at different locations and stages (Appendix FigS5-7). We found that the "hub"-specific signature is almost non-detectable during delamination and early neural crest migrationand switches on in migrating and autonomic fate-biased NCCs at E9.5, becoming further upregulated at E10.5 and reaching its peak of expression in SOX10+ cells at E11.5 (Fig 2C, Fig EV3). Although some late neural crest cells show the expression of "hub" markers and generally smoothly transit into the "hub" following the established time of nerve-association (Fig EV3, Appendix FigS5-7), our experiments using RNAscope® in situ hybridization show that the "hub" signature becomes strong and prominent in SOX10+ cells after E11.5 (please see revised Figure 2C and Figure 3B). Still, it is important to understand that this "hub" signature is gradually upregulated starting from the late migratory neural crest and is not switched on instantly. Thus, the peak of the ""hub"" combinatorial signature is observed at E11.5-E12.5 stages. This suggests that the "hub" signature is not necessarily triggered by nerve-association, but rather becomes supported by the nerve-association developing to a full extent in nerve-associated cells.

  All these aspects are mentioned in the revised manuscript:
  Lines 184-195: "The "hub" cells (represented as grey in Fig 1B) mainly originated from E10.5, E11.5 and E12.5 stages (Fig 2C, Fig EV1B) with few admixed E9.5 NCCs. The "hub" cells expressed both pan-neural crest as well as SCP markers (Fig 2D,E) and spanned all peripheral locations, including mixed nerves and developing autonomic and enteric ganglia as validated

experimentally with combined SOX10 immunodetection and RNAscope® for the "hub" genes Itga4, Serpine2 and Sox8 (Appendix Figures S5,6,7). Even though SOX10+ cells representing neural crest migrate freely at E9.5, very few of them are found near the emerging axons. At E10.5, most of SOX10+ cells become nerve (or peripheral neuron)-associated. Starting from E11.5, all SOX10+ cells are associated with the outgrowing peripheral nerves or neurons in consistency with previous studies (Adameyko, Lallemend et al. 2012, Dyachuk, Furlan et al. 2014, Furlan, Dyachuk et al. 2017)."

Lines 425-430: "With the help pf our "hub"-specific gene expression signature (Sox8, Itga4 etc.), we mapped the "hub"-state onto the neural crest Soldatov et al. dataset to detect the emergence of the early "hub" program (Fig EV3). For this, we relied on the described states of neural crest development including delaminating, multipotent migrating progenitors, autonomic-biased cells and cells differentiating towards neurons (Fig EV3A)."

Lines 433-438: "Jointed analysis of the genes expressed by the neural crest and "hub" cells uncovered different degrees of similarity in gene expression (Fig EV3B, Table 1). Some cells previously considered as multipotent migrating progenitors in Soldatov et al. dataset appeared more similar to the "hub" state as compared to others with the highest "hub" signature identified in the neural crest cells biased to the autonomic fate before neuro-glial separation (Fig EV3A, C, D)."

2) Likewise, they often refer to early migratory NCCs and late migratory NCCs. Wnt1-Cre also labels premigratory NCCs. How do the authors know that what they call "NCCs" (blue in Fig.1 B) are not premigratory NCCs, while the "hub" cells are/comprise migratory NCCs?

- The definitions of the neural crest stages in the current mouse Smartseq2 single cell data were taken directly from our previous paper published in Science in 2019 (Soldatov, Kaucka et al. 2019), where these stages were reliably characterized based on combination of Smartseq2 single cell transcriptomics and spatial transcriptomics. Since our dataset contained the exact same published cells from that time and location, we could directly colour them by the already characterized clustering from that study. We hope the reviewer will not insist on repeating experiments and definitions we already published recently. In brief, delaminating cells are defined by Dlx5, Pak3, Sox9, Sox10 genes, and migrating groups are characterized by *Sfrp5*, *Heyl* and *Nkain4* expression. Since we previously characterized extensively the signature of the premigratory neural crest (Soldatov, Kaucka et al. 2019) by the expression of classical neural tube markers *Olig3*, *Sox2* and *Msx1*, we could specifically omit this population from our subselected data set (please see Appendix FigS1B).
  We have introduced this explanation in the manuscript as follows:

Lines 143-147: “After computational clean-up of contaminating populations (including predelaminating neural crest based on neural tube signature defined as Olig3+/Sox2+/Msx1+ cells) and low-quality transcriptomes (Appendix Figure S1, S2), we recovered the transcriptomes of 8842 cells covering embryonic and postnatal stages, different locations and neural crest-derived fates (Fig EV1A,B).”

Lines 425-433: With the help pf our “hub”-specific gene expression signature (Sox8, Itga4 etc.), we mapped the “hub”-state onto the neural crest Soldatov et al. dataset to detect the emergence of the early “hub” program (Fig EV3). For this, we relied on the described states of neural crest development including delaminating, multipotent migrating progenitors, autonomic-biased cells and cells differentiating towards neurons (Fig EV3A). Briefly, delaminating neural crest cells are defined by Dlx5, Pak3, Sox9, Sox10 genes, and migrating groups are characterized by Sfrp5, Heyl and onset of Nkain4 expression, while sensory neurons expressed Neurog1/2, Pou4f1, Isl1, Six1 and Neurod1/4.”

3) Along these lines, the authors discuss at length about the resulting fates from the "“hub”" cells and also mention that it is a "common transcriptional state", but fail to actually describe exactly what this common state is. Could they elucidate how exactly is this "“hub” state" defined? What are the markers for this population? How is this different from the neighbouring populations?

- We thank the reviewer for this comment. In the revised manuscript, we provide clear explanations for how we define the “hub” state and list specific markers enriched in the “hub” (also validated experimentally):

  Lines 185-189: The “hub” cells expressed both pan-neural crest as well as SCP markers (Fig 2D,E) and spanned all peripheral locations, including mixed nerves and developing autonomic and enteric ganglia as validated experimentally with combined SOX10 immunodetection and RNAscope® for the “hub” genes Itga4, Serpine2 and Sox8 (Appendix Figures S5,6,7).”

  Lines 1032-1039: “Cell-type assignment and identification of cells assigned to the “hub”
  Cell-types were assigned by performing gene scoring using the MAGIC imputed gene expression known markers (see Appendix FigS3). Cells were annotated by setting specific threshold for each cell-type. Any cell assigned to two or more cell-types were discarded from annotation. “hub” cells were identified as being a member of a leiden cluster containing more than 80% of unannotated cells. “hub” markers were identified by performing differential gene expression via Wilcoxon ranksum test, comparing the “hub” cells to the rest.”

Specifically, any leiden cluster composed of ≥80% of unannotated cells to a specific fate/cell type, using known terminal markers of differentiation (shown in Fig 1B), was considered to be part of the “hub” state. The resulting selected

clusters were tightly connected and located in a convergence point downstream of trunk and cranial neural crest populations and all known progenies (Fig 2A,B). The common markers of the “hub” were detected using differential expression analysis between this group of cells and all the rest of cells (neural crest cells and their progeny) (shown in Fig 2D). To assess intra-”hub” heterogeneity we performed differential expression analysis between the leiden clusters composing the “hub” and put particular emphasis in uncovering potential pre-existing fate-bias by using a selection of known markers of the differentiated lineages that are neural crest/Schwann cell precursor-derivatives (see Fig 3A,C).

4) The authors mention in the text: "When observing the root of the resulting tree of transcriptional events, we observed that one of the directions of differentiation..." in context of Fig. 1 - however, there is no tree or trajectory shown in the Figure. The overall trajectory has to be established.

- The trajectory is now established and shown in revised Figure 1E. The trajectory is consistent with RNA Velocity and Cytotrace analysis shown for the revised UMAP plot in Figure 1C. Specifically, cranial and trunk neural crest converge towards the “hub” state and then cells radiate to all downstream fates. When focusing on the bottom part of the UMAP, the cranial neural crest (characterized by *Snai1* expression and onset of *Prrx2* and *Twist1*) also gives rise to mesenchyme. Additionally, mainly trunk neural crest cells give rise to sensory neurons. A second wave of “hub”-derived sensory neurogenesis follows.

5) Related to this, Figure 1 suggests that almost all derivatives are generated via SCPs. Do the authors interpret these data that none of the derivatives, including the entire population of enteric neurons/glia and sympathetic neurons, are directly generated from migratory NCCs? This contradicts previous literature and should be discussed. Again, the cluster labelled "SCPs" (i.e. nerve-associated cells) might just be wrongly annotated (see point 1) above).

- No, we do not suggest that all derivatives derived by going through the “hub” state. The single cell transcriptomics data are used to reconstruct the tree of possible transcriptional events, and it might, or might not coincide with the lineage tree (please see our latest review on a subject Erickson and Kameneva et al., 2022). To be more precise, we think that there are distinct theoretical situations that occur in the neural crest lineage. Firstly, some neural crest cells quickly slide through the “hub” state (for them, the “hub” is very transient to the extent that they might not upregulate the entire “hub” program, only recapitulating the basic properties). Secondly, some neural crest cells do not switch on the “hub” state program, and they tunnel to the terminal fates while bypassing the “hub”. Thirdly, many cells become nerve-associated SCPs representing the “hub”. To find out, which situation dominates embryonic development, we studied how the “hub” markers emerge in the tissue. These results support that numerous neural crest cells that assume fates before nerve-association do not switch on “hub” markers,

and rather tunnel via the "hub" to their final destinations. A notable exception is the autonomic fate-biased Sox10/Foxd3/Phox2b-expressing cells (FigEV4C, Appendix FigS5-7). Therefore, the "hub" represents an intercalated state, which appears predominantly for the populations of the crest that become associated with the nerves. However, in the data set, it is impossible to separate "lineage subtrees" of the neural crest that assume fates during migration from those that become "hub" cells because the unbiased clustering will align cells according to their similarity. Thus, despite the fact that the visualization of the data set resembles the universal lineage tree, we do not interpret it this way, and we will treat these observations with caution.

On the other hand, the intercalation of the "hub" between the migratory neural crest and terminal fates suggests that "hub" is indeed a prolongation or variation of the neural crest phenotype, which is adapted to the life on the nerves.

We explain this view in the Discussion:

Lines 684-703: "While visualization and construction of trees of transcriptional events from individual transcriptomes helps to formulate testable hypotheses regarding actual lineage transitions towards terminal states, these may not accurately reflect the actual clonal lineage tree. Based on the generated tree of transcriptional states, there are three possible scenarios of actual NCC lineage transition towards terminal fates. In the first scenario, some NCCs slide through the "hub" state without nerve-association to produce differentiated cell types. The second scenario suggests that NCCs do not switch on the "hub" state gene program and instead proceed to the terminal fates while bypassing the "hub" or tunnelling through the "hub". In the third option, numerous NCCs become nerve-associated SCPs expressing "hub" genes, followed by generation of definitive cells types in nerve-dependent fashion. The real clonal lineage portrait can be the sum of all three options mixing in different proportions. In any case, the intercalation of the transcriptomes of cells of the "hub"/SCP state between those of NCCs and downstream terminal fates suggests that SCPs are indeed a cellular state functioning as an extension of NCCs in terms of multipotency and expressed genes. The cells of this "extension state" differ from the NCCs in that they use peripheral nerves as their navigation routes and express additional gene expression programs. According to experimental evidence, these additional programs gradually increase in the neural crest-derived "hub" cells after nerve-association, and are likely driven by strengthening interactions between the cells and the nerve fibers."

6) Likewise, based on Fig.1 and Suppl. Fig. 1, melanocytes appear to be generated only from cranial neural crest, and neither from the SCP "hub" state nor from trunk NCCs. This also contradicts previous literature, including some of the authors' own studies, and should be clarified.

- We agree, and we explain the situation with melanocytes in the following way: melanocytes represent the problem for visualizing their root (origin) in such

UMAP embeddings, because they originate from both neural crest populations and from the “hub”. The different timeline of their development depending on the location and different starting population disrupt the stability of attachment of melanocytes to the one place of the tree. In addition, the majority of melanocytes in mouse embryos originates from the cranial neural crest population at the time it becomes nerve-associated with the cranial nerves (Adameyko, Lallemend et al. 2012). Later, melanocytes do not form at such high density anywhere in the body. Please have a look at Adameyko et al. 2012 to see the progression of melanocytes in mouse embryos at cranial and trunk levels. This massive production of melanocytes around cranial nerves VIII-IX-X at E9.5 causes the effect that many of them in the dataset from E9.5 comes from this location. This previously drove the overall affinity of the melanocyte cluster to cranial NCCs, although many melanocytes in the data set came from trunk NCCs and “hub” state. The UMAP representation will tether the melanocytes to the highest likelihood origin place, tearing the other origin from other less represented locations. In the revised manuscript, the injection of additionally sequenced cells from the trunk separated the melanocyte cluster from the cranial neural crest (as expected) but did not connect it to any specific branch because of the multiple origin of melanocytes (Figure 1B-F). At the same time, in the revised SCENIC-based embedding (based on gene expression regulation), the melanocyte branch forms and becomes connected to SCPs of the “hub” (see Figure 1G). Overall, the diverse origin of melanocytes cannot be efficiently analysed with gene-based data representation and works better with the SCENIC-based tree. In the latter case, the analysis of transcriptional states leading to melanocyte formation are informative and this is one of the points we wanted to make with this study.

- We provided the following explanations in the revised text:

Lines 232-238: “When it comes to the developmental origin of melanocytes, the corresponding branch in the gene-based embedding (Fig 1B,E) does not attach to the rest of the tree likely because of the multiple convergent origin of pigment cells, which are derived from migratory neural crest and from different populations of cranial and trunk SCPs. At the same time, the SCENIC-based embedding (Fig 1G) connects the melanocyte branch to the rest of the tree, which suggests common mechanisms in melanocyte fate biasing, specification and differentiation.”

7) On p.7, the authors state that they don't focus on mesenchymal fate-biased cells (i.e. cranial NCCs (?)) and, yet, later in the manuscript (e.g. Fig. 3A) they appear to specifically include the cranial NCC population. This is confusing and should be clarified.

- To remove any confusion and to make the story complete, we included all neural crest and all types of progeny into the revised manuscript. Previously, the logic was the following: cranial skeletogenic neural crest also gives rise to

Schwann cell precursors sitting on the cranial nerves and satellite glial cells of cranial ganglia. Thus, we wanted the cranial crest to be present in the atlas as well. Because we were not interested in mesenchymal progeny, as it does not arise massively from the "hub" (despite there are some marginal exceptions), we trimmed the mesenchymal progeny of the cranial crest. Now, we added back the cranial neural crest-derived mesenchyme (Fig1B, Appendix FigS1) to present a global view on neural crest-derived populations. Now the revised embeddings shown in Figure 1 and other figures contain mesenchymal differentiation as well (purple branch). Furthermore, we repeated the regulon analysis for both subgroups of neural crest (Appendix FigS8) and include the following explanations:

Lines 378-392: "Next, we questioned the differences in regulon activity and transition into the "hub" in the cranial and trunk neural crest cells, which are both multipotent populations distinguished by the ability of cranial neural crest to give rise to ectomesenchyme, a property lacking in the trunk neural crest. Additionally, a significant portion of the cranial neural crest cells delaminates from the anterior Hox-negative neuroepithelium. During a comparative regulon analysis between the trajectories of cranial versus trunk neural crest progressing towards the iSCs stage (Appendix FigS8), we observed a significant overlap between regulons detected in the cranial and trunk trajectory (175 regulons in common, with 28 trunk-specific and 14 cranial-specific). While cranial-specific regulons included Tfap2c, Msx1/2, Lbx2, trunk specific regulons included, as expected, Hox genes and Six1/4, Neurog1, which correspond to sensory neurogenesis prominent in the trunk region. However, both cranial and trunk neural crest cells converge later towards the "hub" state, as shown by RNAvelocity and Cytotrace (Fig1C,H) and contribute equally to the generation of multipotent Schwann cell precursors found on cranial and trunk peripheral nerves."

8) In Supplementary File A, (Page1, A) the authors show a "UMAP embedding of the QC-filtered dataset" and then highlight the clusters used for the rest of the study. However, the UMAP of the highlighted clusters does not seem to match the final UMAP embedding shown in Fig 1A and the rest of the paper. Could the authors clarify the relation between these different UMAPs?

The final UMAP shown in the revised main figure (revised Fig 1B) and the rest of the paper is different from the initial one containing all sequenced cells with large proportion of CNS populations (Appendix FigS1). This general dataset has been reprocessed and re-analysed (including UMAP generation), as it is common to do when sub-setting single cell data. To be more precise, in this reanalysis step, we selected for Sox10+ neural crest-derived cells, NCC-derived peripheral neurons and chromaffin cells (identified by *Isl1* expression) and NCC-derived mesenchyme. During this process, a step of diffusion maps has been added before the updated UMAP generation, leading to a 2D embedding that is more representative of the cell transitions from NCC to all

downstream progenies, because the contaminating populations were removed (Appendix FigS2).

9) Related to Figure 1, the authors state: "Joint UMAP embedding based on 1) multiscale diffusion space derived from PCA space (generated by gene-based pagoda2) and on 2) multiscale diffusion space derived from regulon activity scores (AUC, generated by SCENIC) showed striking similarities in their structure. This observation suggested that the main differentiation paths of the NC lineage could be abstracted to the level of transcription factor (TF) activities alone."

Could the authors indicate where (and if) they show these two UMAP embeddings in the paper and what is the exact similarity/overlap and differences if any?

We apologise for the possible confusion the use of different embeddings caused. We show both gene-based and SCENIC-based UMAP (Fig 1B – gene-based UMAP and Fig 1G – SCENIC-based UMAP). The reason why we introduce both of them is the similarity of the global structure in both cases, as we focus throughout the manuscript on the gene-based embedding, but use the SCENIC-based embedding to perform the regulon activity analysis along the path linking trunk neural crest to immature Schwann cells (see Fig 5). It is worth noting that while consistent with the gene-based UMAP, SCENIC-based UMAP fails to recover the fine terminal states of myelinating Schwann cells and terminal Schwann cells (shown in detail in Fig 7). Thus, even though the use of each type of embedding has its advantages, analysis of subpopulations of cells works better in the case of the gene-based UMAP, mainly because of the fact that the discerning markers are not transcription factors (of which the activity would be picked up by the AUC score) but rather signalling molecules or receptors.

For instance, the revised text below shows how we use both types of embeddings to make better interpretation of our results using the examples of melanocytes and sympathetic neurons:

Lines 232- 243: “When it comes to the developmental origin of melanocytes, the corresponding branch in the gene-based embedding (Fig 1B,E) does not attach to the rest of the tree likely because of the multiple convergent origin of pigment cells, which are derived from migratory neural crest and from different populations of cranial and trunk SCPs. At the same time, the SCENIC-based embedding (Fig 1G) connects the melanocyte branch to the rest of the tree, which suggests common mechanisms in melanocyte fate biasing, specification and differentiation. Similarly, the sympathetic neuronal branch stays disconnected in the gene-based embedding, as sympathoblasts can originate directly from the ventral pathway of neural crest migration and via SCPs along the ventral nerves (Weston 1970, Le Douarin 1974, Kastriti, Kameneva et al. 2019). In the SCENIC-based embedding (Fig 1G), the sympathetic neuron branch connects perfectly due to the common regulation of differentiation process.”

10) The authors should compare the gene expression signatures that they found to published literature. For example, the gene expression signature of the endoneurial fibroblast population should be compared to the one shown in Carr et al, 2019.

The genes we use as fibroblast markers in Fig1A (Cd34, Dlk1, Fn1, UMAPs shown in Appendix FigS3) and Fig6E (Cd34, Pdgfra) are in agreement with the paper from Carr and colleagues and other works (Joseph, Mukouyama et al. 2004, Richard, Vedrenne et al. 2014, Carr, Toma et al. 2019).

However, to satisfy the curiosity of the reviewer we extracted the data set from Carr and colleagues work and re-analysed the scRNAseq data. We generated a dataset of Pdgfra+ cells, similarly to the strategy of the authors, and combined with captured SOX10+ Schwann cells. We then applied CONOS integration with label transfer between that subset and the post-natal subset of our glial trajectory. Label transfer led to a match between Schwann cells from the two data sets. The signature of epineurial and perineurial fibroblasts matched to our endoneurial fibroblast population due to their mesenchymal program. Interestingly, using this approach endoneurial fibroblasts from Carr et al. were assigned to our endoneurial fibroblast and Schwann cell population, with a higher proportion assigned to the later. Upon close inspection of the upregulated genes in the endoneurial cluster from Carr et al, *Apod, Fos, Egr1, Jun* were present. These markers, which are not mentioned in their study, are also highly expressed in our Schwann cell population at the level of bifurcation between non-myelinating and myelinating Schwann cells. We assume that these genes are part of a general program of activation shared by different cell types, as we also detected this signature on the re-analysed satellite glia from Mapps, Aurelia A et al. (Appendix FigS4). This signature may reflect a transient program that will downregulate upon biasing towards the endoneurial fibroblast fate. Instead of using CONOS results which are not accurate in this case, we propose to show three markers mentioned in their study as being endoneurial fibroblast specific: Sox9, being the sole marker validated in their study for that population, and Cp and Enpp2 which are shown as specific markers in their computational analysis.

Please find the related figure below:

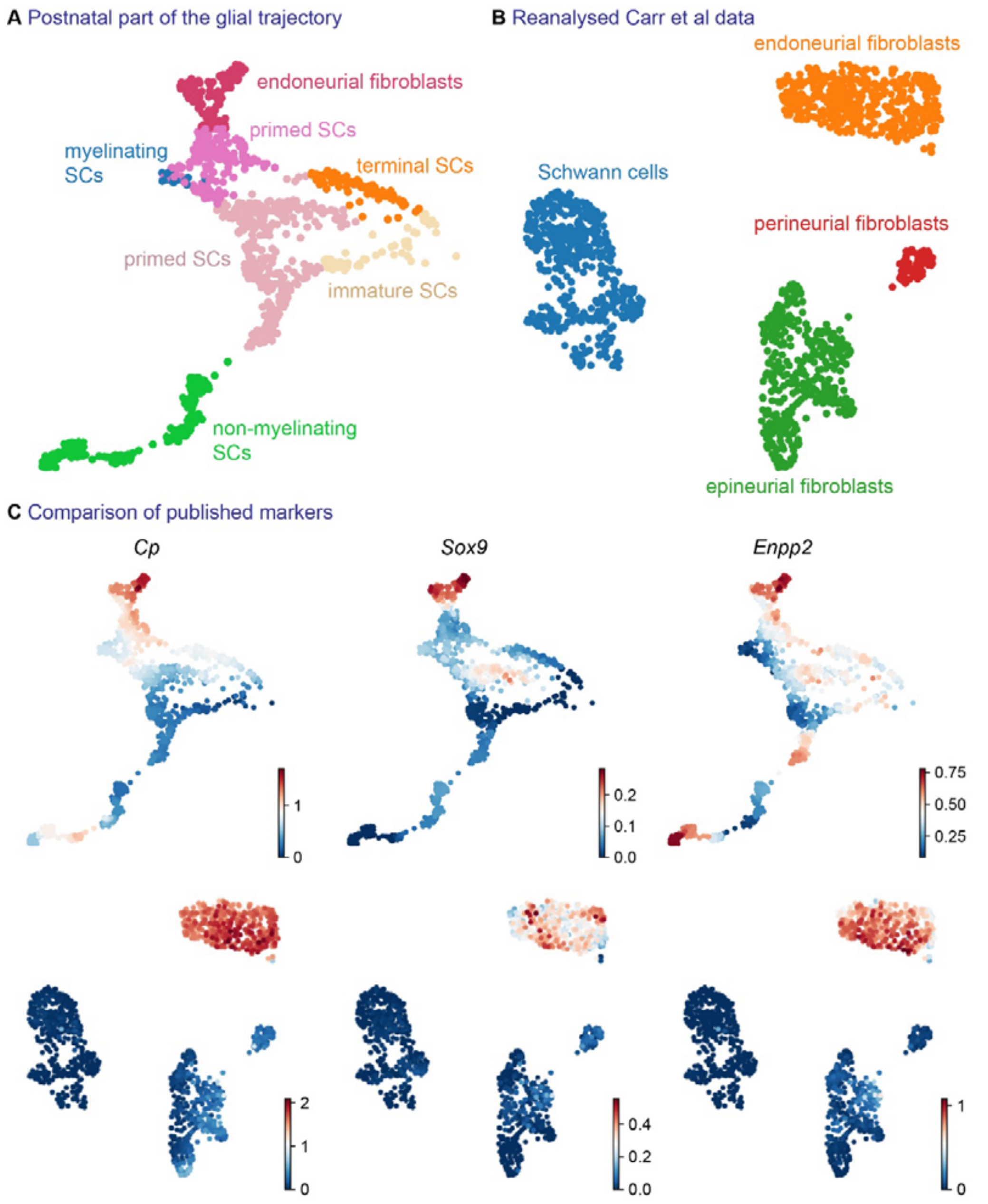

Fig1. A. Subset of the glial trajectory from our dataset, keeping only post-natal cells (P0 and later). B. Reanalysed Carr et al dataset (Carr, Toma et al. 2019), combining Pdgfra+ cells from uninjured data and glial cells from injured data (as none were present in uninjured). epi: epineurial, pre: perineurial, endo: endoneurial. C. Comparison of markers discussed (Cp and Enpp2) and validated (Sox9) as endoneurial specific in Carr et al study. Genes are knn smoothed and plotted on our dataset (top) and Carr et al reanalysed dataset (Carr, Toma et al. 2019).

Furthermore, they also do not comment on the discrepancies between their SCP and iSCs signatures and the ones proposed a while ago by the Jessen and Mirsky

lab. Moreover, the markers that they used to delineate the adult mSCs and nmSCs are not very common in the field, so they should comment on this.

Regarding the published markers of the Schwann cell lineage by Jessen and Mirsky, we do not believe there are major disagreements between those and the markers we uncover. To show this effectively, we generated Fig 7E, where the dynamics of many reported Schwann cell markers from the work of Jessen and Mirsky are shown. We also show markers resulting from unbiased analysis (dot plot in Fig6A and some markers in Fig6E), but these are only shown to offer more knowledge to the field and not to contradict previously published works.

11) Likewise, the metaregulons presented in Fig. 2 need some discussions in view of current knowledge in the field. For instance, how to explain the presence of Foxd3 or Jun (p. 11) in metaregulon 6? Both genes have been shown before to be associated with early steps in NC/SCPO development. In addition, Jun is a negative regulator of myelination (Parkinson et al. 2008) by inhibiting Krox20-induced myelin protein expression (Mpz), so it is surprising to find it in the same group as e.g. the pro-myelinating factor Pou3f1 (Oct6). Is there any evidence for their statement (p.11) that "the role of FoxD3 changes from transcriptional repression ....to transcriptional activation..."?

- We thank the reviewer for this comment, as it is crucial to interpret the results in relation to the knowledge in the field.

Indeed, Jun is a negative regulator of myelination but it is not a surprise to find its expression in the same regulon as Pou3f1 for two reasons. Firstly, along the developmental time, the point of Pou3f1(+) regulon activation corresponds to the generation of terminal Schwann cells, which we now show in the revised manuscript to have high levels of the protein (OCT6). Along the same path, Jun (coding for c-Jun) is identified as a marker reaching a peak in the Schwann cell population before their split between myelinating and non-myelinating Schwann cells. Thus, both markers co-activate by an early onset of activity in the end of the subset trajectory to radiate the cell fate choices toward different directions. Another temporal difference is seen, as the activity of the Jun(+) regulon shows an downregulation upon the time that the Pou3f1(+) regulon shows the peak of activity which is required for myelination and Egr2 expression (Fig EV2).

This is specifically discussed in the revised manuscript:

Lines 325-332: "Notably, Jun represses myelination (Parkinson, Bhaskaran et al. 2008), whereas Pou3f1 and Sox10 synergistically promote myelination (Britsch, Goerich et al. 2001, Ghislain and Charnay 2006, Schreiner, Cossais et al. 2007). The simultaneous presence of active repressors and facilitators of myelination within the same group of coordinated regulons reflects opposing cell biasing tendencies, which eventually result in separation of Schwann cell fates towards myelinating and non-myelinating, as seen by the decrease of Jun activity and increase of Pou3f1 activity (Fig EV2)."

In the revised manuscript, we put focus on the discussion of metaregulon dynamics in relation to the stages of neural crest, “hub” and Schwann cell precursor to Schwann cell differentiation in the section *“Coordinated metaregulons maintain the neural crest-like state in “hub” cells”*

- As for the request to provide evidence of the role of Foxd3 as a transcriptional repressor to activator, this was recently shown in a work focusing on zebrafish neural crest (Lukoseviciute, Gavriouchkina et al. 2018), but our revised regulon analysis did not reproduce Foxd3(+) activity, thus we had to remove this phrase.

12) Do the regulons shown in Fig. 2(B) correspond to the entire set of regulons resulting from the analysis? If no, could the authors provide the complete set? And do the genes mentioned in each regulon correspond to the entire list for that particular regulon? Is the principal tree/pseudotime trajectory mentioned here the same as referred to in my point 4 above?

We addressed this comment by providing the extended list of all regulons in each of the five metaregulons, which can be found in the new FigEV2. In this new supplementary figure, we show all regulons composing five meta-regulons clusters. We also show the target genes for Six1 and Ets1 regulon for the inter-intra module correlation analysis in Fig. 5F. The trajectory here has been selected from the main inferred tree (shown on Fig. 1E) by sub-setting the path from trunk neural crest cells to immature Schwann cells.

The entire spectrum of regulons covering the neural crest lineage atlas can be generated by running the code that we openly provide in the methods section. We do not provide such list because of space limitations, as this list will contain dozens of metaregulons with hundreds of regulons identified. Therefore, anyone interested in regulons guiding specific transition of interest can produce their map using our dataset and the code.

13) On p.14 and elsewhere (related to Fig. 4), the authors suggest a new path to myelination. How do they explain in this context (absence of previously dubbed immature SCs) the process of radial sorting? Are these Pou3f1-expressing (OCT6+) immature pro-myelinating SCs associated with large caliber axons? If Oct6 is ablated in this population does this, in addition to loss of mSCs, result into loss of the endoneurial fibroblast population, too? Is it known whether Oct6 binds to any transcriptional locus that drives fibroblast gene expression? How would myelinating (!) SCs be able to generate endoneurial fibroblasts without de-differentiation? In sum, there are several novel (and surprising) statements regarding the SC lineage tree that should be, at least to a certain extent, functionally validated. Otherwise, one starts to doubt the validity of the conclusions drawn from the (after all "descriptive") transcriptomics data.

This is also relevant because the authors' only approach for functional validation in this context actually failed: they validated the functionality of Nr4a2 that they have introduced as a novel gene in terminal glia specification or myelination. However, upon loss of this nuclear receptor, mSCs (and nmSCS) are still formed normally during development.

We thank the reviewer for this comment, and we attempted to clarify the situation. This led us to one of the most interesting and unexpected observations. When taking a closer look to that branch of cells after adding new sequenced cells, we uncovered the unique transcriptome of developing terminal Schwann cells of the neuromuscular junction, along with important validations of specific markers that are in agreement with recently published data from the adult neuromuscular junction (Castro, Taetzsch et al. 2020) (Fig 7, Fig EV4). Therefore, this branch is not a new path to myelination as we previously thought. We should have been more attentive in the beginning, as the reason for the initial misinterpretation was rooted in a human factor, not in the quality of dataset or our analysis. Please see the updated text in the revised manuscript:

Lines 515-522: "Differentially expressed markers suggested an early splitting cell type derived from iSCs, which corresponded to terminal SCs of neuromuscular junction SCs (tSCs) (Fig 8C,E, Fig EV4), based on previously reported markers such as Cspg4, Itga8, Slitrk3, Cpm, Pou3f1 (Castro, Taetzsch et al. 2020). Biological validations showed the early origin of Itga8+/Cpm+/Pou3f1+ terminal SCs as seen by RNAscope® or immunofluoresnce against OCT6 (encoded by Pou3f1) on E18.5 Plp1YFP-traced embryos when recombination was induced at E13.5 (Fig 8F, Fig EV4F)."

Lines 588-596: "Next, OCT6^{high} cells (encoded by Pou3f1) were predominantly found in developing and postnatal limbs, with an increasing gradient of OCT6 levels from proximal to distal part of the peripheral nerves (Fig 8E). These cells where characterized by the signature of terminal neuromuscular SCs as seen by the expression of Cpm, Itga8, Slitrk3, Cspg4 and other markers previously shown enriched in this SC subpopulation (Fig EV4A,B,D, Appendix FigS13). As compared to this subgroup of limb-dwelling glial cells, sympathoadrenal or enteric glia had much lower Gjc3 expression during development, which caught up with expression levels of Gjc3 found in embryonic limbs only postnatally (Appendix Figure S13)."

Furthermore, the ablation of Pou3f1/OCT6 only in the OCT6^{high} cells of this branch was not feasible, as this would require the generation of a novel conditional knock out strain. However, the histological validations we performed during the revision and additional cross-checking of the terminal NMJ Schwann cell markers provided a strong proof for these conclusions.

Regarding the other part of the referee's comment: it is indeed unfortunate that Nr4a2 mutants do not show any defects in myelination or terminal NMJ glia, but we do not feel comfortable removing these negative results from the Appendix (revised figure found in the Appendix as FigS12)

14) The authors propose that there is heterogeneity in SCPs/SCs associated with a specific spatial and temporal code. However, the authors completely neglect the presence of satellite glia in different ganglia (sensory, different types of autonomic) that could explain their findings. The statement made on p.7 that starting from E11.5 all Sox10+ cells are associated with peripheral nerves is wrong as satellite glia that are known to express lineage/location-specific markers also express Sox10 and many other glial markers. This issue needs to be addressed.

- We agree and we specifically addressed the presence of satellite glial cells and boundary cap cells in our revised dataset, using previously published works to select appropriate markers (Mapps, Thomsen et al. 2022). Thanks to the recent addition of newly sequenced cells derived from dorsal root ganglia, satellite glia and boundary cap cells are now identifiable (Fig1B, yellow and red clusters respectively). Satellite glia found in the dorsal root ganglia were identified by re-analysing Mapps, Aurelia A et al single cell data, and applying CONOS for projection of published satellite glia to our dataset (with label transfer). We found that Fabp7+/Rgcc+ cells from their satellite glia population perfectly matched with our E12.5 glial population obtained from dorsal root ganglia which express the markers Fabp7, Ptn and Rgcc (Appendix FigS3, S4). Furthermore we identified boundary cap cells via the expression of Prss56, Egr2, Wif1, Hey2 (Coulpier, Le Crom et al. 2009) (Appendix FigS3). Finally, addition of more cells sampled from the developing gut enriched the enteric glia population, which now clearly stands out in the atlas dataset (Fig1B).

We incorporated the following information in the manuscript as follows:

Lines 244-251: *"Finally, we identified and connected the satellite glial cells found in association with sensory neurons within dorsal root ganglia, and boundary cap stem cells (BCCs) associated with motor exit points from the neural tube. Using previously known markers (Coulpier, Le Crom et al. 2009, Mapps, Thomsen et al. 2022), we found the satellite glia and BCCs in position where the neural crest populations transit into the "hub" (Fig 1B, Appendix FigS4). The expression of "hub" markers in these cell populations suggests that they represent highly specialized subtypes of multipotent neuron- and nerve-associated cells."*

- Regarding the wrong statement identified above, we now rephrased it and hope that it satisfies the reviewer:

  Lines 191-195: ". At E10.5, most of SOX10+ cells become nerve (or peripheral neuron)-associated. Starting from E11.5, all SOX10+ cells are associated with the outgrowing peripheral nerves or neurons in consistency with previous studies (Adameyko, Lallemend et al. 2012, Dyachuk, Furlan et al. 2014, Furlan, Dyachuk et al. 2017)."

15) In the last chapter of the Results section, the authors say that malignant cells re-enact developmental gene expression. This is not novel; the findings presented in Figures 6, 7 are rather descriptive (the transcriptome of some NCCs/SCPs/SCs maps to some tumor cells) and do not add much to what is already known in the field. In their present form, these data are preliminary (because they don't give novel insights, for instance, into how NCC/SCP signatures contribute to tumorigenesis, etc.), the link to the rest of the paper is poorly established. I suggest to delete these figures.

- We thank the reviewer for this fair opinion. We were leaning towards removing these data, but consulted our Editor, who on the opposite supported keeping these data and figures to showcase the possible types of analysis in case the neural crest tumour community will be interested in using our data in this way. The novelty in these comparisons is better defining SCPs in their temporal and spatial diversity, which improved the mapping and analysis of tumour cell similarity. Anyways, to find a compromise, we placed all tumour-related data into supplementary information and reduced their discussion in the main text. We thank the reviewer for sharing the views regarding these data.

16) The legend of Fig S1 D reads "D) Dot plot showing top differentially expressed genes (left) and regulon AUC scores (right) between the two NCC selections made in (C)". However, in the figure there is no dot plot or regulon AUC scores in D, but what seems to be a feature plot differentiating between cranial and neural stem cells.

We want to thank the reviewer for spotting this inconsistency. In the revised manuscript, we decided to replace this figure completely, as the addition of the neural crest-derived mesenchymal cells to the atlas clearly showed the difference between cranial (with mesenchymal potency) and trunk neural crest clusters (Fig 1B). The cranial neural crest-derived mesenchymal progeny became a part of the atlas during this revision.

17) With respect to Figure 3, the authors mention that "a version of the NC and SC developmental tree as a system of interconnected "nodes"..." - Is this a new tree fitting or merely a subset of the tree referred to earlier?

In the revised version of the manuscript, we abandoned this type of data representation, and now we use subtrees (we refer to them as subtrajectories) generated for Figures 5 (neural crest to immature Schwann cell regulon analysis), 6 and Appendix FigS9-11 (bifurcation analysis). These are indeed subtrees representing a subset of the main tree generated from the whole data set (Fig 1E).

18) In the discussion, the authors state that Dhh is expressed in the progenitors of endoneurial fibroblasts that are stated in this paper as Pou3f1-expressing (OCT6+)

immature pro-myelinating SCs. However, in Figure 4J, high Dhh gene expression does not appear to be present in this population, how can this be explained?

This came up as an unexpected issue, and we are thankful for pointing it out. In order to address this discrepancy, we reanalysed the cells expressing high levels of *Pou3f1*, which we previously termed "immature promyelinating Schwann cells" and were stemming out of immature Schwann cells. In these cells, we uncovered the specific molecular signature including *Pou3f1, Itga8* and *Cpm*, which urged us to further validate the location of these cells experimentally. As a result, we identified these *Pou3f1, Itga8, Cpm* $Plp1^{YFP}$-traced cells in association with tips of axons innervating hindlimb muscles at E18.5 (Fig 8E). After we further mined the literature and used published bulk transcriptomic data set from Castro and colleagues, we identified these cells as terminal Schwann cells of the neuromuscular junctions (Fig EV4).

In relation to the previous study from Joseph et al. where the authors used Dhh-Cre lineage tracing to trace the newly-generated endoneurial fibroblasts, we now represent the dynamics of *Dhh* expression in new Fig 7E. High levels of *Dhh* are observed in the cells we name primed Schwann cells I and in agreement with the mentioned study they give rise to endoneurial fibroblasts according to RNA velocity and Cytotrace (updated Fig 7D). Hence, the revised manuscript shows no disagreement with previous studies. In this light, we described the bioinformatics and experimental observations in the revised manuscript:

Lines 494-497: "Additionally, during perinatal stages in rodents, a Dhh-expressing subpopulation of iSCs has been described to generate endoneurial fibroblasts (Parmantier, Lynn et al. 1999, Joseph, Mukouyama et al. 2004, Sharghi-Namini, Turmaine et al. 2006)."

Lines 515-522: Differentially expressed markers suggested an early splitting cell type derived from iSCs, which corresponded to terminal SCs of neuromuscular junction SCs (tSCs) (Fig 8C,E, Fig EV4), based on previously reported markers such as Cspg4, Itga8, Slitrk3, Cpm, Pou3f1 (Castro, Taetzsch et al. 2020). Biological validations showed the early origin of Itga8+/Cpm+/Pou3f1+ terminal SCs as seen by RNAscope® or immunofluoresnce against OCT6 (encoded by Pou3f1) on E18.5 Plp1YFP-traced embryos when recombination was induced at E13.5 (Fig 8F, Fig EV4F).

Lines 525-528: "Primed SCs I show progressive downregulation of Ngfr/Cdh2 with Pou3f1/Dhh upregulation differentiating towards Igf2+/Cd34+/Pdgfra endoneurial fibroblasts (Fig 7C,D,E,F) (Richard, Vedrenne et al. 2014, Carr, Toma et al. 2019). "

Minor points:

Because of the missing figure labeling and inconsistent figure formatting, it was somewhat tedious to evaluate the data presented in the figures.

- We apologize for this, and we hope that we managed to improve the revised manuscript by carefully referencing the panels and placing all supplementary figures in the Appendix. Please note that now we have 8 main figures, 4 Extended View figures and figures S1-S15 are found in the Appendix.

On page 2 (summary) there is a word missing in this sentence: "Our results revealed that early SCPs and late migratory crest show an indistinguishable...

We have rephrased the corresponding phrase to "*By using single-cell transcriptomics to generate the atlas of the entire neural crest lineage, we show that early SCPs and late migratory crest have similar transcriptional profiles..*" (lines 38-40).

On page 3, wrong references (Le Douarin, Westin) are given regarding the statement that SCPs give rise to multiple cell types.

We thank the reviewer for pointing out this mistake. This part is now rephrased, and all relevant references are cited:

Lines 73-80: "They spread throughout the developing body via branching innervation, detach from the nerves in specific locations and produce large quantities of pigment cells, autonomic and enteric neurons, chromaffin cells of the adrenal medulla and specific mesenchymal populations within nerves and cranial locations (Joseph, Mukouyama et al. 2004, Adameyko, Lallemend et al. 2009, Nitzan, Pfaltzgraff et al. 2013, Dyachuk, Furlan et al. 2014, Uesaka, Nagashimada et al. 2015, Espinosa-Medina, Jevans et al. 2017, Furlan, Dyachuk et al. 2017, Kastriti, Kameneva et al. 2019)."

On page 6, the stage(s) at which Plp-CreERT2 and Sox10-CreERT2 were induced should be indicated.

- When using these tamoxifen-inducible strains, we always performed the tamoxifen injections as reported in the Methods, 48 hrs prior to collection of the tissue. We clarify this in the suggested place in the revised manuscript:

Lines 135-138: "Neural crest-derived cells were obtained via Cre-based labelling with the constitutively active Wnt1-Cre;R26TOMATO and tamoxifen-inducible $Plp1^{CreERT2}$*;*$R26^{TOMATO}$ *and* $Sox10^{CreERT2}$*;*$R26^{TOMATO}$ *transgenes, with recombination induced 48hrs prior to tissue collection (Fig 1A)."*

In Fig 2(B): What is meant by 'Density', what are the values defining the different fields ranging from yellow to purple?

In the revised figure (now Fig 5), we have changed the denomination of the y axis on that panel to "Proportion". The colors from yellow to purple indicate the contribution of developmental times (in cell proportions) along the pseudotime axis, and a label for this has been added next to the panel.

In Fig 2(D): What are the p values associated with the correlation?

We removed that information and panel because we decided that this was unnecessary. The focus on Six1 and Ets1 is already justified (1) biologically as both are known NC and neuronal markers and (2) computationally by the presence of repulsion between the two sets of target genes of these two transcriptional regulators (Fig 5). The new experimental validation experiments are shown in Figure 5D.

In Fig 2 (G): Heatmap legend is missing.

Thank you for noticing this mistake. The revised figure (Fig 5), as well as others with heatmaps, have been double-checked, and they all have corresponding legends.

In Figure 3 and Suppl. Fig. 4, what is the evidence for convergence between a NCC and a “hub” state, i.e. why is there a bi-directional arrow between the two states?

We thank the reviewer for focusing on this. We have removed the previous simplified tree and instead show the updated tree in Fig 1E combined with RNAvelocity and Cytotrace vectors shown in Fig 1C. The results show the convergence of trunk NCC and cranial NCC into the “hub” state, which is supported by experimental evidence that SCPs covering cranial nerves are multipotent and generate majority of cranial melanocytes and chromaffin-like cells of the carotid oxygen sensing organ (Adameyko, Lallemend et al. 2012, Hockman, Adameyko et al. 2018).

Fig. 4(E) is referred to in the text before 4(A) and others.

We have corrected all such instances.

Fig 6(A): "We extracted single cells forming a trajectory from a single patient and applied..." - why was only a single a patient chosen for this?

We focused on PRAME+ class 2 tumour cells from a single patient because we aimed to examine large number of cells while avoiding possible patient-wise batch effect (now explained in Methods, see “Comparison with uveal melanoma data”, lines 1180-1206).

The exact message of the middle panel for Fig 6(A) is not clear as there are significant differences between what the figure legend says and what is mentioned in the text.

The reviewer is right. Please find the corrected phrase in the manuscript:

Lines 624-628: “As expected, we found that the majority of the tumour cells along the entire progression identified by CytoTRACE mapped to the population of embryonic melanocytes (Appendix Figure S15A). At the same time, a

significant portion of melanoma cells appeared transcriptionally similar to SCPs and the NC (Appendix Figure S14C).”

The legend to Figure 7(A) says "uveal melanoma" - it should be "neuroblastoma".

The legends of the revised figures related to this (now in the Appendix, as FigS14 and S15) were proofread, and the mistake was corrected.

On page 9, the authors describe Ednrb, Heyl and Fabb7 as glial markers. This should be supported by references. Furthermore, the authors say that the Sp7 regulon is absent upon NCC differentiation into SCPs. In both figures, 2B and S3, Sp7 is not depicted, though; this should be corrected or clarified. Moreover, metaregulon 4 contains Ets1 and Tfap2a regulons that are described as essential for NC gene expression program. References for this statement are missing.

We agree and we are now referring to the original publications every time we mention these or other markers.

Concerning the regulons, our revised analysis following the addition of newly sequenced cells provided more reliable sets genes causing that we no longer focus on less significant Sp7(+) regulon.

On page 11, the last sentence before the new subchapter should be written more clearly. What is meant by locked between the activity of metaregulon 1,2, and 6? Also including 3?

We agree that that sentence was not well written. We rephrased and expanded the text as follows:

Lines 409-415: “herefore, the “hub”/SCPs might be defined as a cell state with re-activation of metaregulon 5 coinciding with gradually reducing activity of metaregulons 1 and 3 (linked to neural crest properties and cell reprogramming) before the activation of metaregulon 2 (linked to the terminal differentiation of Schwann cells) (Fig 5B,C). Thus, the “hub” state is distinct in terms of regulation from the majority of the neural crest cells where metaregulons 1 and 3 are dominating.”

On page 37 (supplementary figure legends), the authors should include the references for the published markers they have used.

The revised UMAPs of genes that we used to annotate the clusters in Fig 1 are now shown in Appendix Figure S3. Marker genes used to annotate the cell types were obtained from (Simoes-Costa and Bronner 2015, Tabula Muris, Overall et al. 2018) (referenced in the Results and Appendix).

In Figure 5, B should be in bold. Importantly, in B and D, the labeling for 'sensory nerve' and 'visceral nerve' should be reconsidered; the labelled structures appear to be cartilage, not nerves.

We corrected these in the revised figured (new Figure 8), and the annotations of structures are now placed closer to the corresponding area.

On page 27, in Figure legend 6B, the authors write that the dot plot shows the top 8 gene markers, however there are actually 10 displayed, this should be corrected.

In the revised figure (now Figure 7), we show five unbiased markers per cluster, which is also corrected in the figure legend.

In Figure 7, why do the dot plots show bipotent sympathoadrenal progenitors in A and in B SCs? Should not both contain both of these populations to illustrate that in A the cells are more similar to progenitors, whereas in B to differentiated cells?

This figure was completely replaced by Appendix FigureS15 (specifically, the panel C), which makes it easier to compare tumour composition.

Referee #4:

Eleni et al use single-cell transcriptomics to address the question of Schwann cell precursor (SCP) potency and identity. The authors build their conclusions on RNA velocity, regulon (co-expression), cell embedding and FISH analysis taking advantage of new and publicly available data sets.

Overall they performed a detailed and thorough analysis that will be interesting for many in the field. Nevertheless, addressing some points might further improve the manuscript and will help to better grasp the main points of the study. This mainly refers to the “hub” state which is the main finding of the paper.

We want to thank the reviewer for making critical points that needed improvements. We addressed them all in the revised manuscript and hope it increased the scientific interest and value. All changes in the text are marked in yellow.

The single cell atlas is available for exploration here:

https://adameykolab.srv.meduniwien.ac.at/glia_gene_umap/ with the (gene-based embedding)

https://adameykolab.srv.meduniwien.ac.at/glia_scenic_umap/ with (SCENIC-based UMAP embedding).

Major points

The calculation of the regulons is only based on one portion of the NCCs. It is stated in the manuscript that this was done intentionally as the other NCC state was already analyzed elsewhere. However, it should be still included in the analysis maybe showing that these cells have a similar programme or a constrasting one. This can be a supplementary figure but the reasoning to exclude should come from the data and needs to be justified. Overall it seems that the “hub” cells orginiate from both mesenchymal and non-mesenchymal NCCs.

- We agree that the data suggest that “hub” cells originate from both cranial and trunk neural crest cells and it is an important thing to show. To address this concern, we applied the regulon analysis on the trajectory starting from cranial neural crest cells, and while we obtained similar clusters with similar trends and overlapping regulons, the main difference was that no sensory biased meta-regulon was detected in that part of the cranial trajectory (Appendix

FigS8). Furthermore, as expected, the specific metaregulon that is specific to trunk neural crest cells includes the targets of many Hox genes, which are not expressed in cranial neural crest cells. This result clarified some differences in regulation of fates and transitions between cranial and trunk neural crest. At the same time, the concept of a “hub” and transition into the “hub” from both cranial and trunk neural crest did not change.

- We have removed the phrase “*We intentionally chose to not focus on the mesenchymal fate-biased cells, as those develop from early cranial NC streams and were analysed previously*” and explain the results of the cranial versus trunk regulon analysis in the main text:

  Lines 378-389: “Next, we questioned the differences in regulon activity and transition into the “hub” in the cranial and trunk neural crest cells, which are both multipotent populations distinguished by the ability of cranial neural crest to give rise to ectomesenchyme, a property lacking in the trunk neural crest. Additionally, a significant portion of the cranial neural crest cells delaminates from the anterior Hox-negative neuroepithelium. During a comparative regulon analysis between the trajectories of cranial versus trunk neural crest progressing towards the iSCs stage (Appendix FigS8), we observed a significant overlap between regulons detected in the cranial and trunk trajectory (175 regulons in common, with 28 trunk-specific and 14 cranial-specific). While cranial-specific regulons included Tfap2c, Msx1/2, Lbx2, trunk specific regulons included, as expected, Hox genes and Six1/4, Neurog1, which correspond to sensory neurogenesis prominent in the trunk region.”

In Figure 3A it is suggested that there might be a fluidic fate split with two distinct “hub” cell types stemming from mesenchymal and non-mesenchymal NCCs. Would this mean that any cell type can originate from any NCC and that there is fluidity in the “hub” state? Can these states be defined via the regulon analysis (see first point)? Can the authors comment on other possible scenarios?

- We thank the reviewer for this question, and to address it, we created new figures explaining how the “hub” state is defined and what are the similarities between the “hub” and different migratory neural crest states. For example, in the revised Figure 2, we show how the “hub” is defined as a group of cells biased, but not committed to other fates (commitment was defined based on the expression of differentiation and terminal fate-related genes). Therefore, any neural crest cell, from either cranial or trunk population, can potentially give rise to “hub” cells according to transcriptional analysis data, supporting a fluidity concept mentioned by the reviewer. This is fully consistent with what we know about the formation of nerve-associated Schwann cell precursors in the cranial domain: those covering cranial nerves, for example, arise from the mesenchymogenic cranial neural crest cells and give rise to melanocytes and likely to chromaffin-like oxygen sensing cells of a carotid organ (for details, please see Adameyko et al., Development 2012 and Kaucka et al., Science Advances 2016). Next, we found the markers correlated with this “undefined”

“hub” state, and we validated these makers, including TF Sox8 in a functional experiments in updated Figure 3. In line with this, knockout and knockdown of Sox8 allowed to deplete “hub” state cells along the nerves in developing embryos, and this depletion was not related to decreased proliferation, but rather to increased rate of conversion of SCPs to other fates (sympatho-adrenal).
Finally, in the new Figure EV3, we show the similarity between “hub” and the migratory neural crest in terms of gene expression. Although the new comparative regulon analysis revealed differences between cranial and trunk neural crest cells in terms of regulation and fates, this does not suggest anything contradicting the fact that “hub” cells can be derived from any neural crest location. The spatial aspect, however, regulates the most probable future fate options taken by the “hub” cells in the cranial and trunk regions.

These points are covered in several parts of the revised manuscript:
Lines 168-176: “The structure of the NC pool revealed that cells within this population divided into two subgroups, separated by the presence of mesenchymal bias in cranial neural crest cells (Fig 1B, Fig EV1B). Intuitively, two parallel paths of NC differentiation converge in terms of non-mesenchymal fates at later time points. The examination of directionality of the NC and downstream non-mesenchymal populations with CytoTRACE and RNA velocity further produced a continuum of transitions and cell states towards the most differentiated transcriptional states (Fig 1 C,H). This indicates that the molecular changes that govern the switch from migrating NC to nerve-bound SCPs take place as a smooth transition of transcriptional states.”
Lines 217-219: “These “hub”-specific genes were downregulated when the cells entered differentiation into terminal neural crest fates (Fig 2C,D,E, Fig 3A,B).”
Lines 226-231: “On the other hand, the “hub” cells appeared heterogeneous by showing mixed expression of terminal fate-related genes, which suggests a number of fate biasing programs operating within the “hub” (preceding particular commitment and further differentiation) (Fig 3C). This is supported by the expression of fate-specific genes Pmp22 and Mpz (biasing towards SCs) or Phox2a/b and S100b (biasing towards autonomic neurons and glia), in the “hub” population (Fig 3A).”

Section “Loss of “hub” gene Sox8 results in SCPs defects and biased cell differentiation” (lines 253-275):
To test the function of the “hub”-specific transcription factor Sox8, we electroporated the trunk neural tube of HH11 chick embryos with a single plasmid containing gRNA, Cas9 and a citrine reporter (Gandhi, Li et al. 2021) to knock-out Sox8 in neural crest-derived cells (Fig 4, A-B). Embryos were then allowed to develop until HH24-25 (Fig 4, C), by which time the dorsal root ganglia (DRG) and visceral nerves had developed (Fig 4H). Quantification revealed a markedly reduced proportion of electroporated

CITRINE+/SOX10+ cells along the ventral nerve after loss of Sox8 as compared to controls (Fig 4 D-E and G). No significant differences were noted in numbers of cells undergoing mitosis between experimental and control embryos, suggesting that Sox8 might be involved in regulating migration or other processes rather than proliferation of neural crest-derived cells along the ventral nerve. This is consistent with a previous study suggesting a role for Sox8 in neural crest migration (O'Donnell, Hong et al. 2006). Despite the phenotype in the ventral nerves, the loss of Sox8 did not alter the number of glial (SOX10+) or neuronal (ISL+) cells in the DRGs (Fig 4 D-F), which supports that loss of SOX8 does not affect cell survival. By contrast, in the sympathoadrenal domain, the proportion of CITRINE+/SOX10+ nerve-associated cells appeared reduced, whereas the proportion of CITRINE+/TH+ cells significantly increased (Fig 4 I-J). This result suggests elevated rates of conversion of sympatho-adrenal SCPs into adrenergic fates. Taken together, high levels of Sox8 might specifically stabilise the SCP/"hub" phenotype and assist the migration of neural crest-derived SOX10+ cells along the peripheral nerves.

Lines 389-392: "However, both cranial and trunk neural crest cells converge later towards the "hub" state, as shown by RNAvelocity and Cytotrace (Fig1C,H) and contribute equally to the generation of multipotent Schwann cell precursors found on cranial and trunk peripheral nerves."

The data presented here stem from two different collections - previously prepared cells up to E11.5 and cells starting from E12.5. Could this explain the unexpected behaviour in Figure 2B with oscillations and a peak in E12.5 and later samples? Can the authors exclude a technical bias due to the data collection differences?

- To address this question, we additionally sequenced cells (during this revision) from E10.5 and E12.5 dorsal root ganglia (DRG). The resulting dataset shows that the decrease in *Ets1* combined with the peak in *Six1* regulons coincide with sensory neurogenesis (seen as the first wave) (revised Fig 5E-G), and is not related to any gap in sampling. After specifically checking the compositional effects, we are confident that oscillations and peaks arise not from any issues related to the timing of cell collection, but from compositional effects i.e. gene expression mosaics in individual cells in the same place on the trajectory that reveal that Ets1 regulon and Ets1 expression show contrary pattern to Six1 regulon and expression in the same cells independently of a developmental stage. This we additionally validated during the revision (please see Figure 5D-E). The cells inclining towards neurogenesis start to activate Six1-dependent program, which leads to downregulation of Ets1 program. Cells that do not engage into neurogenic program retain high levels of Ets1. The mixture of cells with high Six1 and low Six1 (and low and high Ets1 correspondingly) plotted on a pseudotime results in an oscillatory and antagonistic pattern the reviewer questioned. The new

experimental validations confirmed this logic and increased the confidence of our results. To show that these correlations are not due to batch effects due to the methodology, we ran the analysis from Fig 5F on cells from the same plate, focusing on the window at which the opposing module repulsion is seen. The repulsion is maintained even when we observe only cells from a single plate:

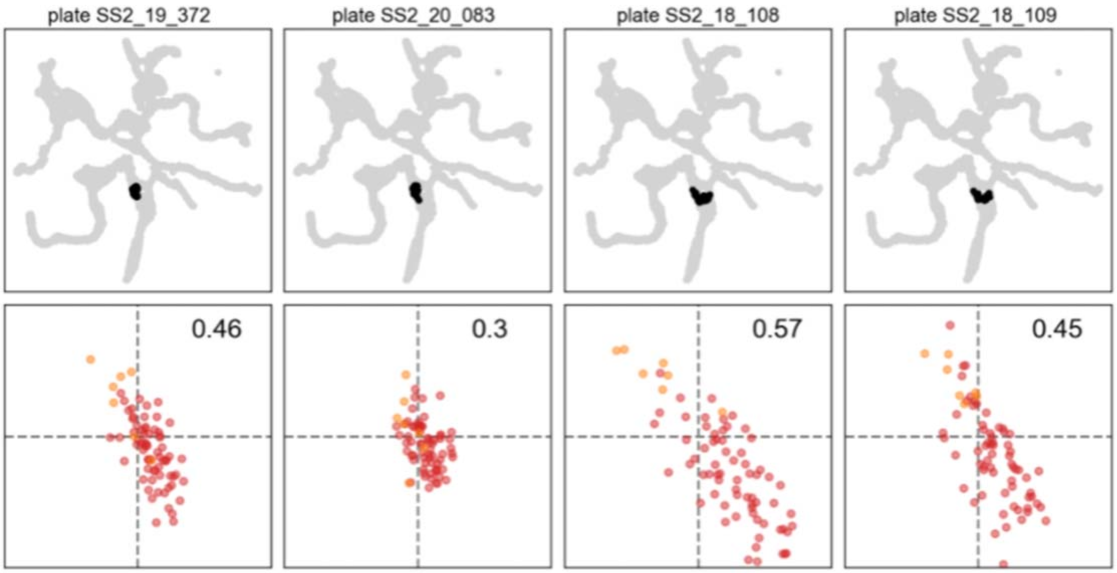

-

The effects of this mosaic of opposing modules in now explained in the text:

Lines 339-350: "Surprisingly, metaregulon 5 showed two peaks of activity, interrupted by the peak of the pro-neurogenic metaregulon 4 which resolved upon neuronal differentiation as the two lineages split, with Ets1 expression retained in SOX10+ SCPs but downregulated in SIX1+ sensory progenitors and neurons, as confirmed by experimental validations using immunofluorescence and RNAscope® (Fig 5D-F). Consistent with this, overexpression of neurogenic factor Six1 suppresses the expression of Ets1 in vitro (Hosseinipour 2017). The temporary downregulation of Ets1 metaregulon in the middle of the differentiation trajectory is due to mosaic compositional effects that result from a significant proportion of cells upregulating the activity of the neurogenic Six1 regulon and downregulating the activity of Ets1 regulon; other cells in the same location on the trajectory maintained high levels of Ets1 regulon without switching on Six1 regulon (and were intermixed with the first population) (Fig 5F)."

The authors have used data from human tumour samples and integrated / projected the cells on the mouse data. Can the authors comment on how they extracted the malignant cells from the data sets? Could they also include non-malignant cells to show the robustness of the embedding? Have the authors corrected for copy number variations in the tumours?

We thank the reviewer for pointing out the lack of precision in the process of selecting tumour cells for projection onto the mouse data. For the Melanoma dataset, we followed the same approach as the authors from the related publication, who were identifying tumour cells with MLANA, MITF, and DCT markers expression. In our case, we focused further on PRAME+ class 2 tumour cells from one single patient because we aimed at a big number of cells, while avoiding possible patient-wise batch effect (now explained in Methods, see "Comparison with uveal melanoma data", lines 1180-1206). For the Neuroblastoma datasets, the cells were already annotated as being tumour or non-tumour based on copy-number variations or specific phenotype. One selected patient additionally presented non-malignant Schwann cells and fibroblasts, which both perfectly aligned with our own annotated matching cell types (Appendix FigS15C, patient T19). We have not corrected for copy number variations in the tumours, as it is not a necessary step for integration and projection. Moreover, both original Melanoma and Neuroblastoma studies did not employ this approach thorough their own analysis of scRNAseq tumour data. Overall, we diminished the presentation and discussion of these data and keep them rather as a methodological example inspiring further research in this direction according to the requests of one of the reviewers.

Minor points

Due to the detailed view on the data set it is not easy to follow which cell type would correspond to which cluster in the central figure F1B. The naming is not consistent throughout the manuscript and should be clarified. One example (among many) relates to Figure 2A - here the SCPs relate to the "hub"/early SCPS while the previously assigned SCPs are called iSCs.

We agree that in the previous version of the manuscript there was some inconsistency between terminology and cell types. We are thankful for pointing this out. In the revised manuscript, we adhere to the use of uniform terminology.

Of course, sometimes the terms we use characterize progenitors of different stage and potency. For instance, we would like to clarify that in the case of bifurcation analysis, we select two endpoints upon fate commitment, but not final differentiation and adapt terms for such situations. For example, in Fig5 we use the general term "sympathoadrenal progenitors" since these cells are not yet chromaffin, sympathetic and "immature Schwann cells" on autonomic nerves. In a different situation, the general population of Schwann cell lineage in Fig 1B splits into subpopulations that are specifically examined in Fig7. In that case, the terms become uniformly based on specific gene expression patterns and spatial aspects such as nerve-association.

Additionally, the neural crest population is given twice in Figure 1B and split up in the supplements. It would be better to state at least one discriminating gene in the list in the figure.

- The two neural crest populations shown in the main and supplementary figures correspond to trunk and cranial neural crest populations discriminated by the presence or absence of mesenchymal progeny and expression of Hox genes. Following the reviewer's advice, we now provide key genes next to the population names in Fig 1B and the corresponding UMAPs (see Appendix FigS3). Also, we generated the online data-mining resource corresponding to our single cell atlas of the entire neural crest tree based on PAGODA browser visualizations (see data availability section). Checking this resource helps to understand the further spectrum of specific marker genes and differential gene expression for any of the subpopulations.

Figure 2B: What are the yellow and lilac curves? Developmental time? Needs a label.

This is correct, the curves ranging from bright yellow to purple reflect the actual developmental time. We inserted a key in the revised figure (now Fig 5B) to improve clarity of representation.

Typo in abstract - there is at least one word missing ("show an indistinguishable" XXX).

Corrected – lines 38-40: "“By using single-cell transcriptomics to generate the atlas of the entire neural crest lineage, we show that early SCPs and late migratory crest have similar transcriptional profiles..” (lines 38-40)."

On page 14 the wrong Figure is cited. (Fig 6A, I is given should be Figure 4?)

Figure 5B and 5C are not referenced in the text.

We acknowledge both mistakes and correct them. In the revised manuscript, figures were revised and their numbering was changed, and we hope they are referenced correctly throughout the text.

References

Adameyko, I., F. Lallemend, A. Furlan, N. Zinin, S. Aranda, S. S. Kitambi, A. Blanchart, R. Favaro, S. Nicolis, M. Lubke, T. Muller, C. Birchmeier, U. Suter, I. Zaitoun, Y. Takahashi and P. Ernfors (2012). "Sox2 and Mitf cross-regulatory interactions consolidate progenitor and melanocyte lineages in the cranial neural crest." Development **139**(2): 397-410.

Carr, M. J., J. S. Toma, A. P. W. Johnston, P. E. Steadman, S. A. Yuzwa, N. Mahmud, P. W. Frankland, D. R. Kaplan and F. D. Miller (2019). "Mesenchymal Precursor Cells in Adult Nerves Contribute to Mammalian Tissue Repair and Regeneration." Cell Stem Cell **24**(2): 240-256 e249.

Castro, R., T. Taetzsch, S. K. Vaughan, K. Godbe, J. Chappell, R. E. Settlage and G. Valdez (2020). "Specific labeling of synaptic schwann cells reveals unique cellular and molecular features." Elife **9**.

Coulpier, F., S. Le Crom, G. S. Maro, J. Manent, M. Giovannini, Z. Maciorowski, A. Fischer, M. Gessler, P. Charnay and P. Topilko (2009). "Novel features of boundary cap cells revealed by the analysis of newly identified molecular markers." Glia **57**(13): 1450-1457.

Hockman, D., I. Adameyko, M. Kaucka, P. Barraud, T. Otani, A. Hunt, A. C. Hartwig, E. Sock, D. Waithe, M. C. M. Franck, P. Ernfors, S. Ehinger, M. J. Howard, N. Brown, J. Reese and C. V. H. Baker (2018). "Striking parallels between carotid body glomus cell and adrenal chromaffin cell development." Dev Biol **444 Suppl 1**: S308-S324.

Joseph, N. M., Y. S. Mukouyama, J. T. Mosher, M. Jaegle, S. A. Crone, E. L. Dormand, K. F. Lee, D. Meijer, D. J. Anderson and S. J. Morrison (2004). "Neural crest stem cells undergo multilineage differentiation in developing peripheral nerves to generate endoneurial fibroblasts in addition to Schwann cells." Development **131**(22): 5599-5612.

Lukoseviciute, M., D. Gavriouchkina, R. M. Williams, T. Hochgreb-Hagele, U. Senanayake, V. Chong-Morrison, S. Thongjuea, E. Repapi, A. Mead and T. Sauka-Spengler (2018). "From Pioneer to Repressor: Bimodal foxd3 Activity Dynamically Remodels Neural Crest Regulatory Landscape In Vivo." Dev Cell **47**(5): 608-628 e606.

Mapps, A. A., M. B. Thomsen, E. Boehm, H. Zhao, S. Hattar and R. Kuruvilla (2022). "Diversity of satellite glia in sympathetic and sensory ganglia." Cell Rep **38**(5): 110328.

Richard, L., N. Vedrenne, J. M. Vallat and B. Funalot (2014). "Characterization of Endoneurial Fibroblast-like Cells from Human and Rat Peripheral Nerves." J Histochem Cytochem **62**(6): 424-435.

Simoes-Costa, M. and M. E. Bronner (2015). "Establishing neural crest identity: a gene regulatory recipe." Development **142**(2): 242-257.

Soldatov, R., M. Kaucka, M. E. Kastriti, J. Petersen, T. Chontorotzea, L. Englmaier, N. Akkuratova, Y. Yang, M. Haring, V. Dyachuk, C. Bock, M. Farlik, M. L. Piacentino, F. Boismoreau, M. M. Hilscher, C. Yokota, X. Qian, M. Nilsson, M. E. Bronner, L. Croci, W. Y. Hsiao, D. A. Guertin, J. F. Brunet, G. G. Consalez, P. Ernfors, K. Fried, P. V. Kharchenko and I. Adameyko (2019). "Spatiotemporal structure of cell fate decisions in murine neural crest." Science **364**(6444).

Tabula Muris, C., c. Overall, c. Logistical, c. Organ, processing, p. Library, sequencing, a. Computational data, a. Cell type, g. Writing, g. Supplemental text writing and i. Principal (2018). "Single-cell transcriptomics of 20 mouse organs creates a Tabula Muris." Nature **562**(7727): 367-372.

1st Revision - Editorial Decision 31st May 2022

Dear Igor and team,

Thank you for submitting your revised manuscript (EMBOJ-2021-108780R) to The EMBO Journal. Your amended study was sent back to two of the referees for their re-evaluation, and we have received comments from both of them, which I enclose below. As you will see, the reviewers stated that their issues have been comprehensively resolved and they are now broadly in favour of publication.

Thus, we are pleased to inform you that your manuscript has been accepted in principle for publication in The EMBO Journal.

We now need you to take care of a number of minor issues related to formatting and data presentation as detailed below, which should be addressed at re-submission.

Please contact me at any time if you have additional questions related to below points.

As you might have noted on our web page, every paper at the EMBO Journal now includes a 'Synopsis', displayed on the html and freely accessible to all readers. The synopsis includes a 'model' figure as well as 2-5 one-short-sentence bullet points that summarize the article. I would appreciate if you could provide this figure and the bullet points.

Thank you for giving us the chance to consider your manuscript for The EMBO Journal. I look forward to your final revision.

Again, please contact me at any time if you need any help or have further questions.

with
Best regards,

Daniel

Daniel Klimmeck PhD
Senior Editor
The EMBO Journal

Formatting changes required for the revised version of the manuscript:

>> Please add maximally five keywords to the manuscript.

>> Limit the number of authors listed in the references to maximally ten et al throughout main text and appendix.

>> Please provide Dataset EV1 as excel file.

>> Author contributions: CRediT has replaced the traditional author contributions section as of now because it offers a systematic machine readable author contributions format that allows for more effective research assessment. Please use the free text boxes beneath each contributing author's name to add specific details on the author's contribution. More information is available in our guide to authors.
https://www.embopress.org/page/journal/14602075/authorguide

>> Data Availability Section: Please recheck functionality of the PAGODA links.

Further information is available in our Guide For Authors: https://www.embopress.org/page/journal/14602075/authorguide

Use the link below to submit your revision:

https://emboj.msubmit.net/cgi-bin/main.plex

Referee #2:

The very careful and comprehensive revision of this paper has substantially improved the manuscript, and this both in the clarity of the text and also data wise. I highly appreciate that the authors took this reviewer's comments very seriously into consideration and made great efforts to address them both experimentally and in writing.
I am especially happy about how the authors were able to address my main point 13), in which I criticized the proposed "new path to myelination". The integration of more sequenced cells now actually shows that the first SC lineage to branch off are terminal SCs at the neuromuscular junction. This clarification is very important and of high interest to the scientific community. Likewise, it is nice and relevant how the authors take into account satellite glia and boundary cap cells (reviewer's point 14) and integrate the analysis of these glial subtypes into their study. Overall, I now fully support publication of this important and well-done paper.

Referee #4:

The authors have acquired additional experimental data as requested and have re-fined their analysis and statements accordingly. Recommended for publication.

2nd Revision - Editorial Decision 15th Jun 2022

Dear Dr Adameyko,

Thank you for submitting the revised version of your manuscript. I have now evaluated your amended manuscript and concluded that the remaining minor concerns have been sufficiently addressed.

Thus, I am pleased to inform you that your study has been accepted for publication in the EMBO Journal.

Please note that it is EMBO Journal policy for the transcript of the editorial process (containing referee reports and your response letter) to be published as an online supplement to each paper. I would thus like to ask for your consent on keeping the additional referee figures included in this file.

Also, in case you might NOT want the transparent process file published at all, you will also need to inform us via email immediately. More information is available here:
https://www.embopress.org/page/journal/14602075/authorguide#transparentprocess

----

Please note that in order to be able to start the production process, our publisher will need and contact you shortly regarding the page charge authorisation and licence to publish forms.

Authors of accepted peer-reviewed original research articles may choose to pay a fee in order for their published article to be made freely accessible to all online immediately upon publication. The EMBO Open fee is fixed at €5,500 EUR (+VAT where applicable).

We offer two licenses for Open Access papers, CC-BY and CC-BY-NC-ND.
For more information on these licenses, please visit:
http://creativecommons.org/licenses/by-nc-nd/3.0/deed.en_US

Notably, please be reminded that under the DEAL agreement of European scientific institutions with our publisher Wiley, you could be eligible for free publication of your article in the open access format. Please contact either the administration at your institution or Wiley (embojournal@wiley.com) to clarify further questions.

Should you be planning a Press Release on your article, please get in contact with embojournal@wiley.com as early as possible, in order to coordinate publication and release dates.

On a different note, I would like to alert you that EMBO Press is offering a new format for a video-synopsis of work published with us, which essentially is a short, author-generated film explaining the core findings in hand drawings, and, as we believe, can be very useful to increase visibility of the findings. This has proven to offer a nice opportunity for exposure i.p. for the first author(s) of the study. Please see the following link for representative examples and their integration into the article web page:
https://www.embopress.org/video_synopses
https://www.embopress.org/doi/full/10.15252/embj.2019103932

Please let me know, should you be interested to engage in commissioning a similar video synopsis for your work. According operation instructions are available and intuitive.

If you have any questions, please do not hesitate to call or email the Editorial Office.

Thank you for this contribution to The EMBO Journal and congratulations on a successful publication! Please consider us again in the future for your most exciting work.

with
Kind regards,

Daniel Klimmeck

Daniel Klimmeck, PhD
Senior Editor
The EMBO Journal
EMBO
Postfach 1022-40
Meyerhofstrasse 1
D-69117 Heidelberg
contact@embojournal.org
Submit at: http://emboj.msubmit.net

** Click here to be directed to your login page: https://emboj.msubmit.net

EMBO Press Author Checklist

	USEFUL LINKS FOR COMPLETING THIS FORM
Corresponding Author Name: Igor Adameyko	The EMBO Journal - Author Guidelines
Journal Submitted to: The EMBO Journal	EMBO Reports - Author Guidelines
Manuscript Number: EMBOJ-2021-108780	Molecular Systems Biology - Author Guidelines
	EMBO Molecular Medicine - Author Guidelines

Reporting Checklist for Life Science Articles (updated January 2022)
This checklist is adapted from Materials Design Analysis Reporting (MDAR) Checklist for Authors. MDAR establishes a minimum set of requirements in transparent reporting in the life sciences (see Statement of Task: 10.31222/osf.io/9sm4x). Please follow the journal's guidelines in preparing your manuscript.
Please note that a copy of this checklist will be published alongside your article.

Abridged guidelines for figures
1. Data
The data shown in figures should satisfy the following conditions:
- the data were obtained and processed according to the field's best practice and are presented to reflect the results of the experiments in an accurate and unbiased manner.
- ideally, figure panels should include only measurements that are directly comparable to each other and obtained with the same assay.
- plots include clearly labeled error bars for independent experiments and sample sizes. Unless justified, error bars should not be shown for technical replicates.
- if n<5, the individual data points from each experiment should be plotted. Any statistical test employed should be justified.
- Source Data should be included to report the data underlying figures according to the guidelines set out in the authorship guidelines on Data Presentation.

2. Captions
Each figure caption should contain the following information, for each panel where they are relevant:
- a specification of the experimental system investigated (eg cell line, species name).
- the assay(s) and method(s) used to carry out the reported observations and measurements.
- an explicit mention of the biological and chemical entity(ies) that are being measured.
- an explicit mention of the biological and chemical entity(ies) that are altered/varied/perturbed in a controlled manner.
- the exact sample size (n) for each experimental group/condition, given as a number, not a range;
- a description of the sample collection allowing the reader to understand whether the samples represent technical or biological replicates (including how many animals, litters, cultures, etc.).
- a statement of how many times the experiment shown was independently replicated in the laboratory.
- definitions of statistical methods and measures:
  - common tests, such as t-test (please specify whether paired vs. unpaired), simple χ2 tests, Wilcoxon and Mann-Whitney tests, can be unambiguously identified by name only, but more complex techniques should be described in the methods section;
  - are tests one-sided or two-sided?
  - are there adjustments for multiple comparisons?
  - exact statistical test results, e.g., P values = x but not P values < x;
  - definition of 'center values' as median or average;
  - definition of error bars as s.d. or s.e.m.

Please complete ALL of the questions below.
Select "Not Applicable" only when the requested information is not relevant for your study.

Materials

Newly Created Materials	Information included in the manuscript?	In which section is the information available? (Reagents and Tools Table, Materials and Methods, Figures, Data Availability Section)
New materials and reagents need to be available; do any restrictions apply?	Yes	Data availability and Code reproducibility

Antibodies	Information included in the manuscript?	In which section is the information available? (Reagents and Tools Table, Materials and Methods, Figures, Data Availability Section)
For **antibodies** provide the following information:<br>- Commercial antibodies: RRID (if possible) or supplier name, catalogue number and or/clone number<br>- Non-commercial: RRID or citation	Yes	STAR Methods

DNA and RNA sequences	Information included in the manuscript?	In which section is the information available? (Reagents and Tools Table, Materials and Methods, Figures, Data Availability Section)
**Short novel DNA or RNA including primers, probes**: provide the sequences.	Yes	STAR Methods

Cell materials	Information included in the manuscript?	In which section is the information available? (Reagents and Tools Table, Materials and Methods, Figures, Data Availability Section)
**Cell lines:** Provide species information, strain. Provide accession number in repository **OR** supplier name, catalog number, clone number, and/**OR** RRID.	Not Applicable	
**Primary cultures:** Provide species, strain, sex of origin, genetic modification status.	Not Applicable	
Report if the cell lines were recently **authenticated** (e.g., by STR profiling) and tested for mycoplasma contamination.	Not Applicable	

Experimental animals	Information included in the manuscript?	In which section is the information available? (Reagents and Tools Table, Materials and Methods, Figures, Data Availability Section)
**Laboratory animals or Model organisms:** Provide species, strain, sex, age, genetic modification status. Provide accession number in repository **OR** supplier name, catalog number, clone number, **OR** RRID.	Yes	STAR Methods
**Animal observed in or captured from the field:** Provide species, sex, and age where possible.	Not Applicable	
Please detail **housing and husbandry conditions**.	Yes	STAR Methods

Plants and microbes	Information included in the manuscript?	In which section is the information available? (Reagents and Tools Table, Materials and Methods, Figures, Data Availability Section)
**Plants:** provide species and strain, ecotype and cultivar where relevant, unique accession number if available, and source (including location for collected wild specimens).	Not Applicable	
**Microbes:** provide species and strain, unique accession number if available, and source.	Not Applicable	

Human research participants	Information included in the manuscript?	In which section is the information available? (Reagents and Tools Table, Materials and Methods, Figures, Data Availability Section)
If collected and within the bounds of privacy constraints report on age, sex and gender or ethnicity for all study participants.	Not Applicable	

Core facilities	Information included in the manuscript?	In which section is the information available? (Reagents and Tools Table, Materials and Methods, Figures, Data Availability Section)
If your work benefited from core facilities, was their service mentioned in the acknowledgments section?	Not Applicable	

Design

Study protocol	Information included in the manuscript?	In which section is the information available? (Reagents and Tools Table, Materials and Methods, Figures, Data Availability Section)
If study protocol has been **pre-registered, provide DOI in the manuscript**. For clinical trials, provide the trial registration number **OR** cite DOI.	Not Applicable	
Report the **clinical trial registration number** (at ClinicalTrials.gov or equivalent), where applicable.	Not Applicable	

Laboratory protocol	Information included in the manuscript?	In which section is the information available? (Reagents and Tools Table, Materials and Methods, Figures, Data Availability Section)
Provide DOI OR other citation details if **external detailed step-by-step protocols** are available.	Yes	

Experimental study design and statistics	Information included in the manuscript?	In which section is the information available? (Reagents and Tools Table, Materials and Methods, Figures, Data Availability Section)
Include a statement about **sample size** estimate even if no statistical methods were used.	Yes	STAR Methods
Were any steps taken to minimize the effects of subjective bias when allocating animals/samples to treatment (e.g. **randomization procedure**)? If yes, have they been described?	Not Applicable	
Include a statement about **blinding** even if no blinding was done.	Yes	STAR Methods
Describe **inclusion/exclusion criteria** if samples or animals were excluded from the analysis. Were the criteria pre-established?<br><br>If sample or data points were omitted from analysis, report if this was due to attrition or intentional exclusion and provide justification.	Not Applicable	
For every figure, are **statistical tests** justified as appropriate? Do the data meet the assumptions of the tests (e.g., normal distribution)? Describe any methods used to assess it. Is there an estimate of variation within each group of data? Is the variance similar between the groups that are being statistically compared?	Yes	STAR Methods

Sample definition and in-laboratory replication	Information included in the manuscript?	In which section is the information available? (Reagents and Tools Table, Materials and Methods, Figures, Data Availability Section)
In the figure legends: state number of times the experiment was **replicated** in laboratory.	Yes	Figure legends
In the figure legends: define whether data describe **technical or biological replicates**.	Yes	Figure legends

Ethics

Ethics	Information included in the manuscript?	In which section is the information available? (Reagents and Tools Table, Materials and Methods, Figures, Data Availability Section)
Studies involving **human participants**: State details of **authority granting ethics approval** (IRB or equivalent committee(s), provide reference number for approval.	Not Applicable	
Studies involving **human participants**: Include a statement confirming that **informed consent** was obtained from all subjects and that the experiments conformed to the principles set out in the WMA Declaration of Helsinki and the Department of Health and Human Services Belmont Report.	Not Applicable	
Studies involving **human participants:** For publication of **patient photos**, include a statement confirming that consent to publish was obtained.	Not Applicable	
Studies involving experimental **animals**: State details of **authority granting ethics approval** (IRB or equivalent committee(s), provide reference number for approval. Include a statement of compliance with ethical regulations.	Yes	STAR Methods
Studies involving **specimen and field samples:** State if relevant **permits** obtained, provide details of authority approving study; if none were required, explain why.	Not Applicable	

Dual Use Research of Concern (DURC)	Information included in the manuscript?	In which section is the information available? (Reagents and Tools Table, Materials and Methods, Figures, Data Availability Section)
Could your study fall under dual use research restrictions? Please check biosecurity documents and list of **select agents and toxins** (CDC): https://www.selectagents.gov/sat/list.htm	Not Applicable	
If you used a select agent, is the security level of the lab appropriate and reported in the manuscript?	Not Applicable	
If a study is subject to dual use research of concern regulations, is the name of the **authority granting approval and reference number** for the regulatory approval provided in the manuscript?	Not Applicable	

Reporting

The MDAR framework recommends adoption of discipline-specific guidelines, established and endorsed through community initiatives. Journals have their own policy about requiring specific guidelines and recommendations to complement MDAR.

Adherence to community standards	Information included in the manuscript?	In which section is the information available? (Reagents and Tools Table, Materials and Methods, Figures, Data Availability Section)
State if relevant guidelines or checklists (e.g., **ICMJE, MIBBI, ARRIVE, PRISMA**) have been followed or provided.	Not Applicable	
For **tumor marker prognostic studies,** we recommend that you follow the **REMARK** reporting guidelines (see link list at top right). See author guidelines, under 'Reporting Guidelines'. Please confirm you have followed these guidelines.	Not Applicable	
For **phase II and III randomized controlled trials**, please refer to the **CONSORT** flow diagram (see link list at top right) and submit the CONSORT checklist (see link list at top right) with your submission. See author guidelines, under 'Reporting Guidelines'. Please confirm you have submitted this list.	Not Applicable	

Data Availability

Data availability	Information included in the manuscript?	In which section is the information available? (Reagents and Tools Table, Materials and Methods, Figures, Data Availability Section)
Have **primary datasets** been deposited according to the journal's guidelines (see 'Data Deposition' section) and the respective accession numbers provided in the Data Availability Section?	Yes	Data availability and Code reproducibility
Were **human clinical and genomic datasets** deposited in a public access-controlled repository in accordance to ethical obligations to the patients and to the applicable consent agreement?	Not Applicable	
Are **computational models** that are central and integral to a study available without restrictions in a machine-readable form? Were the relevant accession numbers or links provided?	Yes	Data availability and Code reproducibility
If publicly available data were reused, provide the respective **data citations in the reference list**.	Yes	Reference list
